# Key Amino Acids Controlling pH Optima in Avian Chia Paralogs: Mechanistic Insights into Functional Divergence

**DOI:** 10.3390/molecules31060999

**Published:** 2026-03-16

**Authors:** Eri Tabata, Keita Suzuki, Yuki Suzuki, Kazuaki Okawa, Yuri Usui, Akinori Kashimura, Peter O. Bauer, Fumitaka Oyama

**Affiliations:** 1 Department of Chemistry and Life Science, Kogakuin University, Hachioji 192-0015, Japan; bd18004@g.kogakuin.jp (E.T.); st13796@ns.kogakuin.ac.jp (K.S.); s121039@g.kogakuin.jp (Y.S.); st13649@ns.kogakuin.ac.jp (K.O.); yuri1171999@gmail.com (Y.U.); bu41265@gmail.com (A.K.); 2 Bioinova a.s., Videnska 1083, 142 00 Prague, Czech Republic; peter.bauer@bioinova.cz

**Keywords:** acidic chitinase, amino acid substitutions, catalytic mechanism, Chia paralogs, chicken, enzyme structure-function relationship, glycoside hydrolase family 18, pH-dependent activity, residue pKa modulation, site-directed mutagenesis

## Abstract

Acidic chitinase (Chia) degrades chitin, a structural polysaccharide in insect exoskeletons, and plays important roles in omnivorous and insectivorous mammals and birds. In birds, gene duplications have generated multiple Chia paralogs with functional divergence, but the molecular basis for this diversification remains unclear. Here, we characterized three chicken Chia paralogs (Chia1–3) and identified distinct pH-dependent enzymatic profiles. Chia1 is enzymatically inactive but was captured by chitin-affinity resin despite lacking a canonical chitin-binding domain, suggesting residual substrate interaction through the catalytic domain or a non-catalytic role. Chia2 exhibits maximal activity at pH 2.0, whereas Chia3 peaks at pH 5.0 and displays broader activity. Exon swapping and site-directed mutagenesis identified residues 104 (Ala in Chia2, Asp in Chia3) and 269 (His vs. Asn) as key contributors to pH-dependent activity differences. Reciprocal substitutions shifted pH profiles accordingly. Structural modeling and computational pKa predictions suggested that D213 and residue 269 may function as a pKa-regulating module influencing catalytic ionization. Comparative sequence analysis revealed lineage-specific conservation of these residues, consistent with adaptive divergence. Our findings show that limited amino acid substitutions can markedly modify pH-dependent enzymatic activity, providing mechanistic insight into how local residue variation contributes to the functional diversification of duplicated genes.

## 1. Introduction

Chitin is a widely distributed structural polysaccharide composed of *N*-acetyl-D-glucosamine (GlcNAc) units linked by β-1,4-glycosidic bonds. It is a key component of the exoskeletons of insects and other invertebrates and is also present in the cell walls of fungi [1,2,3]. Although mammals do not synthesize chitin, they express chitin-degrading enzymes such as chitotriosidase (Chit1) and acidic chitinase (Chia; also known as acidic mammalian chitinase, AMCase), both of which belong to glycoside hydrolase family 18 (GH18) and exhibit robust chitinolytic activity [4,5,6,7,8].

Among these enzymes, Chia is particularly notable for its ability to function under highly acidic conditions, with an optimal pH of around 2.0, making it well-suited for activity in the gastric environment [7,9,10]. Structurally, Chia consists of an N-terminal catalytic domain (CatD) and a C-terminal chitin-binding domain (CBD) [7,11]. Its enzymatic activity is mediated by a conserved DXXDXE motif, in which the glutamate residue at the C-terminal position serves as the key catalytic residue [8,12,13,14].

Beyond its digestive role, Chia has also been implicated in immunomodulation and the treatment of some respiratory diseases. Genetic polymorphisms in the human *CHIA* gene are associated with asthma susceptibility and the altered expression of associated genes [15,16,17,18,19]. In mouse models, Chia deficiency leads to airway chitin accumulation, an enhanced inflammatory response, impaired tissue repair, and increased mortality following lung injury. Conversely, transgenic expression of Chia alleviates these pathologies, highlighting its dual role in digestive and respiratory homeostasis [20,21,22,23].

Recent studies have suggested that the evolutionary adaptation of Chia is associated with dietary specialization. Insectivorous and omnivorous mammals express high levels of Chia in the stomach. In contrast, expression is often reduced or lost in herbivores and carnivores, likely due to pseudogenization or inactivating amino acid substitutions [24,25,26]. Furthermore, the type 2 immune circuit in the stomach has been shown to coordinate chitin degradation with mucosal immunity, linking immune function to dietary niche [27].

The existence of multiple Chia paralogs across vertebrate lineages, including mammals and birds, suggests that repeated gene duplication events have been followed by functional divergence of this enzyme family [28,29]. In birds, the *Chia* gene family appears particularly expanded. For example, the chicken genome encodes three *Chia* paralogs (*Chia1*, *Chia2*, and *Chia3*) on the same chromosome. Chia2 is predominantly expressed in the glandular stomach, indicating its digestive role [30], but the physiological roles and biochemical properties of Chia1 and Chia3 remain largely uncharacterized.

In this study, we investigated the functional divergence of avian Chia paralogs using chicken as a representative model. We characterized their enzymatic activity profiles, pH optima, and domain structures, leading to the identification of two key amino acid residues that determine pH-dependent enzymatic activity among the three paralogs. Furthermore, through reciprocal site-directed mutagenesis and comparative sequence analysis across diverse bird species, we show that these residues are lineage-specific and conserved, suggesting that Chia paralogs have undergone evolutionary functional adaptation in response to ecological and dietary variation.

## 2. Results

### 2.1. Chia Paralogs Are Primarily Expressed in the Glandular Stomach of the Chicken

**Figure 1:**
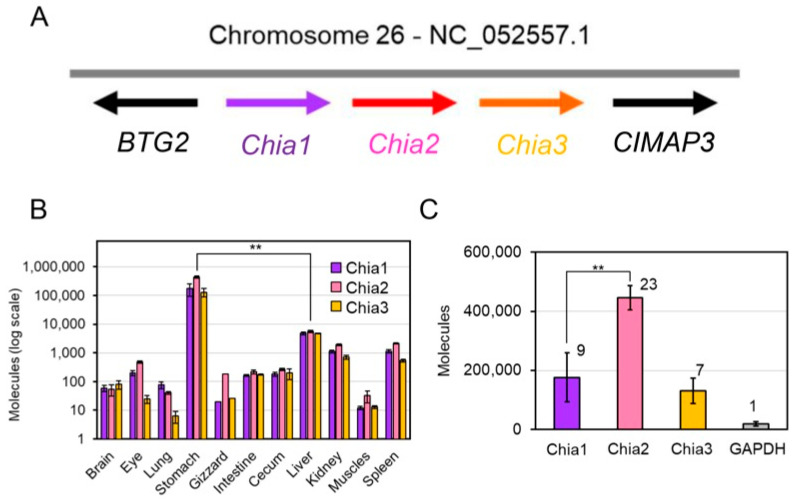
Chicken *Chia* paralog genes and their mRNA expression analysis in chicken tissues. (**A**) Schematic representation of the genomic location and classification of chicken *Chia* paralog genes on chromosome 26. The paralogs are designated as *Chia1*, *Chia2*, and *Chia3* based on the proximity to the marker genes *BTG2* and *CIMAP3*. (**B**) RT-qPCR determined the expression profiles of the Chia paralogs in various chicken tissues. Data show the highest expression in the glandular stomach, followed by the liver, kidney, and spleen, indicating tissue-specific expression patterns. (**C**) Comparative expression levels of the Chia paralogs to the housekeeping gene GAPDH in the glandular stomach. The numbers above the bars indicate the fold of the expression when GAPDH is set to 1. Data are presented as mean ± SD from at least three independent experiments. ** *p* < 0.01. *p*-values were determined using Welch’s *t*-test.

To investigate the tissue distribution of Chia paralogs in chicken, we analyzed the transcript levels of *Chia1*, *Chia2*, and *Chia3*, which are clustered on chromosome 26 (Figure 1A). Reverse transcription quantitative PCR (RT-qPCR) [30] was performed using RNA extracted from various chicken tissues.

The highest expression of all three paralogs was observed in the glandular stomach (proventriculus), with moderate to low expression in the liver, kidney, and spleen (Figure 1B). Notably, in the glandular stomach, transcript levels of all three *Chia* genes exceeded that of the housekeeping gene glyceraldehyde-3-phosphate dehydrogenase (GAPDH) (Figure 1C) [10], indicating their potentially indispensable physiological function.

These findings suggest that *Chia* paralogs are predominantly expressed in the glandular stomach and may play specific roles in gastric function. Detected transcripts in other tissues may also serve auxiliary or non-digestive roles depending on tissue type or physiological context. However, their biological significance remains to be elucidated.

### 2.2. Divergent Enzymatic Properties and pH Optima of the Chicken Chia Paralogs

**Figure 2:**
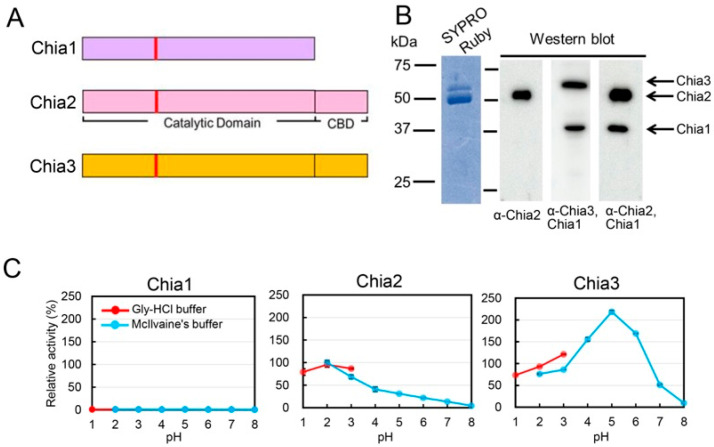
Protein expression analysis in chicken glandular stomach and enzymatic properties of Chia paralogs. (**A**) Diagram illustrating the domain composition of the Chia2 and Chia3 proteins, each featuring an N-terminal catalytic domain (CatD) and a C-terminal chitin-binding domain (CBD). Vertical bold red lines represent the active center of Chia. Chia1 lacks CBD, highlighting its unique structural variation. (**B**) (**Left**) SDS-PAGE analysis of proteins purified from the glandular stomach using chitin bead columns, stained with SYPRO Ruby. (**Right**) Western blot analysis using specific antibodies to detect the presence of the three Chia paralogs. The anti-Chia2 antibody showed a band at 52 kDa, anti-Chia1/Chia3 antibody identified bands at 55 and 37 kDa, and anti-Chia1/Chia2 antibody revealed bands at 52 and 37 kDa. (**C**) Enzymatic properties and pH optima of chicken Chia paralogs. All enzymatic activities were normalized to Chia2 activity at pH 2.0, which was defined as 100%. Data are presented as mean ± SD from at least three independent experiments.

Chia2 and Chia3 share evolutionarily conserved domain architecture, consisting of an N-terminal catalytic domain (CatD) and a C-terminal chitin-binding domain (CBD). In contrast, Chia1 lacks CBD (Figure 2A).

To evaluate endogenous protein expression, Chia proteins were purified from chicken glandular stomach extracts using chitin-affinity chromatography [30]. SDS-PAGE analysis of the purified fraction revealed two major bands at approximately 54 and 51 kDa along with minor bands around 37 kDa (Figure 2B, left). The individual Chia paralogs were detected by specific antibodies, with the Chia2 antibody having a signal at 52 kDa, and the Chia3/Chia1 antibody detecting bands at 55 and 37 kDa. The Chia2/Chia1 antibody generated signals at 52 and 37 kDa (Figure 2B, right). These comprehensive analyses confirm the expression and presence of all three Chia paralogs in the chicken glandular stomach. All three proteins were captured by chitin-affinity purification (Figure 2B). While this observation suggests a possible interaction with chitin, it does not exclude non-specific adsorption or residual substrate binding via the catalytic domain. Therefore, further functional analyses are required to clarify their intrinsic binding properties and physiological relevance.

To assess their enzymatic properties, recombinant Chia1–3 proteins were expressed in *Escherichia coli* [25] (Supplementary Figure S1). Despite high sequence similarity—91% identity within the aligned catalytic domain (excluding the CBD region) and 89% between Chia2 and Chia3, the paralogs exhibited distinct catalytic profiles (Figure 2C).

Chia2 showed maximal chitinolytic activity at pH 2.0, consistent with previous reports (Figure 2C, middle) [30]. In contrast, Chia3 exhibited peak activity at pH 5.0 and retained 50% of Chia2’s maximal activity at pH 7.0 (Figure 2C, right). Chia1, lacking the CBD, displayed virtually no catalytic activity (Figure 2C, left). Even so, Chia1 was detected in the chitin-bound fraction (Figure 2B), although the absence of a canonical CBD raises questions about the mechanism of this interaction.

These results indicate functional divergence among the chicken Chia paralogs. Chia2 primarily functions under highly acidic conditions, whereas Chia3 operates across a broader pH spectrum. Chia1, although enzymatically inactive under the assay conditions used, may retain biological relevance through potential substrate interactions, though its precise physiological role remains to be determined.

### 2.3. Exons 4 and 8 Govern the pH Optima of Chia2 and Chia3

**Figure 3:**
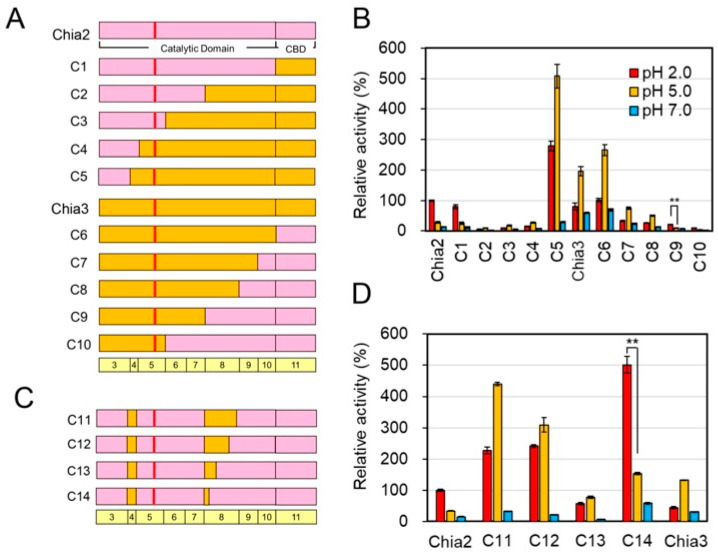
Role of exons 4 and 8 in determining the pH optima of Chia2 and Chia3. (**A**) Schematic representation of chimeric proteins (C1–C10) generated by exchanging segments between Chia2 and Chia3 to explore the regions governing pH optima shifts. The amino acid sequences are color-coded: pink for Chia2 and orange for Chia3. The size of each exon is shown above the schematic diagram indicating the exon number encoding the region. The coding region of the chicken Chia paralogs comprises exons 3–11. The schematic exon structures of the chimeras are shown above the figure. (**B**) Enzymatic activity profiles of chimeras C1 through C10 at different pH levels. (**C**) Diagram of chimeras C11, C12, C13, and C14, focusing on the roles of Chia3’s exon 4 and exon 8 in shifting Chia’s activity pH optimum. (**D**) Activity analysis of chimeras C11–C14. All enzymatic activities were normalized to Chia2 activity at pH 2.0, which was defined as 100%. Data are presented as mean ± SD from at least three independent experiments. ** *p* < 0.01. *p*-values were determined using Welch’s *t*-test.

As shown above, the pH optima of chicken Chia2 and Chia3 differed markedly. To identify the regions responsible for this divergence, we generated a series of chimeric constructs (C1–C10) by segmentally exchanging regions between Chia2 and Chia3 (Figure 3A; Supplementary Figure S2). When Chia3 sequences were introduced into the C-terminal region of Chia2, chimeras C2, C3, and C4 (Chia3 CBD and expanding portions of CatD) exhibited significantly reduced activity at pH 2.0 compared to wild-type Chia2, whereas C1 (Chia3 CBD) retained activity levels comparable to the Chia2 protein.

In contrast, C5 displayed a similar pH profile to wild-type Chia3; however, its absolute activity levels were approximately 2–3 fold higher at both pH values (Figure 3B; Supplementary Figure S3). Similarly, C7 and C8 showed pH 2.0:pH 5.0 activity ratios similar to Chia3, they retained 19–24% of Chia3 activity at pH 2.0 and 15–22% at pH 5.0, respectively. Despite decreasing activity with higher representation of the Chia2 sequence, these insertions did not perturb the pH profile of Chia3’s function. In contrast, chimeras C9 and C10 exhibited a shift in pH preference toward pH 2.0, although their absolute activities were reduced (12–25% of wild-type levels). Comparison among constructs C4–C10 revealed that replacement of exon 8 alone was insufficient to alter the pH preference (C8), whereas combined incorporation of exon 4 and exon 8 sequences (C9 and C10) was associated with a shift in pH responsiveness, albeit with reduced catalytic efficiency. These observations suggest that both exon 4 and the central region of exon 8 contribute to the modulation of pH-dependent activity.

To directly test this hypothesis, we constructed chimera C11 by inserting exons 4 and 8 from Chia3 into the Chia2 backbone (Figure 3C). This chimera exhibited a clear shift in peak activity from pH 2.0 to pH 5.0 (Figure 3D; Supplementary Figure S3), supporting the role of these two exons as critical determinants of pH optima. To further narrow down the regions involved in pH responsiveness, we constructed additional chimeras (C12–C14) combining exon 4 from Chia3 with partial segments of exon 8 (Figure 3C; Supplementary Figure S2). Notably, chimeras C12 and C13 exhibited a clear shift in optimum pH from 2.0 to 5.0. In contrast, C14, which included only the N-terminal portion of Chia3 exon 8, retained high activity at pH 2.0. These results suggest that the central region of exon 8 plays a critical role in modulating enzymatic activity in response to pH variation—i.e., in determining pH responsiveness (Supplementary Figure S4).

### 2.4. The Critical Role of Residues 104 and 269 in Determining pH Optima of Chia2 and Chia3

To further examine the functional importance of residues 104 and 269, we performed site-directed mutagenesis experiments (Supplementary Text S1; Supplementary Figure S5). The H269N substitution shifted the pH preference toward a more neutral pH range (pH 5–6), whereas A104D alone had little effect on activity. The A104D/H269N double mutant exhibited enhanced activity at pH 5.0. These preliminary analyses suggested that residues 104 and 269 may cooperatively influence pH-dependent activity.

**Figure 4:**
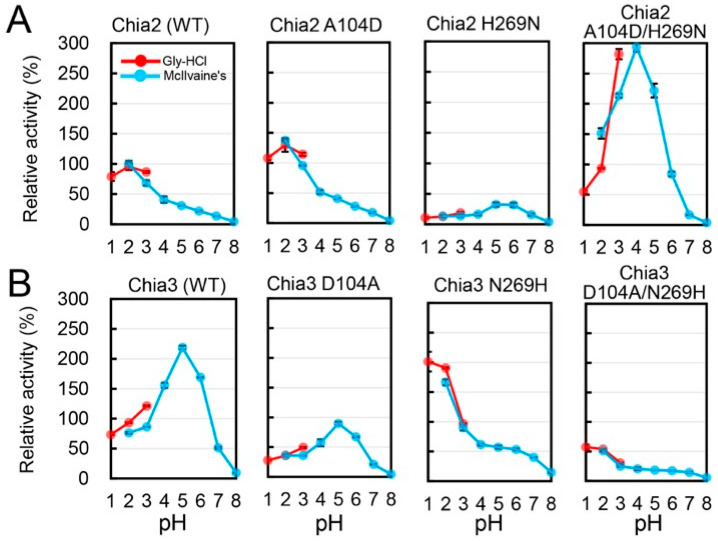
Impact of amino acids at positions 104 and 269 on the pH optima of Chia2 and Chia3. (**A**) Enzymatic activity of Chia2 mutants with and without A104D and/or H269N substitutions. (**B**) Enzymatic activity of Chia3 mutants with and without D104A and/or N269H introduced into its sequence. All enzymatic activities were normalized to Chia2 activity at pH 2.0, which was defined as 100%. Data are presented as mean ± SD from at least three independent experiments.

Next, we tested the functional importance of residues 104 and 269 in the enzymatic profiles of Chia2 and Chia3. The introduction of A104D alone into Chia2 had little impact on its activity at pH 5.0 (Figure 4A; Supplementary Figure S6). In contrast, the H269N substitution generally reduced enzyme activity, particularly at acidic conditions; however, it importantly shifted the optimum pH to 5.0–6.0. The A104D/H269N double mutant exhibited a robustly enhanced activity, especially at pH 5.0, indicating a synergistic effect of these two substitutions in shifting Chia2’s optimal pH (Figure 4A; Supplementary Figure S7).

We then explored whether reciprocal mutations in Chia3 could confer Chia2-like enzymatic properties. The D104A mutation alone did not affect the pH preference, whereas N269H increased the activity at pH 2.0 and reduced it at pH 5.0 (Figure 4B; Supplementary Figure S7). Strikingly, the D104A/N269H double mutant exhibited an activity profile closely resembling that of Chia2, indicating that residues 104 and 269 are key determinants of pH-dependent enzymatic activity.

### 2.5. Mechanistic Basis for pH Tuning by Residues 104 and 269

The biochemical analyses (Figure 2, Figure 3 and Figure 4) demonstrated that double substitutions at positions 104 and 269 markedly altered the pH optima of both Chia2 and Chia3. To gain insight into the molecular basis of this shift, we generated structural models using the AlphaFold Server (powered by AlphaFold 3) and estimated pKa values of ionizable residues with PROPKA3 [31,32,33,34,35]. Because experimental structures are not available for chicken Chia paralogs, these structure- and pKa-based results were used here as a qualitative guide. Among the predicted pKa values, D213 showed the largest and most consistent shift, and its direction matched the observed direction of the pH-optimum changes (Table 1). D213 is a conserved, catalytically essential residue located at the substrate-binding site. In Chia2, its predicted pKa increased from 2.98 to 5.25 upon A104D/H269N substitutions, whereas in Chia3, it decreased from 5.14 to 2.85 upon D104A/N269H mutations.

This trend is consistent with previous structural studies of acidic mammalian chitinase (AMCase; here referred to as Chia), in which H269 is positioned to form a hydrogen bond with D213 and thereby influence the ionization state of D213 and pH-dependent activity [36]. Together with the mutational data, these observations suggest that residue 269 (H or N) may contribute to pH tuning through its interaction with D213 (Figure 4).

**Figure 5:**
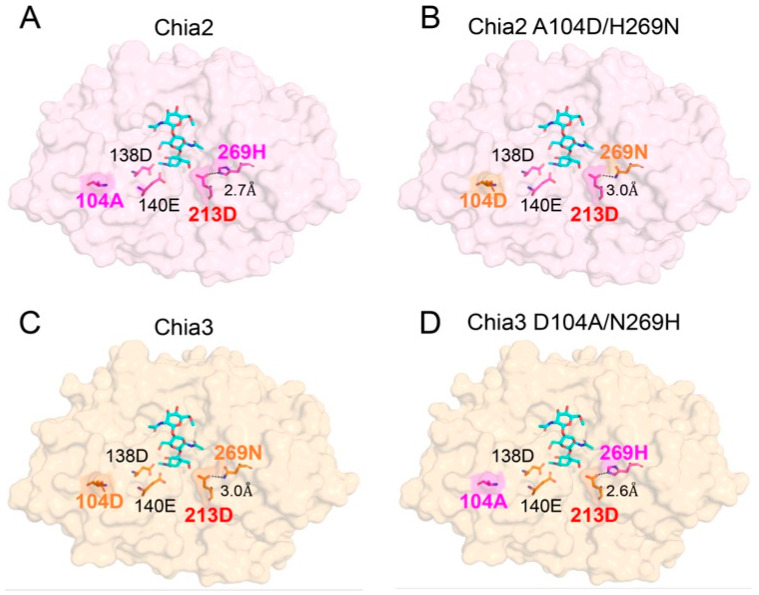
Structural analysis of Chia2 and Chia3 variants based on AlphaFold-predicted models. Close-up views of the catalytic sites are shown for four chitinase variants: (**A**) Chia2 WT, (**B**) Chia2 A104D/H269N, (**C**) Chia3 WT, and (**D**) Chia3 D104A/N269H. The positions of residues 104 and 269 are highlighted, along with their spatial relationship to the catalytic residues D138 and E140 within the conserved DXXDXDXE motif. Hydrogen bond distances between residue 269 and D213 are indicated. Allosamidin, a chitinase inhibitor, was superimposed from the human AMCase structure (PDB ID: 3FY1) for visualization only to indicate the approximate location of the substrate-binding cleft relative to the catalytic center; it was not used for model building. Color coding: magenta, Chia2-derived residues; orange, Chia3-derived residues; cyan, allosamidin.

To investigate the structural basis for these pKa shifts, we mapped the relevant residues onto AlphaFold-predicted models of four Chia variants (Figure 5). The model showed that in Chia2 WT (Figure 5A), A104 is located near the catalytic pair D138 and E140 within the conserved DXXDXDXE motif but does not form direct hydrogen bonds. Nevertheless, its position in the vicinity suggests a possible effect on the electrostatic microenvironment of the catalytic site. H269 formed a hydrogen bond with D213, with a donor–acceptor distance of approximately 2.7 Å, supporting a direct interaction influencing D213’s ionization state.

In the A104D/H269N double mutant of Chia2 (Figure 5B), the substituted D104 remained near D138 and E140 with no new hydrogen bond formation. However, the introduction of a negative charge at position 104 may alter the local electrostatics. In contrast, the H269N substitution increased the distance to D213 from 2.7 Å to approximately 3.0 Å, suggesting a weakened hydrogen bond, potentially destabilizing D213’s protonation state and contributing to the observed shift in pKa and pH optimum.

We next examined the corresponding structures in Chia3 (Figure 5C,D). In the WT (Figure 5C), D104 occupies a position similar to that of D104 in the Chia2 mutant (again near D138 and E140) with no additional hydrogen bonds. N269 forms a hydrogen bond with D213 at a distance of approximately 3.0 Å. In the D104A/N269H double mutant (Figure 5D), His269 restores the hydrogen bond with D213 and shortens the distance to ~2.6 Å, closely resembling the interaction observed in Chia2 WT. This restoration likely contributes to the re-establishment of a lower pKa and pH optimum.

In summary, the structural observations reinforce the scheme of residues 104 and 269 modulating the local electrostatic environment of the active site and influencing the protonation state of D213. In particular, residue 269, through its interaction with D213, appears to function as part of a pKa-regulating module that fine-tunes pH sensitivity in avian chitinase paralogs.

### 2.6. Molecular Evolution of Chia Paralog Genes in Birds

**Figure 6:**
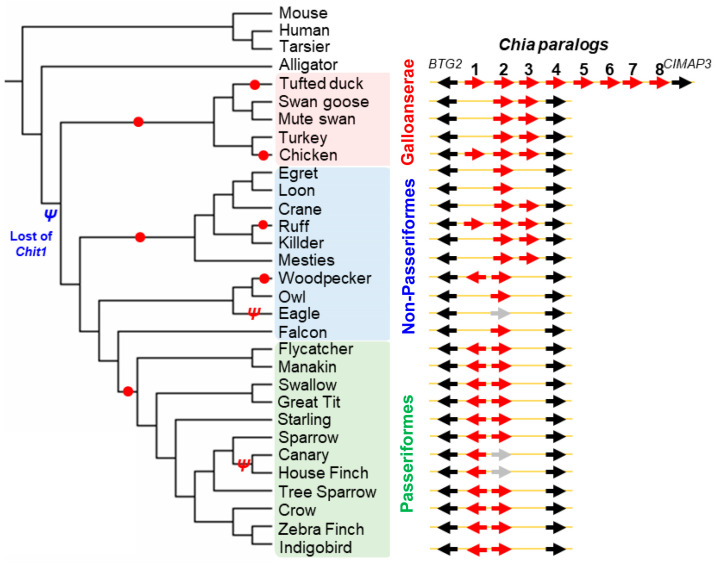
Phylogenetic analysis and evolutionary history of *Chia* paralog genes in birds. A phylogenetic tree constructed using *Chia* gene sequences illustrates the evolutionary relationships among avian *Chia* paralogs, with mammalian and alligator sequences included as outgroups for comparison. (**Left**) Closed red circles highlight lineage-specific Chia clades. *Ψ* indicates clades where *Chia* pseudogenization is expected to have occurred. (**Right**) Genomic organization and copy number variation of *Chia* genes are shown for representative bird species. Arrows indicate *Chia* paralogs (red), *Chia* pseudogenes (light gray), and neighboring marker genes *BTG2* and *CIMAP3* (black). The numbers represent the count of *Chia* genes adjacent to the *BTG2* gene. Notable examples include: tufted duck (*Aythya fuligula*, Anseriformes, Galloanserae) with up to eight *Chia* paralogs due to multiple duplication events; chicken (*Gallus gallus*, Galliformes, Galloanserae) with two or three paralogs; and barn swallow (*Hirundo rustica*, Passeriformes) with two *Chia* genes, one of which is encoded in reverse orientation, indicative of a lineage-specific genomic rearrangement.

To elucidate the evolutionary origins and diversification of *Chia* paralogs in birds, we constructed a phylogenetic tree covering a broad range of avian species using data from the NCBI Gene database (Figure 6; Supplementary Data file). The analysis revealed that avian *Chia* genes are generally located within a conserved genomic region flanked by *BTG2* and *CIMAP3* (*PIFO*) and that they form a divergent, bird-specific monophyletic clade distinct from the *Chia* genes of mammals (human, tarsier, mouse) and reptiles (alligator) (Figure 6, left).

Lineage-specific gene duplication events are particularly pronounced in certain avian clades and differ even within major groups. For instance, the tufted duck (*Aythya fuligula*), a representative of the Anseriformes order within Galloanserae, possesses up to eight *Chia* paralogs (Figure 6, right), suggesting at least seven duplication events. In contrast, Galliformes (e.g., chicken), another order within Galloanserae, retained two to three *Chia* paralogs, likely through two successive duplication events. Meanwhile, Passeriformes exhibited a distinct pattern, encoding only two *Chia* paralogs, one of which was arranged in the reverse orientation, indicating a lineage-specific genomic rearrangement (Figure 6, right).

These patterns suggest that *Chia* gene expansion occurred independently across avian lineages, shaped by shared and lineage-specific selective pressures potentially linked to dietary habits and ecological specialization.

### 2.7. Functional Diversity of Chia Paralogs in Birds

Avian *Chia* paralogs typically comprise 11 coding exons and include a conserved catalytic domain (CatD) and a chitin-binding domain (CBD). While this structural conservation implies shared enzymatic potential, sequence comparison revealed notable divergence at two key residues at positions 104 and 269 that modulate pH-dependent activity in chicken Chia2 and Chia3 (Figure 2, Figure 3 and Figure 4).

**Figure 7:**
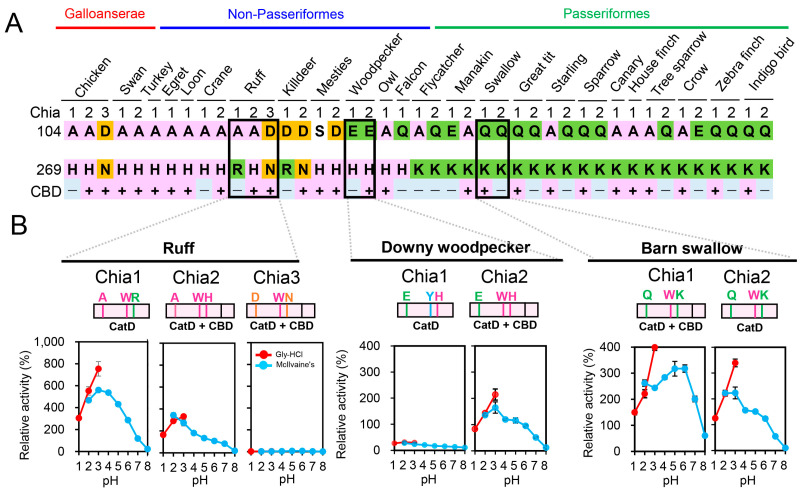
Functional diversity of Chia paralogs in birds highlighted by chitinase activity and amino acid substitutions. (**A**) The diagram illustrates the relationship between enzyme structure and function, specifically highlighting two critical residues (104 and 269) that influence the pH optima of chicken Chia paralogs. Residues are color-coded as follows: high conservation across birds (pink); high conservation in Passeriformes (green); residues matching those conserved in chicken Chia3 (orange). *Chia* genes in each bird species were numbered based on their chromosomal location in Figure 6. (**B**) Comparative enzymatic activity profiles of Chia paralogs from various bird species, ruff (*Calidris pugnax*), downy woodpecker (*Dryobates pubescens*), and barn swallow (*Hirundo rustica*) Chia paralogs, emphasizing the diversity in activity based on amino acid configurations at positions 104 and 269. Data are presented as mean ± SD from at least three independent experiments.

Amino acid combinations at these positions varied across species and paralogs (Figure 7A). The 104A/269H combination, which conferred maximal activity at pH 2.0 in chicken Chia2, was most widely found in many Galloanserae and non-Passeriformes. The 104D/269N combination, which conferred maximal activity at pH 5.0 in chicken Chia3, was frequently observed in diverse avian taxa, suggesting convergent adaptation to moderately acidic digestive environments.

To assess the functional impact of these variations, we expressed Chia paralogs from several representative bird species. In the ruff (*Calidris pugnax*; non-Passeriformes), Chia1 (A104/R269) exhibited peak activity at pH 3–4 (Figure 7A,B, left; Supplementary Figure S8), slightly resembling chicken Chia3. Ruff Chia2 (A104/H269) showed an acidic activity profile comparable to chicken Chia2. In contrast, ruff Chia3 (D104/N269) displayed low overall activity but retained a pH optimum near 5.0, similar to chicken Chia3.

In the downy woodpecker (*Dryobates pubescens*; non-Passeriformes), Chia1 and Chia2 harbored the E104/H269 combination, while the woodpecker Chia1 displayed approximately half of the activity of chicken Chia2, indicating that additional sequence features beyond residues 104 and 269 influence the catalytic efficiency of these enzymes (Figure 7A,B, middle; Supplementary Figure S8).

In the barn swallow (*Hirundo rustica*; Passeriformes), Chia1 and Chia2 carried the Q104/K269 combination (Figure 7A and Supplementary Figure S8). The swallow Chia1 showed high activity at a broad pH range (2.0 to 6.0), while Chia2 was predominantly active only at pH 2.0 and 3.0. These results suggest that the Q104/K269 combination confers functional versatility across a wide pH spectrum (Figure 7B, right).

Our findings indicate that avian *Chia* paralogs have undergone functional diversification following gene duplication, largely driven by residue-level substitutions. Based on activity profiles, the paralogs can be classified into three broad categories: (1) enzymes with high activity under strongly acidic conditions (e.g., chicken Chia2-like); (2) enzymes with moderate activity over a broader pH range (e.g., chicken Chia3-like); (3) paralogs with low or undetectable activity with presumably non-catalytic functions.

These functional shifts illustrate how discrete amino acid changes can reshape enzymatic properties and contribute to the adaptive evolution of gene families in response to ecological and dietary pressures.

## 3. Discussion

Chitin is one of the most abundant biopolymers in nature, and chitinases play essential roles in its degradation across animal taxa. In mammals and birds, acidic chitinase (Chia) has adapted to function in acidic environments such as the stomach, contributing to digestion and mucosal immunity [24,25,26,27,30,37,38]. While most vertebrates possess a single *Chia* gene, certain avian lineages harbor multiple paralogs [28,29], suggesting lineage-specific gene expansion and functional diversification.

In this study, we show that three chicken Chia paralogs exhibit distinct pH-dependent enzymatic profiles. Through chimeric analysis and reciprocal mutagenesis, we identified residues 104 and 269 as major contributors to these differences. Reciprocal double substitutions substantially shifted pH-dependent activity profiles, indicating that limited amino acid substitutions can markedly influence enzymatic behavior.

Structural modeling and computational pKa estimation suggested that D213 may act as a key residue linking substitutions at positions 104 and 269 to altered pH sensitivity. In particular, residue 269 appears to influence the electrostatic environment surrounding D213, consistent with previously reported structural observations in acidic mammalian chitinase [36]. However, because these interpretations rely on predicted structural models, further experimental structural analyses will be required to validate the proposed mechanism.

A recent study has highlighted the critical role of the DXXDXDXE motif in modulating catalytic activity under varying pH conditions in mouse Chia [39], further describing how specific residues can adapt enzyme function to acidic or neutral environments by modulating protonation dynamics. By integrating previously published mechanistic frameworks [36,39] with our structural modeling (Figure 5), we introduce a comprehensive perspective on the interplay between structural evolution and functional adaptation in Chia paralogs. This deeper understanding of pH specificity not only elucidates enzyme adaptation but also lays a conceptual foundation for engineering chitinases with tailored properties for biomedical and industrial applications [23,40].

Phylogenetic and comparative analyses across bird species revealed lineage-specific variation in residues 104 and 269. These patterns are consistent with functional diversification following gene duplication, potentially reflecting adaptation to different dietary or physiological contexts. Although residues 104 and 269 are strongly associated with pH-dependent activity differences, additional sequence features also contribute to catalytic efficiency across species.

Collectively, our findings illustrate how gene duplication combined with limited amino acid substitutions can generate functional diversity within enzyme families. This study provides a mechanistic framework for understanding the pH-dependent modulation of chitinase activity and provides a foundation for future studies investigating the in vivo roles and evolutionary trajectories of Chia paralogs.

## 4. Materials and Methods

### 4.1. RNA and cDNA Preparation

The Chicken Total RNA Panel (Zyagen, San Diego, CA, USA) was used to examine the distribution of Chia transcripts in various chicken tissues. Normal chicken glandular and gizzard stomachs were purchased from Funakoshi Co., Ltd. (Tokyo, Japan). These tissues had been dissected from white leghorn chickens, quickly frozen on dry ice, and stored at −80 °C by the supplier. No animal experiments were performed by the authors in this study. Total RNA was isolated from these tissues using TRIzol Reagent (Thermo Fisher Scientific, Waltham, MA, USA) according to the manufacturer’s instructions and reverse transcribed into cDNA essentially as described previously [30].

### 4.2. Selection of Primer Pairs for qPCR

Primers for qPCR were designed using PrimerQuest Input (Integrated DNA Technologies, Coralville, IA, USA), and their suitability was evaluated based on whether they gave single products, as reflected by a single melting temperature (Tm). PCR reactions (final volume 13 µL) were performed as described previously [30]. Melting curves were generated after amplification.

### 4.3. Construction of the DNA Standard and qPCR

Construction of the four genes’ standard DNA containing the Chia1, Chia2, Chia3, pepsinogen A, and GAPDH template DNA were synthesized and inserted into the pTAKN-2 vector by Eurofins Genomics (Tokyo, Japan). After ligation, these genes were amplified using the forward primer 5′-CTTTCAGACTGCAAAACC3′ and the reverse primer 5′-GTGGCAATAATGTCTACCT-3′. The standard DNA (491 bases; Supplementary Figure S9) was prepared by PCR reamplification from the plasmid DNA using the same primers and used as the standard DNA for qPCR afterward.

### 4.4. Glandular Stomach Extract Preparation

As described above, the soluble fraction was prepared from chicken glandular stomach tissues, except that the protease inhibitors were omitted in the homogenizing buffer. The homogenates were centrifuged at 15,000× *g* for 10 min at 4 °C, and the supernatants were used as chicken protein extract.

### 4.5. Antibody Preparation

Eurofins Genomics generated rabbit polyclonal antibodies specific to chicken Chia paralogs. Cys-peptides were conjugated through the added C-terminal or N-terminal cysteine to keyhole limpet hemocyanin (KLH). Sera from immunized rabbits were affinity-purified using the antigen with Cys (chicken Chia1, GQQSSSCLTN, Chia2, CVPPAQPNPPI, and Chia3, CRLQNPSNHGL) coupled to Sulfolink (Thermo Fisher Scientific). The specificity of each antibody was confirmed by Western blot.

### 4.6. SDS-Polyacrylamide Gel Electrophoresis and Western Blotting

The obtained protein fractions were analyzed using standard SDS-polyacrylamide gel electrophoresis (PAGE), followed by Western blot. Separated proteins were transferred to a polyvinylidene fluoride (PVDF) membrane (Immobilon-P, Merck Millipore, Burlington, MA, USA), which was probed with polyclonal anti-chicken Chia1, anti-chicken Chia2, and anti-chicken Chia3, followed by peroxidase-conjugated AffiniPure F (ab’)_2_ Fragment Donkey Anti-Rabbit IgG (H + L) (Jackson ImmunoResearch Laboratories, West Grove, PA, USA).

### 4.7. Construction of Chia Expression Vector

Coding regions of the mature form of chicken Chia1, Chia2, and Chia3 cDNAs were amplified from the chicken cDNA by PCR using KOD Plus DNA polymerase (Toyobo Co., Ltd., Osaka, Japan) and oligonucleotide primers (Eurofins Genomics) anchored with the restriction sites for EcoRI and XhoI (Supplementary Tables S1 and S2) as described previously [24]. Amplified cDNA was digested with EcoRI and XhoI and cloned into the same sites of the pEZZ18/pre-Protein A-Chia-V5-His. The entire nucleotide sequence of the resulting plasmid DNA (pEZZ18/Chia/V5-His) was confirmed by sequencing (Eurofins Genomics) [24]. Expressing this plasmid DNA in *E. coli* cells led to the production of the mature Protein A-Chia-V5-His (Supplementary Figure S1).

We expressed chicken Chia paralogs as recombinant fusion proteins with pre-Protein A (PA) and V5-His (pEZZ18/PA-chicken Chia-V5-His) [24]. In this report, PA-chicken Chia-V5-His and their derivative chimeric or mutant proteins were expressed by pET22b using the T7 promoter system (designated pET22b/Protein A-Chia-V5-His) as described recently [25].

The pEZZ18/PA-chicken Chia-V5-His was digested with EcoRI and XhoI and generated chicken Chia cDNA. Fragments were purified and subcloned into similarly digested pET22b/pre-Protein A-mouse Chia-V5-His to produce pET22b/pre-Protein A-chicken Chia-V5-His.

### 4.8. Construction of Expression Plasmids for Chimeric and Mutant Proteins and Preparation of Recombinant Proteins

Chicken Chia paralogs have similar exon structures at the nucleotide level, except for Chia1, which lacks exon 11. To create Chia2/Chia3 chimeric proteins, we fused two units at the junctions among exons 3–5, exons 6–7, exons 8–10, and exon 11 using template DNAs and primers (Supplementary Tables S1 and S2) as described previously [25]. More chimeras were also produced by combining templates and primers (Supplementary Tables S1 and S2). Chia mutant proteins were prepared by PCR using a template and primers (Supplementary Tables S1 and S2) [25].

*E. coli* BL21 (DE3) was transformed to express pre-Protein A-Chia-V5-His proteins using the plasmid DNAs. Transformed *E. coli* were grown in 250 mL of LB medium containing 100 µg/mL ampicillin at 37 °C for 18 h. After induction with 0.1 mM isopropyl β-D-thiogalactopyranoside (IPTG), the bacteria were cultured for two h in an LB medium. Cells were harvested by centrifugation at 6500× *g* for 20 min at 4 °C. The recombinant protein was prepared from *E. coli* and purified by IgG Sepharose (Cytiva, Marlborough, MA, USA) chromatography as described previously [25]. The protein-containing fractions were desalted using PD MidiTrap G-25 (GE Healthcare, Chicago, IL, USA) equilibrated with TS buffer [20 mM Tris-HCl (pH 7.6), 150 mM NaCl, and a protease inhibitor (Roche Diagnostics, Mannheim, Germany)].

### 4.9. Western Blot

All recombinant proteins contained a V5 epitope at their C-terminus. Recombinant proteins were detected by Western blot using an anti-V5-HRP monoclonal antibody (Thermo Fisher Scientific). The recombinant protein fractions were analyzed by standard SDS-polyacrylamide gel electrophoresis (PAGE), followed by Western blotting with the anti-V5-HRP monoclonal antibody. Immunoblot signals were visualized and quantified using a luminescent image analyzer (ImageQuant LAS 4000, Cytiva) according to the manufacturer’s instructions. Based on the quantified signals, equivalent amounts of recombinant proteins were used in the subsequent chitinase activity assays.

### 4.10. Chitinase Enzymatic Assays

Chitinolytic activity was measured using the synthetic fluorogenic substrate 4-methylumbelliferyl β-D-*N,N*′-diacetyl-chitobioside [4-MU-(GlcNAc)_2_] (Sigma-Aldrich, St. Louis, MO, USA) at a concentration of 100 μM. Each reaction was performed in triplicate. Enzymatic reactions were carried out in a final volume of 50 μL containing recombinant proteins expressed in *E. coli* and quantified by Western blot analysis. Reactions were conducted in McIlvaine’s buffer (0.1 M citric acid and 0.2 M Na_2_HPO_4_; pH 2.0–8.0) or 0.1 M Gly-HCl buffer (pH 1.0–3.0) at 37 °C for 30 min. Reactions were terminated by adding 20 μL of 1 M sodium carbonate. The fluorescence of released 4-methylumbelliferone was measured using a GloMax Discover Multimode Microplate Reader (Promega, Madison, WI, USA) with excitation at 365 nm and emission at 445 nm.

The substrate 4-MU-(GlcNAc)_2_ is widely used for chitinase activity assays and enables a sensitive and quantitative comparison of enzymatic activity among variants.

### 4.11. Statistical Analysis

Statistical analyses were performed using Welch’s *t*-test for pairwise comparisons. Data are presented as mean ± SD from at least three independent experiments. Differences were considered statistically significant at *p* < 0.01.

### 4.12. Structure Prediction and pKa Estimation

Structural models of chicken Chia variants (WT and mutants) were generated using the AlphaFold Server (powered by AlphaFold 3) with default settings. For each sequence, five models were generated using seed settings 1–5. The seed 1 model was used for structural visualization (Figure 5). The predicted structures (mmCIF) were converted to PDB format, and residue pKa values were estimated using PROPKA3 with default settings. For each residue, the pKa values reported in this study were calculated as the mean across the five seed models for the corresponding variant.

### 4.13. Sequence Analysis

We used annotated gene sequences available in GenBank. GenBank accession numbers and deduced *Chia* nucleotide sequences are described in Supplementary Table S3 and the Supplementary Data file. All sequences, including the mouse sequence as an outgroup, were imported into MEGA version 10 (MEGA X) [41] and aligned using the MUSCLE algorithm [42] implemented in MEGA. The evolutionary relationships of the *Chia* genes in Aves were estimated by the maximum-likelihood method.

### 4.14. Construction of a Phylogenetic Tree

The evolutionary history was inferred using the maximum likelihood method based on the Tamura–Nei model [43]. The tree with the highest log likelihood (−875.81) is shown. Initial tree(s) for the heuristic search were obtained automatically by applying neighbor-Joining and BioNJ algorithms to a matrix of pairwise distances estimated using the maximum composite likelihood (MCL) approach and then selecting the topology with superior log likelihood value. Evolutionary analyses were conducted in MEGA11 [44]. Sequences are listed in Supplementary Table S1 and the Supplementary Data file.

## Figures and Tables

**Table 1:** Predicted protein pKa values in amino acid residues in Chia paralogs and their mutants at positions 104 and 269.

	Chia2 Wild-Type	Chia2 A104N/H269N	Chia3 Wild-Type	Chia3 D104A/N269H
TYR 34	13.88	13.89	13.00	13.01
ARG 35	14.55	14.57	13.81	13.81
ASP 52	4.73	4.63		
TYR 56	18.29	18.20	16.87	16.89
TYR 70	6.63	6.58	5.69	5.70
TYR 77	15.67	15.64	14.62	14.64
GLU 114	3.62	3.78	3.76	3.60
ASP 133	3.11	3.12	3.21	3.24
ASP 136	2.98	2.92	2.57	2.57
ASP 138	7.88	8.00	8.13	8.12
GLU 140	8.75	8.83	8.84	8.94
TYR 141	16.49	16.40	15.25	15.26
LYS 145	8.46	8.88		
ARG 177	13.61	13.56	13.67	13.67
HIS 208	6.32	6.34	6.24	6.23
ASP 213	2.98	5.25	5.14	2.85
TYR 267	18.05	17.79	17.74	18.00
TYR 269	7.30			7.63
ARG 296	11.71	11.70		
GLU 297			5.76	5.54
TYR 303	13.06	13.03	12.62	12.66
GLU 305	5.06	5.05	4.53	4.58
ASP 319	5.79	5.83	6.07	6.10
TYR 336	12.50	12.40	13.75	13.77
LYS 450	10.49	10.49	10.49	10.51

## Data Availability

Data supporting the reported results will be available from the corresponding author (Fumitaka Oyama).

## Supplementary Materials

The following supporting information can be downloaded at: https://www.mdpi.com/article/10.3390/molecules31060999/s1, Figure S1: Deduced amino acid sequences of the recombinant Chia paralogs expressed in *E. coli*; Figure S2: Deduced amino acid sequences of the recombinant Chia proteins expressed in *E. coli*; Figure S3: Western blot analysis of the recombinant proteins, related to Figure 3; Figure S4: Amino acid sequence of the central region of exon 8 plays a crucial role in determining the pH-related enzymatic activity; Text S1: Amino Acids at Positions 104 and 269 Contribute to the Determination of the pH Optimum; Figure S5: Identification of key amino acids affecting pH optima of Chia2 and Chia3; Figure S6: Deduced amino acid sequences of the mutant Chia paralogs expressed in *E. coli*; Figure S7: Western blot analysis of recombinant proteins related to Figure 4; Figure S8: Deduced amino acid sequences of the recombinant Chia proteins expressed in *E. coli*; Figure S9: Single standard DNA molecule used for qPCR; Table S1: NCBI Gene Accession numbers of Chia sequences; Table S2: Construction of chimeric and mutant proteins by PCR; Table S3: Primers for construction of chimeric and mutant proteins; Supplementary Data file.
