# Supplementary material for: Key Amino Acids Controlling pH Optima in Avian Chia Paralogs: Mechanistic Insights into Functional Divergence
Source: Molecules. 2026 Mar 16;31(6):999. doi: 10.3390/molecules31060999 (PMC13029220; doi:10.3390/molecules31060999)
Supplement: Supplementary file 1 [file molecules-31-00999-s001.zip › molecules-4170619-supplementary.pdf]

Chicken Chia1

YVLSCYFTNWAQYRPGSGRFTVDNIDPCLCDHLIYAFAGMSNNEITTIEWNDVTLYKSFNGLKNQNG
NLKTLLAIGGWNFGTAKFSTMVSTPENRQTFINSVIKFLRQYQFDGLDIDWEYPGSRGSPSQDKGLF
TVLVQEMLAAFEQEAKQVNKPRLMITAAVAAGLSNIQAGYQIAELGKYLDYFHVMTYDFYSSGDGQT
GENSPLYSGNNVYLSVDYAMNYWKSNGAPAEKLLVGFPTYGHSFNLQNPSNTAVGAPTSGPGPAGPY
TKEAGLLAYYEICTFLDSGATQAWDAPQDVPYAYKSSEWVGYDNIKSFNIKIDWLKKNNYGGAMVWT
LDLDDFTGTFCKQGKYPLITTLKNALGQQSSSCLTN

Chicken Chia2

YVLSCYFTNWAQYRPGLGKYMPDNIDPCLCDHLIYAFAGMSNNEITTYEWNDETLYKSFNGLKNQNG
NLKTLLAIGGWNFGTAKFSTMVSTPENRQTFINSVIKFLRQYQFDGLDIDWEYPGSKGSPSQDKGLF
TVLVQEMLAAFEQEAKQVNKPRLMITAAVAAGLSNIQAGYQIAELGKYLDYFHVMTYDFHGSWDGQT
GENSPLYKGPADTGDLIYFNVDYAMNYWKSNGAPAEKLLVGFPTYGHSYILKNPSDTAVGAPTSGPG
PAGPYTRQSGFLAYYEICTFLDSGATQAWDAPQDVPYAYKSSEWVGYGNIKSFNIKIDWLKKNNYGG
AMVWSLDMDDFTGTFCKQGKYPLITTLKNALGQQSSSCVPPAQPNPPITAAPSTGSGSGSGSGSGSS
GSNTGSSGGSGFCAGKANGIYADPTNKSKFYNCNNGETFVQSCQAGLVFDSSCSCCNWA

Chicken Chia3

YVLSCYFTNWAQYRPGVGSFMPDNIDPCLCTHLLYAFAGMSNNEITTIEWNDVTLYKSFNGLKNQNK
NLKTLLSIGGWNFGTDKFSTMVSTPENRQTFINSVIKFLRRYQFDGLDIDWEYPGSRGSPSQDKGLF
TVLVQEMLAAFEQEAKQVNKPRLMITAAVAAGLSNIQAGYQIAELGKYLDYFHVMTYDFHGSWDTQT
GENSPLYQGPSDTGDNIYFNVDYAMNYWKSNGAPAEKLVVGFPAYGNTFRLQNPSNHGLGAPTSGPG
PAGPYTQEAGTLAYYEICTLLNSGGTQVWDAPQDVPYAYKGSTWVGYDNIKSFNIKADWLKKNNYGG
AMVWAIDLDDFTGTFCKQGKYPLITTLKNALGQQSNSCVPSTQPSPTTTAVPCNTHGSGSGSESSGS
NTDSSGESGFCAGKANGIYADPTNKSKFYNCNNGETFAQSCQAGLVFDSSCSCCNWA

Figure S1. Deduced amino acid sequences of the recombinant Chia paralogs expressed in *E. coli*. The amino acid sequences are color-coded, consistent with Figs. 1 and 2. Purple, chicken Chia1; pink, chicken Chia2; yellow, chicken Chia3.

Chia2/Chia3 Chimera C1
YVLSCYFTNWAQYRPGLGKYMPDNIDPCLCDHLIYAFAGMSNNEITTYEWNDETLYKSFNGLKNQNG
NLKTLLAIGGWNFGTAKFSTMVSTPENRQTFINSVIKFLRQYQFDGLDIDWEYPGSKGSPSQDKGLF
TVLVQEMLAAFEQEAKQVNKPRLMITAAVAAGLSNIQAGYQIAELGKYLDYFHVMTYDFHGSWDGQT
GENSPLYKGPADTGDLIYFNVDYAMNYWKSNGAPAEKLLVGFPTYGHSYILKNPSDTAVGAPTSGPG
PAGPYTRQSGFLAYYEICTFLDSGATQAWDAPQDVPYAYKSSEWVGYGNIKSFNIKIDWLKKNNYGG
AMVWSLDMDDFTGTFCKQGKYPLITTLKNALGQQSSSCVPSTQPSPTTTAVPCNTHGSGSGSESSGS
NTDSSGESGFCAGKANGIYADPTNKSKFYNCNNGETFAQSCQAGLVFDSSCSCCNWA

Chia2/Chia3 Chimera C2
YVLSCYFTNWAQYRPGLGKYMPDNIDPCLCDHLIYAFAGMSNNEITTYEWNDETLYKSFNGLKNQNG
NLKTLLAIGGWNFGTAKFSTMVSTPENRQTFINSVIKFLRQYQFDGLDIDWEYPGSKGSPSQDKGLF
TVLVQEMLAAFEQEAKQVNKPRLMITAAVAAGLSNIQAGYQIAELGKYLDYFHVMTYDFHGSWDGQT
GENSPLYKGPADTGDLIYFNVDYAMNYWKSNGAPAEKLVVGFPAYGNTFRLQNPSNHGLGAPTSGPG
PAGPYTQEAGTLAYYEICTLLNSGGTQVWDAPQDVPYAYKGSTWVGYDNIKSFNIKADWLKKNNYGG
AMVWAIDLDDFTGTFCKQGKYPLITTLKNALGQQSNSCVPSTQPSPTTTAVPCNTHGSGSGSESSGS
NTDSSGESGFCAGKANGIYADPTNKSKFYNCNNGETFAQSCQAGLVFDSSCSCCNWA

Chia2/Chia3 Chimera C3
YVLSCYFTNWAQYRPGLGKYMPDNIDPCLCDHLIYAFAGMSNNEITTYEWNDETLYKSFNGLKNQNG
NLKTLLAIGGWNFGTAKFSTMVSTPENRQTFINSVIKFLRQYQFDGLDIDWEYPGSKGSPSQDKGLF
TVLVQEMLAAFEQEAKQVNKPRLMITAAVAAGLSNIQAGYQIAELGKYLDYFHVMTYDFHGSWDTQT
GENSPLYQGPSDTGDNIYFNVDYAMNYWKSNGAPAEKLVVGFPAYGNTFRLQNPSNHGLGAPTSGPG
PAGPYTQEAGTLAYYEICTLLNSGGTQVWDAPQDVPYAYKGSTWVGYDNIKSFNIKADWLKKNNYGG
AMVWAIDLDDFTGTFCKQGKYPLITTLKNALGQQSNSCVPSTQPSPTTTAVPCNTHGSGSGSESSGS
NTDSSGESGFCAGKANGIYADPTNKSKFYNCNNGETFAQSCQAGLVFDSSCSCCNWA

Chia2/Chia3 Chimera C4
YVLSCYFTNWAQYRPGLGKYMPDNIDPCLCDHLIYAFAGMSNNEITTYEWNDETLYKSFNGLKNQNG
NLKTLLAIGGWNFGTAKFSTMVSTPENRQTFINSVIKFLRRYQFDGLDIDWEYPGSRGSPSQDKGLF
TVLVQEMLAAFEQEAKQVNKPRLMITAAVAAGLSNIQAGYQIAELGKYLDYFHVMTYDFHGSWDTQT
GENSPLYQGPSDTGDNIYFNVDYAMNYWKSNGAPAEKLVVGFPAYGNTFRLQNPSNHGLGAPTSGPG
PAGPYTQEAGTLAYYEICTLLNSGGTQVWDAPQDVPYAYKGSTWVGYDNIKSFNIKADWLKKNNYGG
AMVWAIDLDDFTGTFCKQGKYPLITTLKNALGQQSNSCVPSTQPSPTTTAVPCNTHGSGSGSESSGS
NTDSSGESGFCAGKANGIYADPTNKSKFYNCNNGETFAQSCQAGLVFDSSCSCCNWA

Chia2/Chia3 Chimera C5
YVLSCYFTNWAQYRPGLGKYMPDNIDPCLCDHLIYAFAGMSNNEITTYEWNDETLYKSFNGLKNQNK
NLKTLLSIGGWNFGTDKFSTMVSTPENRQTFINSVIKFLRRYQFDGLDIDWEYPGSRGSPSQDKGLF
TVLVQEMLAAFEQEAKQVNKPRLMITAAVAAGLSNIQAGYQIAELGKYLDYFHVMTYDFHGSWDTQT
GENSPLYQGPSDTGDNIYFNVDYAMNYWKSNGAPAEKLVVGFPAYGNTFRLQNPSNHGLGAPTSGPG
PAGPYTQEAGTLAYYEICTLLNSGGTQVWDAPQDVPYAYKGSTWVGYDNIKSFNIKADWLKKNNYGG
AMVWAIDLDDFTGTFCKQGKYPLITTLKNALGQQSNSCVPSTQPSPTTTAVPCNTHGSGSGSESSGS
NTDSSGESGFCAGKANGIYADPTNKSKFYNCNNGETFAQSCQAGLVFDSSCSCCNWA

Chia2/Chia3 Chimera C6
YVLSCYFTNWAQYRPGVGSFMPDNIDPCLCTHLLYAFAGMSNNEITTIEWNDVTLYKSFNGLKNQNK
NLKTLLSIGGWNFGTDKFSTMVSTPENRQTFINSVIKFLRRYQFDGLDIDWEYPGSRGSPSQDKGLF
TVLVQEMLAAFEQEAKQVNKPRLMITAAVAAGLSNIQAGYQIAELGKYLDYFHVMTYDFHGSWDTQT
GENSPLYQGPSDTGDNIYFNVDYAMNYWKSNGAPAEKLVVGFPAYGNTFRLQNPSNHGLGAPTSGPG
PAGPYTQEAGTLAYYEICTLLNSGGTQVWDAPQDVPYAYKGSTWVGYDNIKSFNIKADWLKKNNYGG
AMVWAIDLDDFTGTFCKQGKYPLITTLKNALGQQSNSCVPPAQPNPPITAAPSTGSGSGSGSGSGSS
GSNTGSSGGSGFCAGKANGIYADPTNKSKFYNCNNGETFVQSCQAGLVFDSSCSCCNWA

Chia2/Chia3 Chimera C7
YVLSCYFTNWAQYRPGVGSFMPDNIDPCLCTHLLYAFAGMSNNEITTIEWNDVTLYKSFNGLKNQNK
NLKTLLSIGGWNFGTDKFSTMVSTPENRQTFINSVIKFLRRYQFDGLDIDWEYPGSRGSPSQDKGLF
TVLVQEMLAAFEQEAKQVNKPRLMITAAVAAGLSNIQAGYQIAELGKYLDYFHVMTYDFHGSWDTQT
GENSPLYQGPSDTGDNIYFNVDYAMNYWKSNGAPAEKLVVGFPAYGNTFRLQNPSNHGLGAPTSGPG
PAGPYTQEAGTLAYYEICTLLNSGGTQVWDAPQDVPYAYKGSTWVGYDNIKSFNIKIDWLKKNNYGG
AMVWSLDMDDFTGTFCKQGKYPLITTLKNALGQQSSSCVPPAQPNPPITAAPSTGSGSGSGSGSGSS
GSNTGSSGGSGFCAGKANGIYADPTNKSKFYNCNNGETFVQSCQAGLVFDSSCSCCNWA

Chia2/Chia3 Chimera C8
YVLSCYFTNWAQYRPGVGSFMPDNIDPCLCTHLLYAFAGMSNNEITTIEWNDVTLYKSFNGLKNQNK

NLKTLLSIGGWNFGTDKFSTMVSTPENRQTFINSVIKFLRRYQFDGLDIDWEYPGSRGSPSQDKGLF
TVLVQEMLAAFEQEAKQVNKPRLMITAAVAAGLSNIQAGYQIAELGKYLDYFHVMTYDFHGSWDTQT
GENSPLYQGPSDTGDNIYFNVDYAMNYWKSNGAPAEKLVVGFPAYGNTFRLQNPSNHGLGAPTSGPG
PAGPYTQEAGTLAYYEICTFLDSGATQAWDAPQDVPYAYKSSEWVGYGNIKSFNIKIDWLKKNNYGG
AMVWSLDMDDFTGTFCKQGKYPLITTLKNALGQQSSSCVPPAQPNPPITAAPSTGSGSGSGSGSGSS
GSNTGSSGGSGFCAGKANGIYADPTNKSKFYNCNNGETFVQSCQAGLVFDSSCSCCNWA

Chia2/Chia3 Chimera C9
YVLSCYFTNWAQYRPGVGSFMPDNIDPCLCTHLLYAFAGMSNNEITTIEWNDVTLYKSFNGLKNQNK
NLKTLLSIGGWNFGTDKFSTMVSTPENRQTFINSVIKFLRRYQFDGLDIDWEYPGSRGSPSQDKGLF
TVLVQEMLAAFEQEAKQVNKPRLMITAAVAAGLSNIQAGYQIAELGKYLDYFHVMTYDFHGSWDTQT
GENSPLYQGPSDTGDNIYFNVDYAMNYWKSNGAPAEKLLVGFPTYGHSYILKNPSDTAVGAPTSGPG
PAGPYTRQSGFLAYYEICTFLDSGATQAWDAPQDVPYAYKSSEWVGYGNIKSFNIKIDWLKKNNYGG
AMVWSLDMDDFTGTFCKQGKYPLITTLKNALGQQSSSCVPPAQPNPPITAAPSTGSGSGSGSGSGSS
GSNTGSSGGSGFCAGKANGIYADPTNKSKFYNCNNGETFVQSCQAGLVFDSSCSCCNWA

Chia2/Chia3 Chimera C10
YVLSCYFTNWAQYRPGVGSFMPDNIDPCLCTHLLYAFAGMSNNEITTIEWNDVTLYKSFNGLKNQNK
NLKTLLSIGGWNFGTDKFSTMVSTPENRQTFINSVIKFLRRYQFDGLDIDWEYPGSRGSPSQDKGLF
TVLVQEMLAAFEQEAKQVNKPRLMITAAVAAGLSNIQAGYQIAELGKYLDYFHVMTYDFHGSWDGQT
GENSPLYKGPADTGDLIYFNVDYAMNYWKSNGAPAEKLLVGFPTYGHSYILKNPSDTAVGAPTSGPG
PAGPYTRQSGFLAYYEICTFLDSGATQAWDAPQDVPYAYKSSEWVGYGNIKSFNIKIDWLKKNNYGG
AMVWSLDMDDFTGTFCKQGKYPLITTLKNALGQQSSSCVPPAQPNPPITAAPSTGSGSGSGSGSGSS
GSNTGSSGGSGFCAGKANGIYADPTNKSKFYNCNNGETFVQSCQAGLVFDSSCSCCNWA

Chia2/Chia3 Chimera C11
YVLSCYFTNWAQYRPGLGKYMPDNIDPCLCDHLIYAFAGMSNNEITTYEWNDETLYKSFNGLKNQNK
NLKTLLSIGGWNFGTDKFSTMVSTPENRQTFINSVIKFLRQYQFDGLDIDWEYPGSKGSPSQDKGLF
TVLVQEMLAAFEQEAKQVNKPRLMITAAVAAGLSNIQAGYQIAELGKYLDYFHVMTYDFHGSWDGQT
GENSPLYKGPADTGDLIYFNVDYAMNYWKSNGAPAEKLVVGFPAYGNTFRLQNPSNHGLGAPTSGPG
PAGPYTQEAGTLAYYEICTLLDSGATQAWDAPQDVPYAYKSSEWVGYGNIKSFNIKIDWLKKNNYGG
AMVWSLDMDDFTGTFCKQGKYPLITTLKNALGQQSSSCVPPAQPNPPITAAPSTGSGSGSGSGSGSS
GSNTGSSGGSGFCAGKANGIYADPTNKSKFYNCNNGETFVQSCQAGLVFDSSCSCCNWA

Chia2/Chia3 Chimera C12
YVLSCYFTNWAQYRPGLGKYMPDNIDPCLCDHLIYAFAGMSNNEITTYEWNDETLYKSFNGLKNQNK
NLKTLLSIGGWNFGTDKFSTMVSTPENRQTFINSVIKFLRQYQFDGLDIDWEYPGSKGSPSQDKGLF
TVLVQEMLAAFEQEAKQVNKPRLMITAAVAAGLSNIQAGYQIAELGKYLDYFHVMTYDFHGSWDGQT
GENSPLYKGPADTGDLIYFNVDYAMNYWKSNGAPAEKLVVGFPAYGNTFRLQNPSNHGLGAPTSGPG
PAGPYTRQSGFLAYYEICTFLDSGATQAWDAPQDVPYAYKSSEWVGYGNIKSFNIKIDWLKKNNYGG
AMVWSLDMDDFTGTFCKQGKYPLITTLKNALGQQSSSCVPPAQPNPPITAAPSTGSGSGSGSGSGSS
GSNTGSSGGSGFCAGKANGIYADPTNKSKFYNCNNGETFVQSCQAGLVFDSSCSCCNWA

Chia2/Chia3 Chimera C13
YVLSCYFTNWAQYRPGLGKYMPDNIDPCLCDHLIYAFAGMSNNEITTYEWNDETLYKSFNGLKNQNK
NLKTLLSIGGWNFGTDKFSTMVSTPENRQTFINSVIKFLRQYQFDGLDIDWEYPGSKGSPSQDKGLF
TVLVQEMLAAFEQEAKQVNKPRLMITAAVAAGLSNIQAGYQIAELGKYLDYFHVMTYDFHGSWDGQT
GENSPLYKGPADTGDLIYFNVDYAMNYWKSNGAPAEKLVVGFPAYGNTFRLQNPSDTAVGAPTSGPG
PAGPYTRQSGFLAYYEICTFLDSGATQAWDAPQDVPYAYKSSEWVGYGNIKSFNIKIDWLKKNNYGG
AMVWSLDMDDFTGTFCKQGKYPLITTLKNALGQQSSSCVPPAQPNPPITAAPSTGSGSGSGSGSGSS
GSNTGSSGGSGFCAGKANGIYADPTNKSKFYNCNNGETFVQSCQAGLVFDSSCSCCNWA

Chia2/Chia3 Chimera C14
YVLSCYFTNWAQYRPGLGKYMPDNIDPCLCDHLIYAFAGMSNNEITTYEWNDETLYKSFNGLKNQNK
NLKTLLSIGGWNFGTDKFSTMVSTPENRQTFINSVIKFLRQYQFDGLDIDWEYPGSKGSPSQDKGLF
TVLVQEMLAAFEQEAKQVNKPRLMITAAVAAGLSNIQAGYQIAELGKYLDYFHVMTYDFHGSWDGQT
GENSPLYKGPADTGDLIYFNVDYAMNYWKSNGAPAEKLVVGFPAYGHSYILKNPSDTAVGAPTSGPG
PAGPYTRQSGFLAYYEICTFLDSGATQAWDAPQDVPYAYKSSEWVGYGNIKSFNIKIDWLKKNNYGG
AMVWSLDMDDFTGTFCKQGKYPLITTLKNALGQQSSSCVPPAQPNPPITAAPSTGSGSGSGSGSGSS
GSNTGSSGGSGFCAGKANGIYADPTNKSKFYNCNNGETFVQSCQAGLVFDSSCSCCNWA

Figure S2. Deduced amino acid sequences of the recombinant Chia proteins expressed in *E. coli*. The amino acid sequences are color-coded, consistent with Figs. 1-4. In chimeras or mutants, pink, chicken Chia2; yellow, chicken Chia3.

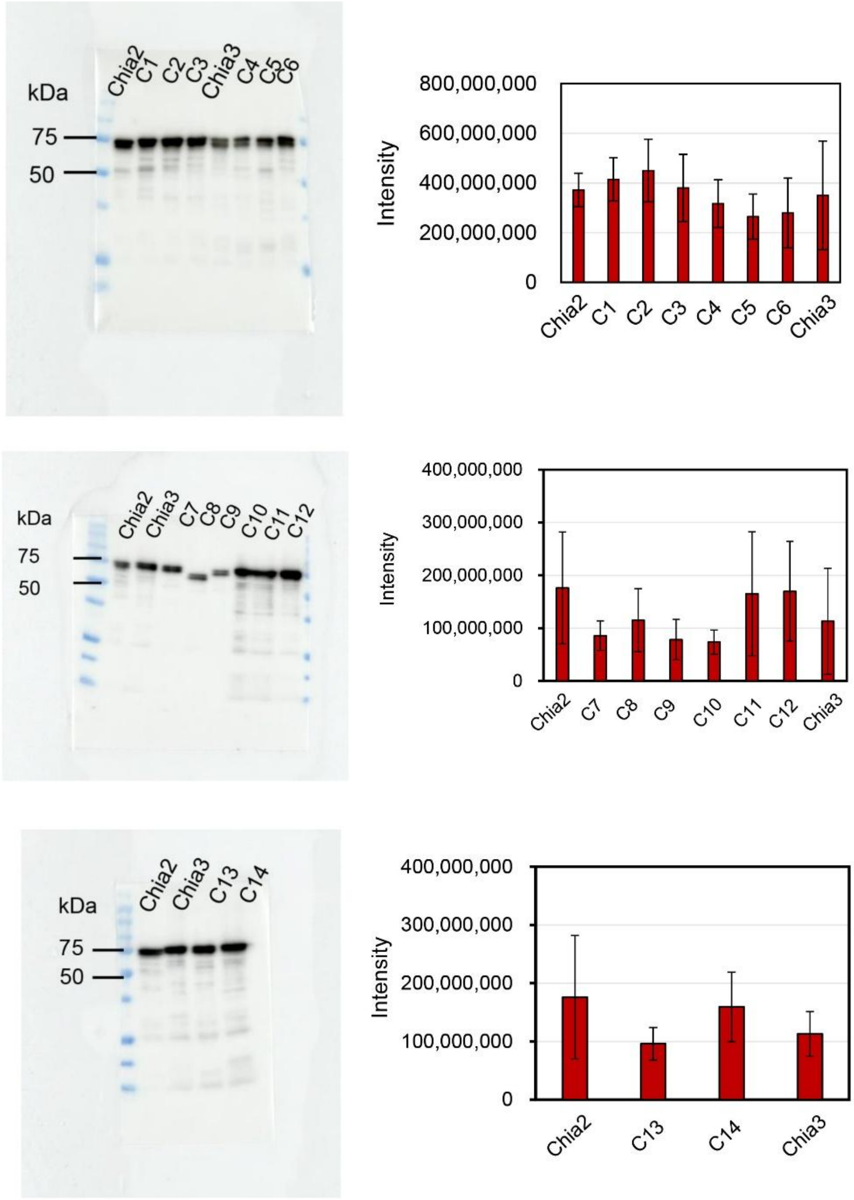

Figure S3. Western blot analysis of the recombinant proteins, related to Figure 3. Each gel image and the corresponding quantification graph relate to the experiments shown in Figure 3B and 3D. Recombinant protein fractions were analyzed by standard SDS–polyacrylamide gel electrophoresis (SDS-PAGE) followed by Western blotting using an anti-V5-HRP monoclonal antibody. Full-length images of the Western blots are shown in the left panels. Signal intensities were quantified as described in the Materials and Methods (right panels).

YVLSCYFTNWAQYRPGLGKYMPDNIDPCLCDHLIYAFAGMSNNEITTYEWNDETLYKSFNGLKNQNG
NLKTLLAIGGWNFGTAKFSTMVSTPENRQTFINSVIKFLRQYQFDGLDIDWEYPGSKGSPSQDKGLF
TVLVQEMLAAFEQEAKQVNKPRLMITAAVAAGLSNIQAGYQIAELGKYLDYFHVMTYDFHGSWDGQT
GENSPLYKGPADTGDLIYFNVDYAMNYWKSNGAPAEKLVVGFPAYGNTFRLQNPSNHGLGAPTSGPG
PAGPYTQEAGTLAYYEICTLLDSGATQAWDAPQDVPYAYKSSEWVGYGNIKSFNIKIDWLKKNNYGG
AMVWSLDMDDFTGTFCKQGKYPLITTLKNALGQQSSSCVPPAQPNPPITAAPSTGSGSGSGSGSGSS
GSNTGSSGGSGFCAGKANGIYADPTNKSKFYNCNNGETFVQSCQAGLVFDSSCSCCNWA

Figure S4. Amino acid sequence of the central region of exon 8 plays a crucial role in determining the pH-related enzymatic activity. The amino acid sequences are color-coded. In chimeras or mutants, pink, chicken Chia2; yellow, chicken Chia3 exon 8; green background, critical region of exon 8.

Supplementary Text S1.

Amino Acids at Positions 104 and 269 Contribute to the Determination of the pH Optimum

The critical region within exon 8 was further narrowed to its central portion, which distinguishes chimeras C12 and C13 from C14 (Supplementary Fig. S5A, lower; Supplementary Fig. S3). Comparative sequence analysis of exon 4 and the central region of exon 8 revealed eight amino acid differences between the Chia2 and Chia3 sequences—three in exon 4 and five in exon 8 (Supplementary Fig. S5A). These residues were hypothesized to be responsible for the observed differences in pH optima.

To test this, we engineered a series of chimeras (C15–C22) introducing specific amino acid substitutions in exon 8. Among these, the H269N mutation (C22) was identified as a major contributor to the shift in pH optimum (Supplementary Fig. S5B–D; Supplementary Fig. S2). To evaluate potential synergistic effects, we also introduced combined mutations involving residues in exon 4—specifically, G88K/H269N, A95S/H269N, and A104D/H269N (2MT-1 to 2MT-3; Fig. 4B; Supplementary Fig. S2). The A104D/H269N combination (2MT-3) was particularly effective, shifting the pH optimum toward 5.0 (Supplementary Fig. S5E), indicating that A104D contributes to pH modulation, especially in synergy with the H269N substitution.

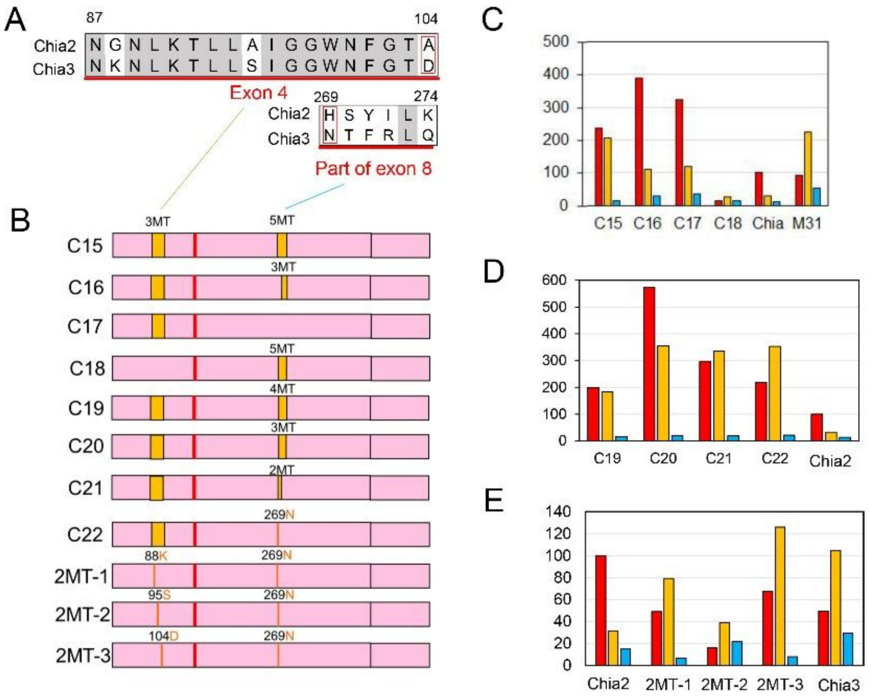

Supplementary Figure S5. Identification of key amino acids affecting pH optima of Chia2 and Chia3. (A) Comparative analysis of amino acid sequences of exon 4 and the middle region of exon 8 between Chia2 and Chia3, highlighting eight specific amino acid differences. This panel suggests potential roles for three amino acids in exon 4 and five in the middle region of exon 8 in contributing to pH-related activity (white backgrounds). (B) Schematic representation of chimeras C15-C21, designed to investigate the role of exon 8 sequence, with a focus on position 269. (C and D) Enzymatic activity profiles of chimeras and mutants. (E) Analysis of mutants 2MT-1, 2MT-2, and 2MT-3, targeting three amino acids in exon 4, focusing on the A104D mutation. All enzymatic activities were normalized to Chia2 activity at pH 2.0, which was defined as 100%.

Chia2_A104D
YVLSCYFTNWAQYRPGLGKYMPDNIDPCLCDHLIYAFAGMSNNEITTYEWNDETLYKSFNGLKNQNG
NLKTLLAIGGWNFGTDKFSTMVSTPENRQTFINSVIKFLRQYQFDGLDIDWEYPGSKGSPSQDKGLF
TVLVQEMLAAFEQEAKQVNKPRLMITAAVAAGLSNIQAGYQIAELGKYLDYFHVMTYDFHGSWDGQT
GENSPLYKGPADTGDLIYFNVDYAMNYWKSNGAPAEKLLVGFPTYGHSYILKNPSDTAVGAPTSGPG
PAGPYTRQSGFLAYYEICTFLDSGATQAWDAPQDVPYAYKSSEWVGYGNIKSFNIKIDWLKKNNYGG
AMVWSLDMDDFTGTFCKQGKYPLITTLKNALGQQSSSCVPPAQPNPPITAAPSTGSGSGSGSGSGSS
GSNTGSSGGSGFCAGKANGIYADPTNKSKFYNCNNGETFVQSCQAGLVFDSSCSCCNWA

Chia2_ H267N
YVLSCYFTNWAQYRPGLGKYMPDNIDPCLCDHLIYAFAGMSNNEITTYEWNDETLYKSFNGLKNQNG
NLKTLLAIGGWNFGTAKFSTMVSTPENRQTFINSVIKFLRQYQFDGLDIDWEYPGSKGSPSQDKGLF
TVLVQEMLAAFEQEAKQVNKPRLMITAAVAAGLSNIQAGYQIAELGKYLDYFHVMTYDFHGSWDGQT
GENSPLYKGPADTGDLIYFNVDYAMNYWKSNGAPAEKLLVGFPTYGNSYILKNPSDTAVGAPTSGPG
PAGPYTRQSGFLAYYEICTFLDSGATQAWDAPQDVPYAYKSSEWVGYGNIKSFNIKIDWLKKNNYGG
AMVWSLDMDDFTGTFCKQGKYPLITTLKNALGQQSSSCVPPAQPNPPITAAPSTGSGSGSGSGSGSS
GSNTGSSGGSGFCAGKANGIYADPTNKSKFYNCNNGETFVQSCQAGLVFDSSCSCCNWA

Chia2_A104D/H267N
YVLSCYFTNWAQYRPGLGKYMPDNIDPCLCDHLIYAFAGMSNNEITTYEWNDETLYKSFNGLKNQNG
NLKTLLAIGGWNFGTDKFSTMVSTPENRQTFINSVIKFLRQYQFDGLDIDWEYPGSKGSPSQDKGLF
TVLVQEMLAAFEQEAKQVNKPRLMITAAVAAGLSNIQAGYQIAELGKYLDYFHVMTYDFHGSWDGQT
GENSPLYKGPADTGDLIYFNVDYAMNYWKSNGAPAEKLLVGFPTYGNSYILKNPSDTAVGAPTSGPG
PAGPYTRQSGFLAYYEICTFLDSGATQAWDAPQDVPYAYKSSEWVGYGNIKSFNIKIDWLKKNNYGG
AMVWSLDMDDFTGTFCKQGKYPLITTLKNALGQQSSSCVPPAQPNPPITAAPSTGSGSGSGSGSGSS
GSNTGSSGGSGFCAGKANGIYADPTNKSKFYNCNNGETFVQSCQAGLVFDSSCSCCNWA

Chia3_D104A
YVLSCYFTNWAQYRPGVGSFMPDNIDPCLCTHLLYAFAGMSNNEITTIEWNDVTLYKSFNGLKNQNK
NLKTLLSIGGWNFGTAKFSTMVSTPENRQTFINSVIKFLRRYQFDGLDIDWEYPGSRGSPSQDKGLF
TVLVQEMLAAFEQEAKQVNKPRLMITAAVAAGLSNIQAGYQIAELGKYLDYFHVMTYDFHGSWDTQT
GENSPLYQGPSDTGDNIYFNVDYAMNYWKSNGAPAEKLVVGFPAYGNTFRLQNPSNHGLGAPTSGPG
PAGPYTQEAGTLAYYEICTLLNSGGTQVWDAPQDVPYAYKGSTWVGYDNIKSFNIKADWLKKNNYGG
AMVWAIDLDDFTGTFCKQGKYPLITTLKNALGQQSNSCVPSTQPSPTTTAVPCNTHGSGSGSESSGS
NTDSSGESGFCAGKANGIYADPTNKSKFYNCNNGETFAQSCQAGLVFDSSCSCCNWA

Chia3_D104A
YVLSCYFTNWAQYRPGVGSFMPDNIDPCLCTHLLYAFAGMSNNEITTIEWNDVTLYKSFNGLKNQNK
NLKTLLSIGGWNFGTAKFSTMVSTPENRQTFINSVIKFLRRYQFDGLDIDWEYPGSRGSPSQDKGLF
TVLVQEMLAAFEQEAKQVNKPRLMITAAVAAGLSNIQAGYQIAELGKYLDYFHVMTYDFHGSWDTQT
GENSPLYQGPSDTGDNIYFNVDYAMNYWKSNGAPAEKLVVGFPAYGNTFRLQNPSNHGLGAPTSGPG
PAGPYTQEAGTLAYYEICTLLNSGGTQVWDAPQDVPYAYKGSTWVGYDNIKSFNIKADWLKKNNYGG
AMVWAIDLDDFTGTFCKQGKYPLITTLKNALGQQSNSCVPSTQPSPTTTAVPCNTHGSGSGSESSGS
NTDSSGESGFCAGKANGIYADPTNKSKFYNCNNGETFAQSCQAGLVFDSSCSCCNWA

Chia3_D104A/N267H
YVLSCYFTNWAQYRPGVGSFMPDNIDPCLCTHLLYAFAGMSNNEITTIEWNDVTLYKSFNGLKNQNK
NLKTLLSIGGWNFGTAKFSTMVSTPENRQTFINSVIKFLRRYQFDGLDIDWEYPGSRGSPSQDKGLF
TVLVQEMLAAFEQEAKQVNKPRLMITAAVAAGLSNIQAGYQIAELGKYLDYFHVMTYDFHGSWDTQT
GENSPLYQGPSDTGDNIYFNVDYAMNYWKSNGAPAEKLVVGFPAYGHTFRLQNPSNHGLGAPTSGPG
PAGPYTQEAGTLAYYEICTLLNSGGTQVWDAPQDVPYAYKGSTWVGYDNIKSFNIKADWLKKNNYGG
AMVWAIDLDDFTGTFCKQGKYPLITTLKNALGQQSNSCVPSTQPSPTTTAVPCNTHGSGSGSESSGS
NTDSSGESGFCAGKANGIYADPTNKSKFYNCNNGETFAQSCQAGLVFDSSCSCCNWA

Figure S6. Deduced amino acid sequences of the mutant Chia paralogs expressed in *E. coli*. The amino acid sequences are color-coded, consistent with Fig. 4. In mutants, pink, chicken Chia2; yellow, chicken Chia3.

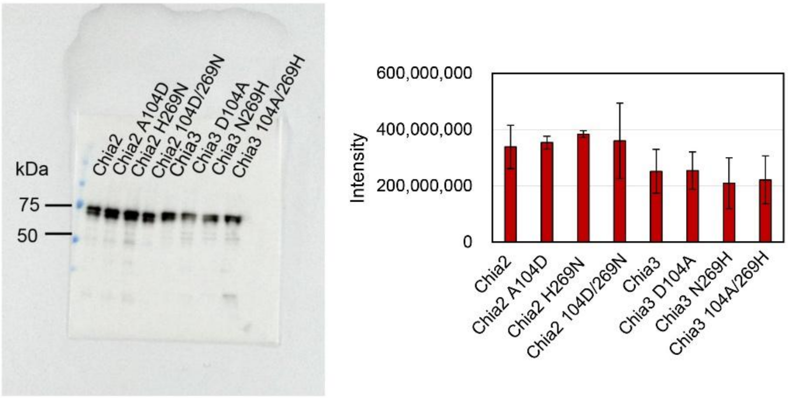

Figure S7. Western blot analysis of recombinant proteins related to Figure 4. Each gel image and the corresponding quantification graph relate to the experiments shown in Figure 4A and 4B. Recombinant protein fractions were analyzed by SDS–PAGE followed by Western blotting using an anti-V5-HRP monoclonal antibody. Full-length images of the Western blots are shown in the left panel. Signal intensities were quantified as described in the Materials and Methods (right panel).

Ruff Chia1

YVLSCYFTNWAQYRPGLGKFMPDDIDPCLCDHLIYAFAGMNNNEITTYEWNDETLYKSFNGLKNQNR
NLKTLLAIGGWNFGTAKFTTMVSTPANRQTFIKSVIKFLRQHQFDGLDLDWEYPGSRGSPAQDKALF
TVLVKELVAAFEQEAKQSNKPRLMVTAAVAAGISTVQAGYEIPELGKYLDYIHVMTYDFHSSWDRVT
GENSPLYDGSHSQLSVEYAMNYWKNNGAPSKKLLVGFPTYGRNFNLQNPSNTAVGAPATGPGPAGPY
TKEAGFLAYYEICTFLDNGATQAWDAAEDVPYAYKGSEWVGYDNIKSFNIKVDWLKKGNFGGAMVWA
IDLDDFTGTFCKQGKYPLITTLKNGLGLQSSGCRAN

Ruff Chia2

YVLSCYFTNWAQYRPGLGKFMPDDIDPCLCDHLIYAFAGMNNNEITTYEWNDETLYKSFNGLKNQNG
KLKTLLAIGGWNFGTAKFTTMVSTPANRQTFIKSVIKFLRQYQFDGLDLDWEYPGSRGSPAQDKALF
TVLVKELVAAFEQEAKQSNKPRLMVTAAVAGGRSTIEAGYEIPELGKYLDYIHVMTYDFHGSWDGVT
GENSPLYEGPVDTGDLIYFNVDFAMNYWKNNGAPAEKLLVGFATYGHNYILQNPSNTAVGAPATGPG
PAGPYTRQAGFLAYYEICTFLANGATQAWDAPQDVPYAYKGSEWVGYDNIKSYNIKVDWLKKNKFGG
AMVWAIDMDDFTGTFCKEGKYPLITTLKNGLGLQNSDCVPPAHPNPPITEAPSQGGGSGSGGSGGGS
GGSGFCAGKANGIYADPTNKSNFYNCINGETFMQTCQAGLVFDASCSCCNWP

Ruff Chia3

YVLSCYFTNWAQYRPGLGKFMPDDIDPCLCTHLLYAFAGMNNNEITTYEWNDETLYKSFNGLKNQNS
NLKTLLSIGGWNFGSDKFTTMVSTPRNRQTFIKSVIKFLRQYQFDGLDLDWEYPGSRGSPAQDKALF
TVLVKELVAAFEQEAKQSNKPRLMVTAAVAAGVSKIEAGYEIPELGKYLDYIHVMTYDFHGSWDTNT
GENSPLYKGPADTGDFIYFNVDYAMNYWKSHGAPAEKLVVGFATYGNTFTLRNPSNNGLGAPASGPG
PAGHYTQEAGTLAYYEICSLLNSGATQAWDAPQDVPYAYKGSEWVGYDNIKSFNIKVDWLKKNKFGG
AMVWAIDLDDFTGTFCKQGKYPLITTLKNGLGLQNSDCVSPNQPTTTTASTTTIDSTTTTFYLCTSG
LTHGSESGGSGGSSFCAGKANGIYADPTNKRNFYNCINGETFMQSCDNGLVFDTSCSCCNWP

Downy woodpecker Chia1

YILSCYFTNWAQYRPGLGKYTPENVDPCLCNHLIYAFAGMANNEITTYEWNDETLYKSFNGLKNQNK
NLKTLLAIGGWNFGTEKFSTMVSTPQNRQTFIKSVIRFLRQYQFDGLDLDWEYPGSRGSPAQDKALF
TVLVKEMVAAFEQEAKEVNKPRLMVTAAVAAGLSTIQAGYEIAELGKYLDYIHVMTYDFSSAYDGRT
GENSPLHNTANRQFSVEYAMSYWRDSGAPAQKLLVGFPTYGHSFTLQNPSNTAVGAPTTGPGPAGPY
TGEDGLLAYYEICTILDSGATQAWDASQDVPYAYKGSQWVGYDNTKSFSLKVDWLKKNNFGGAMVWT
IDLDDFTGTFCKQGKYPLISTLKNGLGLRGCSSY

Downy woodpecker Chia2

YILSCYFTNWAQYRPGLGKYTPENVDPCLCNHLIYAFAGMANNEITTYEWNDETLYKSFNGLKNQNK
NLKTLLAIGGWNFGTEKFSTMVSTPQNRQTFIKSVIRFLRQYQFDGLDLDWEYPGSRGSPAQDKALF
TVLVKEMVAAFEQEAKEVNKPRLMVTAAVAAGLSTIQAGYEIAELGKYLDYFHVMTYDFHGAWDSTT
GENSPLYKGPADTGDLVYFNVDYAMNYWKDNGAPAEKLLVGFPTYGHNFVLQNPSNTAVGAPASGPG
PAGPYTRQSGFLAYYEICTFLSEGATQAWDAPQDVPYAYKGNEWVGYDNIKSFNIKVDWLKKNKFGG
AMVWALDMDDFTGTFCKEGKYPLINTLKKGLGLDSGDCVPPAEPIPPITEAPPSQGGGSGGSGGSGG
SGGSGFCAGKANGLYADPQNKKNFYNCVNGVTYLESCQAGLVFDASCSCCNWA

Barn swallow Chia1

YVLTCYFTNWAQYRPGLGKFTPENVDPCLCNHLIYAFAGMNNNEITTYEWNDETLYKSFNGLKNQNK
DLKTLLAIGGWNFGTQKFTTMVSSPENRQTFIKSVIKFLRQYQFDGLDLDWEYPGSRGSPAQDKALF
TVLVKELLAAFEQEAKQSNRPRLMVTAAVAAGLSTIQAGYEIAELGKYLDYIHVMTYDFHGSWERNT
GENSPLYAGPADSGDYKYFNVEYAMNYWKSNGAPAEKLLVGFPTYGKSFTLQNPSDTSVGAPASGPG
PAGPYTREAGTLAYYEICTLLSSGATQAWDEPQDVPYAYQGSEWVGYDNIKSFGLKVDWLKKNNFGG
AMVWALDMDDFTGDFCKEGKYPLISTLKKGLGLQSGDCVPPAEPLPPITEAPTTTSSSSSGGSGGSG
FCAGKPNGIYADPNNKRNFYSCLNGQTFLQSCEQGLVFDPVCTCCNWPQ

Barn swallow Chia2

YVLTCYFTNWAQYRPGLGKFTPENVDPCLCNHLIYAFAGMNNNEITTYEWNDETLYKSFNGLKNQNK
DLKTLLAIGGWNFGTQKFTTMVSSPENRQTFIKSVIKFLRQYQFDGLDLDWEYPGSRGSPAQDKALF
TVLVKELLAAFEQEAKQSNRPRLMVTAAVAAGLSTIQAGYEIAELGKYLDYIHVMTYDFHGSWERNT
GENSPLYAGPADSGDYKYFNVEYAMNYWKSNGAPAEKLLVGFPTYGKSFTLQNPSDTSVGAPASGPG
PAGPYTREAGTLAYYEICTLLSSGATQAWDEPQDVPYAYQGSEWVGYDNIKSFGLKVDWLKKNNFGG
AMVWALDMDDFTGDFCKEGKYPLISTLKKGLGLQSGDCVPPAEPLPPITEAPTTTSSSSSGGSGGSG
FCAGKPNGIYADPNNKRNFYSCLNGQTFLQSCEQGLVFDPVCTCCNWPQ

Figure S8. Deduced amino acid sequences of the recombinant Chia proteins expressed in *E. coli.* The amino acid sequences are color-coded, consistent with Fig. 7.

(a)

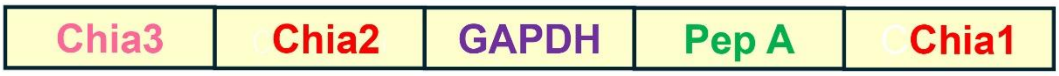

(b)

CTTTCAGACTGCAAAACCCATCTAATCATGGTCTTGGTGCACCG
ACTTCAGGACCAGGACCTGCTGG**ACCTTATACACAGGAGGCTGG**
GACGCGACATAGCTACATCCTCAAGAATCCATCCGACACTGCTG
TTGGGGCACCAACATCGGGCCCTGGGCCAGCCGGGCCTTACACA
AGACAGTCTGGTTTCTTAGCTCAACGGATTTGGCCGTATTGGCC
GCCTGGTCACCAGGGCTGCCGTCCTCTCTGGCAAAGTCCAAGTG
GTGGCCATCAA**TGATCCCTTCATCGATCTGAACTGTGGGTGCCC**
TCTATCTATTGCAAAAGCTCGGCCTGCAGCAACCACAAACGCTT
TGACCCCTCCAAGTCCTCAACCTACGTGAGCACCAAC**GAAACCG**
TCTACATCGCCTACGATGACTTACGACTTCTACAGCTCTGGGGA
TGGACAAACAGGGGAGAACAGCCCTTTGTACA**GTGGCAATAATG**
TCTACCT

Figure S9. Single standard DNA molecule used for qPCR. (a) Schematic representation of the standard DNA molecule. (b) Nucleotide sequence of the single standard DNA. The single standard DNA has 491 nucleotides, contains cDNA fragments of Chia1, Chia2 and Chia3 as well as reference genes [pepsinogen A (Pep A) and GAPDH] of chicken shown in (a). Primers for qPCR in each target DNA region are underlined.

Table S1. NCBI Gene Accession numbers of Chia sequences

Order		Scientific name	English name	Gene name	Gene Accession No.
		Mus musculus	House mouse	*Chia1*	NM_023186.3
				Chit1	XM_006529880.2
		Homo sapiens	Human	*CHIA*	NM_201653.4
				CHIT1	NM_001256125.2
		Alligator mississippiensis	American alligator	*Chia1*	XM_059717121.1
				Chia2	XM_059717133.1
				Chia3	XM_059717081.1
				Chia4	XM_006278760.3
				Chia5	XM_019491529.2
				Chia6	XM_019491489.2
				Chia8	XM_019491490.2
				Chit1	XR_009456573.1
Galloanserae	Galliformes	*Gallus gallus*	Red Junglefowl	*Chia2*	NM_204429.2
				Chia1	XM_015298968.4
				Chia3	XM_418051.7
		Numida meleagris	Helmeted Guineafowl	*Chia-like*	XM_021377506.1
		Meleagris gallopavo	Wild Turkey	*Chia*	XM_010726477.2
		Phasianus colchicus	Common Pheasant	*Chia3*	XM_031605946.1
		Tympanuchus pallidicinctus	Lesser Prairie Chicken	*Chia1*	XM_052698482.1
				Chia2	XM_052698422.1
	Anseriformes	*Cygnus olor*	Mute Swan	*Chia1*	XM_040535435.1
				Chia2	XM_040535653.1
		Cygnus atratus	Black Swan	*Chia1*	XM_035561419.2
				Chia2	XM_035561420.1
		Anas platyrhynchos	Mallard Duck	*Chia1*	XM_038168218.1
				Chia2	XM_038168217.1
				Chia3	XM_027445177.2
		Aythya fuligula	Tufted Duck	*Chia1*	XM_032203015.1
				Chia2	XM_032203325.1
				Chia3	XM_032203326.1
				Chia4	XM_032203331.1
				Chia5	XM_032203330.1
				Chia6	XM_032203328.1
				Chia7	XM_032203332.1
				Chia8	XM_032203324.1
		Gavia stellata	Red-throated Loon	*Chia*	XM_059831255.1
		Nestor notabilis	Kea	*Chia*	XM_010022810.1
		Tyto alba	Barn Owl	*Chia*	XM_033009328.2
		Merops nubicus	Northern Carmine Bee-eater	*Chia1*	XM_008942205.1
				Chia2	XM_008945035.1
		Athene cunicularia	Burrowing Owl	*Chia2*	XM_026864165.1
		Fulmarus glacialis		*Chia2*	XM_009573700.1
		Chaetura pelagica	Chimney Swift	*Chia1*	XM_010008318.1
				Chia2	XM_010007061.1
		Caprimulgus carolinensis	Chuck-will's-widow	*Chia2*	XM_010173477.1
		Mesitornis unicolor	Brown Mesite	*Chia1*	XM_010191411.1
				Chia2	XM_010182252.1
		Grus americana	Whooping Crane	*Chia1*	XM_054803871.1
				Chia2	XM_054803875.1
		Egretta garzetta	Little Egret	*Chia*	XM_009638220.1

Neoaves		*Calidris pugnax*	Ruff	*Chia1*	XM_014953617.1
				Chia2	XM_014953623.1
				Chia3	XM_014953628.1
		Gymnogyps californianus	California Condor	*Chia*	XM_050911257.1
		Falco naumanni	Lesser Kestrel	*Chia*	XM_040616538.1
		Falco cherrug	Saker Falcon	*Chia*	XM_055728398.1
		Falco rusticolus	Gyr Falcon	*Chia*	XM_037410455.1
		Falco peregrinus	Peregrine Falcon	*Chia*	XM_055820024.1
		Phalacrocorax carbo	Great Cormorant	*Chia*	XM_009500137.1
		Rissa tridactyla	Black-legged Kittiwake	*Chia1*	XM_054181341.1
				Chia2	XM_054181323.1
		Charadrius vociferus	Killdeer	*Chia1*	XM_009888302.1
				Chia2	XM_009888235.1
		Dryobates pubescens	Downy Woodpecker	*Chia1*	XM_054176286.1
				Chia2	XM_054176431.1
		Opisthocomus hoazin	Hoatzin	*Chia*	XM_009934824.1
	asseriformes	*Motacilla alba*	White Wagtail	*Chia1*	XM_038162392.1
				Chia2	XM_038162418.1
		Onychostruthus taczanowskii	White-rumped Snowfinch	*Chia1*	XM_041422703.1
				Chia2	XM_041427013.1
		Geospiza fortis	Medium Ground-finch	*Chia1*	XM_031063544.1
				Chia2	XM_014310316.2
		Serinus canaria	Island Canary	*Chia*	XM_050985149.1
		Manacus vitellinus	Golden-collared Manakin	*Chia1*	XM_008934306.2
				Chia2	XM_018079618.2
		Pseudopodoces humilis	Tibetan Ground-tit	*Chia1*	XM_014258150.1
				Chia2	XM_005529295.2
		Taeniopygia guttata	Zebra Finch	*Chia1*	XM_030292121.3
				Chia2	XM_041721339.1
		Parus major	Great Tit	*Chia1*	XM_015650926.2
				Chia2	XM_015650925.3
		Molothrus ater	Brown-headed Cowbird	*Chia1*	XM_036398462.1
				Chia2	XM_036398472.1
		Catharus ustulatus	Swainson's Thrush	*Chia1*	XM_033080155.2
				Chia2	XM_033080169.2
		Camarhynchus parvulus	Small Tree-finch	*Chia1*	XM_030965814.1
				Chia2	XM_030965824.1
		Pipra filicauda	Wire-tailed Manakin	*Chia1*	XM_039382315.1
				Chia2	XM_027746954.2
		Cyanistes caeruleus	Eurasian blue tit	*Chia1*	XM_023942222.1
				Chia2	XM_023942018.1
		Lonchura striata	White-rumped Munia	*Chia*	XM_031506969.1
		Ficedula albicollis		*Chia1*	XM_005061593.1
				Chia2	XM_016305051.1
		Passer montanus	Eurasian Tree Sparrow	*Chia1*	XM_039695942.1
				Chia2	XM_039695824.1
		Chiroxiphia lanceolata	Lance-tailed Manakin	*Chia*	XM_032710814.1
		Hirundo rustica	Barn Swallow	*Chia1*	XM_040085797.2
				Chia2	XM_040085799.2
		Sturnus vulgaris	Common Starling	*Chia1*	XM_014872879.1
				Chia2	XM_014872872.1
		Poecile atricapillus	Black-capped Chickadee	*Chia1*	XM_058856014.1

	Pa			*Chia2*	XM_058856013.1
		Empidonax traillii	Willow Flycatcher	*Chia*	XM_027903022.1
		Ammospiza nelsoni	Nelson's Sparrow	*Chia*	XM_059487906.1
				Chia2	XM_058819431.1
		Corapipo altera	White-ruffed Manakin	*Chia*	XM_027659607.1
		Haemorhous mexicanus	House Finch	*Chia*	XM_059867792.1
		Ammospiza caudacuta	Saltmarsh Sparrow	*Chia1*	XM_059487906.1
				Chia2	XM_058819431.1
		Oenanthe melanoleuca	Eastern Black-eared Wheatear	*Chia1*	XM_056511277.1
				Chia2	XM_056511284.1
		Agelaius phoeniceus	Red-winged Blackbird	*Chia1*	XM_054648737.1
				Chia2	XM_054648738.1
		Melozone crissalis	California Towhee	*Chia1*	XM_054291645.1
				Chia2	XM_054291646.1
		Vidua macroura	Pin-tailed Whydah	*Chia1*	XM_053998190.1
				Chia2	XM_053998172.1
		Vidua chalybeata	Village Indigobird	*Chia1*	XM_053963807.1
				Chia2	XM_053963808.1
		Manacus candei	White-collared Manakin	*Chia1*	XM_051808963.1
				Chia2	XM_051808961.1
		Myiozetetes cayanensis	Rusty-margined Flycatcher	*Chia1*	XM_050326673.1
				Chia2	XM_050326973.1
		Lepidothrix coronata	Blue-crowned Manakin	*Chia1*	XM_017832073.1
				Chia2	XM_017832174.1
		Corvus brachyrhynchos	American Crow	*Chia2*	XM_017727156.1
		Corvus hawaiiensis	Hawaiian Crow	*Chia1*	XM_048328288.1
				Chia2	XM_048328293.1
		Corvus kubaryi	Mariana Crow	*Chia1*	XM_042013745.1
				Chia2	XM_042013758.1
		Corvus moneduloides	New Caledonian Crow	*Chia1*	XM_032133461.1
				Chia2	XM_032133463.1
		Corvus cornix	Hooded Crow	*Chia1*	XM_039565494.1
				Chia2	XM_019288974.2

Supplementary Table S2. Construction of chimeric and mutant proteins by PCR

Chimera name	Part	Template	Forward	Reverse
C1	5'	Chia2	Protein A_Fw_2533	Chia2_Chia3_C1_Rv
	3'	Chia2-Chia3	Chia2_Chia3_C1_Fw	Sal_BDH_RV
C2	5'	Chia2	Protein A_Fw_2533	Chia2_Chia3_C2_Rv
	3'	Chia2-Chia3	Chia2_Chia3_C2_Fw	Sal_BDH_RV
C3	5'	Chia2	Protein A_Fw_2533	Chia2_Chia3_C3_Rv
	3'	Chia2-Chia3	Chia2_Chia3_C3_Fw	Sal_BDH_RV
C4	5'	Chia2	Protein A_Fw	Chia2Chia3_C3a_Rv
	3'	Chia2-Chia3	Chia2Chia3_C3a_Fw	Sal_BDH_RV
C5	5'	Chia2	Protein A_Fw_2533	Chia2Chia3_C3b_Rv
	3'	Chia2-Chia3	Chia2Chia3_C3b_Fw	Sal_BDH_RV
C6	5'	Chia2-Chia3	Protein A_Fw_2533	Mu_Dog_C4_Rv
	3'	Chia2	Mu_Dog_C4_Fw	Sal_BDH_RV
C7	5'	Chia2-Chia3	Protein A_Fw_2533	Chia2Chia3_C4a_Rv
	3'	Chia2	Chia2Chia3_C4a_Fw	Sal_BDH_RV
C8	5'	Chia2-Chia3	Protein A_Fw_2533	Chia2Chia3_C4b_Rv
	3'	Chia2	Chia2Chia3_C4b_Fw	Sal_BDH_RV
C9	5'	Chia2-Chia3	Protein A_Fw_2533	Chia2_Chia3_C5_Rv
	3'	Chia2	Chia2_Chia3_C5_Fw	Sal_BDH_RV
C10	5'	Chia2-Chia3	Protein A_Fw_2533	Chia2_Chia3_C6_Rv
	3'	Chia2	Chia2_Chia3_C6_Fw	Sal_BDH_RV
Chimera name	Part	Template	Forward	Reverse
C11-C14 5'-product	①	Chia2	Protein A_Fw_2533	Chia2_ex4_1_Rv
	②	Chia2-Chia3	Chia2_ex4_1_Fw	Chia2_ex4_2_Rv
	③	Chia2	Chia2_ex4_2_Fw	Chia2_ex8_Rv
C11-C14 5'-produ	5'	①-③	Protein A_Fw_2533	Chia2_ex8_Rv
C11 3'-product	④	Chia2-Chia3	Chia2_ex8_Fw	Chia2Chia3_C7_Rv
	⑤	Chia2	Chia2Chia3_C7_Fw	Sal_BDH_RV
C11 3'-product_2	3'	④+⑤	Chia2_ex8_Fw	Sal_BDH_RV
C12 3'-product	⑥	Chia2-Chia3	Chia2_ex8_Fw	Chia2Chia3_C8_Rv
	⑦	Chia2	Chia2Chia3_C8_Fw	Sal_BDH_RV
C12 3'-product_2	3'	⑥+⑦	Chia2_ex8_Fw	Sal_BDH_RV
C13 3'-product	⑧	Chia2-Chia3	Chia2_ex8_Fw	Chia2Chia3_C9_Rv
	⑨	Chia2	Chia2Chia3_C9_Fw	Sal_BDH_RV
C13 3'-product_2	3'	⑧+⑨	Chia2_ex8_Fw	Sal_BDH_RV
C14 3'-product	⑩	Chia2-Chia3	Chia2_ex8_Fw	Chia2Chia3_C10_Rv
	⑪	Chia2	Chia2Chia3_C10_Fw	Sal_BDH_RV
C14 3'-product_2	3'	⑩+⑪	Chia2_ex8_Fw	Sal_BDH_RV

Mutant name	Part	Template	Fw	Rv
Chia2 104D	5'	Chia2	pEZZ18_Fw2533	ChickChia2_A104D_Rv
	3'	Chia2	ChickChia2_A104D_Fw	Sal_BGH_RV
Chia2 266N	5'	Chia2	pEZZ18_Fw2533	ChickChia2_H269N_Rv
	3'	Chia2	ChickChia2_H269N_Fw	Sal_BGH_RV
hia2 104D/266N	5'	Chia2 104D	pEZZ18_Fw2533	ChickChia2_H269N_Rv
	3'	Chia2	ChickChia2_H269N_Fw	Sal_BGH_RV

Chia2-Chia3 104A	5'	Chia2-Chia3	pEZZ18_Fw2533	Chia3_D104A_Rv
	3'	Chia2-Chia3	Chia3_D104A_Fw	Sal_BGH_RV
Chia2-Chia3 266H	5'	Chia2-Chia3	pEZZ18_Fw2533	Chia3_N269H_Rv
	3'	Chia2-Chia3	Chia3_N269H_Fw	Sal_BGH_RV
Chia2-Chia3 104A/266H	5'	Chia2-Chia3 104A	pEZZ18_Fw2533	Chia3_N269H_Rv
	3'	Chia2-Chia3	Chia3_N269H_Fw	Sal_BGH_RV

Supplementary Table S3. Primers for construction of chimeric and mutant proteins

Primer name	Sequence
Chia2_Chia3_C1_Fw	TCTTGGCCAGCAAAGCAGCAGCTGTGTGCCCTCAACTCAGCCCA
Chia2_Chia3_C1_Rv	TGGGCTGAGTTGAGGGCACACAGCTGCTGCTTTGCTGGCCAAGA
Chia2_Chia3_C2_Fw	TGACCTCATCTATTTCAACGTTGATTATGCTATGAATTACTGGA
Chia2_Chia3_C2_Rv	TCCAGTAATTCATAGCATAATCAACGTTGAAATAGATGAGGTCA
Chia2_Chia3_C3_Fw	TCTCTTCACCGTCCTTGTTCAGGAAATGCTGGCCGCCTTTGAGC
Chia2_Chia3_C3_Rv	GCTCAAAGGCGGCCAGCATTTCCTGAACAAGGACGGTGAAGAGA
Chia2_Chia3_C4_Fw	TCTTGGCCAGCAAAGCAACAGCTGTGTGCCCCCAGCTCAGCCCA
Chia2_Chia3_C4_Rv	TGGGCTGAGCTGGGGGCACACAGCTGTTGCTTTGCTGGCCAAGA
Chia2_Chia3_C5_Fw	GGACAATATCTATTTTAATGTTGATTATGCTATGAACTATTGGA
Chia2_Chia3_C5_Fw	TCCAATAGTTCATAGCATAATCAACATTAAAATAGATATTGTCC
Chia2_Chia3_C6_Fw	TCTCTTCACCGTCCTTGTTCAGGAAATGCTGGCCGCCTTTGAGC
Chia2_Chia3_C6_Fw	GCTCAAAGGCGGCCAGCATTTCCTGAACAAGGACGGTGAAGAGA
Chia2_Chia3_C3a_Fw	GAATTTTGGAACAGCCAAGTTCTCCACAATGGTTTCCAC
Chia2_Chia3_C3a_Rv	GTGGAAACCATTGTGGAGAACTTGGCTGTTCCAAAATTC
Chia2_Chia3_C3b_Fw	CAATGGACTGAAAAATCAGAATAAAAACCTGAAGACTC
Chia2_Chia3_C3b_Rv	GAGTCTTCAGGTTTTTATTCTGATTTTTCAGTCCATTG
Chia2_Chia3_C4a_Fw	GAGCTTCAACATCAAGATTGACTGGCTGAAAAAAAAC
Chia2_Chia3_C4a_Rv	GTTTTTTTTCAGCCAGTCAATCTTGATGTTGAAGCTC
Chia2_Chia3_C4b_Fw	CGCTGGCTTACTATGAGATCTGCACATTCCTGGACTC
Chia2_Chia3_C4b_Rv	GAGTCCAGGAATGTGCAGATCTCATAGTAAGCCAGCG
Chia2_Chia3_C7_Fw	ACTATGAGATCTGCACACTACTGGACTCTGGAGCCACC
Chia2_Chia3_C7_Rv	GGTGGCTCCAGAGTCCAGTAGTGTGCAGATCTCATAGT
Chia2_Chia3_C8_Fw	CCTGCTGGACCTTATACAAGACAGTCTGGTTTCTTAGC
Chia2_Chia3_C8_Rv	GCTAAGAAACCAGACTGTCTTGTATAAGGTCCAGCAGG
Chia2_Chia3_C9_Fw	CAGACTGCAAAACCCATCTGACACTGCTGTTGGGGCAC
Chia2_Chia3_C9_Rv	GTGCCCCAACAGCAGTGTCAGATGGGTTTTGCAGTCTG
Chia2_Chia3_C10_Fw	GGATTCCCAGCCTACGGACATAGCTACATCCTCAAG
Chia2_Chia3_C10_Rv	CTTGAGGATGTAGCTATGTCCGTAGGCTGGGAATCC
Chia2_ex4_1_Fw	CAATGGACTGAAAAATCAGAATAAAAACCTGAAGACTC
Chia2_ex4_1_Rv	GAGTCTTCAGGTTTTTATTCTGATTTTTCAGTCCATTG
Chia2_ex4_2_Fw	GGAATTTTGGGACAGACAAGTTCTCCACAATGGTTTCCA
Chia2_ex4_2_Rv	TGGAAACCATTGTGGAGAACTTGTCTGTCCCAAAATTCC
Chia2_ex8_Fw	ACCTCATCTATTTCAACGTTGATTATGCTATGAATTACT
Chia2_ex8_Rv	AGTAATTCATAGCATAATCAACGTTGAAATAGATGAGGT
ChickChia2_H269N_Fw	TCCCAACCTATGGAaATAGCTACATCCTCA
ChickChia2_H269N_Rv	TGAGGATGTAGCTATTTCCATAGGTTGGGA
Chia3_D104A_Fw	GGAATTTTGGGACAGcCAAGTTCTCCACAATGG
Chia3_D104A_Rv	CCATTGTGGAGAACTTGGCTGTCCCAAAATTCC
Chia3_N269H_Fw	GATTCCCAGCCTACGGAcACACTTTCAGACTGC
Chia3_N269H_Rv	GCAGTCTGAAAGTGTGTCCGTAGGCTGGGAATC

Supplementary Data file

>Alligator_mississippiensis_Chit1
ATGGGGCAGGCCATCATTTGGGCTGGCTTAGCAGTCTTGCTGCTTCTGCAGTGTGGCTC
TGCTTGCAAGCTGGTGTGTTATTTCACCAACTGGTCCCAGTACAGACCTGCCCAGGGGC
GTTTCTTCCCCGAGAACATTGACACCAACCTGTGCACTCATCTCATTTATGCCTTTGCT
GGCATGAATGAGAACAGGATCACCACCATCGAGTGGAATGATGAGCAGTTCTACAAGAC
TTTCAATGGACTAAAAAGCAAGAACCCCAGCCTGAAGACCCTTTTGTCCATCGGGGGAT
GGAATTTTGGCTCCCAAAAGTTCTCCACCATGGTGTCCACTCCTGCCACCCGTTGGACA
TTCATCCTCTCTGTGCTTCAGTTCCTGCGCCAGTATGGCTTCGACGGCCTGGACATAGA
CTGGGAGTATCCGGCAGCGAGAGGAAGCCCTCCTGAGGATAAACAGCGTTTCACGGCTC
TAGTCCAGGAAATGGCTAAAGAATTCACGGAGGAAGGGAAGAGGACACAGAAAGAGAGG
CTGCTGTTGACTGCTGCTGTGGCTGCTGGGAGGGAAAAGATCAATGCAGGCTACGAAGT
CAGCGACATCCTAAAAGAACTGGATTTTATCAACCTCATGACCTATGACTTCCATGGCT
CCTGGGAGCATACCACAGGCCACGTCAGTCCTCTCTACAAAGGAAACAATGAGACAGGA
CCTGCCGAATACAGTAACACTGATGCTGCCGTGAAAAACTGGATAAGCAAGGGGGCCCC
AGCTGAGAAGATCATCATGGGGATCCCCACCTACGGGCGGGGTTTCACCCTCTCTTCCT
CGGACTCGGATGTTGGCGCACCTACTTCTGGGCCTTCAACTGCAGGCACTTTCACACGG
GAGGCAGGATTCTTGGCCTACTATGAGATCTGTACTTTTCTGAAAGGTGCCACCACCAA
AAGGATTAAGGATCAGAAGGTGCCTTATTCCTTCAAGGACAAGGAATGGGTGGGATATG
ATGACATGGAGAGCATTACAACTAAAGTTCAGTACCTAAAGAACAACAGTCTGGGAGGT
GCCATGATTTGGGCTCTTGACCTGGATGACTTCAGTGGCTCTTTCTGCAATCAAGGAGC
ATACCCTCTTCTAAACACCCTGAAGAAATTGGGATGTGGGATGGCACAACCAACAGCTG
CTTCCACCACTGCAAGGATGGCACAATCAACAGCTGCTTCAACCACTGCAAGGATGGCA
CGATCAACGGCTGCTTCAACCAGCACCAACCTCAAAACAACTCCTCGGCCTGTTCCAGC
CTCCAAGTTTTGCCTTAATAAGGATGATGGCATCTATCCTGTCCCAGAGGATAACACCA
AGTTTTACATCTGTGCCAACAGGGCCACCTTCAGAATGTCCTGTCCTGATGGACTGGTT
TATAATGGCACATGCAAGTGTTGCAACTGGCCTTAA
>Alligator_mississippiensis_Chia1
ATGGGCAAGCTTCTGCTTCTGACTGGTCTGGCCGTTCTGCTGCAGCTGCAGCTGGCCTC
ATCCACCAACATTGTGTGTTACTTCACCAACTGGTGCCAGTACCGGCCTGGAATTGCAC
GTTACATGCCAGAAGATGTTGACCCATGCCTGTGTACTCACATTATCTATGCCTTCGCC
GGCATGCAAAACAACCAGATCCAGACCATCGAACCAAATGATGTGGCCCTCTACGCTGG
CGTCAATGGACTCAAGAGCTACAATCCGGACCTGAAAACCCTGCTGTCTGTTGGAGGCT
GGAATTTCGGTACCCAAAAGTTTTCCACCATGGTTTCCAATGCTCAGAACCGGCAGACC
TTCATCCAGTCCGCCATCACTTTCCTTCGCAAGTACAGCTTTGATGGTCTGGACATTGA
CTGGGAATATCCTGGTAACAGAGGCAGCCCCGCTGATACTCAGCAGCTCTTCACTGTCT
TGCTGAAGGAAATGTATGAAGCTTTCGAGGCAGAGGCTACAAAGAGCAACAAACCCAGG
CTGCTGATCTCTGCCGCAGTGTCTGCTGGTGTGGGCACCATTGAAACTGCCTATCAGAT
CCCTCAGATGTCCAAGTACATGGACCTGATCAATGTGATGACCTACGACCTGAGGGGCT
CCTGGGAAGGCTTCACAGGGGAGAACAGCCCTCTCTATGCAGGTCCTGCTGACCAGGGC
TCATACATTTACTTCAATGTGAACTACTCCATGAATTACTGGAAGGACAACGGTGCTCC
CGCTGAGAAGCTGATGGTTGGATTCGGGGCTTATGCCCGCACCTTCACACTCAGCAACC
CCTCTAACCATGGCCTGGCAGCTCCAACCGCTGGCCCAGGCGCAGCAGGAGAATACACC
CAGTCAGCCGGGACCCTAGCCTACTTTGAGGTCTGCCAGTTCCTGAAAAGTGGGGCTAC
CACAGTGTGGAATGCTCCTCAGGAGACCCCGTATTCCTACAAAGGAAATCAGTGGATCG
GCTATGACAACCCGAAGAGCTTTGCCATCAAGGCTAAGTGGCTGCTGGAGAACAAGTTT
GGAGGTGCCATGGTTTGGGCTATTGACCTGGATGACTTCCTCGGCACGTTCTGTGGGGA
AGGCAAATACCCTCTGATGAATTCCTTGAAATCTGCCCTTGGCGTCACCACCCCCAACT
GCAAGGTTCCAGCCACCACTCTTGGCACTGCTGCTAACCAGCCAGCCAAGCCAGCTACA
GCTGCTCCCCCTGCCCCTGCTACCACCTCTGGTGGCAAACCAAGCTCTGGTGGCAAACC
AAGCTCTGGTGGCAGCCCAAGCGGTGGCAACAGCAACTTCTGTGCTGGCAAGGCCAGTG
GCCTCTACCCAGATCCCACCGATAAGAACAGCTTCTACAACTGCGTGAATGGAAGGACC
TTCCAGGAGCACTGTGCGAATGGCCTGGTCTTTGACACCAGCTGCTCCTGCTGCAACTG

GGCATGA
>Alligator_mississippiensis_Chia2
ATGGTTTCCACTGCTCAGAACCGGCAGACCTTCATCCAGTCTGCCATCACTTTCCTTCG
CAAGTATGGTTTTGATGGTCTCGACATTGACTGGGAATATCCTGGTAACAGAGGCAGCC
CTGCTGATACTCAACAGCTCTTCACTGTCTTGATGCAGGAAATGTATGAAGCTTTCGAG
GCAGAGGTTACAAAGAGCAACAAACCCAGGCTGCTGATCTCTGCCGCAGTGTCTGCTGG
CCTGGGCACCATTGAAACTGCCTATCAGATCCCTCAGATGTCCAAGTACATGGACCTGA
TCAATGTGATGACCTACGACCTGAGGGGCTCCTGGGAAGGCTTCACAGGGGAGAACAGC
CCTCTCTATGCAGGTCCTGCTGACCAGGGCTCATACATTTACTTCAATGTGAACTACTC
CATGAATTACTGGAAGGACCACGGTGCTCCCGCTGAGAAGCTGATGGTTGGATTTGGAG
CTTATGCCCGCACCTTCACACTCAGCAACCCCTCTAACCATGGCCTGGCAGCTCCGACA
TCTGGCCCAGGCGCAGCAGGAGAATACACCCAGTCAGCCGGGACCCTAGCCTACTTTGA
GGTCTGCCAGTTCCTGAAAAGTGGGGCTACCACAGTGTGGAACGCTCCTCAGGAGACCC
CGTATTCCTACAAAGGAAATCAGTGGATTGGCTATGACAACCCGAAGAGCTTTGCCATC
AAGGCTAAGTGGCTGCTGGAGAACAAGTTTGGAGGTGCCATGGTTTGGGCTATTGACCT
GGATGACTTCCTTGGCACGTTCTGTGGGGAAGGCAAATACCCTCTGATGAATTCCTTGA
AATCTGCCCTTGGCGTCACCACCCCCAACTGCAAGGTTCCAGCCACCACTCTTGGCACT
GCTGCTAACCAGCCAGCCAAGCCAACCACAGCTGCCCCTGCTACCAAAAGCACTGAAAG
CTCGGGTGGCAAACCAAGCTCTAGTGAAAAACCAAGCTCTGGTGGCAAACCAAGCTCTG
GTGGCAGCCCAAGTGGTGGCAACAGCAACTTCTGTGCTGGCAAGGCCAGTGGCCTCTAC
CCAGATCCCACCGATAAGAACAGCTTCTATAACTGCGTGAATGGAAGGACCTTCCAGGA
GAACTGTGCGAATGGCCTGGTCTTCGACACCAGCTGCTCCTGCTGCAACTGGGCATGA
>Alligator_mississippiensis_Chia3
ATGGCAAAGTTGTTCCTTCTCACAGGCTTGGTCCTGCTGCTGAATGCACAGATAGGCTC
TGCCTATGTGCTTTCATGCTATTTCACCAACTGGGGCCAATATAGACCTAGCCCAGGGA
AATACTTCCCAAAGGACATTGACCCATGCCTGTGTACTCACCTGCTCTATGCCTTTGCT
GGAATGACCAACAATGAGATTGCAACCATCGAGTGGAATGACGTGACCCTTTACAAATC
TTTCAATGACCTGAAAAACCAGAACTCCGAGCTGAAGACTCTCCTGTCCATCGGAGGCT
GGAATTTTGGAACAGCACCATTCACAGCCATGGTGGCCAGTTCTGCCAACCGCCAGACC
TTCATTAACTCCGTTATCAGTTTCCTGCGCGAGTATGAATTTGATGGGCTGGACATTGA
CTGGGAATACCCTGGCTCCAGAGGCAGCCCTTCTGTGGACAAGGAACTCTTCACCGTGC
TGGTCAAGGAATTGCTGGAAGCCTTCGAGCAAGAAGCCAAGAAAGTTAACAAACCAAGA
CTCCTGGTCACTGCTGCTGTTGCTGCAGGGCTTTCCAACATCGAGGCTGGCTACCAGAT
TGCTGAGCTGGGCCAGTACCTTGACTTCTTCCATGTGATGACATATGACTTCCATGGCT
CTTGGGATGGATTCACTGGAGAGAACAGCCCTCTCTACAAAGGACCAGCTGACAAAGGC
AGCTATGTCTACTTCAATGTGGACTATGCTATGAACTACTGGAAGAGCAATGGTGCCCC
CGCTGAGAAGCTCGTTGTTGGATTCCCAACCTACGGCCACACCTTCATGCTGAGAGATG
CCTCCGACACCGCCGTTGGCGCCCCAATAAATGGACCTGGGCCTGCTGGACCATACACA
AGACAGTCTGGCTTCCTGGCTTACTATGAGATCTGCACCTTCCTGAAGAACGGAGCCAC
CCAGGTTTGGTACACCCCTCAGGATGTGCCCTATGCTTACAAGGGTGATGAGTGGTTGG
GCTATGACAACCAGAAGAGCTTTGAGATCAAGATTGAATGGCTGAAGAAGAGCGGATTT
GGAGGTGCTGTGGTTTGGTCCCTTGACCTGGATGACTTCACTGGCACTTTCTGCGGCGA
GGGCAAATACCCCTTGATCACCACCCTGAAGACTGGTCTGGGACTGCAGAGCTCTACCT
GCAAGCCCCCAGCTCAGCCCCTTCCTCCGGTCACCAAGCCTCCCAGTACTAGTGCCAGC
GGCAGTGGCAGCGGTAGTGGCAGTGGCAGTGGCAGCGGCAGCTCTGGTGGAAGCCCAGG
CAGCGGCTCTGGAGGCAGTGGCTTCTGTGCCGGCAAAGCCAGTGGCCTCTACCCAGACC
CCACCAACAAGAACAGCTTCTACAACTGCGTGAATGGGGAAACCTACCAAGAGAACTGC
AGCTCCGGTCTTGTCTTTGATACCAGCTGCTCCTGCTGCAACTGGGCGTGA
>Alligator_mississippiensis_Chia4
ATGGCGAAGCTGATCCTTCTCACTGGTTTGGTGTTCCTGTTGAATGCACAAATAGGCTC
TGCCTATGTGCTTTCATGCTATTTCTCCAACTGGGGCCAATACAGACAAGGCCCAGGGA
AATACCTCCCAAAGGACATTGACCCATGCCTGTGTACTCACCTGCTCTATGCCTTTGCT
GGAATGACCAACAATGAGATCACAACCATCGAGTGGAACGACGTGACCCTTTACCAATC
CTTCAATGGCCTGAAAAACCAGAACTCCAACCTGAAGACTCTCCTGTCCATCGGAGGCT

GGAATTTTGGAACAGCAAAATTCACAGCCATGGTGGCCAGTTCTGCCAACCGCCAGACC
TTCATTAACTCCGCTATCAAATTCCTGCGCCAGTATGGTTTTGACGGGCTGGACATTGA
CTGGGAATACCCTGGCGCCAGAGGCAGCCCAGCTCAGGACAAGCAACTCTTCACCGTGC
TGATTAAGGAAATACTGGAAGCCTTCGAGCAGGAAGCCAAGCAAGTTAACAAACCAAGA
CTCCTGATCACTGCTGCTGTTGCTGGAGGGCTTTCCAACATTCAGGGTGGCTACCAGAT
TGCTGAGCTGGGCCAGTACCTTGACTACTTCCATGTGATGACATATGACTTCCATGGCT
CTTGGGAAGGATACACTGGAGAGAACAGCCCTCTCTACCGAGGACCTGCTGACCAAGGC
AGCTACATCTACTACAATGTGGAGTCTGCTATGAATTACTGGAAGAGCAATGGTGCGCC
CGCTGAGAAGCTCGTTGTTGGATTCCCAGCCTACGGACACACCTACCTCCTGAGCAACC
CCTCCAACACTGCCGTTGGTGCTCCGACATCAGGACCTGGGCCTGCTGGACCGTACACA
AAGCAAACCGGCTTCCTGGCTTACTATGAGATCTGCACCTTCCTGAAGAACGGAGCCAC
CCAGGCTTGGGACACCCCTCAGGATGTGCCCTACGCTTACAGCGGCAATGAGTGGCTGG
GCTATGACAACCAGAAGAGCTTTCAGATCAAGGTTGATTGGTTGAAGAAGAACAACTTT
GGAGGCGCTATGGTTTGGACCCTCGACCTGGATGACTTTACTGGCAGTTTCTGTGGCCA
GGGCAAATACCCCTTGATCACCACCCTGAAGAACAGCCTGGGGCTGCAGAGCTCTGGCT
GCTCAGCCCCTGCTCAGCCCCTTCCTCCAGTCACTCAGGCCCCCAGCAGCGGAAGTGGC
AGCGGCAGTGGCAGCGGGAGCTCTGGTGGCAGTGGCTTCTGCACTGGCAGGGCTAATGG
CCTTTACCCAGACCAAAGTGACCAGAACAGCTTCTACCAGTGCTTGAATGGGCAAACCT
ATCTCCAGCATTGCCAGGCCGGTCTTGTCTTTGATGCCAGCTGCTCCTGCTGCACCTGG
GCATGA
>Alligator_mississippiensis_Chia5
ATGGGGAAGATGATGGTATGGGCTGGTTTGGTCATTCTGCTGCAGCTGGGCTCTGCCTA
CAAACTTGTTTGCTACTTCAACAACTGGTCTCAGTTCAGGCCAGGAGCAGCTAAATACA
CTCCTGAAGATGTGGACCCATTTTTGTGTACCCACTTGATTTATTCCTTTGCTGGAATA
AAGGACCATAAGATCACCACTACGGAGTGGAACGATGAAATCCTCTATAGTCAGTTTAA
TGCTCTAAAGAACAGAAATAAAAATCTTGTCACCCTTCTGGCTGTTGGAGGTGGAAACT
TTGGCAGCCACAAATTTACTGCCGTGGTGTCCTCTGCTGCCAACCGTAAAACATTCATT
GATTCAGTGATTGCTTTCCTTCGCAAGCACAAGTTTGATGGGCTGGACCTTGATTGGGA
ATTTCCAGCTTCCGGAGGAAGTCCTCCAGAGGATAAGCACCTCTTCACCATCCTGGTTC
AGGAAATGGTAGCAGCTTTTGCCAAGGAGGGTCAACAAACAGGACGTCCGAGGCTGTTG
CTTTCATCAGCTGTATCAGGCGTCAAAGGTATCGTCGATACTGCGTATGAAACTGCTGC
ACTAGGAAGGAGTCTCGATTTTATCAACGTGATGACCTACGACTTCCACGGTAGCTGGA
GTTCAGTCACAGGACACAACAGTCCCCTGTACAAAGGCTCGAGTGACAAAGTTCCCTTC
TACAATGGTGCTTATGCTATGAAATACTGGGAAGACAATGGCGTCCCAGCTGAGAAGCT
CCTCATGGGATTTCCGACGTATGGACGAACTTTCCGACTTTCTACTGGGAATACTGGTG
TTGGTGCTCCTGCTTCTGGTCCTGGTGCTCCTGGTGCTTATACCCGATCAGCTGGTGTC
TTGGCTTACTTTGAGGTCTGCACGTTTTTAAAAGGAGCTACCACCAAATGGATTGAAGC
ACAAAAAGTCCCATATGCCTTCAAAGACAGAGAATGGGTTGGATATGACAACGAGAGGA
GCTTTGAGATAAAGGCCAACTTTATAAAGGAGCAGCATTATGGGGGTGCCATGGTTTGG
GCTCTTGGCATGGATGACTTCTCTGGCTCTTTCTGTGGGGCAGGAGCCAACCCACTCCT
AAAGAAGCTGAAGACTGTCCTTGGAAACTGA
>Alligator_mississippiensis_Chia6
ATGAGAAAGGTGCTGCTGTGGATGGGCTTGGCCATTTTGCTGCAGTTGCAGCTGGGCTC
TGCCTATAGACTTGTGTGTTACTTCACTAACTGGGCTCAGTACAGACCAGATCCTGCCA
AGTACTTCCCAAACAATATAGACCCCTACCTATGCACCCACTTGATCTATGCTTTTGCA
AAGATGGAAGAAAATAAGATCGCCCCATTTGAATGGAATGATGAAAGCAGACTCTTCCC
AGAATTTCAAGCGCTGAAGAAAAGGAACAACAGGCTGGTGACCCTGCTGGCCATTGGAG
GGTGGAACTTTGACATGAAGATGTTCACTCAAATGGTTGCCTCACAAGCGAATCGTAAG
ACCTTCGTTGACTCTGTGGTTGCATACCTCCGCAAATTTGAGTTTGATGGGATTGACCT
GGATTTCGAATACCCAGGTTCCCGAGGCAGCCCCCCTGAAGACAAGCATCGCTTCACCA
TCTTGATTCGGGAAATGCTGGAAGCCTTCAAGGCGGAAGCCACAAGCACTGGAAAGCCA
CGACTGTTGATTACAGCAGCAGTAGCTGCCGGCAAAGGAACCATTGATGCTGGCTATGA
GATAGCTGAGATAGGGAAGCTCCTGGATTTCATCAGCGTGATGACCTATGACTTCCATG
GAGGCTGGGATCCGGTCACAGGACACAACAGCCCCCTGTATCAAGGCTCCACTGACCAG

GGTGATATGAAGTATTTCAACAGTAAATATGCCATGGAGTACTGGAACAACAAGGGTGT
CCCAGCTGAGAAGCTCCTTATGGGGCTCCCCACCTACGGGCGCACCTTCAAGCTCAGCA
GCAGTGTCACATCTGTGGGAGCGCCTGCATCTGGGGCTGGCTCTGCTGGGCCTTACACC
CGTGAGGCTGGCTTTTGGGCCTACTACGAGATTTGCACGTTCCTACGGAGTGCTTCAGT
ATGCTGGATTAATGACCAGAAAGTCCCATACGCCTACAAAGGGAACGAATGGGCTGGGT
TTGACAATGTTTGCAGCTACAGACACAAAGTCAAGTACCTGAAGGAGAAGAAGTTTGGA
GGAGCCATGGTCTGGACAATTGACCTGGATGACTTCCTGGGCACTTTCTGCAATGAAGG
AAAATATCCTCTGATAACTGAGCTGAAGAGACTGCTTGAAACAAATGAACCAGTTGTTG
CTGATTGTCCTAAAGACGCATCCTGCAACACGGGTGGAGGGACGACAACTCCTACACCT
TCACCTCCTGCTGGTCCCACGCCTAACAGCGAGTTTTGTTCTGGGAAAGCAGACGGAAC
CTATGCTGACCCGGCAGACCGTACGAAATTCTACATGTGTGCTGGGGGCAGAACATACA
GCTTCTCCTGTCAAGCAGGCCTGGTCTTTGATGAGAGCTGCACGTGCTGCAACTATCCC
AAATATTAA
>Alligator_mississippiensis_Chia8
ATGAGAAACGCGCTGCTGTTGATCGGCTTGGCCGTTTTGCTGCAGTTGCAGCTGGGCTC
TGCCTACAAACTTGTGTGCTACTTCACTAACTGGGCCCAGTATAGGCCAGAACCTGCCA
AGTACTTCCCAAACAATGTGGACCCCCATTTATGCACCCACTTGATGTACGCCTTTGCA
ACAATGAATGAAAATAAGATTGCACCCTATGAATGGAATGATGAGGACAGACTCTTTCC
AGCATTTCAAGCACTAAAGACAAGTAATGACAAGCTGGTCACCCTGCTGGCCATCGGCG
GGTGGAACTTTGGCACTCAAAAGTTCACTCAAATGGTGGCATCACAAGCAAATCGTAAG
ACCTTCATTGACTCCGTGATTGCGTACCTCCGCAAATTTGGGTTTGATGGGATCGACTT
GGATTTCGAATACCCAGGTTCCCGAGGCAGCCCCCCTGAAGACAAGCATCGCTTCACCA
TCTTGATTCAAGAAATGCTGGATGCCTTCACAGCTGAAGCTGCAAGTGCCAACAAGCCA
CGTCTATTGATTACAGCAGCAGTATCTGCTGGGAAAGCAACTATTGATGCTGGCTATGA
GATTGCTGCGATAGGGGGGCTCCTGGATTTCATCAGCGTGATGACCTATGATTTCCATG
GAGGCTGGGATCCAGTCACAGGACACAACAGTCCCTTGTATGAAGGCTGCAAGGACCAG
GGTGACTTCAAGTTTTTCAACTGCAAATATGCCATGGAGTACTGGAAGAACAATGGCGC
TCCACCTGAGAAGCTCATCATGGGTTTCCCCACCTATGGGCGCACCTTCAGGATCAGTG
GCACGGACCATTGTGTTGGAGCTCCTGCATCTGGGGCAGGCTCTTCTGGGCCCTACACC
CGGGAAGCTGGCTTTTGGGCATATTATGAGATTTGCACCTTCCTGAAGACGGCTCAAGT
ACGTTGGATGTGTGACCAGCAAGTCCCATATGCCTATAAAGATAATGAGTGGGTTGGAT
TTGACAATCTTTGCAGCTACCGACACAAAGTCCAATACTTGAAGGAAAACAATTTTGGA
GGAGCCATGGTCTGGGCAATTGACTTGGACGATTTCCTGGGCACTTTCTGCAACGAAGG
AAAATATCCTCTGATAAGTGAGCTGAAGAGACTCCTTGAAACGAATGAGCCAATTGATC
TAAACTGCCATAACGACATCCCTGATGATGACTGCTGTTCATGTGGCACTGCTCCACCT
GTTTCACCTGATCCACCTGTTTCACCTGATCCAACTGATCCTTCTTCACCTACAACAAC
ACTTCCACCACCTACACCTGAGAAACCAACAGTCAATCCTGGTGATAAGACATTTTGCA
GTGATAAACAAGATGGCATATATGCTGACCCTGAAGATCAGGCATCCTTCTACCAATGT
GCTGCAGGCACTTCTTACCATTTCACCTGTGCAGGTAGCCTGGTCTATGATGAGATCTG
CAAGTGCTGCAACTATCCCTAA
>Mus_musculus_Chia1
ATGGCCAAGCTACTTCTCGTCACAGGTCTGGCTCTTCTGCTGAATGCTCAGCTGGGGTC
TGCCTACAATCTGATATGCTATTTCACCAACTGGGCCCAGTATCGGCCAGGTCTGGGGA
GCTTCAAGCCTGATGACATTAACCCCTGCCTGTGTACTCACCTGATCTATGCCTTTGCT
GGGATGCAGAACAATGAGATCACCACCATAGAATGGAATGATGTTACTCTCTATAAAGC
TTTCAATGACTTGAAAAACAGGAACAGCAAACTGAAAACCCTCCTGGCAATTGGAGGCT
GGAACTTTGGAACTGCTCCTTTCACTACCATGGTTTCCACTTCTCAGAACCGCCAGACC
TTCATTACCTCAGTCATCAAATTTCTGCGTCAGTATGGGTTTGATGGACTGGACCTGGA
CTGGGAATACCCAGGCTCACGTGGGAGCCCTCCTCAGGACAAGCATCTCTTCACTGTCC
TGGTGAAGGAAATGCGTGAAGCTTTTGAGCAGGAGGCTATTGAGAGCAACAGGCCCAGA
CTGATGGTTACTGCTGCTGTAGCTGGTGGGATTTCCAACATCCAGGCTGGCTATGAGAT
CCCTGAACTTTCTAAGTACCTGGATTTCATCCATGTCATGACATATGACCTCCATGGCT
CCTGGGAGGGCTACACTGGGGAGAATAGTCCTCTTTACAAATACCCTACTGAGACTGGT
AGCAATGCCTACCTCAATGTGGATTATGTCATGAACTATTGGAAGAACAATGGAGCCCC

AGCTGAGAAGCTCATTGTTGGATTCCCAGAGTATGGACACACCTTCATCCTGAGAAACC
CCTCTGATAATGGAATTGGTGCCCCTACCTCTGGTGATGGCCCTGCTGGGCCCTATACC
AGACAGGCTGGGTTCTGGGCCTACTATGAGATTTGCACCTTTCTGAGAAGTGGAGCCAC
TGAGGTCTGGGATGCCTCCCAAGAAGTGCCCTATGCCTATAAGGCCAACGAGTGGCTTG
GCTATGACAATATCAAGAGCTTCAGTGTTAAGGCTCAGTGGCTTAAGCAGAACAATTTT
GGAGGTGCCATGATCTGGGCCATTGACCTTGATGACTTCACTGGCTCTTTCTGTGATCA
GGGAAAATTTCCTCTGACTTCTACTTTGAACAAAGCCCTTGGCATATCCACTGAAGGTT
GCACAGCTCCTGACGTGCCTTCCGAGCCAGTGACTACTCCTCCAGGAAGTGGGAGTGGG
GGTGGAAGCTCCGGAGGAAGCTCTGGAGGCAGTGGATTCTGTGCCGACAAAGCAGATGG
CCTCTACCCTGTGGCAGATGACAGAAATGCTTTTTGGCAGTGCATCAATGGAATCACAT
ACCAGCAGCATTGTCAAGCAGGGCTTGTTTTTGATACCAGCTGTAATTGCTGCAACTGG
CCA
>Mus_musculus_Chit1
ATGGTGCAGTCCCTGGCCTGGGCAGGTGTGATGACTCTGCTGATGGTCCAGTGGGGCTC
TGCTGCAAAACTGGTCTGCTACCTCACCAACTGGTCCCAGTACCGGACGGAGGCAGTTC
GGTTCTTTCCCAGGGATGTGGATCCCAACCTGTGTACCCACGTCATCTTTGCTTTTGCT
GGAATGGACAACCATCAGCTCAGCACTGTGGAGCACAATGACGAACTTCTCTACCAGGA
GCTGAACAGCCTAAAGACTAAGAACCCCAAGCTCAAGACCCTGTTAGCCGTTGGAGGCT
GGACCTTTGGTACCCAGAAGTTCACAGACATGGTGGCCACCGCCAGCAACCGGCAGACC
TTTGTGAAGTCAGCCCTAAGTTTCCTGCGCACTCAAGGTTTTGATGGCCTTGACCTTGA
CTGGGAGTTCCCAGGTGGACGTGGGAGCCCCACAGTAGACAAAGAGAGATTCACAGCCC
TGATACAGGACTTGGCCAAAGCCTTCCAGGAGGAAGCCCAGTCCTCAGGGAAGGAACGC
CTCCTTCTGACTGCAGCTGTACCGAGTGATCGAGGCCTGGTGGATGCTGGCTACGAGGT
GGACAAGATTGCCCAGAGCTTGGATTTCATCAACCTTATGGCCTACGACTTCCACAGCT
CCTTGGAAAAGACCACAGGGCATAACAGCCCCCTCTACAAAAGGCAAGGAGAAAGTGGG
GCAGCCGCTGAGCAAAACGTGGATGCTGCTGTGACGCTCTGGCTGCAGAAGGGGACCCC
AGCCAGCAAACTGATCCTTGGCATGCCTACCTATGGACGCTCTTTCACCTTGGCCTCCT
CGTCAGACAATGGAGTTGGGGCCCCAGCCACAGGGCCTGGTGCCCCAGGCCCCTATACG
AAGGACAAAGGGGTCCTGGCTTACTATGAGGCCTGCTCCTGGAAGGAAAGACACAGAAT
CGAGGACCAGAAGGTGCCTTACGCCTTCCAGGACAACCAGTGGGTGAGCTTTGACGACG
TGGAAAGCTTCAAAGCCAAGGCTGCCTACCTGAAACAGAAGGGGCTGGGAGGAGCCATG
GTCTGGGTCCTGGACTTGGATGACTTCAAGGGTTCCTTCTGCAACCAGGGCCCGTACCC
TCTCATCCGGACACTACGGCAGGAACTAAATCTTCCATCCGAGACTCCAAGGAGCCCAG
AACAGATAATACCTGAGCCACGCCCATCTTCTATGCCAGAGCAGGGACCCAGCCCAGGG
CTAGATAACTTCTGCCAAGGCAAAGCTGATGGGGTCTACCCCAACCCTGGAGACGAGTC
CACTTACTACAACTGTGGAGGAGGGCGGCTGTTCCAGCAGAGCTGTCCTCCAGGCCTGG
TGTTTAGAGCCTCTTGCAAATGTTGTACCTGGAGC
>Homo_sapiens_CHIT1
ATGGTGCGGTCTGTGGCCTGGGCAGGTTTCATGGTCCTGCTGATGATCCCATGGGGCTC
TGCTGCAAAACTGGTCTGCTACTTCACCAACTGGGCCCAGTACAGACAGGGGGAGGCTC
GCTTCCTGCCCAAGGACTTGGACCCCAGCCTTTGCACCCACCTCATCTACGCCTTCGCT
GGCATGACCAACCACCAGCTGAGCACCACTGAGTGGAATGACGAGACTCTCTACCAGGA
GTTCAATGGCCTGAAGAAGATGTTCACAGATATGGTAGCCACGGCCAACAACCGTCAGA
CCTTTGTCAACTCGGCCATCAGGTTTCTGCGCAAATACAGCTTTGACGGCCTTGACCTT
GACTGGGAGTACCCAGGAAGCCAGGGGAGCCCTGCCGTAGACAAGGAGCGCTTCACAAC
CCTGGTACAGGACTTGGCCAATGCCTTCCAGCAGGAAGCCCAGACCTCAGGGAAGGAAC
GCCTTCTTCTGAGTGCAGCGGTTCCAGCTGGGCAGACCTATGTGGATGCTGGATACGAG
GTGGACAAAATCGCCCAGAACCTGGATTTTGTCAACCTTATGGCCTACGACTTCCATGG
CTCTTGGGAGAAGGTCACGGGACATAACAGCCCCCTCTACAAGAGGCAAGAAGAGAGTG
GTGCAGCAGCCAGCCTCAACGTGGATGCTGCTGTGCAACAGTGGCTGCAGAAGGGGACC
CCTGCCAGCAAGCTGATCCTTGGCATGCCTACCTACGGACGCTCCTTCACACTGGCCTC
CTCATCAGACACCAGAGTGGGGGCCCCAGCCACAGGGTCTGGCACTCCAGGCCCCTTCA
CCAAGGAAGGAGGGATGCTGGCCTACTATGAAGTCTGCTCCTGGAAGGGGGCCACCAAA
CAGAGAATCCAGGATCAGAAGGTGCCCTACATCTTCCGGGACAACCAGTGGGTGGGCTT

TGATGATGTGGAGAGCTTCAAAACCAAGGTCAGCTATCTGAAGCAGAAGGGACTGGGCG
GGGCCATGGTCTGGGCACTGGACTTAGATGACTTTGCCGGCTTCTCCTGCAACCAGGGC
CGATACCCCCTCATCCAGACGCTACGGCAGGAACTGAGTCTTCCATACTTGCCTTCAGG
CACCCCAGAGCTTGAAGTTCCAAAACCAGGTCAGCCCTCTGAACCTGAGCATGGCCCCA
GCCCTGGACAAGACACGTTCTGCCAGGGCAAAGCTGATGGGCTCTATCCCAATCCTCGG
GAACGGTCCAGCTTCTACAGCTGTGCAGCGGGGCGGCTGTTCCAGCAAAGCTGCCCGAC
AGGCCTGGTGTTCAGCAACTCCTGCAAATGCTGCACCTGGAAT
>Homo_sapiens_CHIA
ATGACAAAGCTTATTCTCCTCACAGGTCTTGTCCTTATACTGAATTTGCAGCTCGGCTC
TGCCTACCAGCTGACATGCTACTTCACCAACTGGGCCCAGTACCGGCCAGGCCTGGGGC
GCTTCATGCCTGACAACATCGACCCCTGCCTCTGTACCCACCTGATCTACGCCTTTGCT
GGGAGGCAGAACAACGAGATCACCACCATCGAATGGAATGATGTGACTCTCTACCAAGC
TTTCAATGGCCTGAAAAATAAGAACAGCCAGCTGAAAACTCTCCTGGCCATTGGAGGCT
GGAACTTCGGGACTGCCCCTTTCACTGCCATGGTTTCTACTCCTGAGAACCGCCAGACT
TTCATCACCTCAGTCATCAAATTCCTGCGCCAGTATGAGTTTGACGGGCTGGACTTTGA
CTGGGAGTACCCTGGCTCTCGTGGGAGCCCTCCTCAGGACAAGCATCTCTTCACTGTCC
TGGTGCAGGAAATGCGTGAAGCTTTTGAGCAGGAGGCCAAGCAGATCAACAAGCCCAGG
CTGATGGTCACTGCTGCAGTAGCTGCTGGCATCTCCAATATCCAGTCTGGCTATGAGAT
CCCCCAACTGTCACAGTACCTGGACTACATCCATGTCATGACCTACGACCTCCATGGCT
CCTGGGAGGGCTACACTGGAGAGAACAGCCCCCTCTACAAATACCCGACTGACACCGGC
AGCAACGCCTACCTCAATGTGGATTATGTCATGAACTACTGGAAGGACAATGGAGCACC
AGCTGAGAAGCTCATCGTTGGATTCCCTACCTATGGACACAACTTCATCCTGAGCAACC
CCTCCAACACTGGAATTGGTGCCCCCACCTCTGGTGCTGGTCCTGCTGGGCCCTATGCC
AAGGAGTCTGGGATCTGGGCTTACTACGAGATCTGTACCTTCCTGAAAAATGGAGCCAC
TCAGGGATGGGATGCCCCTCAGGAAGTGCCTTATGCCTATCAGGGCAATGTGTGGGTTG
GCTATGACAACATCAAGAGCTTCGATATTAAGGCTCAATGGCTTAAGCACAACAAATTT
GGAGGCGCCATGGTCTGGGCCATTGATCTGGATGACTTCACTGGCACTTTCTGCAACCA
GGGCAAGTTTCCCCTAATCTCCACCCTGAAGAAGGCCCTCGGCCTGCAGAGTGCAAGTT
GCACGGCTCCAGCTCAGCCCATTGAGCCAATAACTGCTGCTCCCAGTGGCAGCGGGAAC
GGGAGCGGGAGTAGCAGCTCTGGAGGCAGCTCGGGAGGCAGTGGATTCTGTGCTGTCAG
AGCCAACGGCCTCTACCCCGTGGCAAATAACAGAAATGCCTTCTGGCACTGCGTGAATG
GAGTCACGTACCAGCAGAACTGCCAGGCCGGGCTTGTCTTCGACACCAGCTGTGATTGC
TGCAACTGGGCA
>Gallus_gallus_Chia
ATGGCCAAGCTCATTTTGATTACCGGTCTGGCCCTCCTGCTGAACGCCCAAATAGGCTC
TGCCTATGTGCTGTCATGCTATTTCACCAACTGGGCTCAATACAGGCCTGGCCTGGGAA
AATACATGCCAGACAACATTGACCCATGCCTCTGTGACCATCTGATCTACGCCTTTGCG
GGGATGTCCAACAATGAAATCACAACTTATGAATGGAATGATGAGACCCTCTACAAATC
TTTCAATGGACTGAAAAATCAGAATGGAAATCTCAAGACCCTCCTGGCAATTGGAGGAT
GGAATTTTGGAACAGCCAAGTTCTCCACAATGGTTTCCACTCCTGAGAACCGCCAGACC
TTCATCAATTCCGTCATCAAATTCCTGCGCCAGTACCAATTTGATGGGCTGGACATTGA
CTGGGAATACCCTGGATCAAGGGGCAGCCCTTCTCAGGACAAAGGTCTCTTCACCGTCC
TTGTTCAGGAAATGCTGGCCGCCTTTGAGCAGGAAGCCAAGCAGGTGAACAAGCCCCGT
CTCATGATCACAGCTGCCGTTGCTGCAGGACTTTCCAACATTCAGGCTGGCTACCAGAT
TGCTGAGCTCGGAAAGTACTTGGACTATTTCCATGTGATGACTTATGATTTCCATGGCT
CCTGGGATGGACAAACAGGGGAGAACAGCCCTCTGTACAAAGGCCCTGCGGACACTGGT
GACCTCATCTATTTCAACGTTGATTATGCTATGAACTATTGGAAGAGCAATGGTGCTCC
AGCTGAGAAACTCCTGGTTGGATTCCCAACCTATGGACATAGCTACATCCTCAAGAATC
CATCCGACACTGCTGTTGGGGCACCAACATCGGGCCCTGGGCCAGCCGGGCCTTACACA
AGACAGTCTGGTTTCTTAGCTTACTATGAGATCTGCACATTCCTGGACTCTGGAGCCAC
CCAGGCTTGGGATGCTCCTCAGGATGTGCCCTATGCCTACAAGAGCAGTGAATGGGTTG
GCTATGACAACATCAAGAGCTTCAACATCAAGATTGACTGGCTGAAAAAAAACAACTAT
GGTGGTGCTATGGTTTGGTCCCTTGATATGGATGACTTCACTGGCACTTTCTGTAAACA
GGGCAAATATCCCCTGATTACCACCCTGAAGAATGCTCTTGGCCAGCAAAGCAGCAGCT

GTGTGCCCCCAGCTCAGCCCAATCCTCCCATCACTGCAGCTCCTAGCACTGGAAGTGGG
AGTGGGAGTGGAAGTGGAAGTGGGAGCTCAGGTAGCAATACCGGTAGCTCAGGTGGGAG
TGGATTCTGTGCTGGCAAAGCCAATGGCATCTACGCAGATCCAACCAACAAGAGCAAGT
TCTACAACTGCAATAATGGCGAAACCTTTGTGCAGTCGTGCCAGGCCGGTCTCGTCTTT
GATTCCAGCTGTTCCTGCTGCAACTGGGCA
>Gallus_gallus_Like
ATGGCCAAGCTCATTTTGCTTACCGGTCTGGCCCTCCTGCTGAACGCCCAAATAGGCTC
TGCCTACGTGCTGTCATGCTATTTCACCAACTGGGCTCAATACAGGCCTGGCTCGGGGC
GCTTTACAGTAGATAACATCGACCCATGCCTCTGTGACCATCTGATCTATGCCTTCACT
GGGATGTCCAACAATGAAATCACAACCATTGAATGGAACGATGTGACCCTGTACAAATC
CTTCAACGGACTGAAAAATCAGAATGGAAATCTCAAGACCCTCCTGGCAATTGGAGGAT
GGAATTTTGGAACAGCCAAGTTCTCCACAATGGTTTCCACTCCTGAGAACCGCCAGACC
TTCATCAATTCCGTCATCAAATTCCTGCGCCAGTACCAATTTGATGGGCTGGACATTGA
CTGGGAATACCCTGGATCAAGGGGCAGCCCTTCTCAGGACAAAGGTCTCTTCACCGTCC
TTGTTCAGGAAATGCTGGCCGCCTTTGAGCAGGAAGCCAAGCAGGTGAACAAGCCCCGT
CTCATGATCACAGCTGCCGTTGCTGCAGGACTTTCCAACATTCAGGCTGGCTACCAGAT
TGCTGAGCTCGGAAAGTACTTGGACTACTTTCACGTGATGACTTACGACTTCTACAGCT
CTGGGGATGGACAAACAGGGGAGAACAGCCCTTTGTACAGTGGCAATAATGTCTACCTC
AGTGTTGATTATGCTATGAACTATTGGAAGAGCAATGGTGCCTCAGCTGAGAAACTCCT
GGTTGGATTCCCAACCTATGGACATAGCTTCAACCTCCAGAATCCATCCAACACTGCTG
TTGGGGCACCAACATCAGGCCCTGGGCCAGCCGGGCCTTACACAAAGGAAGCTGGGTTG
CTGGCTTACTACGAGATCTGCACATTCCTGGACTCTGGAGCCACCCAGGCTTGGGATGC
TCCTCAGGATGTGCCCTATGCCTACAAGAGCAGTGAATGGGTTGGCTATGACAACATCA
AGAGCTTCAACATCAAGATTGACTGGCTGAAGAAAAACAACTATGGTGGTGCTATGGTT
TGGACTCTTGATCTGGATGACTTCACTGGCACTTTCTGTAAACAGGGCAAATATCCCCT
GATTACCACCCTGAAGAATGCTCTTGGCCAGCAAAGCAGCAGCTGTCTGACCAAT
>Gallus_gallus_M31
ATGGCCAAGCTCATTTTGCTTACCGGTCTGGCCCTCCTGCTGAACGCCCAGATAGGTAC
TGCCTACGTGCTGTCATGCTATTTCACCAACTGGGCTCAATACAGGCCTGGTGTGGGAA
GTTTCATGCCTGATAACATCGACCCATGCCTGTGCACTCACCTGCTATATGCCTTTGCT
GGGATGTCCAACAATGAAATCACAACCATTGAATGGAACGATGTGACCCTGTACAAATC
CTTCAACGGACTGAAAAATCAGAATAAAAACCTGAAGACTCTGCTTTCTATCGGAGGAT
GGAATTTTGGGACAGACAAGTTCTCCACAATGGTTTCCACTCCTGAGAACCGCCAGACC
TTCATCAATTCCGTCATCAAATTCCTGCGCCGGTACCAATTTGATGGGCTGGACATTGA
CTGGGAATACCCTGGATCAAGGGGCAGCCCTTCTCAGGACAAAGGTCTCTTCACCGTCC
TTGTTCAGGAAATGCTGGCCGCCTTTGAGCAGGAAGCCAAGCAGGTGAACAAGCCCCGT
CTCATGATCACAGCTGCCGTTGCTGCAGGACTTTCCAACATTCAGGCTGGCTACCAGAT
TGCTGAGCTCGGAAAGTACTTGGACTACTTTCACGTGATGACTTATGATTTCCATGGCT
CCTGGGATACACAAACAGGGGAGAACAGCCCTCTGTACCAAGGCCCGAGTGACACTGGG
GACAATATCTATTTTAATGTTGATTATGCTATGAATTACTGGAAAAGCAATGGTGCCCC
AGCTGAGAAACTAGTTGTTGGATTCCCAGCCTACGGAAACACTTTCAGACTGCAAAACC
CATCTAATCATGGTCTTGGTGCACCGACTTCAGGACCAGGACCTGCTGGACCTTATACA
CAGGAGGCTGGGACGCTGGCTTACTATGAGATCTGCACACTACTGAATTCTGGAGGCAC
ACAAGTTTGGGATGCTCCTCAGGATGTGCCCTATGCCTACAAAGGTAGCACTTGGGTTG
GCTACGACAATATCAAGAGCTTCAACATCAAGGCTGACTGGCTGAAGAAAAACAACTAT
GGTGGTGCTATGGTTTGGGCCATCGATCTGGATGACTTCACTGGCACTTTCTGTAAACA
GGGCAAATATCCCCTGATTACCACCCTGAAGAATGCTCTTGGTCTGCAAAGCAACAGCT
GTGTGCCCTCAACTCAGCCCAGTCCTACCACCACTGCAGTTCCCTGTAGTACACATGGA
AGTGGAAGTGGAAGTGAGAGCTCAGGTAGCAATACCGATAGCTCAGGTGAGAGTGGATT
CTGTGCTGGCAAAGCCAATGGCATCTACGCAGATCCAACCAACAAGAGCAAGTTCTACA
ACTGCAATAATGGCGAAACCTTTGCGCAGTCGTGCCAGGCCGGTCTCGTCTTTGATTCC
AGCTGTTCCTGCTGCAACTGGGCA
>Numida_meleagris
ATGGCCAAGCTCATTTTGCTTACCGGTCTGGCCCTCCTGCTGAACGCCCAGATAGGCTC

TGCCTATGTGCTGTCATGCTATTTCACCAACTGGGCTCAATACAGGCCTGGCCTGGGAA
AATACATGCCAGACAACATTGACCCATGCCTCTGTGACCACCTGATCTATGCCTTTGCT
GGGATGTCCAACAATGAGATCACAACTTATGAATGGAACGATGTGACCCTCTACAAATC
CTTCAACGGACTGAAAAATCAGAATGGAAATCTCAAGACCCTCCTGGCAATTGGAGGAT
GGAATTTTGGGACAGCCAAGTTCTCCACAATGGTTTCCACTCCTGAGAACCGCCAGACT
TTCATCAACTCTGTCATCAAATTCCTGCGCCAGTACCAATTTGATGGGCTGGACATTGA
CTGGGAATACCCTGGATCAAGGGGCAGCCCTTCTCAGGACAAAGGTCTCTTTACTGTCC
TTGTTCAGGAAATGCTGGCCGCCTTTGAGCAGGAAGCCAAGCAGGTTAACAAGCCTCGC
CTCATGATCACAGCTGCTGTTGCTGCAGGACTTTCCAACATTCAGGCTGGCTACCAGAT
TGCTGAGCTGGGAAAGTACTTGGACTACTTCCACGTGATGACTTATGATTTCTACAGCT
CCGGGGATGGACAAACAGGGGAAAACAGCCCTCTGTACAGTGGCAATAATATCTACCTC
AGTGTTGATTATGCTATGAACTATTGGAAGAGCAATGGTGCTCCAGCTGAGAAACTCCT
TGTTGGATTCCCAACCTACGGACATAGCTACATCCTCCAGAATCCATCCAACACTGCTG
TTGGGGCACCAACATCGGGCCCTGGGCCAGCTGGACCTTATACAAGGCAGTCTGGTTTC
TTAGCTTACTACGAGATCTGCACATTCCTGGACTCTGGAGCCACCCAGGCTTGGGATGC
TCCACAGGATGTGCCCTATGCCTACAAGAGCAGTGAATGGGTTGGCTATGACAACATCA
AGAGCTTCAACATCAAGGTTGACTGGCTGAAGAAGAACAACTATGGTGGTGCTATGGTT
TGGACTCTTGATATGGATGACTTCACTGGCACTTTCTGCAAACAGGGCAAATATCCCCT
GATTAACACCCTGAAGAACGCTCTTGGTCAGCAAAGCAGCAGCTGCATGACCTAT
>Phasianus_colchicus_3
ATGGCCAAGCTCATTCTGCTTACCGGTCTGGCCCTCCTGCTGAACGCCCAGATAGGCAC
TGCCTATGTGCTGTCATGCTATTTCACCAACTGGGCTCAATACAGGCCTGGCGTGGGAA
GTTTCATGCCTGATAACATTGACCCATGCCTGTGCACTCATCTGCTATACGCCTTCGCT
GGGATGTCCAACAATGAGATCACAACCATTGAATGGAATGATGAGACCCTCTACAAATC
CTTCAATGGGCTGAAAAATCAGAATAAAAACCTGAAGACTCTGCTTTCTATTGGAGGTT
GGAATTTTGGGACAGCCAAGTTCTCCACAATGGTTTCCACTCCTGAGAACCGCCAGACC
TTCATCAACTCCGTCATCAAATTCCTGCGCCAGTACCAATTTGATGGGCTGGACATTGA
CTGGGAATACCCTGGATCAAGGGGCAGCCCTTCTCAGGACAAAGGTCTCTTCACCGTCC
TTGTTCAGGAAATGCTGGCTGCCTTTGAGCAGGAAGCCAAGCAGGTGAACAAGCCCCGT
CTCATGATCACTGCTGCCGTTGCTGCAGGAGTTTCCAACATTCAGGCTGGCTACCAGAT
TGCTGAGCTCGGAAAGTACTTGGACTACTTTCATGTGATGACTTATGATTTCCATGGCT
CCTGGGATACACAAACTGGGGAGAACAGCCCTCTCTACCAAGGCCCAAATGACACTGGT
GACAATATCTATTTCAATGTTGATTATGCTATGAATTACTGGAAAAGCAATGGTGCCCC
AGCTGAGAAACTAGTTGTTGGATTCCCAGCCTATGGAAACACCTTCAGACTGCAAAACC
CATCTAATCACGGTCTTGGTGCACCAACTTCAGGACCAGGACCTGCTGGACCTTACACA
CAGGAGGCTGGGACGCTGGCTTACTATGAGATCTGCACACTACTGAATTCTGGAGGCAC
ACAAGTTTGGGATGCTCCCCAGGATGTGCCCTATGCCTACAAGAGCAGTGAATGGGTTG
GCTACGACAACATCAAGAGCTTCAACATCAAGGCTGAATGGTTGAAGAAGAACAACTAC
GCAGGTGCTATGGTTTGGGCCATTGATCTGGATGACTTCACTGGCACTTTCTGTAAACA
GGGCAGATATCCCCTGATTACCACCCTGAAGAATGCACTTGGTCTTCAAAGCAACAGCT
GTGTGCCCCCAACTCAGCCCAGTCCTACCACCACTGCAGTCCTCTGTAATACACAAGGA
AGTGAAAGTGGGAGTGAGAGTGAGAGCTCAGGTAGCAATACCGGTAGCTCAGGTGGGAG
TGGATTCTGTGCTGGTAAGGCCGATGGCATCTACGCAGATCCAACCAACAAGAGCAAAT
TCTACAACTGCAATAATGGCGAAACCTTCACGCAGACGTGCCAGGCCGGTCTCGTCTTT
GATTCCAGCTGCTCCTGCTGCAACTGGGCA
>Cygnus_olor_1
ATGGCCAAGCTCACTCTGCTTACCGGTCTGGCCCTGCTACTGAACGCCCAGATAGGCAC
TGCCTATGTGCTGTCGTGCTACTTCACCAACTGGGCCCAGTACAGGCCTGGCCTGGGGT
GCTTCACGGTTGACAACATCGACCCATGCCTGTGTGACCATCTGATCTATGCCTTCGCT
GGGATGTCCAACAACGAGATCACGACCATTGAATGGAACAATGAGACCCTCTACAGATC
CTTCAACGGCCTGAAAAACCAGAATGAAAATCTGAAGACCCTCCTGGCAGTGGGAGGAT
GGAATTTTGGGACAGCCAAGTTCTCCGCAATGGTTTCCACTCCGGAGAACCGCCAGACC
TTCATCAATTCCATCATCAAATTCCTGCGCCAGTACCAGTTTGATGGGCTGGACCTTGA
CTGGGAGTACCCTGGCTCCAGGGGCAGCCCGGCTCAGGACAAGGGTCTCTTAACCGTCC

```
TGGTGAAGGAAATGCTGGCCGCCTTTGAGCAGGAAGCCAAGCAGGTTAGCAAACCCCGT
CTCATGCTCACCGCTGCTGCTGCTGCAGGACTTTCCAACATTCAGTCTGGCTACGAGAT
CACTGAGCTCGGCAAGTACCTGGACTACTTCCATGTGATGACTTATGACTTCTATAGCT
CCTGGGATGGAAAGACTGGGGAGAACAGCCCGCTGTATAGTGACGGCAATGCCTACCTT
AGTGTCGACTACGCTATGAACTACTGGAAGAGCAATGGTGCCCCAGCTGAGAAGCTCCT
TGTTGGATTCCCCACCTATGGACATAACTTCATCCTCCAGAACCCATCTAACACAGCTC
TCGGGGCACTGACATCGGGACCTGGGCCTGCTGGACCTTACACAAAGGAGGCTGGGCTG
CTGGCTTACTACGAGATCTGCACGTTCCTGGACTCTGGAGCCACCCAGGCTTGGGATGC
CCCCCAGGACGTGCCCTATGCCTACAAGGGCAACGAATGGGTTGGCTACGACAACATCA
AGAGCTTCAACATCAAGGCTAACTGGCTGAAGAGCAATAACTATGGAGGTGCTATGGTT
TGGACCATTGATCTGGATGACTTCACTGGCTCTTTCTGCAAACAGGGCAAATATCCCCT
GATCAACACTCTGAAGAATGCTCTTGGTCAGCAAAGCAGCAGCTGCACAGCTTAT
>Cygnus_olor_2
ATGGCCAAGCTCACTCTGCTTACCGGTCTGGCCCTCCTGCTGAACGCCCAGATAGGCAC
TGCCTATGTGCTGTCGTGCTACTTCACCAACTGGGCCCAGTACAGGCCTGGCCTGGGAA
AATATATGCCAGACAACATCGACCCATGCCTGTGTGACCATCTGATCTACGCCTTCGCT
GGGATGTCCAACAATGAGATCACAACCTATGAATGGAACGATGAGACCCTCTACAAATC
CTTCAATGGCCTGAAAAACAAGAATGAAAATCTGAAGACCCTCCTGGCAGTGGGAGGAT
GGAATTTTGGGACAGCCAAGTTCTCCGCAATGGTTTCCACTCCGGAGAACCGCCAGACC
TTCATCAATTCCGTCATCAAATTCCTGCGCCAGTACCAGTTTGATGGGCTGGACCTTGA
CTGGGAGTACCCTGGCTCCAGGGGCAGCCCGGCTCAGGACAAGGGTCTCTTCACCGTCC
TGGTGAAGGAAATGCTGGCCGCCTTTGAGCAGGAAGCCAAGCAGGTTAGCAAACCCCGT
CTCATGCTCACCGCTGCTGTCGCTGCAGGACTTTCCAACATTCAGTCTGGCTACGAGAT
CGCTGAGCTTGGCAAGTACTTGGACTACTTCCATGTGATGACTTATGACTTCCATGGCT
CCTGGGATGGAAAGACTGGGGAGAACAGCCCGCTGTACAAAAGCCCTGTTGACACTGGT
GACCTCATCTACTTCAATGTTGACTACGCTATGAACTACTGGAAGAGCAATGGTGCCCC
AGCTGAGAAGCTCCTTGTTGGATTCCCCACCTATGGACATAACTTCATCCTCCAGAACC
CCTCTGACACTGCTGTTGGGGCACCAACATCGGGACCTGGGCCTGCTGGACCTTACACA
AGGCAGGCTGGGTTCTTGGCTTACTACGAGATCTGCACGTTCCTGGACTCTGGAGCCAC
CCAGGCTTGGGATGCCCCCCAGGACGTGCCCTATGCCTACAAGGGCAACGAATGGGTTG
GCTACGACAACATCAAGAGCTTCAACATCAAGATTGACTGGCTGAAGAAGAACAACTAT
GGAGGTGCCATGGTTTGGTCTCTTGATATGGATGACTTCACTGGCTCTTTCTGCAAACA
GGGCAAATATCCCCTGATTACCACCCTGAAGAACGCTCTTGGTCAGCAAAGCAGCAGCT
GCGTGCCCCCGGCTCAACCCAATCCTCCCATCACTTCAGCTCCTAACACTGGAAGTGGA
AGTGGCAGCGGGAGCGGGAGCTCAGGTAGCAATACTGGTGGCACTGGTGGGAGCGGATT
CTGTGCTGGCAAGGCCAATGGCATCTATGCAGATCCGACCAACAAGAGCAAGTTCTACA
ACTGCAACAACGGTGAAACCTACGAGCAGAGCTGCCAGGCCGGTCTGGTCTTTGATCCC
AGCTGCTCCTGCTGCAACTGGGCA
>Cygnus_atratus_1
ATGATTTCGAAGAAGAAAAGCTATCTAATCTTGTTCTTCAACTACTATGGCAGCACCAC
AAAATGGCACAGCCTGATAATCCTCATGCCTCCTGATGGAGCCTTAAGGTATTGGTATC
ATTTTTGTGATACTTTTATCAACTTGGGTAATGGTTGGGCATTTTTGTTGACACAAGAC
TTGTACGAAATTAGAGAAGGGTATAAAAGTGGGAAGCAGCCAGCCCCAGATCAGTTCTG
GTCCAAGATGGCCAAGCTCACTCTGCTTACCGGTCTGGCCCTGCTGCTGAACGCCCAGA
TAGGCACTGCCTATGTGCTGTCGTGCTACTTCACCAACTGGGCCCAGTACAGGCCTGGC
CTGGGGCGCTTCACGGTTGACAACATCGACCCATGCCTGTGTGACCATCTGATAGACGC
CTTCGCTGGGATGTCCAACAACGAGATCACGACCATTGAATGGAACGATGAGACCCTCT
ACAGATCCTTCAACAGCCTGAAAAAACAGAATGGAAATCTGAAGACCCTCCTGGCAATT
GGAGGATGGAATTTTGGGACAGCCAAGTTCTCCGCAATGGTTTCCACTCCGGAGAACCG
CCAGACCTTCATCAATTCTGTCATCAAATTCCTGCGCCAGTACCAGTTTGATGGGCTGG
ACCTTGACTGGGAGTACCCTGGCTCCAGGGGCAGCCCGGCTCAGGACAAGGGTCTCTTC
ACCGTCCTGGTGAAGGAAATGCTGGCCGCCTTTGAGCAGGAAGCCAAGCAGGTTAACAA
ACCCCGTCTCATGCTCACCGCTGCTGTTGCTGCAGGACTTTCCAACATTCAGTCTGGCT
ACGAGATCGCTGAGCTCAGCAAGTACCTGGACTACTTCCATGTGATGACTTATGACTTC
```

TATAGCTCCTGGGATGGAAAGACTGGGGAGAACAGCCCGCTGTATAGTGACGGCAATGC
CTACCTTAGTGTCATAAGTACAGACTACGCTATGAACTACTGGAAGAGCAATGGTGCCC
CAGCTGAGAAGCTCCTTGTTGGATTCCCCACCTATGGACATAACTTCATCCTCCAGAAC
CCCTCTGACACTGCTGTTGGGGCACCAACATCGGGACCTGGGCCTGCTGGACCTTACAC
AAGGCAGGCTGGGTTCTTGGCTTACTACGAGATCTGCACGTTCCTGGACTCTGGAGCCA
CCCAGGCTTGGGATGCCCCCCAGGACGTGCCCTATGCCTACAAGGGCAACGAATGGGTT
GGCTACGACAACATCAAGAGCTTCAACATCAAGGCTAACTGGCTGAAGAGCAACAACTA
TGGAGGTGCTATGGTTTGGACCATTGATCTGGATGACTTCACTGGCTCTTTCTGCAAAC
AGGGCAAATATCCCCTGATCAACACTCTGAAGAATGCTCTTGGTCTGCAAAGCAGCAGC
TGCACAGCTTAT
>Cygnus_atratus_2
ATGGCCAAGCTCACTCTGCTTACCGGTCTGGCCCTCCTGCTGAACGCCCAGATAGGCAA
TGCCTATGTGCTGTCGTGCTACTTCACCAACTGGGCCCAGTACAGGCCTGGCCTGGGAA
AATATATGCCAGACAACATCGACCCATGCCTGTGTGACCATCTGATCTACGCCTTCGCT
GGGATGTCCAACAACGAGATCACGACCATTGAATGGAACGATGAGACCCTCTACAAATC
CTTCAATGGCCTGAAAAACAAGAATGAAAATCTGAAGACCCTCCTGGCAATCGGAGGAT
GGAATTTTGGGACAGCCAAGTTCTCCGCAATGGTTTCCACTCCGGAGAACCGCCAGACC
TTCATCAATTCCGTCATCAAATTCCTGCGCCAGTACCAGTTTGATGGGCTGGACCTTGA
CTGGGAGTACCCTGGCTCCAGGGGCAGCCCGGCTCAGGACAAGGGTCTCTTCACCGTCC
TGGTGAAGGAAATGCTGGCCGCCTTTGAGCAGGAAGCCAAGCAGGTTAGCAAACCCCGT
CTCATGCTCACCGCTGCTGTTGCTGCAGGACTTTCCAACATTCAGTCTGGCTACGAGAT
CGCTGAGCTCGGCAAGTACTTGGACTACTTCCATGTGATGACTTATGACTTCCATGGCT
CCTGGGATGGAAAGACTGGGGAGAACAGCCCGCTGTACAAAAGCCCTGTTGACACTGGT
GACCTCATCTACTTCAATGTTGACTACGCTATGAACTACTGGAAGAGCAATGGTGCCCC
AGCTGAGAAGCTCCTTGTTGGATTCCCCACCTATGGACATAACTTCATCCTCCAGAACC
CCTCTGACACTGCTGTTGGGGCACCAACATCGGGACCTGGGCCTGCTGGACCTTACACA
AGGCAGGCTGGGTTCTTGGCTTACTACGAGATCTGCACGTTCCTGGACTCTGGAGCCAC
CCAGGCTTGGGATGCCCCCCAGGACGTGCCCTATGCCTACAAGGGCAACGAATGGGTTG
GCTACGACAACATCAAGAGCTTCAACATCAAGATTGACTGGCTGAAGAAGAACAACTAT
GGAGGTGCTATGGTTTGGACCATTGATCTGGATGACTTCACTGGCTCTTTCTGCAAACA
GGGCAAATATCCCCTGATTACCACCCTGAAGAACGCTCTTGGTCAGCAAAGCAGCAGCT
GCGTGCCCCCGGCTCAACCCAATCCTCCCATCACTTCAGCTCCTAACACTGGAAGTGGA
AGTGGCAGCGGGAGCGGGAGCTCAGGTAGCAATACTGGTGGCACTGGTGGGAGCGGATT
CTGTGCCGATAAGGCCAATGGCATCTATGCAGATCCGACCAACAAGAGCAAGTTCTACA
ACTGCAACAACGGTGAAACCTACGAGCAGAGCTGCCAGGCCGGTCTGGTCTTTGATCCC
AGCTGCTCCTGCTGCAACTGGGCA
>Anas_platyrhynchos_1
ATGGCCAAGCTCACTCTGCTTACCGGTCTGGCCCTGCTGCTGAATGCCCAGATAGGCAC
TGCCTATGTGCTGTCGTGCTACTTCACCAACTGGGCCCAGTACAGGCCCAGCCCGGGGC
GCTTCACGGTTGACAACATTGACCCATGCCTGTGTGACCATCTGATCTACGCCTTCGCT
GGGATGTCCAACAATGAGATCACGACCATTGAATGGAACGATGAGACCCTCTACAGATC
CTTCAACGGGCTGAAAAACCAGAATGGAAATCTGAAGACCCTCCTGGCAATTGGAGGAT
GGAATTTTGGGACAGCCAAGTTCTCCACAATGGTTTCCACTCCGGAGAACCGCCAGACC
TTCATCAATTCCGTCATCAAATTCCTGCGCCAGTACCAGTTTGATGGGCTGGACCTCGA
CTGGGAGTACCCTGGCTCCAGGGGCAGCCCGGCTCAGGACAAGGGTCTCTTCACTGTCC
TGGTGAAGGAAATGCTGGCAGCCTTCGAGCAGGAAGCCAGGCAGGTTAACAAGCCCCGT
CTGATGATCACGGCTGCTGTTGCTGCAGGACTTTCCAACATCCAGTCTGGCTACGAGAT
CGCTGAGCTCGGCAAGTACCTGGACTACTTCCATGTGATGACTTATGACTTCTATAGCT
CCTGGGATGGAAAGACTGGGGAGAACAGCCCGCTGTACAGTGATGGCAATGCCTACCTC
AGTGTTGATTATGCTATGAACTACTGGAAGAGCAATGGTGCCCCAGCTGAGAAGCTCCT
TGTTGGATTCCCCACCTATGGACATAACTTCATCCTGCAGAACCCATCTAACACAGCTC
TTGGGGCACCAACATCGGGACCTGGGCCTGCTGGACCTTACACAAAGGAGGCTGGGCTG
CTGGCTTACTACGAGATCTGCACATTCCTGGACTCTGGAGCCACCCAGGCTTGGGATGC
CCCCCAGGATGTGCCCTATGCCTACAAGGGCAACGAATGGGTTGGCTACGACAACATCA

```
AGAGCTTCAACATCAAGGCTGACTGGCTGAAGAAGAACAACTATGCAGGTGCTATGGTT
TGGACCATTGATCTGGATGACTTCACTGGCTCTTTCTGCAAACAGGGCAAATATCCCCT
GATCACCACCCTGAAGAACGCTCTTGGCCTGCAAAGCAGCATCCAGTCCTCAATCCCTG
CAGCTCCTAACACTAGAAGT
>Anas_platyrhynchos_2
ATGGCCAAGCTCACTCTGCTTACCGGTCTGGCCCTTCTGCTGAACGCCCAGATAGGCAC
TGCCTATGTGCTGTCATGCTACTTCACCAACTGGGCCCAGTACAGGCCCGGCCTGGGAA
AGTACATGCCAGACAACATCGACCCATGCCTGTGTGACCATCTGATCTACGCCTTCGCT
GGGATGTCCAACAATGAGATCACAACCTATGAATGGAACGATGAGACCCTCTACAAATC
CTTCAACGGCCTGAAAAACCAGAATGAAAATCTGAAGACCCTCCTGGCAATCGGAGGAT
GGAATTTTGGGACAGCCAAGTTCTCCACAATGGTTTCCACCCTGGAGAACCGCCAGACC
TTCATCAATTCCGTCATCAAATTCCTGCGCCAGTACCAGTTTGATGGGCTGGACCTTGA
CTGGGAGTACCCTGGCTCCAGGGGCAGCCCGGCTCAGGACAAGGGGCTCTTCACCATCC
TGGTGAAGGAAATGCTGGCTGCCTTCGAGCAGGAAGCCAAGCAGGTTGGCAAGCCTCGT
CTGATGATCACGGCTGCTGTTGCTGCAGGACTTTCCAACATCCAGTCTGGCTACGAGAT
CGCTGAGCTCGGCAAGTACTTGGACTACTTCCATGTGATGACTTATGACTTCCACGGTT
CCTGGGATGGAAAGACTGGGGAGAACAGCCCGCTGTACGAAAGCCCTGTTGACACTGGT
GAACTCATCTACTTCAACGTTGATTACGCTATGAACTACTGGAAGAACAATGGTGCCCC
TGCTGAGAAGCTCCTGGTTGGATTCCCCACCTACGGACATAACTTCATCCTGCAGAACC
CATCTGACACTGCTGTTGGGGCACCAGCATCAGGACCTGGGCCTGCTGGACCGTACACA
AGGCAGGCTGGATTCTTGGCTTACTACGAGATCTGCACGTTCCTGGACTCTGGAGCCAC
CCAGGCTTGGGATGCCCCCCAGGACGTGCCCTATGCCTACAAGGGCAACGAATGGGTTG
GCTACGACAACATCAAGAGCTTCAACATCAAGATTGACTGGCTGAAGAAGAACAACTTT
GGAGGTGCTATGGTTTGGGCTCTTGATATGGATGACTTCACTGGCACATTCTGCAAGCA
GGGCAAATATCCCCTGATCACCACCCTGAAGAACGGTCTTGGTCAGCAAGGCAGCAGCT
GCGTGCCCCCAGCTCAGCCCAATCCTCCCATCACTTCAGCTCCTAACACTGGAAGTGGA
AGTGGCAGCGGCAGCGGGAGCTCGGGTGGCAATACTGGTGGCACTGGTGGGAGTGGATT
CTGTGCTGGCAAGGCCAACGGCATCTATGCAGATCCGACCAACAAGAGCAAGTTCTACA
CCTGCAACAACGGTGAAACCTACGAGCAGAGCTGCCAGGCCGGGCTGGTCTTTGATCCT
AGCTGCTCCTGCTGCAACTGGGCA
>Anas_platyrhynchos_3
ATGGCCAAGCTCACTCTGCTTACCGGTCTGGCCCTCCTGCTGAACGCCCAGATAGGCAC
TGCCTATGTGCTGTCATGCTACTTCACCAACTGGGCCCAGTACAGGCCCGGCCTGGGAA
GTTTCATGCCAGACAACATCGACCCCTGCCTGTGCACTCACCTGCTGTACGCCTTCGCT
GGGATGTCCAACAATGAGATCACAACCTATGAATGGAACGATGAGACCCTCTACAAATC
CTTCAACGGGCTGAAAAACCAGAATAAAAACCTGAAGACTCTTCTTTCTATTGGAGGAT
GGAATTTTGGAACAGACAAGTTCTCCACAATGGTTTCCACTCCGGAGAACCGACAGACC
TTCATCAATTCCGTCATCAAATTCCTGCGCCAGTACCAGTTTGATGGGCTGGACATTGA
CTGGGAGTACCCTGGCTCCAGGGGCAGCCCGGCTCAGGACAAGGGTCTCTTCACTGTCC
TGGTGAAGGAAATGCTGGCTGCCTTTGAGCAGGAAGCCAAGCAGGTTGGCAAGCCCCGT
CTGATGATCACGGCTGCTGTTGCTGCAGGACTTTCCAACATCCAGTCTGGCTACGAGAT
CGCTGAGCTCGGCAAGTACCTGGACTACTTCAACGTGATGACTTATGATTTCCACGGCT
CCTGGGATGCACAAACTGGGGAGAACAGCCCTCTCTACAAAGGCCCAGGTGACACTGAT
GGCAATATCTATTTCAACGTTGATTACGCTATGAACTACTGGAAGAGCAACGGTGCCCC
AGCTGAGAAGTTAGTTGTTGGGTTCCCCACCTATGGAAACACCTTCAGGCTGCAAAACC
CATCCAACAATGGTCTTGGTGCACCAGCATCGGGACCTGGGCCTGCTGGACCTTACACA
CAGGAGGCTGGGACACTGGCTTACTATGAGATCTGCACTCTGCTAAATTCTGGAGCCAC
CCAAGTTTGGGATGCCCCCCAGGATGTGCCCTATGCCTACAAGGGCAATGAATGGGTTG
GCTACGACAACATCAAGAGCTTCAACATCAAGGCTGACTGGCTGAAGAAGAACAACTAT
GCAGGTGCTATGGTTTGGGCCATTGATCTGGATGACTTCACTGGCACATTCTGCAAGCA
GGGCAAATATCCCCTGATCACCACCCTGAAGAATGCTCTTGGCCTGCAAAGCAGCAGCT
GTGTGCCCCCAGCTCAACCCAATCCTCTGGTGACTGCAGCTCCTGGTACTACAAGTGGA
AGTGGAAGTGGAAGTGGGAGTGGGAGTGGAAGTGGAAGTGGCAGCGGGAGCTCGGGTGG
CAATACTGGTGGCACTGGTGGGAGCGGATTCTGTGCTGGCAAGGCCAACGGCATCTATG
```

CAGATCCGACCAACAAGAGCAAGTTCTACACCTGCAACAACGGTGAAACCTACGAGCAG
AGCTGCCAGGCCGGGCTGGTCTTTGATCCCAGCTGCTCCTGCTGCAACTGGGCA
>Aythya_fuligula_1
ATGGCCAAGCTCACTCTGCTTACCGGTCTGGCCCTGCTGCTGAATGCCCAGATAGGCAC
TGCCTATGTGCTGTCGTGCTACTTCACCAACTGGGCCCAGTACAGGCCCGGCCCGGGGC
GCTTCACGGTTGACAACATTGACCCATGCCTGTGTGACCATCTGATCTACGCCTTCGCT
GGGATGTCCAACAATGAGATCACGACCATTGAATGGAATGATGAGACCCTCTACAGATC
CTTCAACGGGCTGAAAAACCAGAATGGAAATCTGAAGACCCTCCTGGCAATCGGAGGAT
GGAATTTTGGAACAGCCAAGTTCTCCACAATGGTTTCCACTCCACAGAACCGCCAGACC
TTCATCAAGTCCGTCATCAAATTCCTGCGCCAGTACCAGTTTGATGGGCTGGACCTCGA
CTGGGAGTACCCTGGCTCCAGGGGCAGCCCGGCTCAGGACAAGGGTCTCTTCACCGTCC
TGGTGAAGGAAATGCTGGCCGCCTTCGAGCAGGAAGCCAGGCAGGTTGGCAAGCCCCGT
CTGATGATCACGGCTGCTGTTGCTGCAGGACTTTCCAACATCCAGTCTGGCTACGAGAT
CGCTGAGCTCGGCAAGTACCTGGACTACTTCCATGTGATGACTTATGACTTCTATAGCT
CCTGGGATGGAAAGACTGGGGA*GAACAGCCCGCTGTACA*GTGATGGCAATGCCTACCTC
AGTGTTGATTACGCTATGAACTACTGGAAGAGCAATGGTGCCCCTGCTGAGAAGCTCCT
TGTTGGATTCCCCACCTATGGACATAGCTTCAACCTGCAGAACCCATCTAACACAGCTC
TTGGGGCACCAACATCAGGACCTGGGCCCGCGGGACCTTACACAAAGGAGGCTGGGCTG
CTGGCTTACTACGAGATCTGCACGTTCCTGGACTCTGGAGCCACCCAGGCTTGGGATGC
CCCCCAGGACGTGCCCTATGCCTACAAGGGCAACGAATGGGTTGGCTACGACAACATCA
AGAGCTTCAACATCAAGGCTGACTGGCTGAAGAAGAACAACTATGCAGGTGCTATGGTT
TGGACCATTGATCTGGATGACTTCACTGGCTCTTTCTGCAAGCAGGGCAAATATCCCCT
GATCACCACCCTGAAGAACGCTCTTGGCCTGCAAAGCAGCATCCAGTCCTCAATCCCTG
CAGCTCCTAACACTAGAAGT
>Aythya_fuligula_2
ATGGCCAAGCTCACTCTGCTTACCGGTCTGGCCCTCCTGCTGAACGCCCAGATAGGCAC
TGCCTATGTGCTGTCATGCTACTTCACCAACTGGGCCCAGTACAGGCCCGGCCTGGGAA
AGTATATGCCAGACAACATCGACCCATGCCTGTGTGACCATCTGATCTATGCCTTCGCT
GGGATGTCCAACAATGAGATCACAACCTATGAATGGAACGATGAGACCCTCTACAAATC
CTTCAATGGCCTGAAAAACCAGAATGAAAATCTGAAGACCCTCCTGGCAATCGGAGGAT
GGAATTTTGGGACAGCCAAGTTCTCCGCAATGGTTTCCACTCCGGAGAACCGCCAGACT
TTCATCAATTCCGTCATCAAATTCCTGCGCCAGTACCAGTTTGATGGGCTGGACCTCGA
CTGGGAGTACCCTGGCTCCAGGGGCAGCCCGGCTCAGGACAAGGGTCTCTTCACCGTCC
TGGTGAAGGAAATGCTGGCTGCCTTCGAGCAGGAAGCCAAGCAGGTTGGCAAGCCCCGT
CTGATGATCACGGCTGCTGTTGCTGCAGGACTTTCCAACATCCAGTCTGGCTACGAGAT
CGCTGAGCTCGGCAAGTACTTGGACTACTTCCATGTGATGACTTATGACTTCCACGGTT
CCTGGGATGGAAAGACTGGGGAGAACAGCCCGCTGTACAAAAGCCCTGTTGACACTGGT
GACCTCATCTACTTCAACGTTGATTACGCTATGAACTACTGGAAGAGCAACGGTGCCCC
TGCTGAGAAGCTCCTTGTTGGATTCCCCACCTACGGACATAACTTCGTCCTGCAGAACC
CATCTGACACTGCTGTTGGGGCACCAGCATCGGGACCTGGGCCTGCTGGACCGTACACA
AGGCAGGCTGGGTTCTTGGCTTACTACGAGATCTGCACGTTCCTGGACTCTGGAGCCAC
CCAGGCTTGGGATGCCCCCCAGGACGTGCCCTATGCCTACAAGGGCAACGAATGGGTTG
GCTACGACAACATCAAGAGCTTCAACATCAAGATTGACTGGCTGAAGAAGAACAACTTT
GGAGGTGCTATGGTTTGGTCTCTTGATATGGATGACTTCACCGGCACATTCTGCAAGCA
GGGCAAATATCCCCTGATCACCACCCTGAAGAACGCTCTTGGTCAGCAGGGCAGCAGCT
GTGTGCCCCCAGCTCAGCCCAATCCTCCCATCACTTCAGCTCCTAACACTGGAAGTGGA
AGTGGCAGCGGCAGCGGGAGCTCGGGTGGCAATACTGGTGGCACTGGTGGGAGCGGATT
CTGTGCTGGCAAGGCCAACGGCATCTATGCAGATCCGACCAACAAGAGCAAGTTCTACA
ACTGCAACAACGGTGAAACCTACGAGCAGAGCTGCCAGGCCGGGCTGGTCTTTGATCCC
AGCTGCTCCTGCTGCAACTGGGCA
>Aythya_fuligula3
ATGGCCAAGCTCACTCTGCTTACCGGTCTGGCCCTCCTGCTGAACGCCCAGATAGGCAC
TGCCTATGTGCTGTCATGCTACTTCACCAACTGGGCCCAGTACAGGCCCGGCCTGGGAA
AGTATATGCCAGACAACATCGACCCATGCCTGTGTGACCATCTGATCTATGCCTTCGCT

GGGATGTCCAACAATGAGATCACAACCTATGAATGGAACGATGAGACCCTCTACAAATC
CTTCAATGGCCTGAAAAACCAGAATGAAAATCTGAAGACCCTCCTGGCAATTGGAGGAT
GGAATTTTGGGACAGCCAAGTTCTCCGCAATGGTTTCCACTCCGGAGAACCGCCAGACT
TTCATCAATTCCGTCATCAAATTCCTGCGCCAGTACCAGTTTGATGGGCTGGACCTCGA
CTGGGAGTACCCTGGCTCCAGGGGCAGCCCGGCTCAGGACAAGGGTCTCTTCACCGTCC
TGGTGAAGGAAATGCTGGCTGCCTTCGAGCAGGAAGCCAAGCAGGTTGGCAAGCCCCGT
CTGATGATCACGGCTGCTGTTGCTGCAGGACTTTCCAACATCCAGTCTGGCTACGAGAT
CGCTGAGCTCGGCAAGTACTTGGACTACTTCCATGTGATGACTTATGACTTCCACGGTT
CCTGGGATGGAAAGACTGGGGAGAACAGCCCGCTGTACAAAAGCCCTGTTGACACTGGT
GACCTCATCTACTTCAACGTTGATTACGCTATGAACTACTGGAAGAGCAACGGTGCCCC
TGCTGAGAAGCTCCTTGTTGGATTCCCCACCTACGGACATAACTTCGTCCTGCAGAACC
CATCTGACACTGCTGTTGGGGCACCAGCATCGGGACCTGGGCCTGCTGGACCGTACACA
AGGCAGGCTGGGTTCTTGGCTTACTACGAGATCTGCACGTTCCTGGACTCTGGAGCCAC
CCAGGCTTGGGATGCCCCCCAGGACGTGCCCTATGCCTACAAGGGCAACGAATGGGTTG
GCTACGACAACATCAAGAGCTTCAACATCAAGATTGACTGGCTGAAGAAGAACAACTTT
GGAGGTGCTATGGTTTGGTCTCTTGATATGGATGACTTCACCGGCACATTCTGCAAGCA
GGGCAAATATCCCCTGATCACCACCCTGAAGAACGCTCTTGGTCAGCAGGGCAGCAGCT
GTGTGCCCCCAGCTCAGCCCAATCCTCCCATCACTTCAGCTCCTAACACTGGAAGTGGA
AGTGGCAGCGGCAGCGGGAGCTCGGGTGGCAATACTGGTGGCACTGGTGGGAGCGGATT
CTGTGCTGGCAAGGCCAACGGCATCTATGCAGATCCGACCAACAAGAGCAAGTTCTACA
ACTGCAACAACGGTGAAACCTACGAGCAGAGCTGCCAGGCCGGGCTGGTCTTTGATCCC
AGCTGCTCCTGCTGCAACTGGGCA
>Aythya_fuligula_4
ATGGCCAAGCTCACTCTGCTTACCGGTCTGGCCCTCCTGCTGAACGCCCAGATAGGCAC
TGCCTATGTGCTGTCATGCTACTTCACCAACTGGGCCCAGTACAGGCCCGGCCTGGGAA
AGTATATGCCAGACAACATCGACCCATGCCTGTGTGACCATCTGATCTATGCCTTCGCT
GGGATGTCCAACAATGAGATCACAACCTATGAATGGAACGATGAGACCCTCTACAAATC
CTTCAATGGCCTGAAAAACCAGAATGAAAATCTGAAGACCCTCCTGGCAATTGGAGGAT
GGAATTTTGGGACAGCCAAGTTCTCCGCAATGGTTTCCACTCCGGAGAACCGCCAGACT
TTCATCAATTCCGTCATCAAATTCCTGCGCCAGTACCAGTTTGATGGGCTGGACCTCGA
CTGGGAGTACCCTGGCTCCAGGGGCAGCCCGGCTCAGGACAAGGGTCTCTTCACCGTCC
TGGTGAAGGAAATGCTGGCTGCCTTCGAGCAGGAAGCCAAGCAGGTTGGCAAGCCCCGT
CTGATGATCACGGCTGCTGTTGCTGCAGGACTTTCCAACATCCAGTCTGGCTACGAGAT
CGCTGAGCTCAGCAAGTACTTGGACTACTTCCATGTGATGACTTATGACTTCCACGGTT
CCTGGGATGGAAAGACTGGGGAGAACAGCCCGCTGTACAAAAGCCCTGTTGACACTGGT
GACCTCATCTACTTCAACGTTGATTACGCTATGAACTACTGGAAGAGCAACGGTGCCCC
TGCTGAGAAGCTCCTTGTTGGATTCCCCACCTACGGACATAACTTCGTCCTGCAGAACC
CATCTGACACTGCTGTTGGGGCACCAGCATCGGGACCTGGGCCTGCTGGACCGTACACA
AGGCAGGCTGGGTTCTTGGCTTACTACGAGATCTGCACGTTCCTGGACTCTGGAGCCAC
CCAGGCTTGGGATGCCCCCCAGGACGTGCCCTATGCCTACAAGGGCAACGAATGGGTTG
GCTACGACAACATCAAGAGCTTCAACATCAAGATTGACTGGCTGAAGAAGAACAACTTT
GGAGGTGCTATGGTTTGGTCTCTTGATATGGATGACTTCACCGGCACATTCTGCAAGCA
GGGCAAATATCCCCTGATCACCACCCTGAAGAACGCTCTTGGTCAGCAGGGCAGCAGCT
GTGTGCCCCCAGCTCAGCCCAATCCTCCCATCACTTCAGCTCCTAACACTGGAAGTGGA
AGTGGCAGCGGCAGCGGGAGCTCGGGTGGCAATACTGGTGGCACTGGTGGGAGCGGATT
CTGTGCTGGCAAGGCCAACGGCATCTATGCAGATCCGACCAACAAGAGCAAGTTCTACA
ACTGCAACAACGGTGAAACCTACGAGCAGAGCTGCCAGGCCGGGCTGGTCTTTGATCCC
AGCTGCTCCTGCTGCAACTGGGCA
>Aythya_fuligula_5
ATGGCCAAGCTCACTCTGCTTACCGGTCTGGCCCTCCTGCTGAACGCCCAGATAGGCAC
TGCCTATGTGCTGTCATGCTACTTCACCAACTGGGCCCAGTACAGGCCCGGCCTGGGAA
AGTATATGCCAGACAACATCGACCCATGCCTGTGTGACCATCTGATCTATGCCTTCGCT
GGGATGTCCAACAATGAGATCACAACCTATGAATGGAACGATGAGACCCTCTACAAATC
CTTCAATGGCCTGAAAAACCAGAATGAAAATCTGAAGACCCTCCTGGCAATTGGAGGAT

GGAATTTTGGGACAGCCAAGTTCTCCACAATGGTTTCCACTCCGGAGAACCGCCAGACT
TTCATCAATTCCGTCATCAAATTCCTGCGCCAGTACCAGTTTGATGGGCTGGACCTCGA
CTGGGAGTACCCTGGCTCCAGGGGCAGCCCGGCTCAGGACAAGGGTCTCTTCACCGTCC
TGGTGAAGGAAATGCTGGCTGCCTTCGAGCAGGAAGCCAAGCAGGTTGGCAAGCCCCGT
CTGATGATCACGGCTGCTGTTGCTGCAGGACTTTCCAACATCCAGTCTGGCTACGAGAT
CGCTGAGCTCGGCAAGTACTTGGACTACTTCCATGTGATGACTTATGACTTCCACGGTT
CCTGGGATGGAAAGACTGGGGAGAACAGCCCGCTGTACAAAAGCCCTGTTGACACTGGT
GACCTCATCTACTTCAACGTTGATTACGCTATGAACTACTGGAAGAGCAACGGTGCCCC
AGCTGAGAAGCTCCTTGTTGGATTCCCCACCTACGGACATAACTTCGTCCTGCAGAACC
CATCTGACACTGCTGTTGGGGCACCAGCATCGGGACCTGGGCCTGCTGGACCGTACACA
AGGCAGGCTGGGTTCTTGGCTTACTACGAGATCTGCACGTTCCTGGACTCTGGAGCCAC
CCAGGCTTGGGATGCCCCCCAGGACGTGCCCTATGCCTACAAGGGCAACGAATGGGTTG
GCTACGACAACATCAAGAGCTTCAACATCAAGATTGACTGGCTGAAGAAGAACAACTTT
GGAGGTGCTATGGTTTGGTCTCTTGATATGGATGACTTCACTGGCACATTCTGCAAGCA
GGGCAAATATCCCCTGATCACCACCCTGAAGAACGCTCTTGGTCAGCAGGGCAGCAGCT
GTGTGCCCCCAGCTCAGCCCAATCCTCCCATCACTTCAGCTCCTAACACTGGAAGTGGA
AGTGGCAGCGGCAGCGGGAGCTCGGGTGGCAATACTGGTGGCACTGGTGGGAGCGGATT
CTGTGCTGGCAAGGCCAACGGCATCTATGCAGATCCGACCAACAAGAGCAAGTTCTACA
ACTGCAACAACGGTGAAACCTACGAGCAGAGCTGCCAGGCCGGGCTGGTCTTTGATCCC
AGCTGCTCCTGCTGCAACTGGGCA
>Aythya_fuligula_6
ATGGCCAAGCTCACTCTGCTTACCGGTCTGGCCCTCCTGCTGAACGCCCAGATAGGCAC
TGCCTATGTGCTGTCATGCTACTTCACCAACTGGGCCCAGTACAGGCCCGGCCTGGGAA
AGTATATGCCAGACAACATCGACCCATGCCTGTGTGACCATCTGATCTATGCCTTCGCT
GGGATGTCCAACAATGAGATCACAACCTATGAATGGAACGATGAGACCCTCTACAAATC
CTTCAATGGCCTGAAAAACCAGAATGAAAATCTGAAGACCCTCCTGGCAATTGGAGGAT
GGAATTTTGGGACAGCCAAGTTCTCCACAATGGTTTCCACTCCGGAGAACCGCCAGACT
TTCATCAATTCCGTCATCAAATTCCTGCGCCAGTACCAGTTTGATGGGCTGGACCTCGA
CTGGGAGTACCCTGGCTCCAGGGGCAGCCCGGCTCAGGACAAGGGTCTCTTCACCGTCC
TGGTGAAGGAAATGCTGGCTGCCTTCGAGCAGGAAGCCAAGCAGGTTGGCAAGCCCCGT
CTGATGATCACGGCTGCTGTTGCTGCAGGACTTTCCAACATCCAGTCTGGCTACGAGAT
CGCTGAGCTCGGCAAGTACTTGGACTACTTCCATGTGATGACTTATGACTTCCACGGTT
CCTGGGATGGAAAGACTGGGGAGAACAGCCCGCTGTACAAAAGCCCTGTTGACACTGGT
GACCTCATCTACTTCAACGTTGATTACGCTATGAACTACTGGAAGAGCAACGGTGCCCC
AGCTGAGAAGCTCCTTGTTGGATTCCCCACCTACGGACATAACTTCGTCCTGCAGAACC
CATCTGACACTGCTGTTGGGGCACCAGCATCGGGACCTGGGCCTGCTGGACCGTACACA
AGGCAGGCTGGGTTCTTGGCTTACTACGAGATCTGCACGTTCCTGGACTCTGGAGCCAC
CCAGGCTTGGGATGCCCCCCAGGACGTGCCCTATGCCTACAAGGGCAACGAATGGGTTG
GCTACGACAACATCAAGAGCTTCAACATCAAGATTGACTGGCTGAAGAAGAACAACTTT
GGAGGTGCTATGGTTTGGTCTCTTGATATGGATGACTTCACTGGCACATTCTGCAAGCA
GGGCAAATATCCCCTGATCACCACCCTGAAGAACGCTCTTGGTCAGCAGGGCAGCAGCT
GCGTGCCCCCAGCTCAGCCCAATCCTCCCATCACTTCAGCTCCTAACACTGGAAGTGGA
AGTGGCAGCGGCAGCGGGAGCTCGGGTGGCAATACTGGTGGCACTGGTGGGAGCGGATT
CTGTGCTGGCAAGGCCAACGGCATCTATGCAGATCCGACCAACAAGAGCAAGTTCTACA
ACTGCAACAACGGTGAAACCTACGAGCAGAGCTGCCAGGCCGGGCTGGTCTTTGATCCC
AGCTGCTCCTGCTGCAACTGGGCA
>Aythya_fuligula_7
ATGGCCAAGCTCACTCTGCTTACCGGTCTGGCCCTCCTGCTGAACGCCCAGATAGGCAC
TGCCTATGTGCTGTCATGCTACTTCACCAACTGGGCCCAGTACAGGCCCGGCCTGGGAA
AGTATATGCCAGACAACATCGACCCATGCCTGTGTGACCATCTGATCTATGCCTTCGCT
GGGATGTCCAACAATGAGATCACAACCTATGAATGGAACGATGAGACCCTCTACAAATC
CTTCAATGGCCTGAAAAACCAGAATGAAAATCTGAAGACCCTCCTGGCAATTGGAGGAT
GGAATTTTGGGACAGCCAAGTTCTCCACAATGGTTTCCACTCCGCAGAACCGCCAGACC
TTCATCAATTCTGTCATCAAATTCCTGCGCCAGTACCAGTTTGATGGGCTGGACCTCGA

CTGGGAGTACCCTGGCTCCAGGGGCAGCCCGGCTCAGGACAAGGGTCTCTTCACCGTCC
TGGTGAAGGAAATGCTGGCTGCCTTCAAGCAGGAAGCCAAGCAGGTTGGCAAGCCCCGT
CTGATGATCACGGCTGCTGTTGCTGCAGGACTTTCCAACATCCAGTCTGGCTACGAGAT
CGCTGAGCTCGGCAAGTACTTGGACTACTTCCATGTGATGACTTATGACTTCCACGGTT
CCTGGGATGGAAAGACTGGGGAGAACAGCCCGCTGTACAAAAGCCCTGTTGACACTGGT
GACCTCATCTACTTCAACGTTGATTACGCTATGAACTACTGGAAGAGCAACGGTGCCCC
TGCTGAGAAGCTCCTTGTTGGATTCCCCACCTACGGACATAACTTCGTCCTGCAGAACC
CATCTGACACTGCTGTTGGGGCACCAGCATCGGGACCTGGGCCTGCTGGACCGTACACA
AGGCAGGCTGGGTTCTTGGCTTACTACGAGATCTGCACGTTCCTGGACTCTGGAGCCAC
CCAGGCTTGGGATGCCCCCCAGGACGTGCCCTATGCCTACAAGGGCAACGAATGGGTTG
GCTACGACAACATCAAGAGCTTCAACATCAAGATTGACTGGCTGAAGAAGAACAACTTT
GGAGGTGCTATGGTTTGGTCTCTTGATATGGATGACTTCACCGGCACATTCTGCAAGCA
GGGCAAATATCCCCTGATCACCACCCTGAAGAACGCTCTTGGTCAGCAGGGCAGCAGCT
GTGTGCCCCCAGCTCAGCCCAATCCTCCCATCACTTCAGCTCCTAACACTGGAAGTGGA
AGTGGCAGCGGCAGCGGGAGCTCGGGTGGCAATACTGGTGGCACTGGTGGGAGCGGATT
CTGTGCTGGCAAGGCCAACGGCATCTATGCAGATCCGACCAACAAGAGCAAGTTCTACA
ACTGCAACAACGGTGAAACCTACGAGCAGAGCTGCCAGGCCGGGCTGGTCTTTGATCCC
AGCTGCTCCTGCTGCAACTGGGCA
>Aythya_fuligula_8
ATGGCCAAGCTCACTCTGCTTACCGGTCTGGCCCTCCTGCTGAACGCCCAGATAGGCAC
TGCCTATGTGCTGTCATGCTACTTCACCAACTGGGCCCAGTACAGGCCTGGCCTGGGAA
GTTTCATGCCAGACAACATCGACCCATGCCTGTGCACTCACCTGCTCTACGCCTTCGCT
GGGATGTCCAACAACGAGATCACGACCATTGAATGGAACGATGAGACCCTCTACAAATC
CTTCAATGGCCTGAAAAACCAGAATAAAAACCTGAAGACTCTTCTTTCTATTGGAGGAT
GGAATTTTGGAACAGACAAGTTCTCCACAATGGTTTCCACTCCGCAGAACCGCCAGACC
TTCATCAAGTCCGTCATCAAATTCCTGCGCCAGTACCAGTTTGATGGGCTGGACATTGA
CTGGGAGTACCCTGGCTCCAGGGGCAGCCCGGCTCAGGACAAGGGTCTCTTCACTGTCC
TGGTGAAGGAAATGCTGGCCGCCTTTGAGCAGGAAGCCAAGCAGGTTGGCAAGCCCCGT
CTGATGATCACGGCTGCTGTTGCTGCAGGACTTTCCAACATCCAGTCTGGCTACGAGAT
CGCTGAGCTCGGCAAGTACCTGGACTACTTCAACGTGATGACTTATGATTTCCATGGCT
CCTGGGATGCACAAACTGGGGAGAACAGCCCTCTCTACAAAGGCCCAGGTGACACTGAT
GGCAATATCTATTTCAACGTTGATTACGCTATGAACTACTGGAAGAGCAATGGTGCCCC
AGCTGAGAAGTTAGTTGTTGGATTCCCCACCTATGGAAACACCTTCAGACTGCAAAACC
CATCCAACAATGGTCTTGGTGCACCAGCATCGGGACCTGGACCTGCTGGACCTTACACA
CAGGAGGCTGGGACACTGGCTTACTATGAGATCTGCACTCTGCTGAATTCTGGAGCCAC
CCAAGTTTGGGATGCCCCCCAGGACGTGCCCTATGCCTACAAGGGCAACGAATGGGTTG
GCTACGACAACATCAAGAGCTTCAACATCAAGGCTGACTGGCTGAAGAAGAACAACTAT
GCAGGTGCTATGGTTTGGGCCATTGATCTGGATGACTTCACCGGCACATTCTGCAAGCA
GGGCAAATATCCCCTGATCACCACCCTGAAGAACGCTCTTGGCCTGCAAAGCAGCAGCT
GTGTGCCCCCAGCTCAACCCAATCCTCTAGTGACTGCAGCTCCCAGTACTACAAGTGGA
AGTGGAAGTGGAAGTGGGAGTGGGAGTGGAAGTGGAAGTGGCAGCGGGAGCGGGAGCTC
GGGTGGCAATACTGGTGGCACTGGTGGGAGCGGATTCTGTGCTGGCAAGGCCAACGGCA
TCTATGCAGATCCGACCAACAAGAGCAAGTTCTACAACTGCAACAACGGTGAAACCTAC
GAGCAGAGCTGCCAGGCCGGGCTGGTCTTTGATCCCAGCTGCTCCTGCTGCAACTGGGC
A

>Nothoprocta_perdicaria_1
ATGGCCAAGCTCATTGTGCTCACCGGTCTGGTGCTCCTGCTCAACGCCCACATAGGCTC
GGCCTATGTGCTGTCATGTTACTTCACCAACTGGGCCCAGTACCGGCCTGGCCTGGGAG
CCTTTATGCCTGACAATATTGACCCCTGCCTGTGCACTCACCTTCTATATGCCTTTGCT
GGAATGAACAACAACGAGATCGCGACCATTGAATGGAATGATGTGACCCTTTACAAGTC
CTTCAACGGCTTGAAGAACCAAAATAAAGATTTGAAGACCCTCCTCTCCATCGGAGGAT
GGAATTTTGGAACAGACAAGTTCTCGGCAATGGTTTCCACTCCTGAGAACCGCCAGACC
TTCATCAAGTCYGTCATCAAATTCCTGCGCCAGTATGAATTTGATGGGCTTGACATTGA

CTGGGAATACCCAGGGTCTAGGGGCAGCCCAGCTCAGGACAAGAGTCTCTTCACTGTTC
TTGTAAAGGAAATGCTGGCAGCCTTTGAGCAGGAAGCTCAACAGGTCAACAAGCCCAGG
CTCCTCATCACTGCTGCCGTTGCTGCTGGACTTTCCAACATCCAGTCTGGCTACCAGAT
TGCTGAGCTTGGAAAATACCTGGACTACTTCCATGTGATGACTTACGACTTCCATGGTT
CATGGGATGGACACACCGGAGAGAACAGCCCTCTGTACAAAGGCCCAGCTGACACCGGC
AGCTACATCTACTTCAATGTCGATTATACTATGAACTATTGGAAGAGCAACGGTGCCCC
AGCCGAGAAGCTCCTTGTTGGATTCCCAACCTATGGACACAACTTCAACCTCCAAAACC
CCTCTGACACTGCTCTCGGCGCACCGACATCAGGACCTGGGCCTGCTGGACCTTACACG
CAAGAGGCTGGCACGCTGGCTTACTATGAGATCTGCACTCTGCTGAAATCTGGAGCCAC
CCAAGTTTGGGATGCCCCCCAGGACGTGCCCTATGCCTACAAGGGCAGCACATGGGTTG
GCTATGACAACATCAAGAGCTTCAACATYAAGGCTGACTGGCTGACCAAAAACAAATAT
GGAGGTGCTATGGTTTGGGCCATTGATCTGGATGATTTCACAGGCTCTTTCTGCAAGGA
GGGCAAATACCCCCTGATCACCACTCTGAAGAACGCTCTTGGTCTGCAAAGCAGCAGTG
AG
>Gavia_stellata
ATGGCCAAGCTCACTTTGCTTACCGGTCTGGCCCTCCTGCTGAACGCCCAGCTAGGCAC
TGCCTATGTGCTGTCATGCTACTTCACCAGCTGGGCCCAGTATAGGCCTGGCCTGGGAA
AATTCATGCCTGACAACATCGACCCGTGCCTGTGCAACCATCTGATCTACGCCTTTGCT
GGGATGTCCAACAATGAGATCACAACTTACGAATGGAATGACGAGACCCTTTACAAATC
CTTCAACGGCTTGAAGAACCAGAACAGAAATCTGAAGACTCTCCTGGCCATTGGAGGAT
GGAATTTTGGGACAGCCAAATTCACCACAATGGTTTCCACTCCCGCGAACCGCCAGACC
TTCATCAAGTCCGTCATCAAATTCCTGCGCCAGTATCAATTTGATGGGCTGGACTTTGA
CTGGGAATACCCTGGGGCCAGGGGCAGCCCACCCCAGGACAAGGCTCTCTTTACTGTCC
TGGTTAAGGAAATGCTGGCAGCCTTTGAGCAGGAAGCCAAACAGGTCAACAAGCCCCGT
CTCATGATCACCGCCGCTGTCGCTGCAGGACTTTCCACCATTCAGGCTGGCTACGAGAT
TCCTGAGCTTGGAAAGTACTTGGACTACATCCATGTGATGACTTACGACTTCTATAGCT
CCTGGGATGGACGCACTGGGGAGAACAGCCCTCTGTACAGTGGAGGTAACAGCTACCTC
AGTGTTGATTATGCTATGAACTATTGGAAGAACAATGGTGCCCCAGCTGAGAAGCTTAT
TGTTGGATTCCCAACCTATGGACATAACTTCAACCTCCAAAACCCATCTGACACTGGTA
TTGGGGCACCAACATCAGGACCTGGGCCAGCTGGACCTTACACAAAGCAGGCTGGGTTT
TTGGCTTACTACGAGATCTGCACATTCCTGGACTCTGGAGCCACCCAGGCTTGGGATGC
CCCCCAGGACGTGCCCTATGCCTACAAGGGCAACGAATGGGTTGGCTATGACAACGTCA
AGAGCTTCAACATCAAGGTTGACTGGCTGAAGAAGAACAATTTTGGAGGTGCTATGGTT
TGGACCATTGATCTGGATGATTTCACTGGCACTTTCTGCAAGGAAGGCAAATATCCCCT
GATCACTGCCCTGAAGAACGGTCTTGGTCTGCAAAACAGCGACTGCGTGCCTCCGCCTG
AGCCCAATCCTCCAGTCACTGAAGCTCCTAACCAAGTAGGTGGAAGTGGGAGCGGGGGC
TCAGGTGGCAACACTGGTGGCTCTGGTGGGAGCGGTTTCTGTGCTGGCAAGGCCAACGG
CATCTATGCGGATCCAACCAACAAGAGCAACTTCTACAACTGTGTTAATGGTGAAACCT
TCATGCAGAGCTGCCAGGATGGCCTCATCTTTGATACCAGCTGCTCCTGCTGCAACTGG
CCA
>Nestor_notabilis_2
ATGGCCAAGCTCACTTTGCTCACCAGTCTGGCCCTGCTGCTGAATGCCCAGGCAGGCAC
TGCCTATGTGCTGTCATGTTACTTCACCAACTGGGCCCAGTATAGGCCTGGCTTGGGAA
GATTCCTGCCTGACAACATCGACCCATTCCTGTGTGACCATCTGATCTATGCCTTTGCT
GGGATGTCCAACAATGAGATCACAACTTCCGAATGGAACGACAACACCCTTTACCAGTC
CTTCAATGGCTTGAAGAACCGGAACACAAATCTGAAGACCCTCTTGGCAATTGGAGGAT
GGAATTTTGGAACAGCCCAGTTCTCCACAATGGTTTCCACACCTGAGAACCGCCAGACG
TTCATCAATTCCGTCATCAAATTCCTGCGCCAGAATCAATTTGATGGGCTGGACATTGA
TTGGGAATACCCTGGCTCCAGAGGCAGCCCAGCCCAGGACAAGACTCTCTTTACCGTCC
TGGTTAAGGAAATGGTGGCAGCCTTTGAGCAGGAAGCCCAACAGGTCAACAAGCCCCGT
CTCATGGTCACCGCAGCCGTTGCCGCAGGACTTTCCAACATCCAGGCTGGCTACCAGAT
TCCTGAGCTTGGGAAGTACTTGGACTACATCCATGTGATGACTTATGATTTCTATACCT
CTGGGGATGGACAAACTGGGGAGAACAGCCCTCTCTACGGTGATGGTAACACCTACTTC
AGTGTTGAATATGCTATGAACTATTGGAAGAACAGTGGTGCTCCAGCTGAGAAGCTCCT

TGTTGGATTCCCAACCTATGGACATACCTTCAACCTCCAAAGCCCATCCAACACTGCTA
TTGGGGCACCAACATCAGGACCTGGGCCAGCTGGACCTTACACAAGGCAGGCTGGGTTT
TTGGCTTACTACGAGATCTGCACATTCCTGAAGTCTGGAGCCACCCAGGCTTGGGATGC
CCCCCAGGATGTGCCCTATGCCTACAGCGGCAGCGAGTGGGTTGGCTATGACAACATCA
AGAGCTTCAACATCAAGGTTGACTGGTTGAAGAAGAACAATTTTGGAGGCGCTATGGTT
TGGGCCCTTGATCTGGATGACTTCACTGGCTCTTTCTGCCAGCAGGGCAAATACCCCCT
GATCACCACCCTGAAGAACAGCCTTGGTCTGCAGAATGGCAACTCTGCGTCTGCATCTC
AGCCCAGTGCTCCTGCCGCTGAGCCCCCCTCTAACGCAGGTGGAAGTGAGAGCGGGGGC
TCTAGTGTTAGTAACTTCTGTGCTGGCAAGGCCAATGGCCTCTATGCAGATCCAACCAG
CCAGAGAAGCTTCTACAACTGCCTGAATGGAGAAACCTATGTGCAGAGCTGCCAGAGCA
ATCTCGTCTTTGATACCAGCTGCTCCTGCTGCAACTGGCCA
>Tyto_alba
ATGAGAGGAGCATATAAAAGTGGAAGTTGGTCTGCACCACGTCAGTCCTGGTCAAAGAT
GGCCAAGCTCGCTTTGCTTACCGCTCTGGTCCTCCTGCTGAACGCCCAGATAGGCACTG
CCTATGTGCTGTCATGTTACTTCACCAACTGGGCCCAATATAGGCCTGGCCTGGGAAAA
TTCATGCCTGACAACATTGACCCGTGCCTGTGTGACCATCTGATCTACGCCTTTGCTGG
CATGTCCAACAACGAGATCACAACTTACGAATGGAACGATGAGACCCTTTACAAATCCT
TCAACGGCTTGAAGAACAAAAATGGAAAACTGAAGACCCTCCTGGCGATTGGAGGATGG
AATTTTGGGACAGCCAAGTTCTCCACAATGGTCTCCACTCCCGAGAACCGACAGACCTT
CATCAAGTCCGTCATCAAATTCCTGCGCCAGTACCAGTTTGATGGGCTGGACATTGACT
GGGAATACCCTGGGTCCAGGGGCAGCCCATCCCAGGACAAGACTCTTTTTACTGTCCTG
GTTAAGGAAATGCTGGCAGCCTTTGAGCAGGAAGCCAAACAGGTCAACAAGCCCCGTCT
CATGCTCACCGCGGCCGTTGCTGCAGGACTTTCTACCATTCAGGCTGGCTACCAGATTC
CTGAGCTTGGAAAGTACCTGGACTACTTCCATGTGATGACCTACGACTTCCACGGCTCC
TGGGACAGAAACACTGGGGAGAACAGCCCTCTGTACAAAGGCCCAGCTGACACTGGTGA
CCTCATCTACTTCAATGTTGATTATGCCATGAACTATTGGAAGAGCAATGGTGCCCCTG
CTGAGAAGCTCCTTGTTGGATTCCCAACCTATGGACATAACTTCAACCTCCAAAACCCA
GCTGACACAGCTGTTGGGGCACCAGCAACAGGACCTGGGCCAGCTGGACCTTACACAAG
GCAGGCTGGATTCTTGGCTTACTACGAGATCTGCACATTCCTGGACTCTGGAGCCACCC
AGGCTTGGGATGCTCCCCAGGACGTGCCCTACGCTTACAAAGGCAACGAGTGGGTTGGC
TATGACAACATCAAGAGCTTCAACATCAAGGTTGACTGGCTGAAGAAGAACAACTTTGG
AGGTGCTATGGTTTGGGCCCTTGATATGGATGACTTCACTGGCACTTTCTGCAAGGAAG
GCAAATATCCCCTGATCACCACCCTGAAGAACGGTCTTGGTCTGCAAAATGGTGACTGT
GTGCCTCCAGCTCAGCCCAATCCTCCAGTCACTGAGGCTCCTAACCACGGAGGTGGAAG
TGGGGGCTCAGGTGGCGATACTGGTGGCTCTGGTGGGAGTGGTTTCTGTGCTGGCAAGG
CCAACGGAGTCTATGCAGATCCAACCAACAAGAGGAACTTCTACAACTGCATCAATGGT
GAAACCTTTGTGCAGAGCTGCCAGGAAGGCCTCATCTTTGATACCAGCTGCTCCTGCTG
CAACTGGCCA
>Motacilla_alba_1
ATGGCCAAGCTCACTCTGCTCACCGGCCTGGCGCTGCTGCTGAACGCCCAGCTCGGCAC
TGCCTATGTGCTGACCTGTTACTTCACCAACTGGGCCCAGTACCGGCCCGGTCTGGGTA
AGTTCACCCCCGAAAATGTCGACCCTTGCCTGTGCAACCACCTGATCTACGCCTTCGCC
GGCATGAACAACAACGAGATCACCACCTACGAGTGGAACGACGAGACCCTCTACAAGTC
CTTCAATGGCCTCAAGAACCAGAACAAAGATCTGAAGACCCTGCTGGCCATTGGAGGAT
GGAATTTTGGCACACAGAAGTTCACCACCATGGTCTCCACACCCCAGAACCGCCAGACC
TTCATCAAGTCTGTCATCAAATTCCTGCGCCAGTATCAGTTTGACGGGCTGGACCTGGA
CTGGGAATACCCCGGCTCCAGGGGCAGCCCTGCCCAGGACAAGTCTCTCTTCACCGTCC
TGGTTAAGGAAATGCTGGAAGCCTTCGAGCAGGAAGCCAAACAGACCAACCAGCCCCGG
CTCATGGTCACCGCCGCTGTCGCTGCCGGACTTTCCACCATCCAGGCTGGCTACGAGAT
CGCTGAGATTGGCAAGTACCTGGACTACATCCACGTCATGACCTACGACTTCCACGGCT
CCTGGGAGAGGAACACCGGCGAGAACAGCCCCCTGTACGCCGGCCCTGCTGACAGCGGC
GACTACAAATACTTCAACGTTGAATACGCCATGAATTATTGGAAGAGCAATGGTGCCCC
AGCTGAGAAGCTCCTTGTGGGATTCCCGACCTATGGAAAGAGCTTCACCCTGCAGAACC
CATCTGACACCTCTGTTGGAGCTCCAGCATCCGGCCCTGGCCCCGCTGGGCCCTACACC

AGGGAGGCTGGAACTCTGGCTTACTACGAGATCTGCTCTCTCCTGAGCTCCGGAGCCAC
CCAGGCTTGGGATGAACCCCAGGATGTCCCCTACGCCTACAAGGGCAGCGAATGGGTCG
GCTATGACAACGTCAAGAGCTTCGGCCTCAAGGTGGACTGGCTGAAGAAGAACAGCTTT
GGAGGAGCCATGGTGTGGGCCCTGGACATGGATGACTTCACTGGGGATTTCTGCAAGGA
AGGCAAATACCCGCTGATCTCCAGCCTGAAGAAGGGCCTGGGGCTGCAGAGCGGCGACT
GCGTTCCCCCCGCTGAGCCTCTGCCTCCCATTACCGAGGCTCCCACCACCCCCGGCGGC
GGCTCCGGCGGCTCCGGCGGCTCTGGCGGCTCCGGATTCTGCGCCGGGAAACCCAACGG
GATCTACGCGGACCCCAACAACAAGAGGAACTTCTACAGCTGCCTGAACGGCCAGACCT
TCGTGCAGAGCTGCGAGGAGGGGCTGGTCTTCGACCCCACCTGCTCCTGCTGCAACTGG
CCCCAG
>Motacilla_alba_2
ATGGCCAAGCTCACTCTGCTCACCGGCCTGGCGCTGCTGCTGAACGCCCAGCTCGGCAC
TGCCTATGTGCTGACCTGTTACTTCACCAACTGGGCCCAGTACCGGCCCGGCGAGGGCA
GATACACCCCTGAGAACATCGACCCCAACCTGTGCAGCCACCTGATCTACGCCTTCGCC
GGCATGAACAACAACGAGATCACCACCTACGAGTGGAACGACGAGACCCTCTACAAGTC
CTTCAATGGCCTCAAGAACCAGAACAGGAACCTGAAGACCCTGCTGGCCATTGGAGGAT
GGAATTTCGGCACACAGAAGTTCACCACCATGGTCTCCACACCCCAGAACCGCCAGACC
TTCATCAACTCCGTGGTCAGGTTCCTGCGCCAGTACGGATTCGACGGGCTGGACCTGGA
CTGGGAATACCCCGGCTCCAGGGGCAGCCCTGCCCAGGACAAGTCTCTCTTCACCGTCC
TGGTTAAGGAACTGGTGGCAGCCTTCGAGCAGGAAGCCAGACAGAGCAACAGGCCCCGG
CTCATGGTCACCGCCGCTGTGGCCGGAGGACTTTCCACCATCCAGGCTGGCTACGAGAT
CGCTGAGCTGGGCAAGTACCTGGACTACATCCACGTCATGACCTACGACTTCCACGGGC
CCTGGGACGGCTCCACGGGCGAGAACAGCCCCCTGTTCAGCAGCGGCAGCACCCTCAGT
GTTGAATACGCCATGAACTACTGGAAGAACAACGGCGCCCCGGCTCAGAAGCTGCTGGT
GGGATTCCCGACCTATGGAAAGAGCTTCACCCTGCAGAGCCCATCCAACACCGGCATCG
GAGCCCCCAGCTCCGGCCCTGGCCCCGCAGGACCCTACACCAGGGAGGCCGGGCTCCTG
GCTTACTATGAGATCTGCACCTTCCTGAGCTCCGGAGCCACCCAGGCTTGGGATGCCCC
CGAGGATGTCCCCTACGCCTACAAGGGCAGCGAATGGGTCGGCTATGACAACGTCAAGA
GCTTCGGCCTCAAGGTGGACTGGCTGAAGAAGAACAACTTTGGAGGAGCCATGGTGTGG
ACCATCGACCTGGATGACTTCACCGGCAACTTCTGCCACGAGGGCAAATACCCGCTGAT
CTCCACCCTGAAGAGGGGCCTGGGGCTG
>Onychostruthus_taczanowskii_1
ATGAACAACAACGAGATCACCACCTACGAGTGGAATGACGAGACCCTGTACAAGTCCTT
CAATGGCCTCAAGAACCAGAACAAAGATCTGAAGACCCTGCTGGCCATTGGAGGATGGA
ATTTCGGCACAGCCAAGTTCTCCACCATGGTCTCCACACCTGAGAACCGCCAGACCTTC
ATCAAGTCCGTCATCAAATTCCTGCGCCAGTATCAGTTTGACGGGCTGGACCTGGACTG
GGAATACCCCGGCTCCAGGGGCAGCCCTGCCCAGGACAAGTCTCTCTTCACCGTCCTGG
TTAAGGAGCTGCTGGCAGCCTTCGAGCAGGAAGCCAAACAGACCAACAGGCCCCGGCTC
ATGGTCACCGCCGCTGTCGCTGCCGGACTTTCCACCATCCAGTCTGGCTACGAGATCGC
CGAGATTGGCAAGTACCTGGATTACATCCACGTCATGACCTACGACTTCCACGGCTCCT
GGGAGAGGAACACCGGCGAGAACAGCCCCCTGTTCGCCGGCCCTGCCGACAGCGGCGAC
TACAAATACTTCAACGTTGAATACGCCATGAATTATTGGAAGAGCAACGGCGCCCCAGC
TGAGAAGCTCCTGGTGGGATTCCCAACCTACGGAAAGAGCTTCACCCTGCAGAGCCCAT
CTGACACCTCTGTTGGAGCTCCAGCATCCGGCCCTGGCCCCGCTGGGCCCTACACCAGG
GAGGCTGGAACTCTGGCTTACTACGAGATCTGCTCTCTGCTGAGCTCTGGAGCCACCCA
GGCTTGGGATGCCCCCGAGGATGTCCCCTACGCCTACAAGGGCAGCGAGTGGGTCGGCT
ACGACAACGTCAAGAGCTTTGGCCTCAAGGTGGACTGGCTGAAGAAGAACAACTTTGGA
GGAGCCATGGTGTGGGCACTGGACATGGATGACTTCACTGGGGATTTCTGCAAGGAAGG
CAAATACCCCCTGATCTCCAGCCTGAAGAAGGGCCTGGGGCTGCAGAGCGGAGACTGCG
TTCCCCCCTCTGAGCCCCTGCCTCCCATCACTGAGGCTCCCTCCACCACCAGCGGCGGC
TCCGGCGGCTCCGGCGGCTCTGGATTCTGTGCCGGGAAACCCAACGGGATCTACGCAGA
CCCCAACAACAAGAGGAACTTCTACAACTGCCTGAACGGCCAGACCTTTGTGCAGAGCT
GCGAGCAGGGGCTGGTCTTCGACCCCGTCTGCTCCTGCTGCAACTGGCCCCAG
>Onychostruthus_taczanowskii_2

ATGGCCAAGCTCACTCTGCTCGCCGGCCTGGCCCTGCTGCTGAACGCCCAGCTCGTTTG
GGAAAGAGCTGAGCAGGAAGAGGAGGGGAGAAATGTGAGAGGAAAATTAACTGGCTTCA
TCTTCTGGTACGAGATCGCCGAGCTGGGCAAGTACCTGGATTACATCCACGTCATGACC
TACGACTTCCACGGACCCTGGGACGGCTCCACGGGCGAGAACAGCCCCCTGTTCGACAG
CGGCAGCACCTTCAGCGTTGAATACGCCATGAACTACTGGAAGAACAACGGCGCCCCAG
CTCAGAAGCTGCTGGTGGGATTCCCAACCTATGGAAAGAGCTTCACCCTGCAGAGCCCA
TCCAACACCGCCATCGGCGCCCCCAGCTCCGGGCCCGGCCCCGCAGGACCCTACACCAG
GGAGGCCGGGCTCCTGGCTTACTACGAGATCTGCACCTTCCTGAGCTCTGGAGCCACCC
AGGCTTGGGATGCCCCCGAGGATGTCCCCTACGCCTACAAGGGCAGCGAGTGGGTCGGC
TACGGCAACGTCAAGAGCTTTGGCCTCAAGGTGGACTGGCTGAAGAAGAACAACTTTGG
AGGAGCCATGGTGTGGACCATCGACCTGGATGACTTCACTGGCACCTTCTGCCACCAAG
GCAAATACCCCCTGATCTCCAGCCTGAAGAGGGGCCTGGGGCTG
>Corvus_cornix_2
ATGGGAGACGGCAGCGCCGCATGGAATCCTCGTGGAAAAACTCTTCTGAGTCCTCCCGA
AACATCCCTGGGGACGAGGGGTGAGCAGAGCACGCTGGAGAAGCGGCAGCGGGGGGGAA
TGGCTGCAGGATTTCGTAAGCAGGGCACTGCCTATGTGCTGACCTGTTACTTCACCAAC
TGGGCCCAGTACAGGCCTGGCCTGGGCAAATACACCCCTGAGAACATCGACCCCAACCT
GTGCAACCACCTGATCTACGCCTTCGCCGGCATGAACAACAACGAGATCACCACCTACG
AGTGGAACGATGAGACCCTGTACAAGTCCTTCAACGGCCTCAAGAACCAGAACAGGAAC
CTGAAGACCCTTCTGGCCATTGGAGGATGGAATTTCGGCACAGAGAAGTTCACCACCAT
GGTTTCCACACCCCAGAACCGCCAGACCTTCATCAACTCCGTGGTCAGGTTCCTGCGCC
AGTACGGATTCGACGGGCTGGACCTGGACTGGGAATACCCCGGCTCCAGGGGCAGCCCA
GCCCAGGACAAGGGTCTCTTCACCGTCCTGGTTAAGGAACTGCTGGCAGCCTTCGAGCA
GGAAGCCAAACAGACCAACCGGCCCCGGCTCATGGTCACCGCCGCTGTGGCCGGTGGAC
TTTCCACCATCCAAGCTGGCTATGAGATTGCTGAGTTGGGCAAGTACCTGGATTACATC
CACGTGATGACCTACGACTTCCACGGGCCCTGGGACGGCTCCACGGGCGAGAACAGCCC
CCTGTTCAGTAGCAGCACCCTCAGTGTTGAATACGCCATGAACTACTGGAAGAACAACG
GCGCCCCAGCTCAGAAGCTCCTGGTGGGATTCCCAACCTACGGGAAAACCTTCACCCTG
CAGAACCCATCCAACACCGGCATCGAGGCCCCAACCTCCGGCCCTGGTCCTGCGGACC
CTACACCAAGGAGGCCGGGCTCTTGGCTTACTACGAGATCTGCACCTTCCTGAACTCCG
GAGCCACCCAGGCTTGGGATGAACCCCAGGACGTGCCCTACGCCTACAAGGGCAGCGAA
TGGATCGGCTACGACAACGTCAAGAGCTTCGGCCTCAAGGTGGATTGGCTGAAGAAGAA
CAACTTTGGCGGGGCCATGGTCTGGACCATCGACCTGGATGACTTCACCGGCACCTTCT
GCCACGAGGGCAAATACCCCCTGATCTCCACGCTGAAGAAGGGCCTGGGGCTG
>Corvus_cornix_1
ATGGCCAAGCTCACTCTGCTCACCGGCCTGGCCCTGCTGCTGAACGCCCACGTCGGCAC
TGCCTATGTGCTGACCTGTTACTTCACCAACTGGGCCCAGTACAGGCCTGGCCTGGGCA
AATACACCCCCGAAAATGTCGACCCTTGCCTGTGCAACCACCTGATCTACGCCTTCGCC
GGCATGAACAACAACGAGATCACCACCTACGAGTGGAACGATGAGACCCTGTACAAGTC
CTTCAACGGCCTCAAGAACCAGAACAAAGACCTGAAGACCCTGCTGGCCATTGGAGGAT
GGAATTTTGGCACAGCCAAGTTCACCACCATGGTTTCCACACCCCAGAACCGCCAGACC
TTCATCAACTCTGTCATCAAATTCCTGCGCCAGTATCAGTTTGATGGGCTGGACCTGGA
CTGGGAATACCCCGGCTCCAGGGGCAGCCCAGCCCAGGACAAGGGTCTCTTCACCGTCC
TGGTTAAGGAACTGCTGGCAGCCTTCGAGCAGGAAGCCAAACAGACCAACCGGCCCCGG
CTCATGGTCACCGCCGCTGTGGCTGCCGGACTTTCCACCATCCAGGCTGGCTATGAGAT
TGCTGAGATTGGCAAGTACCTGGATTACATCCACGTGATGACCTACGACTTCCACGGCT
CCTGGGAGAGGAACACTGGTGAGAACAGCCCCCTGTTTGCCGGCCCTGCTGACACCGGG
GACTACAAATACTTCAACGTTGAATACGCCATGAATTATTGGAAGAGCAACGGTGCCCC
AGCTGAGAAGCTCCTGGTGGGATTCCCAACCTATGGAAAGAGTTTCACCCTGCAGAACC
CATCTGACACCTCCGTTGGAGCTCCAGCGTCCGGCCCTGGCCCAGCTGGGCCCTACACC
AGGGAGGCCGGAACCCTGGCTTACTACGAGATCTGCACCTTCCTGAGCTCTGGAGCCAC
CCAGGCTTGGGATGAACCCCAGGACGTGCCCTACACCTACAAGGGCAGCGAATGGATCG
GCTACGACAACGTCAAGAGCTTCGGCCTCAAGGTGGATTGGCTGAAGAAGAACAACTTT
GGCGGGGCCATGGTCTGGGCCCTGGACATGGATGACTTCACCGGCACCTTCTGCCACGA

GGGCAAATACCCCCTGATCTCCACGCTGAAGAAGGGCTTGGGGCTGGAGAGCAGCGACT
GTGTTCCCCCCGCTGAGCCCCTGCCTCCCATCACCGAGGCTCCCACCACCACCACCACC
AGCGGCGGCTCCGGCGGCTCCGGCGGCAGCGGCTTCTGCGCCGGGAAACCCAATGGGAT
CTACGCAGACCCCGACAACAAGAGGAACTTCTACAGCTGCGTGAACGGGCAGACCTTCG
TGCAGAGCTGTGAGCAGGGGCTGGTCTTCGACCCCGTCTGCTCCTGCTGCAACTGGCCC
CAG
>Merops_nubicus_2
ATGGTTTCCACTCCCCAGAACCGCCAGACCTTCATCAAGTCTGTCATCAAGTTCCTGCG
CCAGTATCAGTTTGATGGGCTCGACTTTGACTGGGAATACCCTGGGTCCAGGGGCAGCC
CAGCCCAGGACAAGGGTCTCTTCACTGTCCTCATTAAGGAAATGTTGGCAGCCTTTGAG
GGGGAAGCCAAACAGGTCAACAAGCCCCGTCTCATGGTCACTGCTGCTGTTGCTGCAGG
ACTTTCCACCATTCAGGCTGGCTACGAGAACAATGTTCTCTGCAGGTACCTGGACTACA
TCCACGTGATGACTTATGACTTCCACGGCTCCTGGGACAGAACCACTGGGGAGAACAGC
CCCCTGTACAAAGGTCCAGCTGACACTGGTGACCTTGTCTACTTCAACGTCGATTATGC
TATGAACTACTGGAAGAGCAATGGTGCCCCTGCTGAGAAGCTGCTTGTTGGATTCCCAA
CCTATGGACACAACTTCATCCTCCAAAACCCATCTGACACTGCTGTTGGGGCACCTGCA
ACAGGACCTGGGCCAGCTGGACCTTACACAAGGCAGGCTGGATTCTTGGCTTACTATGA
GATCTGCACATTCCTGGACTCTGGAGCCACTGAGGCTTGGGATGAACCCCAGGATGTGC
CCTATGCCTACAAGGGCAATGAATGGGTTGGCTATGACAACATCAAGAGCTTCAACATC
AAGGTTGACTGGCTGAAGAAGAACAACTTTGGAGGTGCTATGGTTTGGTCCCTTGACAT
GGATGACTTCACTGGAACTTTCTGCAAGGAAGGCAAATATCCCCTGATCTCCACTCTGA
AGAAAGGCCTTGGTCTGGATAGTGGTGACTGCGTGCCACCAGCTCAGCCCAATCCTCCA
GTCACTGAAGCTCCAGGCCAGGGAGGTGGCAGTGGGGGCTCTGGTGGCAATCCTGGTGG
CTCTGGTGGGAGTGGCTTCTGTGCTGACAAGGCCAAC
>Merops_nubicus_1
ATGGTTTCCACTCCCCAGAACCGCCAGACCTTCATCAAGTCTGTCATCAAGTTCCTGCG
CCAGTATCAGTTTGATGGGCTCGACTTTGACTGGGAATACCCTGGGTCCAGGGGCAGCC
CAGCCCAGGACAAGGGTCTCTTCACTGTCCTCGTTAAGGAAATGGTGGCAGCTTTTGAG
CAGGAAGCCAGACAGGTCAACAAGCCCCGTCTCATGGTCACTGCTGCTGTTGCTGCAGG
ACTTTCCACCATTCAGGCTGGCTACGAGATTGCTGAGCTTGGAAAGTACCTGGACTACA
TCCACGTGATGACTTACGACTTCTACAGCTCCTCAGACGGTCGCACTGGGGAGAACAGC
CCCCTGCACAATACTGCTAACAGTGCCTTCAGTGTTGAATATGCTATGAACTATTGGAA
GAACAACGGTGCCCCAGCTCAGAAGCTCCTTGTTGGATTCCCAACCTATGGACGTACCT
TCAACCTCCAAAACCCATCTAACACTGCTGTTGGGGCACCCACATCAGGACCTGGGCCA
GCTGGAACTTACACTCAGGAAGCTGGGCTTTTGGCTTACTATGAGATCTGCTCATTCCT
GGCCTCTGGAGCCACTGAGGCTTGGGATGGTCCTGAGGACGTGCCTTATGCCTACAAGG
GCAGCGAATGGGTTGGCTATGACAACACCAAGAGCTTCAGCATCAAGGTTGACTGGCTG
AAGAAGAACAACTTTGGAGGTGCTATGGTTTGGACCATTGATCTGGATGACTTCACTGG
CACTTTCTGCAAGCAAGGCAAATATCCCCTGATCTCCACTCTGAAGAAAGGTCTTGGCC
TG
>Athene_cunicularia_2
ATGAGAGGAGCGTATAAAAGCGGGAAACGGTCTGCACCAGCTCAGTCCTGGTCAAAGAT
GGCCAAGCTCACTTTGCTTACCGGTCTGGTCCTCCTGCTGAACGCCCAGATAGGCTCTG
CCTATGTGCTCTCATGTTACTTCACCAACTGGGCCCAATATAGGCCTGGTGTGGCAAAA
TACATGCCTGACAACATTGACCCATGCCTGTGCACTCACCTGCTATACGCCTTTGCTGG
GATGTCCAACAATAAGATCACAACTTATGAGTGGAACGACGAGGAACTTTACAAATCCT
TCAACGGCTTGAAGAACCAAAACAAAAATCTGAAGACTCTGCTCTCTATTGGAGGATGG
AATTTTGGGACAGACAAGTTTTCCACTATGGTTTCCACTCCCGAGAACCGCCAGACCTT
CATCAAGTCTGTCATCAAATTCCTGCGCCAGTACCAGTTTGATGGGCTGGACATTGACT
GGGAGTACCCCGGCTCCAGGGGCAGCCCAGCCCAGGACAAGGCTCTCTTTACCGTCCTG
GTGAAGGAAATGCTGGCAGCCTTTGAGCAGGAAGCCAAACAGGTCAACAAGCCCCGTCT
CCTGGTCACTGCAGCCGTTGCTGCAGGAGTGTCCACCATCGAGGCTGGCTACCAGATTG
CTGAGCTTGGAAAGTACCTGGACTACTTCCACGTGATGACTTACGACTTCCACGGCTCC
TGGGACAGTACCACTGGGGAGAACAGCCCTCTGTACAAAGGCCCAACTGACACAGGTGA

CTACATCTACTTCAATGTGGATTATGCTATGAATTATTGGAAGAGCAATGGTGCCCCAG
CTGAGAAACTCGTTGTTGGATTCCCAACATATGGAAATACCTTCACACTGCGAAACCCA
TCTGAGAATGGTCTTGGAGCACCAGCATCAGGAGCTGGGCCAGCTGGACCTTACACACA
GGAGGCTGGGGAACTGGCTTACTTTGAGATCTGCACTCTGTTGAATTCTGGAGCCACCG
AGGTTTGGGATGCCCCCCAGGACGTGCCCTACGCTTACAAAGGCAGCGAATGGGTTGGC
TATGACAACATCAAGAGCTTCAACATCAAGGTTGACTGGCTGAAGAAGAACAACTTTGG
AGGTGCCATGGTTTGGTCCCTGGACATGGACGACTTCACTGGCACTTTCTGCAAGGAAG
GCAAATATCCCCTGATCACCACCCTGAAGAAGGGTCTTGGTCTGCAAAACAATGACTGC
GTGCCGCCAGCTCATCCCAATCCTCCTGTCACTGGAGCTCCCTCTACTACAGGTGGAAG
TGGAAGTGGGAGTGGGAGTGGGGGCTCTGGTGTTAGTGACTTCTGTGCTGGCAAGGCCA
ACGGCATCTATGCAGACCCAACCAACAAGAGAAACTTCTACAACTGTGTCAATGGTGAA
ACCTTCATGGAGAGCTGTGAAGAAGGCCTCGTCTTTGATGCCAGCTGCTCCTGCTGCAA
CTGGCCA
>Fulmarus_glacialis_2
ATGGCCAAGCTCACTTTGCTTACCGGTCTGGCCCTCCTGCTGAACGCCCAGATAGGCAC
TGCCTATGTGCTGTCATGTTACTTCACCAACTGGTCCCAATATAGGCCTGGCCTGGGAA
AATTCATGCCTGACAACATCAACCCATTCCTGTGCGACCATCTGATCTATGCCTTTGCT
GGGATGTCCAACAATGAGATCACAACTTACGAATGGAACGACGAGACCCTTTACAAGTC
CTTCAACGGCTTGAAGAACCAGAATGGAAAACTGAAGACCCTCCTGGCGATTGGAGGAT
GGAATTTCGGGACAGCCAAGTTCTCCACAATGGTTTCGACTCCCGAGAACCGCCAGACC
TTCATCAACTCCGTCATCAAATTCCTGCGCCAGTATCAATTTGATGGGCTGGACCTTGA
CTGGGAATACCCTGGGTCCAGGGGCAGCCCACCCCAGGACAAGACTCTCTTTACCGTCC
TGGTTAAGGAAATGCTGGCAGCCTTTGAGCAGGAAGCCAAACAGGTCAACAAGCCCCGT
CTCATGCTCACCGCTGCTGTTGCTGCAGGACTTTCCACCATTCAGGCTGGCTACCAGAT
TGCTGAGCTTGGAGAGTACCTGGACTATTTCCATGTGATGACTTACGACTTCCATGGCC
CCTGGGATGGATACACTGGGGAGAACAGCCCTCTGTACAAAGGCCCAGCAGACACTGGT
GACCTCATCTACTTCAATGTCAATTATGCTATGAACTATTGGAAGAGCAATGGTGCTCC
AGCTGAGAAGCTCCTTGTTGGATTCCCAACCTATGGACATAACTTCAACCTCCAAAACC
CATCTGACACTGCTGTTGGGGCACCAACATCAGGACCTGGGCCAGCGGGACCTTACACA
AGGCAGGCTGGATTCTTGGCTTACTACGAGATCTGCACATTCCTGGCCTCTGGAGCCAC
CCAGGCTTGGGATGCCCCCCAGGATGTGCCCTATGCCTACAAGGGCAATGAATGGGTTG
GCTACGACAACATCAAGAGCTTCAACATCAAGGTGGACTGGCTGAAGAAGAACAATTTT
GGAGGTGCTATGGTTTGGGCCCTTCCTATGGATGATTTCACTGGCACTTTCTGCAAGGA
AGGCAAATATCCCCTGATTACCGCCCTGAAGAACAGTCTTGGTCTGCAGAATGGTGATG
GTGTACCTCCAGCTCAGCCCAATCCTCCAGTCACTAGCGGAAGTGGAAGTGGAAGTGGT
GGAAGTGGAAGTGGAAGTGGAAGTGGAAGTGGAAGTGGGAGCGGAGGCTCTGGTGTTAG
TGACTTCTGTGCTGGCAAGACCAATGGCATCTACGCAGATCCAACCAACAAGAGCATGT
TCTACAACTGCATTAATGGCAAAACCTTTGTGCAGAGCTGCAATAACGGTCTCGTCTTT
GATACCAGCTGCTCCTGCTGCAACTGGCCA
>Chaetura_pelagica_1
ATGGCCAAGCTCACTCTGCTGACCGGCACTGCCAATGTGCTGACATGTTACTTCACCAA
CTGGGCCCAGTACAGGCCTGGCCTGGGAAAGTTCATGCCTGAAAACATTGACCCCTTCC
TGTGCAACCACCTGATCTACGCCTTTGCTAACATGAACAACAACGAGATCACAACTTAC
GAATGGAATGATGAGACCCTCTACAAATCCTTCAATGGCCTGAAGAACCAGAACAGAAA
TCTGAAGACCCTCCTGGCAATTGGAGGATGGAATTTCGGGACAGCCAAGTTCTCCACAA
TGGTTTCCACTCCTCAGAACCGCCGGACCTTCATCAATTCTGTCATCAGATTCCTGCGC
AAGCATAATTTTGATGGTCTGGACCTTGACTGGGAATGCCCCGGGTCCAGGGGCAGCCC
ACCCCAGGCCAAGACTCTCTTCACTGTCCTGGTTAAGGAAATGGTGGCAGCCTTTGAGC
AGGAAGCCAGACAGTCCAACAGGCCCCGTCTCATGGTCACTGCTGCTGTTGCTGCAGGA
CTTTCCACCATTCAGGCTGGCTACGAGATTGCTGAGCTTGGAAAGTACTTGGACTACAT
CCATGTGATGACTTACGACTTCCATGGCTCCGGGGATGGACGCACTGGGGAGAACAGCC
CTCTGCACAGTGGTGGTAACCCCCAGCTCAGTGTTGAATATGCTATGAAATATTGGAGG
GACAATGGTGCTCCAGCTAAGAAGCTCCTTGTGGGATTCCCAACCTATGGACGTACCTT
CACCCTCCAAAACCCATCCAACACTGCTGTGGGGGCACCAGCATCAGGACCTGGGCCAG

CTGGAACTTACACACAGGAGGCTGGACTGTTGGCTTACTATGAGATCTGCTCATTCCTG
AACTCTGGAGCCACCCAGGCTTGGGATGCCCCTGAGGATGTCCCCTATGCCTACAAGGG
CAGTGAATGGGTTGGCTATGACAACGTCAAGAGCTTCAACATCAAGGTTGATTGGCTGA
AGAAGAACAATTTTGGAGGTGCCATGGTTTGGACCGTAGATTTGGATGACTTCACTGGC
ACTTTCTGCAATCAG
>Chaetura_pelagica_2
ATGCCTGAAAACATTGACCCCTTCCTGTGCAACCACCTGATCTACGCCTTTGCTAACAT
GAACAACAACGAGATCACAACTTACGAATGGAACGACGAGACCCTCTACAAATCCTTCA
ATGGCCTGAAGAACCAGAATGGAAAACTGAAGACCCTCCTGGCAATTGGAGGATGGAAC
TTTGGGACAGCCAAGTTCTCCACAATGGTTTCCACTCCTCAGAACCGCCAGACCTTCAT
CAATTCTGTCATCAAATTCCTGCGCCAATATCAATTTGATGGTCTGGACCTTGACTGGG
AATACCCCGGGTCCAGAGGCAGCCCACCCCAGGACAAGACTCTCTTCACTGTCCTGGTT
AAGGAAATGGTGGCAGCCTTTGAGCAAGAAGCCCAACAGACCAACTGGCCCCGTCTCAT
GCTCACCGCTGCTGTTGGTGCAGGACGTCCCACCATTGATGCTGGCTACGAGATTGCTG
AGCTTGGAAAGTACTTGGACTACATCCATGTGATGACTTATGACTTCCATGGCTCCTGG
GATGGACGCACTGGGGAGAACAGCCCTCTGTATGAAGGCCCATCTGACACTGGTGACCT
TGTCTACTTCAATGTTGATTATGCCATGAACTATTGGAAGAATAATGGTGCCCCAGCTG
AGAAGCTCCTTGTTGGATTCCCAACCTATGGACACAACTTCATCCTCCAAAACCAATCC
AACACTGCTGTGGGGGCACCAACAACAGGACCTGGGCCAGCTGGACCTTACACTAGGCA
GGCTGGATTCTTGGCTTACTACGAGATCTGCTCATTCCTGGACTCTGGAGCCACCCAGG
CTTGGGATGACCCCCAGGATGTCCCCTATGCCTACAAGGGCAATGAATGGGTTGGCTAT
GACAACATCAAGAGCTTCAACATCAAGGTTGACTGGCTGAAGAAGAACAATTTTGGAGG
TGCCATGGTTTGGGCCCTTGATATGGATGACTTCACTGGCACTTTCTGCAAGGAAGGTA
GATATCCCCTGATCACTGCCCTGAAGGAGGGTCTTGGTCTGCAAAATGATGACTGTGTG
TCCCCAACTCAGCCCAACCCTCCCATCACTGAAGCTCCAGGTGGCACTCCTGGGGGCTC
TGGTGGAAGTGGCTTCTGTGCTGGGAAGGCTGATGGTCTCTATCCTGACCCAACCAACA
ACGCAAACTTCTACAACTGTGCTAATGGTGTAAGCGTTGTCCAGAGCTGCCAGGAGGGC
CTTGTCTTTGATACCAGCTGCTCCTGCTGCAACTGGCCA
>Caprimulgus_carolinensis_2
ATGGCCAAGCTCATTTTGCTTACTGGTAAGTCCTGCTTCATAAATTTCTCTGCAGGCAC
TGCCTATGTGCTGTCATGTTACTTCACCAACTGGGGCCAGTACAGGCCTGGTGTGGGAA
AATACATGCCTGACAATATTGACCCATGCCTGTGTGACCATCTGATCTACGCCTTCGCT
GGGATGAACAACAATGAGATCACGACTTACGAATGGAACGATGTGACCCTTTACAAATC
CTTCAACGGCCTGAAGAACCAGAATAAAAATCTGAAGACTCTGCTTTCTATTGGAGGAT
GGAATTTTGGGACAGCCAAGTTCTCCACAATGGTTTCCACTCCTGAGAACCGCCAGACC
TTCATCAATTCCGTCATCAAATTCCTGCGCCAGTATGAGTTTGATGGGCTGGACATTGA
CTGGGAATACCCTGGGTCCAGGGGCAGCCCACCCCAGGACAAGGCTCTCTTTACCGTCC
TGATTAAGGAAATGCTGGCAGCCTTTGAGCAGGAAGCCAAACAAGTCAACAAGCCCCGT
CTCTTGATCACCGCGGCTGTTGCTGCAGGAGTTTCCAACATTCAGTCTGGCTACCAGAT
TGCTGAGCTTGGAAAGTACTTGGACTACTTCCATGTGATGACTTACGACTTCCATGGCT
CCTGGGATGGACAAACTGGGGAGAACAGCCCTCTGTTCAAAGGCCCATCTGACACTGGT
GACAACATCTACTTCAACATCGACTACACCATGAATTATTGGAAGAGCAACGGTGCCCC
AGCTGAGAAACTGGTCATTGGATTCCCAACATACGGAAATACCTTCACACTGCAAAACC
CATCTAACAATGGTCTCGGTGCACCAACATCAGGACCTGGGCCAGCTGGACCTTACACA
CAGGAGGCTGGGGAACTGGCTTACTATGAGATCTGCACTCTCCTGAATTCTGGAGCCAC
CCAGGTTTGGGATGCCCCCCAGGATGTCCCCTATGCTTACAAAGGCAACGAATGGGTTG
GCTATGACAACATCAAGAGCTTCAACATCAAGGTTGACTGGCTGAAGAAGAACAACTTT
GGAGGTGCTATGGTTTGGACCATTGATATGGATGACTTCACTGGTACTTTCTGCAAGCA
GGGCAAATATCCCCTGATCACCACTCTGAAGAATGGTCTTGGTCTGCAGAACAGCGACT
GTGTGCCTCCAGCTCATCCCAATCCTCCAGTCACTGCAGCTCCCTCTACTACAGCTGGA
AGTAGTGAAAGTGGAAGTGGAAGCAGTGGAAGTAGTGGAAGGGGGAGGAGTGGGAGGGG
GAGGGGAAGTGGAAGTGGGAGCTCTGGCAGCAATCCCGGTGGCTCTGGTGGGAGTAACT
TCTGCGCTGGCAAGAGCAGTGGCCTCTATGCAGATCCAACCAACAAGAGCAACTTCTAC
AACTGTGTTAATGGAGAAACCTTCTTGGAGAGCTGCCAGAGCGGCCTCGTCTTTGACAC

```
CAGCTGCTCCTGCTGCAACTGGCCA
>Geospiza_fortis_1
ATGAACAACAACGAGATCACCACCTACGAGTGGAACGACGAGACCCTCTACAAGTCCTT
CAATGGCCTCAAGAACCAGAACAAAGATCTGAAGACCCTGCTGGCCATTGGAGGATGGA
ATTTCGGCACACAGAAGTTCTCCACCATGGTCTCCACTCCTGAGAACCGCCAGACCTTC
ATCAAGTCCGTCATCAAATTCCTGCGCCAGTATCAGTTTGATGGGCTGGACCTGGACTG
GGAATACCCCGGCTCCAGGGGCAGCCCTGCCCAGGACAAGTCTCTCTTCACCGTCCTGG
TTAAGGAAATGGTGGCAGCCTTCGAGCAGGAAGCCAAACAGACCAACAAGCACCAGCTC
ATGGCTGCTGTGGCTGGAGGACTCTCCACCATCCAGTCTGGCTATGAGATCGCTGAGCT
GGGCAAGTACCTGGATTACATCCACGTCATGACCTACGACTTCCACGGCTCCTGGGAGA
GGAACACCGGCGAGAACAGCCCCCTGTTCGCCGGCCCTGCTGACAACGGCGACTACAAA
TACTTCAACGTTGAATACGCCATGAATTATTGGAAGAGCAATGGTGCCCCAGCTGAGAA
GCTCCTTGTGGGATTCCCAACCTATGGAAAGAGCTTCACCCTGCAGAACCCATCTGACA
CCTCTGTTGGAGCTCCAGCATCTGGCCCTGGCCCTGCTGGGCCCTACACCAGGGAGGCT
GGAACTCTGGCTTACTACGAGATCTGCTCTCTCCTGAGCTCTGGAGCCACCCAGGCTTG
GGATGAGCCCCAGGATGTCCCCTACGCCTACAAGGGCAGCGAATGGGTCGGCTATGACA
ACATCAAGAGCTTTGGCCTCAAGGTGGACTGGCTGAAGAAGAACAACTTTGGAGGGGCC
ATGGTGTGGGCCTTGGACATGGATGACTTCACTGGGGATTTCTGCAAGGAAGGCAAATA
CCCGCTGATCTCCAGCCTGAAGAAGGGCCTGGGGCTGCAGAGCGGCGACTGCGTTCCCC
CCTCTGAGCCCCTTCCTCCCATCACTGAGGCTCCCACCACCACCAGCGGCGGCTCGGGC
GGCTCCGGTGGCTCTGGCGGTTCCGGTGGCTCTGGATTCTGTGCCGGGAAAGCCAACGG
GATCTACGCAGACCCCAACAACAAGAAGAACTTCTACAACTGCCTGAACGGCCAGACCT
TCGTGCAGAGCTGCATGGAGGGGCTGGTCTTCGACCCCGCCTGCTCCTGCTGCAACTGG
CCCCAG
>Geospiza_fortis_2
ATGGCCAAGCTCACTCTGCTCACCGGTCTGGCCCTGCTGCTGAACGCCCAGCTCGGCAC
TGCCTATGTGCTGACCTGCTACTTCACCAACTGGGCCCAGTACAGGCCTGGCCTGGGCA
AGTTCACCCCTGAAAATGTCGACCCCTGTCTGTGCAACCACCTGATCTACGCCTTCGCC
GGCATGAACAACAACGAGATCACCACCTACGAGTGGAACGACGAGACCCTCTACAAGTC
CTTCAATGGCCTCAAGAACCAGAACAGGAAGCTGAAGACCCTGCTGGCCATCGGAGGAT
GGAATTTCGGCACACAGAAGTTCTCCACCATGGTCTCCACACCCCAGAACCGCCAGACC
TTCATCAACTCCGTGGTCAGGTTCCTGCGCCAGTACGGATTCGATGGGCTGGACCTGGA
CTGGGAATACCCCGGCTCCAGGGGCAGCCCTGCCCAGGACAAGTCTCTCTTCACCGTCC
TGGTTAAGGAAATGGTGGCAGCCTTCGAGCAGGAAGCCAAACAGACCAACAGGCCCCAG
CTCATGGTCACTGCTGCTGTGGCTGGGGGGNNGGCTCCCCCCCCTGGCTATGAGATCGC
TGAGCTGGGCAAGTACCTGGATTACATCCACGTCATGACCTACGACTTCCACGGGCCCT
GGGATGGCTCCACGGGCGAGAACAGCCCCCTGTTCAGCAGCGGCAGCACCCTCAGTGTT
GAATACGCCATGAACTACTGGAAGGACAACGGCGCCCCAGCCCAGAAGCTGCTGGTGGG
ATTCCCAACCTACGGAAAGAGCTTCACCCTGCAGAACCCATCCAACACGGCCATCGGAG
CCCCCAGCTCCGGGCCTGGCCCTGCAGGGCCCTACACCAAGGAGGCCGGGCTCCTGGCT
TACTACGAGATCTGCACCTTCCTGAGCTCTGGAGCCACCCAGGTTTGGGATGCCCCTGA
GGATGTCCCTTACGCCTACAAGGCCAACGAATGGGTCGGCTACGACAACGAGAAGAGCT
TTGGCCTCAAGGTGGACTGGCTGAAGAAGAACAACTTTGGAGGGGCCATGGTGTGGACC
ATCGACCTGGATGACTTCACTGGCAACTTCTGCCACCAGGGCAAATACCCACTGATCTC
CAGCCTCAAGAGGGGCCTGGGGCTG
>Serinus_canaria
ATGGCCAAGCTCACTCTGCTGGCCGGTGTGGCCCTGCTGCTGAACGCCCAGCTCGGCTC
TGCCTATGTGCTGACCTGCTACTTCACCAACTGGGCCCAGTACAGGCCTGGCCTGGGCA
AGTTCACCCCCCAAAATATCGACCCTTGTCTGTGCAACCACCTGATCTACGCCTTCGCC
GGCATGAGCAACAACGAGATCACCACCATCGAGTGGAACGACGAGACCCTCTACCAGTC
CTTCAATGGCCTCAAGAACCAGAACAAAGATCTGAAGACCCTGCTGGCCATTGGAGGAT
GGAATTTCGGCACAGCCAAGTTCTCCACCATGGTCTCCACGCCCCAGAACCGCCAGACC
TTCATCACCTCCGTCATCAAATTCCTGCGCCAGTACGGATTCGACGGGCTGGACCTGGA
CTGGGAATACCCCGGCTCCAGGGGCAGCCCTGCCCAGGACAAGGCTCTCTTCACCGTCC
```

TGGTCAAGGAGCTGCTGGCAGCTTTCCAGCAGGAGGCCGAGCAGACCAACAGGCCCCGG
CTCATGGTCACGGCTGCTGTGGCTGCCGGGCTCTCCACCATCCAGTCTGGCTACGAGAT
CGCTGAGCTGGGCAAGTACCTGGATTACATCCACGTCATGACCTACGACTTCCACGGGC
CCTGGGAGGGCTCCACGGGCGAGAACAGCCCCCTGTTCAGCAGCAGCAGCCCCCTCAGT
GTTGAATACGCCATGAACTACTGGAAGGACAGCGGTGCCCCAGCCCAGAAGCTGCTGGT
GGGATTCCCGACCTATGGAAAGAGCTTCACCCTGCAGAGCCCGTCCAACACCGGTGTTG
GAGCCCCCAGCTCCGGGCCTGGCCCTGCAGGACCCTACACCAGGGAGGCCGGGCTCCTG
GCTTACTACGAGATCTGCTCTCTCCTGAGCTCCGGAGCCACCCAGGCCTGGGATGCCCC
CCAGGATGTCCCCTACACCTACAAGGGCAGCGAGTGGGTTGGCTACGACAACGAGAAGA
GCTTCGGCCTCAAGGTGGACTGGCTGAAGGAGAACAACTTTGGAGGGGCCATGGTGTGG
GCCCTGGACATGGATGACTTCACTGGCACCTTCTGCCACCAGGGCAAATACCCCCTGAT
CTCCAGCCTGAAGAAGGGCCTGGGGCTGCAGAGCGGCGGCTGCGCTCCCCCCGCTGAGC
CCCTTCCTCCCATCGCTGAGGCTCCCAACACCCCCGGCAGTGGCTCTGGTGGCTCCGGT
GGCTCCGGTGGCTCCGGTGGCTCCGGTTTCTGTGCTGGGAAACTCAACGGGATCTACGC
CGACCCCAGCAACAAGAGGAACTTCTACAGCTGCCTGAACGGGAGGACCTTCGTGCAGA
GCTGCCAGCAGGGGCTGGTTTTTGACGCCCTCTGCTCCTGCTGCAACTGGCCCCAG
>Manacus_vitellinus_2
ATGGTTTCCTCTGCTGAGAACCGCCAGACCTTCATCAATTCTGTCATCAAATTCCTGCG
CCAGTACCAGTTTGATGGGCTGGACCTGGACTGGGAATACCCTGGCTCCAGGGGCAGCC
CAGCCCAGGACAAGGGGCTCTTCACCGTCCTGGTTAAGGAATTGCTGGCAGCCTTTGAG
CAGGAAGCCAAACAGACCAACCAGCCCCGGCTCCTGGTCACCGCGGCTGTGGCTGCAGG
ACTTTCCACCATCCAGGCTGGCTACGAGATTGCTGAGCTGGGCAAGTACCTGGACTACA
TCCACGTGATGACCTACGACTTCCACGGCTCCTGGGAGAGAAACACTGGCGAGAACAGC
CCCCTGTTCACAGGCCCAGCTGACACTGGGGACTTCAAGTACTTCAACGTCGAATATGC
TATGAATTACTGGAAGGACAATGGTGCCCCAGCTGAGAAGCTCCTTGTTGGCTTCCCAA
CCTACGGAAAAAGCTTCACCCTGCAAAACCCATCTGACACCTCTGTTGGGGCTCCAGCA
TCAGGCCCTGGACCAGCTGGACCTTACACCAGGGAGGCTGGGACACTGGCTTACTATGA
GATCTGCTCTCTCCTGAGCTCTGGAGCCACCCAGGCTTGGGATGAACCCCAGGACGTGC
CCTATGCCTACAAGGAGAGCGAATGGGTTGGCTATGATAACACAAAGAGCTTCAGCATC
AAGGTCGACTGGCTGAAGAAGAATAACTTTGGAGGGGCCATGGTTTGGGCCCTTGACAT
GGATGATTTCACTGGCACTTTCTGCAATGAAGGCAAATACCCCCTGATCTCCACCCTGA
AGAAGGGCCTCGGTCTGCAGGACGGCAACTGTGTGCCTCCTGCTGAGCCCCTGCCTCCA
GTCACTGAGGCCCCCACTACCACCAGTGGAAGTGGGAGCAGTGGCTCTGGTGGGAGTGG
TGGCTCTGGTGGGAGCGGCTTCTGCGCCGGCAAAGCCAACGGCATCTACGCAGACCCAG
AGAACAGCCAGAACTTCTACAACTGCTTGAACGGCCAGACCTTCGTGCAGAGCTGCCAG
CAGGGCCTCGTCTTCGACCCCGTCTGCTCCTGCTGCAACTGGCCA
>Manacus_vitellinus_1
ATGGCCAAGCTCACTCTGCTCACCGGTCTGGCCCTGCTGCTCAACGCCCACCTCGGCAC
TGCCTACGTGCTGACATGTTACTTCACCAACTGGGCCCAGTACAGGCCTGGGGAGGGGA
AATACACCCCCGAGAACATCGACCCCAACCTGTGCAGCCACCTGATCTACGCCTTCGCC
GGGATGAACAACAACGAGATCACCACTTATGAATGGAACGACGAGACCCTCTACAAATC
CTTCAACGGCCTGAAGAACCAGAACAGAAACCTGAAGACCCTGCTGGCCATTGGAGGAT
GGAATTTCGGCACAGAAAAGTTCACTACAATGGTTTCCACCCCCCAGAACCGCCAGACT
TTCATCAACTCCGTTGTCAGATTCCTGCGCCAGTATGGATTCGATGGGCTGGACCTGGA
CTGGGAATACCCCGGCTCCAGGGGCAGCCCAGCCCAGGACAAGGGGCTCTTCACTGTCC
TGGTTAAGGAATTGCTGGCAGCCTTTGAGCAGGAAGCCAAACAGACCAACCAGCCCCGG
CTCCTGGTCACCGCGGCTGTGGCTGGAGGACTTTCCACCATCCAGGCTGGCTACGAGAT
TGCTGAGCTGGGCAAGTACCTGGACTACATCCACGTGATGACCTACGACTTCCACAGCC
CCTGGGACGGCTCCACTGGCGAGAACAGCCCCCTGTTCAGCAGTGGCAGCAGCTTCAGT
GTGGAATATGCTATGAACTACTGGAAGAACAATGGTGCCCCAGCTCAGAAGCTCCTGGT
TGGCTTCCCAACCTACGGAAAGACCTTCACCCTGCAAAACCCCTCCAACACTGGGATTG
GGGCCCCAACCTCAGGCCCTGGGCCAGTAGGACCCTACACCAGGGAGGCCGGGCTTCTG
GCTTACTACGAGATCTGCTCGTTCCTGAACACCGGAGCCACCCAGTCTTGGGATGCCCC
TGAGGACGTGCCCTATGCCTACAAGGGCAACGAGTGGATTGGCTACGACAACACGAAGA

GCTTCAGTGCAAAGGTCGACTGGCTGAAGCAGAACAACTTTGGAGGGGCCATGGTTTGG
ACCATCGACCTGGATGACTTCACTGGCACTTTCTGCCATGAAGGCAAATACCCCCTGAT
CTCCACCCTGAAGAAGGGCCTTGGTCTG
>Pseudopodoces_humilis_1
ATGTTTGTCAATCACTGTTGTTGCCCTATTGGCAGCAGAGAGGAGCAGGGTATAAAAGC
AGGAGAGAGGCTGAGCACATCAGTCTGGTCCAAGATGGCCAAGCTCACTCTGCTCACCG
GCCTGGCGCTGCTGCTGAACGCCCAGCTCGGCACTGCCTATGTGCTGACCTGTTACTTC
ACCAACTGGGCCCAGTACCGGCCTGGCCTGGGTAAGTACACCCCCGAAAATGTCGACCC
CTGCTTGTGCAACCACCTGATCTACGCCTTTGCCGGCATGAACAACAATGAGATCACCA
CCTACGAGTGGAACGACGAGACCCTGTACAAGTCCTTCAATGGCCTCAAGAACCAGAAC
AAAGATCTGAAGACCCTGCTGGCCATTGGAGGATGGAATTTCGGCACACAGAAGTTCAC
CACCATGGTCTCCACACCCCAGAACCGCCAGACCTTCATCAACTCCGTCATCAAATTCC
TGCGCCAGTACCAGTTTGACGGGCTGGACCTGGACTGGGAATACCCCGGCTCCAGGGGC
AGCCCCGCCCAGGACAAGGCTCTCTTCACCGTCCTGGTTAAGGAAATGCTGGAAGCCTT
CGAGAAGGAAGCCAAACAGACCAACCAGCCCCGGCTCATGGTCACCGCCGCTGTTGCTG
CTGGACTTTCCACCATCCAGGCTGGCTACGAGATTGCTGAGCTGGGCAAGTACCTGGAT
TACATCCATGTGATGACCTACGACTTCCACGGATCCTGGGAGAGGAACACTGGCGAGAA
CAGCCCCCTGTTCGCCGGCCCTGCTGACACCGGCGACTACAAATACTTCAACGTTGAAT
ACGCCATGAATTATTGGAAGAGCAACGGTGCCCCAGCTGAGAAGCTCCTGGTGGGATTC
CCAACCTACGGAAAGAGCTTCACCCTGCAGAACCCATCCGACACCTCCGTTGGAGCTCC
AGCATCTGGCCCTGGCCCCGCTGGGCCCTACACCAGGGAGGCCGGAACTCTGGCTTACT
ACGAGATCTGCAGTCTCCTGAGTTCTGGAGCCACCCAGGCTTGGGATGAACCCCAGGAT
GTTCCCTACGCCTACAAGGGCAGCGAATGGGTCGGCTACGACAACGTCAAGAGCTTCGG
CCTCAAGGTGGACTGGCTGAAGAAGAACAACTTTGGAGGTGCCATGGTGTGGGCCCTGG
ACATGGATGACTTCACTGGCACCTTCTGCCACGAGGGCAAATACCCCCTGATCTCCACC
CTGAAGAAGGGCCTGGGGCTGCAGAACGGCGACTGCGTTCCCCCCACTGAGCCTCCATC
CCCGAGGCTCCCACCACCTCCAGCAGGAGCAGCTCCGTCATGGAGACTCCCAAACTTCC
TACGGAGGCGTGGGAGGGATGAAACACCGGGAACA
>Pseudopodoces_humilis_2
ATGGCCAAGCTCACTCTGCTCACCGGCCTGGCGCTGCTGCTGAACGCCCAGCTCGGCAC
TGCCTATGTGCTGACCTGTTACTTCACCAACTGGGCCCAGTACCGGCCTGGCGAGGGGA
GATACACCCCTGAGAACATCGACCCCAACCTGTGCAGCCACCTGATCTACGCCTTTGCC
GGCATGAACAACAATGAGATCACCACCTACGAGTGGAACGACGAGACCCTGTACAAGTC
CTTCAATGGCCTCAAGAACCAGAACAGCAACCTGAAGACTCTTCTGGCCATTGGAGGAT
GGAATTTTGGCACACAGAAGTTCACCACCATGGTCTCCACACCCCAGAACCGCCAGACC
TTCATCAACTCCGTGGTCAGGTTCCTGCGCCAGTACGGATTTGACGGGCTGGACCTGGA
CTGGGAATACCCCGGCTCCAGGGGCAGCCCCGCCCAGGACAAGGCTCTCTTCACCGTCC
TGGTTAAGGAACTGCTGGCAGCCTTCGAGCAGGAGGCCAGGCAGACCAACCGGCCCCGG
CTCATGGTCACCGCCGCTGTGGCCGGAGGACTTTCCACCATCCAGGCTGGCTACGAGAT
TGCTGAGCTGGGCAAGTACCTGGATTACATCCACGTGATGACCTACGACTTCCACGGGC
CCTGGGATGGCTCCGCAGGCGAGAACAGCCCCCTGTTCAGCAGCGGCAGCACCCTCAGT
GTTGAATACGCCATGAACTACTGGAAGAACAACGGTGCCCCAGCTCAGAAGCTCCTGGT
GGGATTCCCAACCTATGGGAAAACCTTCACCCTGCAGAACCCATCCAACACCGCCATCG
GAGCCCCAACCTCTGGGCCTGGCCCGGCGGGACCCTACACCAGGGAGGCCGGGCTCTTG
GCTTACTACGAGATCTGCACCTTCCTGAGCTCTGGAGCCACCCAGGCTTGGGATGCCCC
TGAGGATGTTCCCTATGCCTACAAGGGCAGCGAATGGGTCGGCTACGACAACGTCAAGA
GCTTCGGCCTCAAGGTGGACTGGCTGAAGAAGAACAACTTTGGAGGTGCCATGGTGTGG
ACCATCGACCTGGATGATTTCACTGGCACCTTCTGCCACGAGGGCAAATACCCCCTGAT
CTCCACCCTGAAGAAAGGCCTGGGGCTG
>Taeniopygia_guttata_2
ATGGCCAAGCTCCCTCTGCTCACCGGCCTGGCCCTGCTGCTGAACGCCCACCTCGGCAC
TGCCCATGTGCTGAGCTGTTACTTCACCAACTGGGCCCAGTACCGGCCTGGCCCGGGCA
AGTTCACCCCTGAGAACATCGACCCCAACCTGTGCAACCACCTGATCTACGCCTTCGCC
GGCATGAACAACAACGAGATCTCCACGTCCGAGTGGAACGACGAGACCCTCTACAAGTC

CTTCAACGGCCTCAAGAAGCAGAACAGGAACCTGAAGACCCTGCTGGCGATTGGAGGAG
GGAGTTTCGGCACGCAGAAGTTCTCCACCATGGTCTCCACTCCCCAGAACCGCCAGATC
TTCATCAAGTCGGTCGTCAAATTCCTGCGGCAGCACGGATTTGATGGGCTGGACCTGGA
CTGGGAATACCCCGGCTCCAGGGGCAGCCCTGCCCAGGACAAGGCTCTCTTCACCCTCC
TGGTTAAGGAGCTGCGGGCAGCCTTCGAGCAGGAGGCCAAAGAGAGCGGCCAGCCCCGG
CTCATGGTCACCGCCGCTGTGGCCGCCGGACTTGCCACCATCCAGGCCGGCTACGAGAT
TGCTGAGCTGGGCAAGCACCTGGATTACATCCATGTCATGACCTACGACTTCCACGGCT
CCTGGGAGAGGAACACGGGTGAGAACAGCCCCCTGCGCAGCGGTGACGACAAATACTTC
AACGTCGAATACGCCATGAATTATTGGAAGAGCAATGGTGCCCCGGCTGAGAAGCTCCT
GGTGGGATTCCCAACCTATGGAAGAGCTTCACCCTGCAGAACCCATCCGACACCTCCG
TTGGGGCTCCAGCATCCGGCCCTGGCCCCGCCGGGCCCTACACCAGGGAGGCCGGAACT
CTGGCTTACCACGAGATCTGCACCTTCCTGAACTCCGGAGCCACCCAGGCTTGGCATGG
CCCCCAGGATGTCCCCTACGCCTACAAGGGCAACGAATGGGTCGGCTACGACAACGTCA
GGAGCTTCGGCCTCAAGGTGGATTGGCTGAAGAACAACAACTTTGGAGGAGCCATGGTG
TGGGATCTGGACATGGATGACTTCACTGGGGATTTCTGCAAGGAGGGCAAATACCCGCT
GATCTCCAGCCTGAAGAAGGGCCTGGGGCTG
>Taeniopygia_guttata_1
ATGGCCAAGCTCCCTCTGCTCACCGGCCTGGCCCTGCTGCTGAACGCCCACCTCGGCAC
TGCCCATGTGCTGAGCTGTTACTTCACCAACTGGGCCCAGTACCGGCCTGGCCCGGGCA
AGTTCACCCCCGAAAATGTCGATCCCTGCCTGTGCAACCACCTGATCTATGCCTTCGCC
GGCATGAGCAACAACGAGATCTCCACGTACGAGTGGAACGACGAGACCCTCTACAAGTC
CTTCAACGGCCTCAAGAAGCAGAACAAAGATCTGAAGACCCTGCTGGCCATTGGAGGAT
GGAATTTCGGCACGCAGAAGTTCTCCACCATGGTCTCCACGCCCCAGAACCGCCAGACC
TTCATCAAGTCCGTTGTCAAATTCCTGCGGCAGCACGGATTCGATGGGCTGGACCTGGA
CTGGGAATACCCCGGCTCCAGGGGCAGCCCTGCCCAGGACAAGGCTCTCTTCACCCTCC
TGGTTAAGGAGCTGCGGGCAGCCTTCGAGCAGGAGGCCAAAGAGAGCGGCCAGCCCCGG
CTCATGGTCACCGCCGCTGTGGCCGCCGGACTTGCCACCATCCAGGCCGGCTACGAGAT
CGCTGAGCTGGGCAAGCACCTGGATTACATCCACGTCATGACCTACGACTTCCACGGCT
CCTGGGAGAGGAACACGGGTGAGAACAGCCCCCTGCGCAGCGGTGACGACAAATACTTC
AACGTTGAATACGCCATGAATTATTGGAAGAGCAATGGTGCCCCGGCTGAGAAGCTCCT
GGTGGGATTCCCAACCTATGGAAGAGCTTCACCCTGCAGAGCCCATCCGACACCTCCG
TTGGGGCTCCAGCATCCGGCCCTGGCCCCGCCGGGCCCTACACCAGGGAGGCCGGAACT
CTGGCTTACCACGAGATCTGCACCTTCCTGAACTCCGGAGCCACCCAGGCTTGGCATGC
CCCCCAGGATGTCCCCTACGCCTACAAGGGCAGCGAATGGGTCGGCTACGACAACGTCA
GGAGCTTCGGCCTCAAGGTGGATTGGCTGAAGAAGAACAACTTTGGAGGGGCCATGGTG
TGGGCCCTGGACATGGATGACTTCACTGGGGATTTCTGCAAGGAGGGCAAATACCCGCT
GATCTCCAGCCTGAAGAAGGGCCTGGGGCTGCAGAGCGGCGACTGCGTTCCCCCCGCTG
AGCCCCAGCCTCCCACCAGCGGCGGCTCCAGTGGCTCCGGTGGCTCCGGTGGCTCCGGT
GGCTCCGGTGGCTCCGGTGGCTCCGGTGGCTCCGGTTTCTGTGCCGGGAAACCCAACGG
GATCTACGCAGACCCCAGCAACGGGAGGAACTTCTACAACTGCCTGAACGGCCAGACCT
TCGTGCAGAGCTGCCAACCTGGGCTGGTCTTCGACCCCGTCTGCTCCTGCTGCAACTGG
CCCCAG
>Parus_major_1
ATGGCCAAGCTCACTCTGCTCACCGGCCTGGCGCTGCTGCTGAACGCCCAGCTCGGCAC
TGCCTATGTGCTGACCTGTTACTTCACCAACTGGGCCCAGTACCGGCCTGGCCTGGGTA
AGTACACCCCCGAAAATGTCGACCCCTGCTTGTGCAACCACCTGATCTATGCCTTTGCT
GGCATGAACAACAACGAGATCACCACCTACGAGTGGAACGACGAGACCCTGTACAAGTC
CTTCAATGGCCTCAAGAACCAGAACAAAGATCTGAAGACCCTGCTGGCCATTGGAGGAT
GGAATTTCGGCACACAGAAGTTCACCACCATGGTCTCCACACCCCAGAACCGCCAGACC
TTCATCAACTCCGTCATCAAATTCCTGCGCCAGTACCAGTTTGATGGTCTGGACCTGGA
CTGGGAATACCCTGGCTCCAGGGGCAGCCCTGCCCAGGACAAGGGTCTCTTCACTGTCC
TGGTTAAGGAAATGCTGGAAGCCTTCGAGAAGGAAGCCAAACAGACCAACCAGCCCCGG
CTCATGGTCACCGCCGCTGTTGCTGCTGGACTTTCCACCATCCAGGCTGGATACGAGAT
TGCTGAGATTGGCAAGTACCTGGATTACATCCACGTGATGACCTACGACTTCCACGGAT

CCTGGGAGAGGAACACTGGCGAGAACAGCCCCCTGTTTGCTGGCCCTGCTGACACCGGC
GACTACAAATACTTCAACGTTGAATACGCCATGAATTATTGGAAGAGCAACGGTGCCCC
AGCTGAGAAGCTCCTGGTGGGATTCCCAACCTACGGAAAGAGCTTCACCCTGCAGAACC
CATCCGACACCTCCGTTGGAGCTCCCGCATCCGGCCCTGGCCCCGCTGGGCCTTACACC
AGGGAGGCTGGAACTCTGGCTTACTACGAGATCTGCAGTCTCCTGAGTTCTGGAGCCAC
CGAGGCTTGGGATGAACCCCAGGATGTTCCCTACGCCTACAAGGGCAGCGAATGGGTCG
GCTACGACAACGTCAAGAGCTTCGGCCTCAAGGTGGACTGGCTGAAGAAGAACAACTTT
GGAGGTGCCATGGTGTGGGCCCTGGACATGGATGACTTCACTGGCACCTTCTGCCACGA
GGGCAAATACCCCCTGATCTCCACCCTGAAGAAGGGCCTGGGGCTGCAGAACGGCGACT
GCGTTCCCCCTGCTGAGCCCCTGCCTCCCATCACCGAGGCTCCCACCACCACCACCACC
AGCGGCGGCGGCGGCTCCGGCTTCTGCGCCGGGAAACCCAACGGGATCTACGCAGACCC
CGAGAACAACAGGAACTTCTACAACTGCGTGAACGGCCAGGGCATCGTGCAGAGCTGCG
AGCCAGGGCTGGTCTTCGACCCCCTCTGCAGCTGCTGCAACTGGCCCCAG
>Parus_major_2
ATGAACAACAACGAGATCACCACCTACGAGTGGAATGACGAGACCCTGTACAAGTCCTT
CAATGGCCTCAAGAACCAGAACAGCAACCTGAAGACCCTGCTGGCCATTGGAGGATGGA
ATTTCGGCACACAGAAGTTCACCACCATGGTCTCCACACCCCAGAACCGCCAGACCTTC
ATCAACTCCGTGGTCAGGTTCCTGCGCCAGTATGGATTTGACGGGCTGGACCTGGACTG
GGAATACCCCGGCTCCAGGGGCAGCCCTGCCCAGGACAAGGCTCTCTTCACTGTCCTGG
TTAAGGAAATGCTGGCAGCCTTCGAGCAGGAGGCCAGGCAGACCAACCGGCCCCGGCTC
ATGGTCACCGCTGCTGTGGCCGGAGGACTTTCCACCATCCAGGCTGGCTACGAGATTGC
TGAGCTGGGCAAGTACCTGGATTACATCCACGTGATGACCTACGACTTCCACGGGCCCT
GGGATGGCTCTGCAGGCGAGAACAGCCCCCTGTTCAGCAGCGGCAGCACCCTCAGTGTT
GAATACGCCATGAACTACTGGAAGAACAACGGTGCCCCAGCTCAGAAGCTCCTGGTGGG
ATTCCCAACCTACGGGAAAACCTTCACCCTGCAGAACCCATCCAACACCGCCATCGGAG
CCCCAACCTCCGGGCCTGGCCCCGCGGACCCTACACCAGGGAGGCCGGGCTCTTGGCT
TACTACGAGATCTGCACCTTCCTGAGCTCCGGAGCCACCCAGGCTTGGGATGCCCCTGA
GGATGTTCCCTACGCCTACAAGGGCAGCGAATGGGTCGGCTACGACAACGTCAAGAGCT
TCGGCCTCAAGGTGGACTGGCTGAAGCAGAATAACTTTGGAGGTGCCATGGTGTGGACC
ATTGACCTGGATGACTTCACTGGCACCTTCTGCCACGAGGGCAAATACCCCCTGATCTC
CACCCTGAAGAAGGGCCTGGGGCTG
>Molothrus_ater_2
ATGGCCAAGCTCACTCTGCTCACCGGTCTGGCCCTGCTGCTGAACGCCCAGCTCGGCAC
TGCCTATGTGCTGACCTGTTACTTCACCAACTGGGCCCAGTACAGGCCTGGGGAGGGGA
GGTACACCCCCGAGAACATCGACCCCAACCTGTGCAACCACCTGATCTACGCCTTCGCC
GGCATGAACAACAACGAGATCACCACCTACGAGTGGAACGACGAGACCCTCTACAAGTC
CTTCAATGGCCTCAAGAACCAGAATAGGAACCTGAAGACCCTGCTGGCCATTGGAGGAT
GGAATTTCGGCACACAGAAGTTCTCCACCATGGTCTCCACGCCCCAGAACCGCCAGACC
TTTATCAACTCCGTCATCAAATTCCTGCGCCAGTACGGATTCGATGGGCTGGACCTGGA
CTGGGAATACCCCGGCTCCAGGGGCAGCCCTGCCCAGGACAAGTCTCTCTTCACCGTCC
TGGTTAAGGAAATGGTGGCAGCCTTCGAGCAGGAAGCCAAGCAGACCAACAGGCCCCGG
CTCATGGTCACTGCTGCTGTTGCTGGAGGAGTTTCCACCATCCAGTCTGGCTACGAGAT
CGCTGAGCTGGGCAAGTACCTGGATTACATCCACGTCATGACCTACGACTTCCACGGGC
CCTGGGACGGCTCCACGGGCGAGAACAGCCCCCTGTTCAGCAGCGGCAGCACCCTCAGT
GTTGAATACGCCATGAACTACTGGAAGAACAACGGCGCCCCAGCCCAGAAGCTGCTGGT
GGGATTCCCAACCTACGGAAAGAGCTTCACCCTGCAGAACCCATCCAACACGGCCATCG
GAGCCCCCAGCTCCGGGCCTGGCCCTGCAGGGCCCTACACCGGGGAGGCCGGACTCCTG
GCTTACTACGAGATCTGCACCTTCCTGAGCTCTGGAGCCACCCAGGCTTGGGATGCCCC
TGAGGATGTCCCCTACACCTACAAGGGCAGCGAATGGGTCGGCTACGACAACGAGAGGA
GCTTTGGCCTCAAGGTGGACTGGCTGAAGAAGAACAACTTTGGAGGGGCCATGGTGTGG
ACCATCGACCTGGATGACTTCACTGGCACCTTCTGCCACCAGGGCAAATACCCCCTGAT
CTCCACCCTGAAGAAGGGCCTGGGGCTG
>Molothrus_ater_1
ATGTTTGTCCATCCCTGTCGGTCCCACGTTGGCAGCAGAGAGGAGCAGGGTATAAAAGA

GGGAGAGAGCCCAAGCACATCAGTCTGGTCCAAGATGGCCAAGCTCACTCTGCTCACCG
GTCTGGCCCTGCTGCTGAACGCCCAGCTCGGCACTGCCTATGTGCTGACCTGTTACTTC
ACCAACTGGGCCCAGTACAGGCCTGGCCTGGGCAAGTTCACCCCCGAAAATGTCGACCC
TTGCCTGTGCAACCACCTGATCTACGCCTTCGCCGGCATGAACAACAACGAGATCACCA
CCTACGAGTGGAACGACGAGACCCTCTACAAGTCCTTCAATGGCCTCAAGAACCAGAAC
AAAGATCTGAAGACCCTGCTGGCCATTGGAGGATGGAATTTCGGCACAGCCAAGTTCTC
CACCATGGTCTCCACGCCCCAGAACCGCCAGACCTTCATCAACTCCGTCATCAAATTCC
TGCGCCAGTATCAGTTTGATGGGCTGGACCTGGACTGGGAATACCCCGGCTCCAGGGGC
AGCCCTGCCCAGGACAAGTCTCTCTTCACCGTCCTGGTTAAGGAAATGGTGGCAGCCTT
CGAGCAGGAAGCCAAGCAGACCAACAGGCCCCGGCTCATGGTCACTGCTGCTGTTGCTG
CAGGACTCTCCACCATCCAGTCTGGCTACGAGATCGCTGAGCTGGGCAAGTACCTGGAT
TACATCCACGTCATGACCTACGACTTCCACGGCTCCTGGGAGAGGAACACGGGCGAGAA
CAGCCCCCTGTTTGCTGGCCCTGCTGACAGCGGCGACTACAAATACTTCAACGTTGAAT
ACGCCATGAATTATTGGAAGAGCAATGGTGCCCCAGCTGAGAAGCTCCTTGTGGGATTC
CCAACCTATGGAAAGAGCTTCACCCTGCAGAGCCCATCTGACACCTCTGTTGGGGCTCC
AGCATCCGGCCCTGGCCCCGCTGGGCCCTACACCAGGGAAGCTGGAACTCTGGCTTACT
ACGAGATCTGCTCCCTCCTGAGCTCTGGAGCCACCCAGGCTTGGGATGAACCCCAGGAT
GTCCCCTACACCTACAAGGGCAGCGAATGGGTCGGCTACGACAACATCAAGAGCTTTGG
CCTCAAGGTGGACTGGCTGAAGAAGAACAACTTTGGAGGAGCCATGGTGTGGGCCCTGG
ACATGGATGACTTCACTGGGGATTTCTGCAAGGAAGGCAAATACCCCCTGATCTCCACC
CTGAAGAAGGGCCTGGGGCTGGAGAGTGGCGACTGCGTTCCCCCCGCTGAGCCCCTTCC
TCCCATCACTGAGGCTCCCACCACCACCAGCGGTGGCTCCGGCGGCTCCGGCGGCTCCG
GCGGCTCTGGATTCTGTGCCGGGAAACCCAACGGGATCTACGCAGACCCCAACAACAAG
AGGAACTTCTACAGCTGCCTGAACGGCCAGACCTTCGTGCAGAGCTGCGAGGAGGGGCT
GGTCTTCGACCCCGCCTGCTCCTGCTGCAACTGGCCCCAG
>Catharus_ustulatus_2
ATGGCCAAGCTCACTCTGCTCACCGGCCTGGCCCTGCTGCTGAACGCCCACCTCGGCTC
TGCCTATGTGCTCACCTGTTACTTCACCAACTGGGCCCAGTACCGGCCTGGTGAGGGCA
AATACACCCCGGAGAACATCGACCCCAACCTGTGCAGCCACCTGATCTACGCCTTCGCT
GGCATGAACAACAATGAGATCACCACGTACGAGTGGAACGATGAGACCCTGTACAAGTC
CTTCAATGGCCTCAAGAACCAGAACAGGAACCTGAAGACCCTGCTGGCCATTGGGGGAT
GGAATTTCGGCACACAGAAGTTCACCACCATGGTCTCCACACCCCAGAACCGCCAGACC
TTCATCAACTCCGTGGTCAGGTTCCTGCGCCAGTACGGATTCGATGGGCTGGACCTGGA
CTGGGAATACCCCGGCTCCAGGGGCAGCCCTGCCCAGGACAAGGCTCTCTTCACCGTCC
TGGTTAAGGAACTGCTGGCAGCCTTCGAGCAGGAAGCCAAACAGACCAACCAGCCCCGG
CTCATGGTCACCGCCGCTGTGGCCGGAGGACTTTCCACCATCCAGGCTGGCTACGAGAT
CGCTGAGTTGGGCAAGTACCTGGACTACATCCACGTCATGACCTACGACTTCCACGGGC
CCTGGGACGGCTCCACGGGCGAGAACAGCCCCCTGTTCAGCAGTGGCAGCACCCTCAGT
GTTGAATATGCAATGAACTACTGGAAGAACAACGGTGCCCCAGCTCAGAAGCTGCTGGT
GGGATTCCCAACCTATGGGAAAACCTTCACTCTGCAGAGCCCATCCAACACAGCTGTGG
GAGCCCCCAGCTCCGGCCCCGGCCCCGCAGGACCCTACACCAGGGAGGCCGGGCTCTTG
GCTTACTACGAGATCTGCAGCTTCCTGAGCTCTGGAGCCACCCAGGCCTGGGATGCCCC
TGAGGATGTTCCCTACGCCTACAAGGGCAGTGAATGGATCGGCTATGACGACATCAAGA
GCTTCGGCCTCAAGGTGGATTGGCTGAAGAAGAACAACTTTGGAGGAGCCATGGTGTGG
ACCATCGACCTGGATGACTTCACTGGCAACTTCTGCCACGAGGGCAAATACCCGCTGAT
CTCCACGCTGAAGAAGGGCCTGGGGCTG
>Catharus_ustulatus_1
ATGGCCAAGCTCACTCTGCTCACCGGCCTGGCCCTGCTGCTGAACGCCCACCTCGGCTC
TGCCTATGTGCTCACCTGTTACTTCACCAACTGGGCCCAGTACCGGCCTGGCCTGGGTA
AGTTCACCCCTGAAAATGTCGACCCCTGCCTGTGCAACCACCTGATCTACGCCTTCGCT
GGCATGAACAACAACGAGCTCACCACGTACGAGTGGAACGACGAGACCCTGTACAAGTC
CTTCAATGGCCTCAAGAACCAGAACAAAGATCTGAAGACCCTGCTGGCCATTGGAGGAT
GGAATTTCGGCACAGCCAAGTTCACCACCATGGTCTCCTCTCCCGAGAACCGCCAGACC
TTCATCAAGTCCGCCATCAAATTCCTGCGCCAGTACCAGTTTGACGGGCTGGACCTGGA

CTGGGAATACCCCGGCTCCAGGGGCAGCCCTGCCCAGGACAAGGCTCTCTTCACCGTCC
TGGTTAAGGAACTGCTGGCAGCCTTCGAGCAGGAAGCCAAACAGACCAACCAGCCCCGG
CTCATGGTCACCGCCGCTGTGGCTGCCGGACTCTCCACCATCCAGGCTGGCTACGAGAT
CGCTGAGATTGGCAAGTACCTGGACTACATCCACGTCATGACCTACGACTTCCACGGCT
CCTGGGAGAGGAACACCGGCGAGAACAGCCCCCTGTTCGCCGGCCCCGCTGACACCGGC
GACTACAAATACTTCAACGTTGAATACGCCATGAATTATTGGAAGAGCAATGGTGCCCC
AGCTGAGAAACTCCTGGTGGGATTCCCAACCTATGGAAAGAGCTTCACCCTGCAGGACC
CATCCAACACAGCTGTGGGAGCTCCAGCATCCGGCCCTGGCCCTGCAGGACCCTACACC
AGGGAGGCTGGAACTCTGGCTTACTATGAGATCTGCTCTCTGCTGAGCTCTGGAGCCAC
CCAGGCTTGGGATGAACCCCAGGATGTTCCCTACGCCTACAAGGGCAGCGAATGGGTCG
GCTACGACAACGTCAAGAGCTTCGGCCTCAAGGTGGATTGGCTGAAGAAGAACAACTTT
GGAGGAGCCATGGTCTGGGCCCTGGACATGGATGACTTCACTGGGGATTTCTGCAAGGA
AGGCAAATACCCGCTGATCTCCACGCTGAAGAAGGGCCTGGGGCTGCAGAACGGCGAGT
GTGTCCCCCCCAGTGAGCCCCTGCCTCCCATCACCGAGGCTCCCACCACCCCCAGCGGC
GGCGGCTCCGGCGGCTCCGGTGGCTCCGGCGGCTCCGGGTTCTGTGCTGGAAAACCCAA
CGGGATTTATGCAGATCCCGACAACAAGAGCAACTTCTACAACTGTGTGAACGGGCAGA
GCTACCTGGAGAGCTGCCAGCAGGGGCTGGTGTTCGACCCTGCCTGTACCTGCTGCAAC
TGGCCCCAG
>Camarhynchus_parvulus_2
ATGGCCAAGCTCACTGTGCTCACCGGTCTGGCCCTGCTGCTGAACGCCCAGCTCGGCAC
TGCCTATGTGCTGACCTGCTACTTCACCAACTGGGCCCAGTACAGGCCTGGTGAGGGGA
GATACACCCCCGAGAACATCGACCCCAACCTGTGCAACCACCTGATCTACGCCTTCGCC
GGCATGAACAACAACGAGATCACCACCTACGAGTGGAACGACGAGACCCTCTACAAGTC
CTTCAATGGCCTCAAGAACCAGAACAGGAAGCTGAAGACCCTGCTGGCCATCGGAGGAT
GGAATTTCGGCACACAGAAGTTCTCCACCATGGTCTCCACACCCCAGAACCGCCAGACC
TTCATCAACTCCGTGGTCAGGTTCCTGCGCCAGTACGGATTCGATGGGCTGGACCTGGA
CTGGGAATACCCCGGCTCCAGGGGCAGCCCTGCCCAGGACAAGTCTCTCTTCACCGTCC
TGGTTAAGGAAATGGTGGCAGCCTTCGAGCAGGAAGCCAAACAGACCAACAGGCCCCAG
CTCATGGTCACTGCTGCTGTGGCTGGAGGACTCTCCACCATCCAGTCTGGCTATGAGAT
CGCTGAGCTGGGCAAGTACCTGGATTACATCCACGTCATGACCTACGACTTCCACGGGC
CCTGGGATGGCTCCACGGGCGAGAACAGCCCCCTGTTCAGCAGCGGCAGCACCCTCAGT
GTTGAATACGCCATGAACTACTGGAAGGACAACGGCGCCCCAGCCCAGAAGCTGCTGGT
GGGATTCCCAACCTACGGAAAGAGCTTCACCCTGCAGAACCCATCCAACACGGCCATCG
GAGCCCCCAGCTCCGGGCCTGGCCCTGCAGGGCCCTACACCAAGGAGGCCGGGCTCCTG
GCTTACTACGAGATCTGCACCTTCCTGAGCTCTGGAGCCACCCAGGTTTGGGATGCCCC
TGAGGATGTCCCTTACGCCTACAAGGCCAACGAATGGGTCGGCTACGACAACGAGAAGA
GCTTTGGCCTCAAGGTGGATTGGCTGAAGAAGAACAACTTTGGAGGGGCCATGGTGTGG
ACCATCGACCTGGATGACTTCACTGGCAACTTCTGCCACCAGGGCAAATACCCACTGAT
CTCCAGCCTCAAGAGGGGCCTGGGGCTG
>Camarhynchus_parvulus_1
ATGTTTGTCCATCCCTGTCGGTCCCACGTTGGCAGCAGAGAGGAGCAGGGTATAAAAGA
GGGAGAGAGCCCGAGCACATCAGTCTGGTCCAGGATGGCCAAGCTCACTGTGCTCACCG
GTCTGGCCCTGCTGCTGAACGCCCAGCTCGGCACTGCCTATGTGCTGACCTGCTACTTC
ACCAACTGGGCCCAGTACAGGCCTGGCCTGGGCAAGTTCACCCCTGAAAATGTCGACCC
CTGTCTGTGCAACCACCTGATCTACGCCTTCGCCGGCATGAACAACAACGAGATCACCA
CCTACGAGTGGAACGACGAGACCCTCTACAAGTCCTTCAATGGCCTCAAGAACCAGAAC
AAAGATCTGAAGACCCTGCTGGCCATTGGAGGATGGAATTTCGGCACACAGAAGTTCTC
CACCATGGTCTCCACACCCGAGAACCGCCAGACCTTCATCAAGTCCGTCATCAAATTCC
TGCGCCAGTATCAGTTTGATGGGCTGGACCTGGACTGGGAATACCCCGGCTCCAGGGGC
AGCCCTGCCCAGGACAAGTCTCTCTTCACCGTCCTGGTTAAGGAAATGGTGGCAGCCTT
CGAGCAGGAAGCCAAGCAGACCAACAGGCCCCAGCTCATGGTCACTGCTGCTGTGGCTG
CTGGACTCTCCACCATCCAGTCTGGCTATGAGATCGCTGAGCTTGGCAAGTACCTGGAT
TACATCCACGTCATGACCTACGACTTCCATGGCTCCTGGGAGAGGAACACCGGCGAGAA
CAGCCCCCTGTTCGCCGGCCCTGCTGACAACGGCGACTACAAATACTTCAACGTTGAAT

ACGCCATGAATTATTGGAAGAGCAATGGTGCCCCAGCTGAGAAGCTCCTTGTGGGATTC
CCAACCTATGGAAAGAGCTTCACCCTGCAGAACCCATCTGACACCTCTGTTGGAGCTCC
AGCATCCGGCCCTGGCCCTGCTGGGCCCTACACCAGGGAGGCTGGAACTCTGGCTTACT
ACGAGATCTGCTCTCTCCTGAGCTCTGGAGCCACCCAGGCTTGGGATGAGCCCCAGGAT
GTCCCCTACGCCTACAAGGGCAGCGAATGGGTCGGCTATGACAACATCAAGAGCTTTGG
CCTCAAGGTGGACTGGCTGAAGAAGAACAACTTTGGAGGGGCCATGGTGTGGGCCTTGG
ACATGGATGACTTCACTGGGGATTTCTGCAAGGAAGGCAAATACCCGCTGATCTCCAGC
CTGAAGAAGGGCCTGGGGCTGCAGAGTGGTGACTGCGTTCCCCCCTCTGAGCCCCTTCC
TCCCATCACTGAGGCTCCCACCACCACCACCAGCGGCGGCTCTGGCGGCTCCGGTGGCT
CTGGCGGTTCCGGTGGCTCTGGATTCTGTGCCGGGAAAGCCAACGGGATCTACGCAGAC
CCCAACAACAAGAAGAACTTCTACAACTGCCTGAACGGCCAGACCTTCGTGCAGAGCTG
CATGGAGGGGCTGGTCTTCGACCCCGCCTGCTCCTGCTGCAACTGGCCCCAG
>Pipra_filicauda_1
ATGGCCAAGCTCACTCTGCTCACCGGTCTGGCCCTGCTGCTCAACGCCCACCTCGGTAG
CAGGCAGCCCCCCCAGCTCCTGTGTCTCCCCACAGGCACTGCTTACGTGCTGACATGTT
ACTTCACCAACTGGGCCCAGTACAGGCCTGGCCTGGGCAAATACACACCCGAGAACATC
GACCCCAACCTGTGCAGCCACCTGATCTACGCCTTCGCCGGGATGAACAACAACGAGAT
CACCACTTATGAATGGAACGACGAGACCCTCTACAAATCCTTCAACGGCCTGAAGAACC
AGAACAGAAACCTGAAGACCCTGCTGGCCATTGGAGGATGGAATTTTGGCACAGAAAAG
TTCACTACGATGGTTTCCACCCCCCAGAACCGCCAGACTTTCATCAATTCCGTTGTCAG
ATTCCTGCGCCAGTATGGATTCGATGGGCTGGACCTGGACTGGGAATACCCCGGCTCCA
GGGGCAGCCCAGCCCAGGACAAGGGGCTCTTCACTGTCCTAGTTAAGGAATTGCTGGCA
GCCTTTGAGCAGGAAGCCAAACAGACCAACCAGCCCCGTCTCCTGGTCACCGCGGCTGT
GGCTGGAGGACTTTCCACCATCCAGGCCGGCTACGAGATTGCTGAGCTGGGCAAGTACC
TGGACTACATCCACGTGATGACCTACGACTTCCACAGCCCCTGGGACGGCTCCACTGGC
GAGAACAGCCCCCTGTTCAGCAGCGGCAGCACCTTCAGTGTGGAATACGCTATGAACTA
CTGGAAGAACAATGGTGCCCCAGCTCAGAAGCTCCTGGTTGGCTTCCCAACCTACGGAA
AGACCTTCACCCTGCAAAACCCCTCCAACACTGGGATTGGGGCCCCAACCTCGGGCCCT
GGGCCAGCAGGACCTTACACCAGGGAGGCCGGGCTTCTGGCTTACTACGAGATCTGCTC
GTTCCTGAACACCGGAGCCACCCAGTCTTGGGATGCCCCTGAGGACGTGCCCTATGCCT
ACAAGGGCAACGAGTGGATTGGCTACGACAACACAAAGAGCTTCAGTGCAAAGGTCGAC
TGGCTGAAGCAGAACAACTTTGGAGGGGCCATGGTTTGGACCATCGACCTGGATGACTT
CACTGGCACTTTCTGCCATGAAGGCAAATACCCCCTGATCTCCACCCTGAAGAAGGGCC
TTGGTCTG
>Pipra_filicauda_2
ATGAACAACAACGAGATCACCACTTATGAATGGAACGACGAGACCCTCTACAAATCCTT
CAACGGCCTGAAGAACCAGAACAAGGATCTGAAGACACTCCTGGCCATTGGAGGATGGA
ATTTTGGCACAGCCAAGTTCACTACAATGGTTTCCTCTGCTGAGAACCGCCAGACCTTC
ATCACGTCTGTCATCAAATTCCTGCGCCAGTACCAGTTTGATGGGTTGGACCTGGACTG
GGAATACCCCGGCTCCAGGGGCAGCCCAGCCCAGGACAAGGGGCTCTTCACCGTCCTGG
TTAAGGAATTGCTGGCAGCCTTTGAGCAGGAAGCCAAACAGACCAACCAGCCCCGTCTC
CTGGTCACCGCGGCTGTGGCTGGAGGACTTTCCACCATCCAGGCCGGCTACGAGATTGC
TGAGCTGGGCAAGTACCTGGACTACATCCACGTGATGACCTATGACTTCCACGGCTCCT
GGGAGAGAAACACTGGCGAGAACAGCCCCCTGTTCACAGGCCCAGCTGACACTGGGGAC
TACAAGTACTTCAACGTCGAATATGCTATGAATTACTGGAAGGATAATGGTGCCCCAGC
TGAGAAGCTCCTTGTTGGCTTCCCAACCTACGGAAAAAGCTTCACCCTGCAAAACCCAT
CTGACACCTCTGTTGGGGCTCCAGCATCAGGCCCTGGACCAGCTGGACCTTACACCAGG
GAGGCTGGGACACTGGCTTACTATGAGATTTGCTCTCTCCTGAGCTCTGGAGCCACTCA
GGCTTGGGATGAACCCCAGGACGTGCCCTATGCCTACAAGGAGAGCGAATGGGTTGGCT
ATGACAACACAAAGAGCTTCAGCATCAAGGTCGACTGGCTGAAGAAGAATAACTTTGGA
GGGGCCATGGTTTGGGCCCTTGACATGGATGATTTCACTGGCACTTTCTGCAATGAAGG
CAAATACCCCCTGATCTCCACCCTGAAGAAGGGCCTCGGTCTGCAGAATGGTGACTGTG
TGCCTCCTGCTGAGCCCCTGCCTCCAGTCACTGAGGCTCCCACTACCACCAGTGGAAGT
GGGAGCGGTGGCTCTGGTGGGAGCAGCGGCTCTGGTGGGAGCGGCTTCTGCGCCGGGAA

AGCCAACGGCATCTACGCAGACCCAGAGAACAGCAACAACTTCTACAACTGCTTGAACG
GCCAAACCTTCGTGCAGAGCTGCCAACAGGGCCTCGTCTTCGACCCCGTCTGCTCCTGC
TGCAACTGGCCA
>Cyanistes_caeruleus_1
ATGGCCAAGCTCACTCTGCTCACCGGCCTGGCGCTGCTGCTGAACGCCCAGCTCGGCAC
TGCCTATGTGCTGACCTGTTACTTCACCAACTGGGCCCAGTACCGGCCTGGCCTGGGCA
AGTACACCCCCGAAAATGTTGACCCCTGCTTGTGCAACCACCTGATCTACGCCTTTGCC
GGCATGAACAACAATGAGATCACCACCTATGAGTGGAACGACGAGACCCTGTACAAGTC
CTTCAACGGCCTCAAGAACCAGAACAAAGATCTGAAGACCCTGCTGGCCATTGGAGGAT
GGAATTTCGGCACACAGAAGTTCACCACCATGGTCTCCACACCCCAGAACCGCCAGACC
TTCATCAACTCCGTCATCAAATTCCTGCGCCAGTATCAGTTTGACGGGCTGGATCTGGA
CTGGGAATACCCCGGCTCCAGGGGCAGCCCCGCTCAGGACAAGGCTCTCTTCACCGTCC
TGGTTAAGGAACTGCTGGAAGCCTTCGAGAAGGAAGCCAAACAGACCAACCAGCCCCGT
CTCATGGTCACCGCCGCTGTGGCTGGAGGACTTTCCACCATCCAGGCTGGCTACGAGAT
TGCTGAGATTGGCAAGTACCTGGATTACATCCACGTGATGACCTACGACTTCCACGGAT
CCTGGGAGAGGAACACTGGCGAGAACAGCCCCCTGTTCGCCGGCCCTGCTGACACTGGC
GACTACAAATACTTCAACGTTGAATACGCCATGAATTATTGGAAGAGCAACGGTGCCCC
AGCTGAGAAGCTCCTTGTGGGATTCCCAACCTATGGAAAGAGCTTCACCCTGCAGAACC
CATCCGACACCTCCGTTGGAGCTCCAGCATCCGGCCCTGGCCCCGCTGGGCCCTATACC
AGGGAGGCCGGAACTCTGGCTTACTACGAGATCTGCAGTCTCCTGAGTTCTGGAGCCAC
CCAGGCTTGGGATGAACCCCAGGATGTTCCCTACGCCTACAAGGGCAGCGAATGGGTCG
GCTACGACAACGTCAAGAGCTTCGGCCTCAAGGTGGACTGGCTGAAGAAGAACAACTTT
GGAGGTGCCATGGTGTGGGCCCTGGATATGGATGACTTCACTGGCACCTTCTGCCACGA
GGGCAAATACCCCCTGATCTCCAGCCTGAAGAAGGGCCTGGGGCTGCAGAACGGCGACT
GCGTTCCCCCTGCTGAGCCCCTGCCTCCCATCACCGAGGCTCCCACCACCACCACCACC
AGCGGCGGCAGCGGCGGCTCCGGCTTCTGCGCCGGGAAACCCAACGGGATCTACGCAGA
CCCCGAGAACAACAGGAACTTCTACAACTGCGTGAACGGCCAGGGCATCCCGCAGAGCT
GCGAGCCAGGGCTGGTCTTCGACCCCCTCTGCAGCTGCTGCAACTGGCCCCAG
>Cyanistes_caeruleus_2
ATGGCCAAGCTCACTCTGCTCACCGGTGAGTGCCGCTGCAAACTGGAGCTCTATGCCCT
CCTGGCCGGGCTGGAGGCTCTGCCTGCCCCGGCAGCAGCGCGGGGATGCTGTGCCCGAG
GGGCCGGGCTGTGCCCAGGGCACTGCAGGCCCCTCCATGGCCGGTGTCTCCCTGCAGGC
ACTGCCTATGTGCTGACCTGTTACTTCACCAACTGGGCCCAGTACCGGCCTGGTGAGGG
CAGATACACCCCTGAGAACATTGACCCCAACCTGTGCAGCCACCTGATCTACGCCTTTG
CCGGCATGAACAACAACGAGATCACCACCTACGAGTGGAACGACGAGACCCTGTACAAG
TCCTTCAACGGCCTCAAGAACCAGAACAGCAACCTGAAGACTCTGCTGGCCATTGGAGG
ATGGAATTTCGGCACAGAGAAGTTCACCACCATGGTCTCCACACCCCAGAACCGCCAGA
CCTTCATCAACTCCGTGGTCAGGTTCCTGCGCCAGTACGGATTTGACGGGCTGGACCTG
GACTGGGAATACCCCGGCTCCAGGGGCAGCCCCGCTCAGGACGGCTCTNNCTTCACCGT
CCTGGTTAAGGAACTGCTGGCAGCCTTCGAGCAGGAGGCCAGGCAGACCAACCGGCCCC
GGCTCATGGTCACTGCTGCTGTGGCTGGAGGACTTTCCACCATCCAGGCTGGCTACGAG
ATTGCTGAGCTGGGCAAGTACCTGGATTACATCCATGTGATGACCTATGACTTCCACGG
GCCCTGGGACGGCTCTGCAGGCGAGAACAGCCCCCTGTTCAGCAGTGGCAGCACCCTCA
GTGTTGAATATGCCATGAACTACTGGAAGAACAACGGTGCCCCAGCTCAGAAGCTCCTG
GTGGGATTCCCAACCTACGGGAAAACCTTCACCCTGCAGAACCCATCCAACACCGCCAT
CGGAGCCCCAACCTCCGGGCCAGGCCCCGCTGGGCCCTACACCAGGGAGGCCGGGCTCT
TGGCTTACTACGAGATCTGCACCTTCCTGAGCTCTGGAGCCACCCAGGCTTGGGATGCC
CCTGAGGATGTTCCCTACGCCTACAAGGGCAGCGAATGGGTCGGCTACGACAACGTCAA
GAGCTTCGGCCTCAAGGTGGACTGGCTGAAGCAGAACAACTTCGGAGGTGCCATGGTGT
GGACAATCGACCTGGATGACTTCACTGGCACCTTCTGCCATGAGGGCAAATACCCCCTG
ATCTCCAGCCTGAAGAAGGGGCTGGGGCTG
>Lonchura_striata_1
ATGGCCAAGCTCGCTCTGCTCAGCGGCCTGGCCCTGCTGCTGAACGCCCACCTCGGCAC
TGCCTTTGTGCTGAGCTGTTATTTCACCAACTGGGCCCAGTACCGGCCTGGCCTGGGCA

AGTTCACCCCCGAAAACATCGACCCCTGCCTGTGCACCCACCTGATCTATGCCTTCGCC
GGCATGAACAACAACGAGATCTCCACATCCGAGTGGAACGACGAGAGCCTCTACAAGTC
CTTCAATGGCCTCAAGAACCAGNNNAAAGATCTGAAGACCCTGCTGGCCATTGGAGGAT
GGAATTTCGGCACACAGAAGTTCTCCACCATGGTCTCCACACCCCAGAACCGCCAGACC
TTCATCAAGTCCGTCGTCAAATTCCTGCGGCAGCACGGATTCGATGGGCTGGACCTGGA
CTGGGAATTCCCCGGCTCCAGGGGCAGCCCTGCCCAGGACAAGGCTCTCTTCACCGTCC
TGGTTAAGGAGCTGCGGGCAGCCTTCGAGCAGGAGGCCAAGCACAGCAACCAGCCCCAG
CTCATGGTCACCGCCGCTGTGGCCGCCGGGCTTCCCACCATCCAGGCCGGCTACGAGAT
CGCTGAGCTGGGCAGGTCCCTGGATTACATCCACGTCATGAGCTACGACTTCCACGGCT
CCTGGGAGAGGAACACGGGTGAGAACAGCCCCCTGCTCCCCGGCCCTGCTGACAGCGGT
GACTACAAATACTTCAACGTCGAATACGCCATGAATTATTGGAAGAGCAATGGTGCTCC
AGCTGAGAAGCTCCTTGTGGGATTCCCGACCTATGGGAAGAGCTTCACCCTGCAGAACC
CATCCGACACCTCCGTTGGGGCTCCAGCGTCCGGCCCTGGCCCCGCCGGGCCCTTCACC
AGGGAGGCCGGAACTCTGGCTTACTACGAGATCTGCACCTTCCTGAGCTCCGGAGCCAC
CCAGGCTTGGGATGCCCCCCAGTATGTCCCCTACGCCTACAAGGGCAGCGAATGGGTGG
GCTACGACAACGTCAGGAGCTTCGGCCTCAAGGTGGATTGGCTGAAGGAGAACAACTTC
GGAGGGGCCATGGTGTGGGCCCTGGACATGGATGACTTCACTGGGGGTTTCTGCAAGGA
AGGCAAATACCCGCTCATCTCCAGCCTGAGGAAGGGCCTGGGGCTGCAGAGTGGCGACT
GCGTTCCCCCCGCTGAGCCCCAGCCTCCCATCACTGAGGCTTCCACCACCACCAGCGGT
GGCTCCGGTGGCTCCGGTTTCTGTGCCGGGAAACCCAATGGGATCTATGCAGACCCCAG
CAACAAGAGGAACTTCTACAGCTGCCTGAATGGCCAGACCTTCGTGCAGAGCTGCCAGC
CTGGGCTGGTCTTCGACCCCGTCTGCTCCTGCTGCAACTGGCCCCAG
>Ficedula_albicollis_1
ATGGCCAAGCTCACTCTGCTCACCGGCCTGGCCCTGCTGCTGAACGCCCACCTCGGCAC
TGCCTATGTGCTGACCTGCTACTTCACCAACTGGGCCCAGTACCGACCTGGCCTGGGTA
AGTTCACCCCCGAAAATGTCGACCCCTGCCTGTGCAACCACCTGATCTACGCCTTCGCT
GGCATGAACAACAACGAGCTCACCACCTACGAGTGGAACGACGAGACCCTGTACAAGTC
CTTCAATGGCCTCAAGAACCAGAACAAAGATCTGAAGACGCTGCTGGCCATTGGGGGAT
GGAATTTCGGCACAGCCAAGTTCACCACCATGGTCTCCTCTCCTGAGAACCGCCAGACC
TTCATCAAGTCCGCCATCAAATTCCTGCGCCAGTACCAGTTTGATGGGCTGGACCTGGA
CTGGGAATACCCCGGCTCCAGGGGCAGCCCCGCCCAGGACAAGGCTCTGTTCACCGTCC
TGGTTAAGGAGCTGCTGGCAGCCTTCGAGCAGGAAGCCAAACAGACCAACCAGCCCCGG
CTCATGGTCACTGCTGCTGTTGCTGCTGGACTTTCCACCATCCAGGCTGGCTACGAGAT
CGCCGAGATTGGCAAGTACCTGGATTACATCCACGTCATGACCTACGACTTCCACGGCT
CCTGGGAGAGGAACACGGGCGAGAACAGCCCCCTGTACGCCGGCCCTGCTGACACTGGT
GACTACAAATACTTCAACGTTGAATACGCCATGAATTATTGGAAGAGCAACGGTGCCCC
AGCTGAGAAACTCCTGGTGGGATTCCCAACCTATGGGAAGAGCTTCACCCTGCAGAACC
CATCCAACACGGCCGTGGGAGCCCCAGCGTCCGGCCCCGGCCCCGCCGGGCCCTACACC
AGGGAGGCTGGAACTCTGGCTTACTACGAGATCTGCACTCTGCTGAGCTCTGGAGCCAC
CCAGGCTTGGGATGAACCCCAGGATGTTCCCTACGCCTACAAGGACAGCGAGTGGGTCG
GCTACGACAACGTCAAGAGCTTTGGCATCAAGGTGGATTGGCTGAAGAAGAACAACTTT
GGAGGAGCCATGGTCTGGACCATT
>Ficedula_albicollis_2
ATGAACAACAACGAGCTCACCACCTACGAGTGGAACGACGAGACCCTGTACAAGTCCTT
CAATGGCCTCAAGAACCAGAACAGGAACCTGAAGACCCTGCTGGCCATTGGAGGATGGA
ATTTCGGCACACAGAAGTTCACCACCATGGTCTCCACACCCCAGAACCGCCAGACCTTC
ATCACCTCCGTGGTCAGGTTCCTGCGCCAGTACGGATTCGACGGGCTGGACCTGGACTG
GGAATACCCCGGCTCCAGGGGCAGCCCCGCCCAGGACAAGGCTCTGTTCACCGTCCTGG
TTAAGGAGCTGCTGGCAGCCTTCGAGCAGGAAGCCAAACAGACCAACCAGCCCCGGCTC
ATGGTCACTGCTGCTGTGGCCGGAGGACTGTCCACCATCCAGGCTGGCTACGAGATCGC
TGAGCTGGGCAAGTACCTGGATTACATCCACGTCATGACCTACGACTTCCACGGCCCT
GGGACGGCTCCACGGGCGAGAACAGCCCCCTGTTCAGCAGCGGCAGCACCCTCAGTGTG
GAATACGCCATGAACTACTGGAAGAACAACGGCGCCCCAGCTCAGAAGCTGCTGGTGGG
ATTCCCAACCTACGGGAAAAGCTTCACCCTGCAGAACCCATCCAACACAGCCGTGGGAG

CCCCCAGCTCTGGCCCTGGCCCCGCTGGGCCCTACACCAGGGAGGCTGGGCTCCTGGCT
TACTACGAGATCTGCACCTTCCTGAGCTCTGGAGCCACCCAGGCCTGGGATGCCCCTGA
GGATGTTCCCTACGCCTACAAGGGCAACGAATGGATCGGCTACGACAACGTCAAGAGCT
TCAGCCTCAAGGTGGATTGGCTGAAGAAGAACAACTTTGGAGGAGCCATGGTCTGGACC
ATCGACCTGGATGACTTCACTGGCAACTTCTGCCACGAGGGCAAATACCCTCTGATCTC
CACGCTGAAGAAGGGCCTGGGGCTG
>Passer_montanus_1
ATGGCCAAGCTCACTCTGCTCACCGGCCTGGCGCTGCTGCTGAACGCCCAGCTCGGCAC
TGCCTATGTGCTGACCTGCTACTTCACCAACTGGGCCCAGTACAGGCCCGGCCTGGGCA
AGTTCACCCCTGAAAATGTCGACCCTTGCCTGTGCAACCACCTGATCTACGCCTTTGCC
GGCATGAACAACAACGAGATCACCACCTACGAGTGGAACGACGAGACCCTGTACAAGTC
CTTCAACGGCCTCAAGAACCAGAACAAAGATCTGAAGACCCTGCTGGCCATTGGAGGAT
GGAATTTCGGCACAGCCAAGTTCTCCACCATGGTCTCCACTCCTGAGAACCGCCAGACC
TTCATCAAGTCCGTCATCAAATTCCTGCGCCAGTATCAGTTTGATGGGCTGGACCTGGA
CTGGGAATACCCCGGCTCCAGGGGCAGCCCTGCCCAGGACAAGTCTCTCTTCACCGTGC
TGGTTAAGGAGCTGCTGGCAGCCTTCGAGCAGGAAGCCAAAGAGACCAACAGGCCCCGG
CTCATGGTCACCGCCGCTGTCGCTGCCGGGCTTTCCACCATCCAGTCTGGCTACGAGAT
CGCCGAGCTGGGCAAGTACCTGGATTACTTCCACGTCATGACCTACGACTTCCACGGCT
CCTGGGAGAGGAACACTGGCGAGAACAGCCCCCTGTTCGCCGGCCCTGCCGACAGCGGC
GACTACAAATACTTCAACGTTGAATACGCCATGAACTATTGGAAGAGCAATGGTGCCCC
AGCTGAGAAGCTCCTGGTGGGATTCCCAACCTATGGAAAGAGCTTCACCCTGCAGAACC
CATCTGACACCTCTGTTGGAGCTCCAGCATCCGGCCCTGGCCCCGCTGGGCCCTACACC
AGGGAGGCTGGAACTCTGGCTTACTACGAGATCTGCTCTCTGCTGAGCTCTGGAGCCAC
CCAGGCTTGGGACGAACCCCAGGATGTCCCCTACGCCTACAAGGGCAGCGAGTGGGTCG
GCTACGACAACGTCAAGAGCTTTGGCCTCAAGGTGGACTGGCTGAAGAAGAACAACTTT
GGAGGAGCCATGGTGTGGGCCCTGGACATGGATGACTTCACTGGGGAATTCTGCAAGGA
AGGCAAATACCCCCTGATCTCCAGCCTGAAGAAGGGCCTGGGGCTGCAGAGCGGAGACT
GCGTTCCCCCCTCTGAGCCCCTGCCTCCCATCACTGAGGCTCCCACCACCACCAGCGGC
GGCTCCGGTGGCTCTGGCGGCTCCAGTGGCTCCGGTGGCTCTGGATTCTGTGCCGGGAA
ACCCAACGGGATCTACGCAGACCCCAACAACAAGAGGAACTTCTACAACTGCCTGAACG
GCCAGACCTTCGTGCAGAGCTGCGAGCAGGGGCTGGTCTTCGACCCCGTCTGCTCCTGC
TGCAACTGGCCCCAG
>Passer_montanus_2
ATGGCCAAGCTCACTCTGCTCACCGGCCTGGCGCTGCTGCTGAACGCCCAGCTCGGCAC
TGCCTATGTGCTGACCTGCTACTTCACCAACTGGGCCCAGTACAGGCCCGGTGAGGGGC
GATACACCCCTGAGAACATCGACCCCAACCTGTGCAGCCACCTGATCTACGCCTTTGCC
GGCATGAACAACAACGAGATCACCACCTACGAGTGGAACGACGAGACCCTGTACAAGTC
CTTCAACGGCCTCAAGAACCAGAACAGGAACCTGAAGACCCTGCTGGCCATTGGAGGAT
GGAATTTTGGCACACAGAAGTTCTCCACCATGGTCTCCACTCCCCAGAACCGCCAGACC
TTCATCAACTCCGTGGTCAGGTTCCTGCGCCAGTACGGATTCGACGGGCTGGACCTGGA
CTGGGAATACCCCGGCTCCAGGGGCAGCCCTGCCCAGGACAAGTCTCTCTTCACCGTGC
TGGTTAAGGAGCTGCTGGCAGCCTTCGAGCAGGAAGCCAGGCAGACCAACAGGCCCCGG
CTCATGGTCACCGCCGCTGTGGCCGGAGGGCTTTCCACCATCCAGTCTGGCTACGAGAT
CGCCGAGCTGGGCAAGTACCTGGATTACATCCACGTCATGACCTACGACTTCCACGGGC
CCTGGGACGGCTCCACGGGCGAGAACAGCCCCCTGTTCGCCAGCGGCAGCACCCTCAGC
GTTGAGTACGCCATGAACTACTGGAAGAACAACGGCGCCCCAGCTCAGAAGCTCCTGGT
GGGATTCCCAACCTACGGGAAGAGCTTCACCCTGCAGAGCCCCTCCAACACCGCCATCG
GCGCCCCCAGCTCCGGGCCCGGCCCCGCCGGACCCTACACCAGGGAGGCCGGGCTCCTG
GCTTACTACGAGATCTGCACCTTCCTGAGCTCTGGAGCCACCCAGACTTGGGATGCCCC
CGAGGATGTCCCCTACGCCTACAAGGGCAGCGAGTGGGTCGGCTACGACAACGAGAAGA
GCTTTGGCCTCAAGGTGGACTGGCTGAAGAAGAACAACTTTGGAGGAGCCATGGTGTGG
ACCATCGACCTGGATGACTTCACTGGCACCTTCTGCCACCAGGGCAAATACCCGCTGAT
CTCCACGCTGAAGAGGGGCCTGGGGCTG
>Chiroxiphia_lanceolata_2

ATGGTTTCCTCTGCTGAGAACCGCCAGACCTTCATCAAGTCTGTCATCAAATTCCTGCG
CCAGTACCAGTTTGATGGGCTGGACTTGGACTGGGAATACCCTGGCTCCAGGGGCAGCC
CAGCCCAGGACAAGGGGCTCTTCACCGTCCTGGTTAAGGAATTGCTGGCAGCCTTTGAG
CAGGAAGCCAAACAGACCAACCAGCCCCGTCTCCTGGTCACCGCGGCTGTGGCTGGAGG
ACTTTCCACCATCCAGGCTGGCTATGAGATTGCTGAGCTGGGCAAGTATCTGGACTACA
TCCACGTGATGACCTACGACTTCCACGGCTCCTGGGAGAGAAACACTGGTGAGAACAGC
CCCCTGTTCACAGGCCCAGCTGACACCGGGGACTACAAGTACTTCAATGTCGAATATGC
TATGAATTACTGGAAGGACAATGGTGCCCCAGCTGAGAAGCTCCTTGTTGGCTTCCCAA
CCTATGGAAAAAGCTTCACCCTGCAAAACCCATCTGACACCTCTGTTGGGGCTCCAGCA
TCAGGCCCTGGACCAGCTGGACCTTACACCAGGGAGGCTGGGACACTGGCTTACTATGA
GATCTGCTCCCTCCTGAGCTCTGGAGCCACCCAGGCTTGGGATGAACCCCAGGACGTGC
CCTATGCCTACAAGGAGAGCGAATGGGTTGGCTACGACAACACGAAGAGCTTCAGTGCA
AAGGTTGACTGGCTGAAGCAGAACAACTTTGGAGGGGCCATGGTTTGGGCCCTCGACAT
AGATGATTTCACTGGCACTTTCTGCCATGAAGGCAAATACCCCCTGATCTCCACCCTGA
AGAAGGGCCTTGGTCTGCAGAACGGTGACTGTGTGCCTCCTGCTGAGCCCCTGCCTCCA
GTCACTGAAGCTCCCACTACCACCAGTGGAAGTGGGAGCAGTGGCTCTGGTGGGAGTGG
TGGCTCTGGTGGGAGTGGTGGCTCTGGTGGGAGCGGCTTCTGCGCCGGCAAAGCCAACG
GCATCTACGCAGACCCAGAGAACAGCAGGAACTTCTACAACTGCTTGAACGGCCAAACC
TTTGTCCAGAGCTGCCAACAGGGTCTCGTCTTCGACCCCGTCTGCTCCTGCTGCAACTG
GCCA
>Mesitornis_unicolor_1
ATGGCCAAGCTCACTCTCCTTCTCTATTTCTCTGCAGGCACTGCCTATATCCTGTCATG
TTACTTCACCAACTGGGCCCAGTACAGGCCTGGTGTGGGGAAGTACATGCCTGACAACA
TCGACCCGTGCCTGTGCGACCATCTGATCTACGCCTTTGCTGGGATGAACAACAATGAG
ATCGCAACTTACGAGTGGGACGACGAGACCCTTTACAAATCCTTCAATGCACTGCGCTT
TATGAACAAAAATCTGAAGACTCTTCTGGCAATTGGAGGTTGGAATTTCGGGACATCCA
AGTTCTCCACAATGGTTTCCACTCCCCAGAACCGCCAGACCTTCATCAAGTCCGTCATC
AAGTTCCTGCGCCAGTATCAGTTTGATGGGCTGGACATCGACTGGGAATACCCTGGCTC
CAGGGGCAGCCCAGCCCAGGACAAGGGGCTTTTTACCACCCTGGTTAAGGAAATGCTGG
AAGCCTTTGAGCAGGAAGCTAAGCAGGTCAACAAGCCCCGTCTCCTGATCACCGCTGCT
GTTGCTGCAGGACTTTCCACCATTCAGTCTGGCTATGAGATTCCTGAGCTCGGAAAGTA
CTTGGACTACTTCCATGTGATGACTTACGACTTCCACGGCTCCTGGGATGGACGCACTG
GGGAGAACAGCCCTCTGTACCAAGGCCCAGCTGACACTGGTGACCTCGTCAACTTCAAT
GTTGATTATGCTATGAACTACTGGAAGAACAACGGTGCCCCAGCTGAGAAGCTCCTCGT
TGGCTTCCCAACCTATGGACATAACTTCAACCTCCAAAACCCATCTGACACCGCTGTTG
GAGCACCCGCATCAGGACCTGGGCCAGCTGGACCTTACACACGGCAGTCTGGATTCTTG
GCTTACTACGAGATCTGCACCTTCCTGAACTCTGGAGCTACCCAGGCTTGGGATGCCCC
CCAGGACGTGCCCTATGCTTACAAAGGCAACGAGTGGGTTGGCTATGACAACATCAAGA
GCTTCAACATCAAGATTGACTGGCTGAAGAAGAACAATTTTGGAGGCGCTATGGTCTGG
TCCCTCGATATGGATGACTTCACTGGCACTTTCTGCAAGGAAGGCAAATATCCCCTGAT
CACCACTCTGAAGAACGGCCTTGGTCGGCAAAACAGCGATTGCGTGCCTCCCGCTCAGC
CCAACCCTCCCATCACTGATGCTCCTCCCAGCCAAGGTGGAAGTGGCAGCGGGAGCTCA
GGCAGCAATCCTGGGGGCTCTGGTGGGAGCGGTTTCTGTGCTGGCAAGTCCAACGGCAT
CTACGCAGACCCCACCAACAAGAGCAACTTCTACAGCTGCGTCAATGGCCAAACCTACA
TGGAGAGCTGCCAGTCCGGTCTCGTCTTTGACAGCAGCTGCTCCTGCTGCAACTGGCCA
>Mesitornis_unicolor_2
ATGGCCAAGCTCCTTTTGCTTACCGGTCTGGCCCTCCTGCTGNNNNTCTCTGCAGGCAC
TGCCTATGTGCTGTCATGTTACTTCAGCAACTGGGCCCAGTACAGGCCTGGTGTGGGGA
AATTCATGCCTGACAACATCGACCCGTGCCTGTGCACTCACCTGCTCTACGCCTTTGCT
GGGATGAGCAACAATAAGATCACGACTTACGAGTGGAACGACGAGACCCTTTACCAATC
CTTCAATGGCCTGAAGAACCAGAACAAAAATCTGAAGACTCTGCTCTCCATCGGAGGAT
GGAATTTCGGGACAGATAAGTTCTCCACAATGGTTTCCACTCCCCAGAACCGCCAGACC
TTCATCAAGTCCGTCATCAAGTTCCTGCGCCAGTATCAGTTTGATGGGCTGGACATCGA
CTGGGAATACCCTGGCTCCAGGGGCAGCCCAGCCCGGGACAAGGAGCTCTTTACCGTCC

TGCTCAAGGAAATGCTGGCAGCTTTTGAGCAGGAAGCCAAACAGGTCAACAAGCCCCGT
CTCCTGATCACCGCAGCCGTTTCCGCAGGACTTTCCACCATTCAGAGCGCCTACCAGAT
TCCTGAGGTTGGAAAGTACTTGGACTACATCCACGTGATGACTTACGACTTCCACGGCT
CCTGGGACAGTAGCACTGGGGAGAACAGCCCCCTGTACAAAGGCCCAACCGACACCGGG
GACAACATCTACTTCAATGTTGATTATGCTATGAATTACTGGAAGAGCAATGGTGCCCC
AGCTGAGAAACTCGTTGTTGGATTCCCAGCGTATGGAAATACCTTCAGGCTGCAAAACC
CATCTGACCACGGTCTCGGGGCACCGGTGTCAGGACCTGGTGCAGCTGGACCTTACACA
CAGGAGGCTGGGACACTGGCTTACTATGAGATCTGCAGCCTCTTGAGCTCAGGAGGGAC
CGAGGTTTGGGATGCCCCCCAGGACGTGCCCTATGCTTACAAAGGCAACGAGTGGGTTG
GCTATGACAACATCAAGAGCTACAACATCAAGGTTGACTGGCTGAAGAAGAACAATTTT
GGAGGTGCTATGGTTTGGGCCATTGACTTGGATGACTTCACTGGCACTTTCTGCAAGGA
AGGCAAATATCCCCTGATCACCACCCTGAAGAACGGCCTTGGTCTGCAGAACAGCGACT
GCGTGCCTCCCGCTCATCCTAATCCTCCAGTCACCCCAACTGCCTCTTCCACAGGAGGA
AGTGATAGTGGGAGCGGGGACTCAGGTAGCAATCCAGGGGACTCTGGTGACTCTGGTGA
CTCTGGTGACAGTGACTTCTGTTCTGGCAAGTCCAACGGCATCTACGCAGACCCCACCA
ACAAGAGCAACTTCTACAGCTGCGTCAATGGCCAAACCTACATGGAGAGCTGCCAGTCC
GGTCTCGTCTTTGACAGCAGCTGCTCCTGCTGCAACTGGCCA
>Grus_americana_1
ATGAGAGGAGTGTATAAAAGTGGGAGACGGTCTGCACCAGATCAGCCCTGGTCAAAAAT
GGCCAAGCTCACTTTGCTTACCGGTCTGGCCCTCCTGCTGAACGCCCAGATAGGCACTG
CCTATGTGCTGTCATGTTACTTCACCAACTGGGCCCAGTATAGGCCTGGCCTGGGAAAA
TTCACGCCAGACAACATCGACCCGTGCCTGTGCGACCATCTGATCTACGCCTTTGCTGG
GATGTCCAACAATGAGATCACAACTTACGAATGGAACGATGAGACCCTTTACAAGACCT
TCAACGGCTTGAAGAACCAGAATGGAAAACTAAAGACCCTCCTGGCAATTGGAGGATGG
AATTTCGGCACAGCCAAGTTCTCCACAATGGTTTCCACTCCCGAGAACCGCCAGACCTT
CATCAAGTCTGTCATCAAATTCCTGCGCCAGTATCAATTTGATGGGCTGGACCTTGACT
GGGAATACCCTGGGTCCAGGGGCAGCCCAGCCCAGGACAAGGCTCTCTTTACCGTCCTG
GTTAAGGAAATGCTGGCAGCCTTTGAGCAGGAAGCCAAACAGGTCAACAAGCCCCGTCT
CATGGTCACCGCGGCTGTTGCTGCAGGACTTTCCAACATTCAGGCTGGCTACGAGATTC
CTGAGCTTGGAAAGTACTTGGACTACATCCATGTGATGACTTATGACTTCTACAGCTCT
GGGGATGGACACACAGGGGAGAACAGCCCTCTGTACAATGGCGGTAACAGCTACCTCAG
TGTTGATTATGCTATGAATTATTGGAAGAACAATGGTGCCCCAGCTGAAAAGCTCCTTG
TTGGATTCCCAACCTATGGACATAACTTCAACCTCCAAAACCCATCTGACACTGCTGTT
GGGGCACCAACATCAGGACCTGGGCCAGCTGGACCTTACACAAAGCAGGCTGGATTCTT
GGCTTACTACGAGATCTGCACGTTCCTGGACTCTGGAGCCACTCAGGCTTGGGATGCCC
CCCAAGACGTGCCCTACGCTTACAAAGGCAATGAATGGGTTGGCTACGACAACATCAAG
AGCTTCAACATCAAGGTTGACTGGCTGAAGAAAAACAATTTTGGAGGTGCTATGGTTTG
GACCATTGATCTGGATGACTTCACTGGCACTTTCTGCAAGCAGGGCAAATATCCCCTGA
TCACCACCCTGAAGAACAGTCTCGGCCTGCAAAATGGTGGCTGC
>Grus_americana_2
ATGAGAGGAGTGTATAAAAGTGGGAGACGGTCTGCACCAGATCAGCCCTGGTCAAAAAT
GGCCAAGCTCACTTTGCTTACCGGTCTGGCCCTCCTGCTGAACGCCCAGATAGGCACTG
CCTATGTGCTGTCATGTTACTTCACCAACTGGGCCCAGTATAGGCCTGGCCTGGGAAAA
TTCACGCCAGACAACATCGACCCGTGCCTGTGCGACCATCTGATCTACGCCTTTGCTGG
GATGTCCAACAATGAGATCACAACTTACGAATGGAACGATGAGACCCTTTACAAGACCT
TCAACGGCTTGAAGAACCAGAATGGAAAACTAAAGACCCTCCTGGCAATTGGAGGATGG
AATTTCGGCACAGCCAAGTTCTCCACAATGGTTTCCACTCCCGAGAACCGCCAGACCTT
CATCAAGTCCGTCATCAAATTCCTGCGCCAGTATCAATTTGATGGGCTGGACCTTGACT
GGGAATACCCTGGGTCCAGGGGCAGCCCAGCCCAGGACAAGGCTCTCTTTACCGTCCTG
GTTAAGGAAATGCTGGCAGCCTTTGAGCAGGAAGCCAAACAGGTCAACAAGCCCCGTCT
CATGGTCACCGCGGCTGTTGCTGCAGGACTTTCCAACATTCAGGCTGGCTACGAGATTC
CTGAGCTTGGAAAGTACTTGGACTACTTCCATGTGATGACTTATGACTTCCACGGCTCC
TGGGATGGACACACAGGGGAGAACAGCCCTCTGTATGAAGGCCCAGCTGACACTGGTGA
CCTTGTCTACTTCAATGTTGATTATGCTATGAATTATTGGAAGAACAATGGTGCCCCAG

CTGAAAAGCTCCTTGTTGGATTCCCAACCTATGGACATAACTTCAACCTCCAAAACCCA
TCTGACACTGCTGTTGGGGCACCAACATCAGGACCTGGGCCAGCTGGACCTTACACAAA
GCAGGCTGGATTCTTGGCTTACTACGAGATCTGCACGTTCCTGGACTCTGGAGCCACTC
AGGCTTGGGATGCCCCCCAAGACGTGCCCTACGCTTACAAAGGCAATGAATGGGTTGGC
TACGACAACATCAAGAGCTTCAACATCAAGGTTGACTGGCTGAAGAAAAACAATTTTGG
AGGTGCTATGGTTTGGGCCCTTGATATGGATGACTTCACTGGCACTTTCTGCAAGCAGG
GCAAATATCCCCTGATCACCACCCTGAAGAACGGTCTTGGTCTTCAAAATGGCGACTGC
GTGCCTCCAGCTCAGCCCAACCCTCCAGTCACTGAAGCTCCTAGCCAAGGAAGTGGAAG
TGGGAGCGGGGGCTCAGGGAGCAATCCTGGTGGCTCTGGTGGGAGCAGCTTCTGTGCTG
GGAAGGCCAACGGCATCTATGCAGATCCAACCAACAAGAGCAACTTCTACAACTGCATG
AATGGTGAAACCTTCGTGCAGAGCTGCCAGGACGGCCTCGTCTTTGATACCAGCTGCTC
CTGCTGCAACTGGCCA

>Egretta_garzetta
ATGGCCAACTTCATTTTGCTTACCGGTCTGGCCCTCCTGCTGACCGCCCAGATAGGCAC
TGCCTATGTGCTGTCATGTTACTTCACCAACTGGGCCCAATATAGGCCCGGCCTTGGAA
AATTCATGCCAGACAATATCGACCCATGCCTGTGTGACCATCTGATCTACGCCTTTGCT
GGGATGTCCAACAATAAGATCACAACTTACGAATGGAACGACGAGACCCTTTACAAATC
CTTCAATGCCTTGAAGAACCAGAATGGAAAACTGAAGACCCTCCTGGCAATTGGAGGAT
GGAATTTTGGGACAGCCAAGTTCTCCACAATGGTTTCCACTCCCGAGAATCGCCAGACC
TTCATCAATTCCGTCATCAGCTTCCTGCGCCAGAATCAATTTGATGGGCTGGACATTGA
CTGGGAATACCCTGGGTCCAGGGGCAGCCCACCCCAGGACAAGACTCTCTTTACTGTCC
TGGTTAAGGAAATGCTGGCAGCCTTTGAGCAGGAAGCCCAACAGGTCAACAAGCCTCGT
CTCATGATCACCGCAGCTGTTGCTGCAGGACTTTCCAACATTCAGGCTGGCTACCAGAT
TGCTGAGCTTGGAAAGTACTTGGACTACTTCCATGTGATGACTTATGACTTCCATACCT
CTGGGGAAGGGTTCACTGGGGAGAATAGCCCTCTATACGAAGGCCCAGCTGACACTGGT
GACTTCATCTACTTCAATGTCGATTATGCTATGAACTATTGGAAGAGCAATGGTGCTCC
AGCTGAGAAGCTCCTTGTTGGATTCCCAACCTATGGGCATAACTTCAACCTCCAAAACC
CATCTAACACTGCTGTTGGGGCACCAACATCAGGACCTGGGCCAGCTGGACCTTACACA
AGGCAGGCTGGATTCTTGGCTTACTACGAGATCTGCACATTCCTCAACTCTGGAGCCAC
CCAGGCTTGGGATGCCCCCCAGGATGTGCCCTATGCTTACGAAGGCAGCGAATGGGTTG
GCTATGACAACATCAAGAGCTTCAACATCAAGGTTGACTGGCTGAAGAAGAACAATTTT
GGAGGTGCTATGGTTTGGGCCCTTGATATGGATGACTTCACTGGCACTTTCTGCAAGGA
AGGCAAATATCCCCTGATCACCACCCTGAAGAACAGTCTTGGTCTGCAGAGTGGCGACT
GTGTGCCTCCAGCTCAGCCCAATCCTCCAATCACTGAAGCTCCTAACCAAGGAAGCGGA
AGTGGGAGCGGGGGCTCAGGTGGCAATCCTGGTGGCTCTGGTGGGAGTGGTTTCTGTGC
TGGCAAGGCCAACGGCATCTATGCAGATCCAACCAACAAGAGCAACTTCTACAACTGCC
TTAATGGTGAAACCTTCGTGGAGAGCTGCCAGAGCAGCCTCGTCTTTGATACCAGCTGC
TCTTGCTGCAACTGGCCA

>Dromaius_novaehollandiae
ATGAGAGGGCTGTATAAAACTGAGAAACAGGCAGCACCAAATCAGTCCCAGGCAGAGAT
GGCCAAGCTCACTTTGCTCACCGGTCTGGCACTCCTGCTGAACGCCCAGACAGGCACTG
CCTACGTGCTGTCATGTTACTTCACCAACTGGGCCCAGTATAGGCCTGGCCTGGGAAGC
TACAAGCCTGAGAACATTGACCCCTGCCTGTGCAATCATCTGATCTACGCCTTCGCTGG
CATGTCCAACAATGAGATCACCACCACTGAATGGAATGATGTGACCCTTTACAAATCCT
TCAATGGCTTGAAAAAACAGAATGGAAATCTGAAGACCCTCCTGGCTATTGGAGGATGG
AATTTCGGAACAGCCAAGTTCTCAGCAATGGTTTCCACTGCCGAGAACCGCCAGACCTT
CATCAAATCAGTCATCAAATTCCTGCGCCAGTATGAGTTCGATGGGCTAGACATTGACT
GGGAATACCCTGGGTCTAGAGGCAGCCCAGCTGAGGACAAGGCTCTCTTTACTGTTCTT
GTGAAGGAAATGCTGGCAGCCTTTGAGCAGGAAGCTAAAGAGGTTAACAAGCCCAGGCT
CCTCATCACCGCTGCTGTTGCTGCAGGACTTTCCAACATTGAGTCTGGCTACCAGATTG
CTGAGCTTGGAAACTACCTGGACTACTTCCATGTGATGACATATGACTTCCATGGTTCA
TGGGCTGGACAAACTGGAGAGAACAGCCCTCTGTACACAGGCTCAGCTGACAGTGGCAG

CAACAGTTACCTCAGTGTCGATTATGCTATGAACTATTGGAAGAGCAATGGTGCCCCAG
CTGAGAAGCTCCTTGTTGGTTTCCCAACCTATGGACACAACTTCGACCTCCAAAACCCA
TCTGACACTGCTGTTGGGGCACCAACATCAGGACCTGGGCCTGCTGGAACTTACACAAG
AGAGGCTGGCACCTTGGCTTACTACGAGATCTGCACGTTCCTGGACTCCGGAGCCACCC
AGGCTTGGGATACCCCTCAGGATGTGCCCTATGCCTACAAGGACAGCACATGGGTGGGC
TATGACAACATCAAGAGCTTCAACATCAAGGCTGACTGGCTGAAGAGCAACAACTATGG
CGGTGCTATGGTATGGTCCCTTGCTATGGACGACTTCACTGGCTCTTTCTGCAAACAGG
GGAAATATCCCCTGATCTCCACCCTGAAGGCGGCTCTTGGCCTGCAAAGCGACAGCTGT
GCTGCCTCTTCTCAATCCAGTTCTGCAATCCCTGAGGCTCCTAGCTCTGGAAGTGGGAG
TGCAAGTGGGAGCGGGAGCTCAGGTAGTGGCTCCACTGCAAGCGGCTTCTGTGCTGGCA
AGGCCAACGGCATCTATGCAGATCCATCCAGCAAGAGAAGCTTCTACAACTGCAGTGAC
GGTGAAACCTACCGGGATAGCTGCCAGGCTGACCTCGTCTTTGATGCCAGCTGCTCCTG
CTGCAACTGGCCA
>Apteryx_australis_1
ATGGCCAAGCTCATTTTGCTCACCGGTCTGGCACTCCTGCTGAACGCCCAGATAGGCAC
TGCCTATGTGCTGTCATGTTACTTCACCAACTGGGCCCAGTACAGGCCTGGCCTGGGGC
GCTACAAGCCTGAGAACATTGACCCCTGCCTGTGCAATCATCTGATCTATGCCTTCGCT
GGGATGTCCAACAATGAGATCACGACCTATGAATGGAATGATGAGACCCTTTATAAGTC
CTTCAATGGCTTGAAAAACCAGAATGGAAATCTGAAGACCCTCCTGGCTATTGGAGGAT
GGAATTTCGGAACAGCCAAGTTCTCAGCAATGGTTTCCACTCCCGAGAACCGCCAGACC
TTCATCAAATCGGTCATCAAATTCCTGCGCCAGTATGAATTCGATGGGCTTGACATTGA
CTGGGAATACCCTGGGTCCAGGGGCAGCCCATCTCAGGACAAGGCTCTCTTTACTGTTC
TCGTCAAGGAAATGCTGGCAGCCTTTGAGCAGGAAGCCAAACAGGTTAACAAGCCCAGG
CTCCTCATCACCGCTGCTGTTGCTGCAGGACTTTCCAACATTCAGGCTGGCTACGAGAT
TGCTGAGCTTGGAAACTACCTGGACTACTTCCACGTGATGACATATGACTTCCATGGTT
CATGGGATGGATACACCGGAGAGAACAGCCCTCTGTACAAAGGCCCAGCTGACACTGGC
AGCAACATCTACTTCAATGTCGATTATGCTATGAGCTACTGGAAGAGCAATGGTGCCCC
AGCTGAGAAGCTCCTTGTTGGGTTCCCAACCTACGGACACAACTTCAACCTCCAAAACC
CATCTAACACTGCTGTTGGGGCACCAATATCAGGACCTGGGCCTGCTGGACCTTACACA
AGACAGGCTGGGACCTTGGCTTACTACGAGATCTGCACGTTCCTGGATTCTGGAGCCAC
CCAGGCTTGGGATGCTCCCCAGGACGTACCCTATGCCTACAAGGGCAGCACATGGGTTG
GCTATGACAACATCAAGAGCTTCAACATCAAGGCTGACTGGCTGAAGAAGAACAACTAT
GGAGGTGCTATGGTATGGTCCCTTGCTATGGACGACTTCACTGGCTCTTTCTGCAAGGA
GGGCAAATATCCCCTGATCTCCACCCTGAAGAAGGCTCTTGGCCTGCAAAGTGACGGCT
GTGTGCCCCCTTCTCACCCCAATCCTCCAATCACTGAGGCTCCTAGCTCTGGAAGTGGG
AGCGGAAGCTCAGGGAGCAATCCCGGTGGCTCCACTGGAAGTGGCTTCTGTGCTGGCAA
GGCCAACGGCATCTATCCAGACCCATCCAACAAGAACAGCTTCTACAACTGCGATAACG
GCGAGACCTACCTGGAGAGTTGCCAGGCCGGCCTCGTCTTTGATACCAGCTGCTCCTGC
TGCAACTGGCCA
>Apteryx_australis_2
ATGGCCAAGCTCATTTTGCTCACCGGTCTGGCACTCCTGCTGAACGCCCAGATAGGCAC
TGCCTATGTGCTGTCATGTTACTTCACCAACTGGGCCCAGTACAGGCCTGGCCTGGGGC
GCTACAAGCCTGAGAACATTGACCCCTGCCTGTGCAATCATCTGATCTATGCCTTCGCT
GGGATGTCCAACAATGAGATCACGACCTATGAATGGAATGATGAGACCCTTTATAAGTC
CTTCAATGGCTTGAAAAACCAGAATGGAAATCTGAAGACCCTCCTGGCTATTGGAGGAT
GGAATTTCGGAACAGCCAAGTTCTCAGCAATGGTTTCCACTCCCGAGAACCGCCAGACC
TTCATCAAATCGGTCATCAAATTCCTGCGCCAGTATGAATTCGATGGGCTTGACATTGA
CTGGGAATACCCTGGGTCCAGGGGCAGCCCATCTCAGGACAAGGCTCTCTTTACTGTTC
TCGTCAAGGAAATGCTGGCAGCCTTTGAGCAGGAAGCCAAACAGGTTAACAAGCCCAGG
CTCCTCATCACCGCTGCGGTTGCTGCAGGACTTTCCAACATTCAGGCTGGCTACGAGAT
TGCTGAGCTTGGAAACTACCTGGACTACTTCCACGTGATGACATATGACTTCCATGGTT
CATGGGATGGATACACCGGAGAGAACAGCCCTCTGTACAAAGGCCCAGCTGACACTGGC
AGCAACATCTACTTCAATGTCGATTATGCTATGAACTATTGGAAGAGCAATGGCGCCCC
AGCTGAGAAGCTTGTTGTTGGATTCCCAACCTATGGAAACACCTTCACACTGCAAAACC

CATCCAACACTGCTCTTGGTGCTCCTACATCAGGACCTGGGCCTGCTGGACCTTACACA
CAAGAGGCTGGGACCCTGGCTTACTATGAGATCTGCACTCTGTTGAATTCTGGAGCTAC
CCAGGTTTGGGATGCTCCCCAGGACGTGCCCTATGCCTACAAGGGCAGCACATGGGTTG
GCTATGACAACATCAAGAGCTTCAACATCAAGGCTGACTGGCTGAAGAAGAACAACTAT
GGAGGTGCTATGGTATGGTCCCTTGCTATGGACGACTTCACTGGCTCTTTCTGCAAGGA
GGGCAAATATCCCCTGATCTCCACCCTGAAGAAGGCTCTTGGCCTGCAAAGTGACGGCT
GTGTGCCCCCTTCTCACCCCAATCCTCCAGTCACTGCAGCTCCCAGTACTACAAGTGGA
AGTGGGAGCGGAAGCTCAGGGAGCAATCCTGGTGGCTCAGGGGTGAGTGACTTTTGCAC
TGGCAAGGCCAACGGCATCTATCCAGACCCATCCAGCAAGAACAGCTTCTACAACTGCA
ATAACGGCGAAACCTATTTCGAGAGTTGCCAGGCCGGCCTCGTCTTTGATACCAGCTGC
TCCTGCTGCAACTGGCCA
>Calidris_pugnax_2
ATGGCCAAGCTCACCCTGCTGACCGGTCTGGTTCTCCTGCTGAACGCCCAGATAGGCAC
TGCCTATGTGCTGTCATGTTACTTCACCAACTGGGCCCAGTACAGGCCTGGCCTGGGAA
AGTTCATGCCAGATGACATCGACCCCTGCCTGTGCGACCATCTGATCTACGCCTTTGCT
GGGATGAACAACAACGAGATCACCACTTACGAATGGAACGACGAGACCCTTTACAAATC
CTTCAACGGCCTGAAGAACCAGAACGGAAAACTGAAGACCCTCCTGGCGATTGGAGGAT
GGAATTTCGGGACAGCCAAGTTCACCACAATGGTGTCCACACCCGCAAACCGCCAGACC
TTCATCAAGTCCGTCATCAAATTCCTGCGCCAGTATCAGTTTGATGGGCTGGACCTTGA
CTGGGAATACCCTGGGTCCAGGGGCAGCCCAGCCCAGGACAAGGCTCTCTTTACCGTCC
TCGTTAAGGAACTGGTGGCAGCCTTTGAGCAGGAAGCCAAACAGTCCAACAAGCCCCGT
CTCATGGTCACCGCTGCTGTTGCTGGAGGTCGTTCCACCATTGAGGCTGGCTACGAGAT
TCCTGAGCTTGGAAAGTACCTGGACTACATCCACGTGATGACTTATGACTTCCACGGCT
CTTGGGATGGAGTCACTGGTGAGAACAGCCCTCTGTACGAAGGCCCAGTTGACACCGGG
GACCTCATCTACTTCAATGTCGATTTTGCTATGAACTACTGGAAGAACAATGGCGCCCC
AGCCGAGAAGCTCCTTGTTGGATTTGCAACCTACGGACATAACTACATCCTCCAAAACC
CATCTAACACCGCTGTTGGGGCACCAGCAACTGGACCTGGGCCAGCTGGACCTTACACA
AGGCAGGCTGGATTCTTGGCTTACTACGAGATCTGCACATTCCTGGCCAATGGAGCCAC
CCAGGCTTGGGATGCCCCCCAGGACGTGCCCTACGCTTACAAAGGCAGCGAATGGGTTG
GCTATGACAACATCAAGAGCTACAACATCAAGGTTGACTGGCTGAAGAAGAACAAATTT
GGAGGTGCTATGGTTTGGGCCATTGATATGGATGACTTCACTGGCACTTTCTGCAAGGA
AGGCAAATATCCCCTCATCACCACCCTGAAGAACGGTCTGGGCCTGCAGAACAGTGACT
GTGTGCCTCCAGCTCATCCCAATCCTCCCATCACTGAAGCTCCTAGCCAAGGAGGTGGC
AGTGGCAGCGGCGGCTCTGGTGGTGGCTCTGGTGGCAGCGGCTTCTGTGCTGGCAAGGC
CAACGGCATCTATGCAGATCCAACCAACAAGAGCAACTTCTACAACTGCATTAATGGGG
AAACCTTCATGCAGACCTGCCAGGCCGGCCTCGTCTTCGACGCCAGCTGCTCCTGCTGC
AACTGGCCA
>Calidris_pugnax_1
ATGGCCAAGCTCACCCTGCTGACCGGTCTGGTTCTCCTGCTGAATGCCCAGATAGGCAC
TGCCTATGTGCTGTCATGTTACTTCACCAACTGGGCCCAGTACAGGCCTGGCCTGGGAA
AGTTCATGCCAGATGACATCGACCCCTGCCTGTGCGACCATCTGATCTACGCCTTTGCT
GGGATGAACAACAACGAGATCACCACTTACGAATGGAACGACGAGACCCTTTACAAATC
CTTCAACGGCCTGAAGAACCAGAACAGAAATCTGAAGACCCTCCTGGCGATTGGAGGAT
GGAATTTCGGGACAGCCAAGTTCACCACAATGGTGTCCACACCCGCAAACCGCCAGACC
TTCATCAAGTCCGTCATCAAATTCCTGCGCCAGCATCAGTTTGATGGGCTGGACCTTGA
CTGGGAATACCCTGGGTCCAGGGGCAGCCCAGCCCAGGACAAGGCTCTCTTTACCGTCC
TCGTTAAGGAACTGGTGGCAGCCTTTGAGCAGGAAGCCAAACAGTCCAACAAGCCCCGT
CTCATGGTCACCGCTGCTGTTGCTGCAGGAATTTCCACCGTTCAGGCTGGCTACGAGAT
TCCTGAGCTTGGAAAGTACCTGGACTACATCCACGTGATGACTTATGACTTCCACAGCT
CTTGGGATAGAGTCACTGGTGAGAACAGCCCTCTGTACGATGGCAGTCACAGCCAACTC
AGTGTTGAATATGCTATGAACTATTGGAAGAACAATGGTGCTCCATCTAAGAAGCTCCT
TGTTGGATTCCCAACCTATGGACGTAACTTCAACCTCCAAAACCCATCTAACACCGCTG
TTGGGGCACCAGCAACTGGACCTGGGCCAGCTGGACCTTACACAAAGGAAGCTGGATTC
TTGGCTTACTACGAGATCTGCACATTCCTGGACAATGGAGCCACCCAGGCTTGGGATGC

TGCTGAGGACGTGCCCTACGCTTACAAAGGCAGTGAATGGGTTGGCTATGACAACATCA
AGAGCTTCAACATCAAGGTTGACTGGCTGAAGAAGGGCAATTTTGGGGGTGCTATGGTT
TGGGCCATCGATCTGGATGATTTCACTGGCACTTTCTGCAAGCAGGGAAAATATCCCCT
GATCACCACCCTGAAGAATGGTCTTGGTCTGCAAAGCAGTGGCTGCAGGGCCAAT
>Calidris_pugnax_3
ATGGCCAAGCTCACCCTGCTGACCGGTCTGGTTCTCCTGCTGAACGCCCAGATAGGCAC
TGCCTATGTGCTGTCATGTTACTTCACCAACTGGGCCCAGTACAGGCCTGGCCTGGGAA
AATTCATGCCAGATGACATCGACCCCTGCCTGTGCACCCACCTACTATACGCCTTTGCT
GGGATGAACAACAACGAGATCACCACTTACGAATGGAACGACGAGACCCTTTACAAATC
CTTCAACGGCCTGAAGAACCAGAACAGCAATCTGAAGACTTTGCTTTCTATAGGAGGAT
GGAATTTTGGGTCAGACAAGTTCACCACAATGGTGTCCACACCCAGGAACCGCCAGACC
TTCATCAAGTCCGTCATCAAATTCCTGCGCCAGTATCAGTTTGATGGGCTGGACCTTGA
CTGGGAATACCCTGGGTCCAGGGGCAGCCCAGCCCAGGACAAGGCTCTCTTTACCGTCC
TCGTTAAGGAACTGGTGGCAGCCTTTGAGCAGGAAGCCAAACAGTCCAACAAGCCCCGT
CTCATGGTCACCGCTGCTGTTGCTGCAGGAGTTTCCAAGATTGAGGCTGGCTACGAGAT
TCCTGAGCTTGGAAAGTACCTGGACTACATCCATGTGATGACTTACGACTTCCATGGCT
CCTGGGACACAAACACCGGGGAGAACAGCCCTCTGTACAAAGGCCCAGCTGACACTGGG
GACTTCATATACTTCAACGTTGATTATGCTATGAACTATTGGAAGAGCCATGGTGCCCC
AGCTGAGAAACTAGTTGTCGGATTCGCAACATATGGGAATACCTTCACACTGCGAAACC
CATCTAACAACGGTCTCGGTGCACCAGCATCAGGACCTGGTCCAGCTGGACATTACACA
CAGGAGGCCGGGACACTGGCTTACTATGAGATCTGCAGTCTTTTGAATTCTGGAGCCAC
CCAGGCTTGGGATGCCCCCCAGGACGTGCCCTACGCTTACAAAGGCAGCGAATGGGTTG
GCTATGACAACATCAAGAGCTTCAACATCAAGGTTGACTGGCTGAAGAAGAACAAATTT
GGAGGTGCTATGGTTTGGGCCATCGATCTGGATGATTTCACTGGCACTTTCTGCAAGCA
GGGAAAATATCCCCTCATCACCACCCTGAAGAACGGTCTGGGCCTGCAGAACAGTGACT
GTGTGTCTCCGAATCAGCCCACTACCACCACCGCTTCTACCACCACCATCGATTCTACC
ACCACCACCTTCTATCTCTGTACCAGTGGTCTAACACATGGCAGTGAGAGCGGCGGCTC
CGGTGGGAGCAGTTTCTGTGCTGGCAAGGCCAACGGCATCTATGCAGATCCAACCAACA
AGCGCAACTTCTACAACTGCATTAATGGGGAAACCTTCATGCAGAGTTGTGATAATGGC
CTCGTCTTTGACACCAGCTGCTCCTGCTGCAACTGGCCA
>Gymnogyps_californianus
ATGGCCAAGCTCACTTTGCTTACTGGTCTGGTTCTCCTGCTGAACGCCCAGATAGGCAC
TGCCTATGTGCTGTCATGTTACTTCACCAACTGGGCCCAGTATAGGCCTGGCCTGGGAA
AATTCATGCCTGACAACATCGACCCGTGCCTGTGCAACCATCTGATCTACGCCTTTGCT
GGGATGTCCAACAATGAGATCACAACTTACGAATGGAACGACGAGATCCTTTACAAATC
CTTCAATGGCTTGAAGAACCAGAACAGAAATCTGAAGACTCTGCTGTCTATCGGAGGAT
GGAATTTCGGGACAGACAAGTTCTCCACAATGGTTTCCACTCCCGAGAACCGCCAGACC
TTCATCAAGTCCGTCATCAAATTCCTGCGCCAGTATCAATTTGATGGGCTGGACATTGA
CTGGGAATACCCTGGGTCCAGGGGCAGCCCACCCCAGGACAAGACTCTCTTTACCGTCC
TGGTTAAGGAAATGTTGGCAGCCTTTGAGCAGGAAGCCAAACAGGTCAGCAAGCCCCGT
CTCATGGTCACTGCAGCTGTTGCTGCAGGACTTTCCAACATTCAGGCTGGCTACCAGAT
TCCTGAGCTTGGAAAGTACTTGGACTACATCCATGTGATGACTTATGACTTCTATGGCT
CCTGGGATGGACAGACTGGGGAGAACAGCCCTCTGTACAGTGGCAGTAACAGCTACCTC
AGTGTTGAATATGCTATGAACTATTGGAAGAATAATGGTGCTCCAGCTGAGAAGCTCCT
TGTTGGATTCCCAACCTATGGACATAACTTCTACCTCCAAAACCCATCTGACACTGCTA
TTGGGGCACCAACATCAGGACCTGGGCCAGCTGGACCTTACACAAAGCAGGCTGGATTC
TTGGCTTACTATGAGATCTGCATGTTCCTGGACTCTGGAGCCACCCAGGCTTGGGATGT
CCCCCAGGACGTGCCCTATGCCTACAAGGGCAATGAATGGGTTGGCTACGACAACATCA
AGAGCTTCAACATCAAGGTTGACTGGCTGAAGAACAATTTTGGAGGTGCTGTGGTTTGG
GCCCTTGATCTGGATGACTTCACTGGCACTTTCTGCAAGGAAGGCAAATATCCCCTGAT
CACCACTCTGAAGAACGGTCTTGGTCTGCAAAATGGCGACTATGTGCCTCCAACTCAGC
CCAGTTCTCCAGTCACTGAAGCTCCTGGTAATACAGCTGGAAGTGGGAGTCAGGGCTCT
GGTGTTAGTGACTTCTGTGCTGGCAAGGCCAATGGCATCTATGCAGATGCAACCAACAA
GAACATCTTCTACAACTGCGCTAATGGCAAAACCTTTGTGCAGAGCTGCAATGACGGTC

TCATCTTTGATACCAGCTGCTCCTGCTGCAACTGGCCA
>Falco_naumanni
ATGGCCAAGCTCACTCTGCTTACCGGTCTGGTCCTCCTGCTGAATGCCCAGATAGGCAC
TGCCTATGTGCTGTCATGTTACTTCACCAACTGGGCCCAGTATAGGCCTGGCCTTGGAA
GATTCATGCCTGACAACATCGACCCGTACCTGTGCAACCACCTGATCTACGCCTTTGCT
GGGATGTCCAACAATCAGATCACGACTTATGAATGGAACGATGAGACCCTTTACAGATC
CTTCAACGGCTTGAAGAACCAGAACAGTAATCTGAAGACCCTCCTGTCGGTTGGAGGAT
GGAATTTTGGGACACAGAAGTTCTCCACAATGGTTTCCACTCCACAGAACCGCCAGACC
TTCATCAATTCCGTCATCAGATTCCTACGCCAGTATCAATTCGATGGACTGGACATTGA
CTGGGAGTACCCTGGGTCCAGGGGCAGCCCAGCCCAGGACAAGACTCTCTTTACCGTCC
TGATTAAGGAAATGCTGGCAGCCTTTGAGCAGGAAGCCGCCCAGGTCAACAAGCCCCGT
CTCATGATCACCGCGGCTGTTGCTGCAGGAATTTCCAACATTCAGGCTGGCTACGAGAT
TCCCGAGATTGGACAGTACTTGGACTACATCCATGTGATGACCTACGACTTCTATGGCT
CCTGGGAAGGATACACTGGGGAGAACAGCCCTCTGTATGGTGGCAGTAATATCGACCTC
AGTGTTGAATATGCTATGAACTACTGGAAGAACAACGGTGCTCCAGCTCAGAAGCTCCT
TGTTGGATTCCCAACCTATGGACATAACTTCAACCTTCAAAACCCATCTAACACTGCTG
TTGGGGCACCAGCATCAGGACCTGGGCCAGCTGGACCTTACACAAGGCAGGCTGGGCTT
TTGGCTTACTATGAGATCTGCACATTCCTGGGCTCTGGAGCCACCCAGGCTTGGGATGC
CCCCCAGGATGTGCCCTATGCCTACAAGAACAGCGAATGGGTTGGCTATGATAACATCA
AGAGCTTCGGCATCAAGGTTGACTGGTTAAAGAACAACAACTTTGGAGGTGCTATGGTT
TGGGCCCTTGATATGGATGACTTCACTGGCTCTTTTTGCAATCAGGGCAAATATCCCCT
GATCACCACCCTGAGTAACGGTCTTGGTCTG
>Falco_rusticolus
ATGGCCAAGCTCACTCTGCTTACCGGTCTGGTCCTCCTGCTGAATGCCCAGATAGGCAC
TGCCTATGTGCTGTCATGTTACTTCACCAACTGGGCCCAGTATAGGCCTGGCCTTGGAA
GATTCATGCCTGACAACATCGACCCGTACCTGTGCAACCACCTGATCTACGCCTTTGCT
GGGATGTCCAACAATCAGATCACGACTTATGAATGGAACGATGAGACCCTTTACAGATC
CTTCAACGGCTTGAAGAACCAGAACAGTAATCTGAAGACCCTCCTGTCGGTTGGAGGAT
GGAATTTTGGGACACAGAAGTTCTCCACAATGGTTTCCACTCCACAGAACCGCCAGACC
TTCATCAATTCCGTCATCAGATTCCTACGCCAGTATCAATTCGATGGACTGGACATTGA
CTGGGAGTACCCTGGGTCCAGGGGCAGCCCAGCCCAGGACAAGACTCTCTTTACCGTCC
TGATTAAGGAAATGCTGGCAGCCTTTGAGCAGGAAGCCGCCCAGGTCAACAAGCCCCGT
CTCATGATCACCGCGGCTGTTGCTGCAGGAATTTCCAACATTCAGGCTGGCTACGACAT
TCCCGAGATCGGACAGTACTTGGACTACATCCATGTGATGACCTACGACTTCTATGGCT
CCTGGGAAGGATACACTGGGGAGAACAGCCCTCTGTATGGTGGCAGTAATACCGACCTC
AGTGTTGAATATGCTATGAACTACTGGAAGAACAACGGTGCTCCAGCTCAGAAGCTCCT
TGTTGGATTCCCAACCTATGGACATAACTTCAACCTTCAAAACCCATCTAACACTGCTG
TTGGGGCACCAGCATCAGGACCTGGGCCAGCTGGACCTTACACAAGGCAGGCTGGGCTT
TTGGCTTACTATGAGATCTGCACATTCCTGGGCTCTGGAGCCACCCAGGCTTGGGATGC
CCCCCAGGATGTGCCCTATGCCTACAAGGGCAGCGAATGGGTTGGCTATGATAACATCA
AGAGCTTCAGCATCAAGGTTGACTGGTTAAAGAACAACAACTTTGGAGGTGCTATGGTT
TGGGCCCTTGATATGGATGACTTCACTGGCTCTTTTTGCAATCAGGGCAAATATCCCCT
GATCACCACCCTGAGTAACGGCCTTGGTCTG
>Falco_cherrug
ATGGCCAAGCTCACTCTGCTTACCGGTCTGGTCCTCCTGCTGAATGCCCAGATAGGCAC
TGCCTATGTGCTGTCATGTTACTTCACCAACTGGGCCCAGTATAGGCCTGGCCTTGGAA
GATTCATGCCTGACAACATCGACCCGTACCTGTGCAACCACCTGATCTACGCCTTTGCT
GGGATGTCCAACAATCAGATCACGACTTATGAATGGAACGATGAGACCCTTTACAGATC
CTTCAACGGCTTGAAGAACCAGAACAGTAATCTGAAGACCCTCCTGTCGGTTGGAGGAT
GGAATTTTGGGACACAGAAGTTCTCCACAATGGTTTCCACTCCACAGAACCGCCAGACC
TTCATCAATTCCGTCATCAGATTCCTACGCCAGTATCAATTCGATGGACTGGACATTGA
CTGGGAGTACCCTGGGTCCAGGGGCAGCCCAGCCCAGGACAAGACTCTCTTTACCGTCC
TGATTAAGGAAATGCTGGCAGCCTTTGAGCAGGAAGCCGCCCAGGTCAACAAGCCCCGT
CTCATGATCACCGCGGCTGTTGCTGCAGGAATTTCCAACATTCAGGCTGGCTACGACAT

TCCCGAGATCGGACAGTACTTGGACTACATCCATGTGATGACCTACGACTTCTATGGCT
CCTGGGAAGGATACACTGGGGAGAACAGCCCTCTGTATGGTGGCAGTAATACCGACCTC
AGTGTTGAATATGCTATGAACTACTGGAAGAACAACGGTGCTCCAGCTCAGAAGCTCCT
TGTTGGATTCCCAACCTATGGACATAACTTCAACCTTCAAAACCCATCTAACACTGCTG
TTGGGGCACCAGCATCAGGACCTGGGCCAGCTGGACCTTACACAAGGCAGGCTGGGCTT
TTGGCTTACTATGAGATCTGCACATTCCTGGGCTCTGGAGCCACCCAGGCTTGGGATGC
CCCCCAGGATGTGCCCTATGCCTACAAGGGCAGCGAATGGGTTGGCTATGATAACATCA
AGAGCTTCAGCATCAAGGTTGACTGGTTAAAGAACAACAACTTTGGAGGTGCTATGGTT
TGGGCCCTTGATATGGATGACTTCACTGGCTCTTTTTGCAATCAGGGCAAATATCCCCT
GATCACCACCCTGAGTAACGGCCTTGGTCTG
>Falco_peregrinus
ATGGCCAAGCTCACTCTGCTTACCGGTCTGGTCCTCCTGCTGAATGCCCAGATAGGCAC
TGCCTATGTGCTATCATGTTACTTCACCAACTGGGCCCAGTATAGGCCTGGCCTTGGAA
GATTCATGCCTGACAACATTGACCCGTACCTGTGCAACCACCTGATCTACGCCTTTGCT
GGGATGTCCAACAATCAGATCACGACTTATGAATGGAACGATGAGACCCTTTACAGATC
CTTCAACGGCTTGAAGAACCAGAACAGTAATCTGAAGACCCTCCTGTCGGTTGGAGGAT
GGAATTTTGGGACACAGAAGTTCTCCACAATGGTTTCCACTCCACAGAACCGCCAGACC
TTCATCAATTCCGTCATCAGATTCCTACGCCAGTATCAATTCGATGGACTGGACATTGA
CTGGGAGTACCCTGGGTCCAGGGGCAGCCCAGCCCAGGACAAGACTCTCTTTACCGTCC
TGATTAAGGAAATGCTGGCAGCCTTTGAGCAGGAAGCCGCCCAGGTCAACAAGCCCCGT
CTCATGATCACCGCGGCTGTTGCTGCAGGAATTTCCAACATTCAGGCTGGCTACGAGAT
TCCCGAGATTGGACAGTACTTGGACTACATCCATGTGATGACCTACGACTTCTATGGCT
CCTGGGAAGGATACACTGGGGAGAACAGCCCTCTGTATGGTGGCAGTAATATCGACCTC
AGTGTTGAATATGCTATGAACTACTGGAAGAACAATGGTGCTCCAGCTCAGAAGCTCCT
TGTTGGATTCCCAACCTATGGACATAACTTCAACCTTCAAAACCCATCTAACACTGCTG
TTGGGGCACCAGCATCAGGACCTGGGCCAGCTGGACCTTACACAAGGCAGGCTGGGCTT
TTGGCTTACTATGAGATCTGCACATTCCTGGGCTCTGGAGCCACCCAGGCTTGGGATGC
CCCCCAGGATGTGCCCTATGCCTACAAGGGCAGCGAATGGGTTGGCTATGATAACATCA
AGAGCTTCAGCATCAAGGTTGACTGGTTAAAGAACAACAACTTTGGAGGTGCTATGGTT
TGGGCCCTTGATATGGATGACTTCACTGGCTCTTTTTGCAATCAGGGCAAATATCCCCT
GATCACCACCCTGAGTAACGGCCTTGGTCTG
>Phaethon_lepturus
ATGCCTGACAACATCGACCCGTACCTGTGTGACCATCTGATCTATGCCTTTGCTGGGAT
GTCCAACAATGAGATCACAACTTACGAATGGAATGACGAGACCCTTTACAAATCCTTCA
ACGGCTTGAAGAACCAGTTCTCCACAATGGTTTCCACTCCTGAGAACCGCCAGACCTTC
ATCAAGTCCGTCATCAAATTCCTGCGCCAATATCAATTTGATGGATTGGACATTGACTG
GGAATACCCTGGGGCCAGGGGCAGCCCACACCAGGACAAGACTCTCTTTACCATCCTGG
TTAAGGAAATGCTGGCAGCCTTTGAGCAGGAAGCTGAACAGGTCAACAAGCCCCGTCTC
ATGCTCACCGCTGCTGTTGCTGCAGGACTTTCCACCATTCAGGCTGGTTACCAGATACC
TGAGCTTGGAAAGTACTTGGACTACTTCCATGTCATGACTTATGACTTCCACGCCTCCT
GGGATGGAAGCACTGGGGAGAACAGCCCTCTGTACGAAGGCCCAGCTGACACTGGTGAC
CTCATCTACTTCAATGTCGATTATGCTATGAACTACTGGAAGAGCAATGGTGCCCCAGC
TGAGAAGCTCCTTGTTGGATTTCCAACCTATGGACATACCTTCAACCTCCAAAACCCAT
CTGACACTGCTGTTGGGGCACCAGCATCAGGACCTGGGCCAGCTGGACCTTACACAAAG
CAGGCTGGATTCTTGGCTTACTATGAGATCTGCACATTCCTGGGCTCGGGAGCCACACA
GGCATGGGATGCCCCTCAGGATGTGCCCTATGCTTACAAAGGCAACGAATGGGTTGGCT
ATGACAACATCAAGAGCTTCAACATCAAGGTTGACTGGCTGAAGAAGAACAATTTTGGA
GGTGCTATGGTTTGGGCCCTTGATATGGATGACTTCACTGGCACTTTCTGCAAGGAAGG
CAAATATCCCCTGATCACCACCCTAAAGAACGGCCTTGGTCTGCAAAACGGTGACTCCG
TGCCTCCAGCTCAGCCCAGTTCTCCAGTCACTGAAGCTCCCTGTACTACAGGTGGATGT
GGAACTGGAAGCGGGGGCTCTGGTGTTAGTGACTTCTGTGCTGGCAAGGCCAACGGCAT
CTATGCAGATCCAACCAACAAGAGCAGCTTCTACAACTGCATTGATGGTGAAACCTTTG
TGCAGAGCTGCCAGAATGGCCTCGTCTTTGATAGCAGCTGCTCCTGCTGCAACTGGCCA
>Phalacrocorax_carbo

ATGCCGGACAACATCAACCCATACCTGTGCCACCATCTGATCTATGCCTTTGCTGGGAT
GTCCAACAACGAGATCACAACTTACGAATGGAACGACGAGACCCTTTACAAATCCTTCA
ACGCCTTGAAGAACCAGTTCTCCACAATGGTTTCCACACCTGAGAACCGCCAGACTTTC
ATCAACTCTGTCATCAAATTCCTGCGCCAGTATCAATTTGATGGGCTGGACATTGACTG
GGAATACCCTGGGTCCAGGGGCAGCCCACCCCAGGACAAGACGCTTTTTACCGTCCTGG
TTCAGGAAATGCTGGCAGCCTTTGAGCAAGAAGCCAAACAGGTCAACAAGCCCCGTCTC
ATGATCACTGCGGCTGTTGCTGCAGGACTTTCCAACATTCAGGCTGGCTACGAGATTTC
TGAGCTTGGAAAGTACTTGGACTACTTCCATGTGATGACTTACGACTTCCACGGCTCCT
GGGAAGGATACACTGGGGAGAACAGCCCTCTGTTCAAAGGCCCAGCTGACACTGGTGAC
CTCATCTACTTCAATGTCGATTATGCTATGAACTATTGGAAGAGCAATGGTGCCCCAGC
TGAGAAGCTCCTTGTTGGATTCCCGACCTATGGACATGACTTCAACCTCCAAAACCCAT
CTGACACTGCTGTTGGGGCACCAACATCAGGACCTGGCCCAGCTGGACCTTACACAAGG
CAGGCTGGATTCTTGGCCTACTATGAGATCTGCACATTCCTGGACTCTGGAGCCACCCA
GGCTTGGGATGCCCCCCAGGATGTGCCCTACGCTTACAAAGGCAATGAATGGGTTGGCT
ATGACAACATCAAGAGCTTCAACATCAAGGTTGACTGGCTGAAGAAGAACAATTTTGGA
GGTGCTATGGTTTGGGCCCTTGATATGGATGACTTCACTGGCACTTTCTGCAAGGAAGG
CAAATATCCCCTGATCACCGCCCTGAAGAATGGTCTTGGTCTGCAAAACAGTGACCTTG
TGCCTCCAACTCAGTCCAATCCTCCAGTCACTGAAGCTCCTAACCAAGGAAGTGGAAAT
GGGAGCGGGGGTTCAGGTAGCAATACTGGTGGCTCTGGTGGGAGCGGTTTCTGTGCCGG
CAAGGCCAACGGCATCTACGCAGATCCAAGCAGCAAGAGCAACTTCTACAACTGCCTTA
ATGGAGAAACCTTCGTGCAGAGCTGCCAGGAAGGCCTCGTCTTTGATGCCAGCTGCTCC
TGCTGCAACTGGCCA
>Meleagris_gallopavo
ATGGCCAAGCTCATTTTGCTTACCGGTCTGGCCCTCCTGCTGAATGCCCAGATAGGCTC
TGCCTACGTGCTCTCATGCTATTTCACCAACTGGGCTCAATACAGGCCTGGCCTGGGAA
AATATATGCCAGACAACATCGACCCATGCCTCTGTGACCATCTGATCTACGCCTTTGCT
GGGATGTCCAACAATGAGATCACAACTTATGAATGGAATGATGAGACCCTCTACAAGTC
CTTCAACGGACTGAAAAATCAGAATGGAAATCTCAAGACTCTCCTGGCAATTGGAGGAT
GGAATTTTGGAACAGCCAAGTTCTCCACAATGGTTTCCACTCCTGAGAACCGCCAGACC
TTCATCAAGTCCGTCATCAAATTCCTGCGCCAGTACCAATTTGATGGGCTGGACATTGA
CTGGGAATACCCTGGATCAAGGGGCAGCTCTTCTCAGGACAAAGGTCTCTTCACCGTCC
TTGTTCAGGAAATGCTGGCTGCCTTTGAGCAGGAAGCCAAGCAGGTGAACAAGCCCCGT
CTCATGATCACTGCTGCCGTTGCTGCAGGGCTTTCCAACATTCAGGCTGGCTACCAGAT
TGCTGAGCTCGGAAAGTACTTGGACTATTTCCATGTGATGACTTACGATTTCCATGGCT
CCTGGGACGGACAAACAGGGGAGAACAGCCCTCTGTACAAAGGCCCCGCTGATACTGGT
GACCTCATCTATTTCAACGTTGATTATGCTATGAATTACTGGAAAAGCAATGGTGCTCC
AGCCGAGAAACTCCTGGTTGGATTCCCAACCTATGGACATAGCTACATCCTCAAGAATC
CTTCCAACACTGCTGTTGGGGCACCAACATCGGGCCCTGGGCCAGCTGGGCCTTACACA
AGGCAGTCTGGTTTCTTAGCTTACTATGAGATCTGCACATTCCTGGACTCTGGAGCCAC
CCAGGCTTGGGATGCTCCCCAGGATGTGCCCTATGCCTACAAGAGCAGTGAATGGGTTG
GCTATGACAACATCAAGAGCTTCAACATCAAGGTTGATTGGCTGAAGAAGAACAACTAC
GGCGGTGCTATGGTTTGGTCCCTTGATATGGATGACTTCACGGGCACTTTCTGTAAACA
GGGCAAATATCCCCTGATTACTACCCTGAAGAATGCTCTTGGTCTTCAAAGCAACGGCT
GTGTGCCTCCAGCTCAGCCCAATCCTCCCATCACTGCAGCTCCTAGCACTGGAAGTGGA
AGTGGAAGCGGGAGCTCAGGTAGCAGTACTGGCAGCTCAGGTGGGAGTGGATTCTGTGC
TGGCAAGGCCAATGGCATCTATGCAGATCCAACCAACAAGAGCAAATTCTACAACTGCA
ATAATGGCGAAACCTTCGCGCAGTCGTGCCAGGCCGGTCTCGTCTTTGATTCCAGCTGC
TCCTGCTGCAACTGGGCA

>Rissa_tridactyla_1
ATGGCCAAGCTCACTTTGCTTACCGGTCTGGTCTTCCTGCTGAACGCCCAAATAGGCAC
TGCCTATGTGCTGTCATGTTACTTCACCAACTGGGCCCAGTACAGGCCCGGCCTGGGAA
AATTCATGCCCAACAACATCGACCCGTGCCTGTGCGACCATCTGATCTACGCCTTTGCT
GGGATGAACAACAATGAGATCACCACTTACGAATGGAACGACGAGACCCTTTACAAATC

CTTCAATGGCTTGAAGAACCAGAACAGAAATCTGAAGACCCTCCTGGCAATTGGAGGAT
GGAGTTTCGGGACAGCTAAGTTCTCCACAATGGTTTCTACGCCTCAGAACCGCCAGACC
TTCATCAAGTCCGTCATCAAATTCCTGCGCCAGCATCAATTTGATGGGCTGGACCTTGA
CTGGGAATACCCTGGGTCCAGGGGCAGCCCACCTCAGGACAAGACTCTCTTTACCGTCC
TCGTTAAGGAAATGGTGGCAGCCTTTGAGCAGGAAGCCAAACAGGTCAACAAGCCCCGT
CTCATGGTCACCGCTGCTGTTGCTGCAGGAGTTTCCACCATTCAGGCTGGCTACGAGAT
TCCTGAGCTTGGAAAGTACTTGGACTACATCCACGTGATGACTTACGACTTCTACAGCT
CTTGGGATGGACACACTGGGGAGAACAGCCCTCTGCACGGCGGCAGTAACAGCCAACTC
AGTGTTGAATACGCTATGAACTATTGGAAGAACAATGGTGCTCCAGCGGAGAAGCTCCT
TGTTGGATTCCCAACCTATGGACGTAGCTTCAACCTCCAGAACCCATCCAACACTGCTG
TTGGGGCACCAACATCAGGACCAGGGCCAGCTGGACCTTATACAAAGGAGGCTGGATTC
CTGGCTTACTATGAGATCTGCACATTCCTGGACTCTGGAGCCACCCAGGCTTGGGATGC
CCCCCAGGACGTGCCCTACGCTTACAAAGGCAGCGAATGGGTTGGCTATGACAACATCA
AGAGCTTCAACATCAAGGTTGACTGGCTGAAGAAGAACAATTTTGGAGGCGCTATGGTT
TGGACCATCGATCTGGATGACTTCACTGGCACTTTCTGCAAGGAAGGCAAATATCCCCT
GATCACCACCCTGAAGAACGGTCTTGGTCTGCAAAACAGCGGCTGCAGGAGCTAT
>Rissa_tridactyla_2
ATGGCCAAGCTCACTTTGCTTACCGGTCTGGTCTTCCTGCTGAACGCCCAAATAGGCAC
TGCCTATGTGCTGTCATGTTACTTCACCAACTGGGCCCAGTACAGGCCCGGCCTGGGAA
AATTCATGCCCAACAACATCGACCCGTGCCTGTGCGACCATCTGATCTACGCCTTTGCT
GGGATGAACAACAATGAGATCACCACTTACGAATGGAACGACGAGACCCTTTACAAATC
CTTCAATGGCTTGAAGAACCAGAACAGAAATCTGAAGACCCTCCTGGCAATTGGAGGAT
GGAGTTTCGGGACAGCTAAGTTCTCCACAATGGTTTCTACGCCTCAGAACCGCCAGACC
TTCATCAAGTCCGTCATCAAATTCCTGCGCCAGCATCAATTTGATGGGCTGGACCTTGA
TTGGGAATACCCTGGGTCCAGGGGCAGCCCACCTCAGGACAAGACTCTCTTTACCGTCC
TCGTTAAGGAAATGGTGGCAGCCTTTGAGCAGGAAGCCAAACAGGTCAACAAGCCCCGT
CTCATGGTCACCGCTGCTGTTGCTGCAGGAGTTTCCACCATTCAGGCTGGCTACGAGAT
TCCTGAGCTTGGAAAGTACTTGGACTACATCCACGTGATGACTTACGACTTCTACAGCT
CTTGGGATGGACACACTGGGGAGAACAGCCCTCTGCACGGCGGCAGTAACAGCCAACTC
AGTGTTGAATACGCTATGAACTATTGGAAGAACAATGGTGCTCCAGCGGAGAAGCTCCT
TGTTGGATTCCCAACCTATGGACGTAGCTTCAACCTCCAGAACCCATCCAACACTGCTG
TTGGGGCACCAACATCAGGACCAGGGCCAGCTGGACCTTATACAAAGGAGGCTGGATTC
CTGGCTTACTATGAGATCTGCACATTCCTGGACTCTGGAGCCACCCAGGCTTGGGATGC
CCCCCAGGACGTGCCCTACGCTTACAAAGGCAGCGAATGGGTTGGCTATGACAACATCA
AGAGCTTCAACATCAAGGTTGACTGGCTGAAGAAGAACAATTTTGGAGGCGCTATGGTT
TGGACCATCGATCTGGATGACTTCACTGGCACTTTCTGCAAGGAAGGCAAATATCCCCT
GATCACCACCCTGAAGAACGGTCTTGGTCTGCAAAACAGCGACTGTGTGCCTCCAGCTC
AGCCCAATCCTCCAGTCACTGAAGCTCCTAACCAAGGAGGTGGAAGTGGGAGCGGGGGC
TCAGGTGGTGGCTCTGGTGGCGGCTCTGGTGGCAGCGGTTTCTGTGCTGGCAAGGCCAA
CGGCATCTATGCAGATCCAACCAACAAGAGCAACTTCTACAACTGCATTAATGGTGAAA
CTTTCATGCAGAGCTGCCAGAACGGTCTTGTCTTTGATACCAGCTGCTCCTGCTGCAAC
TGGCCA
>Charadrius_vociferus_2
ATGGCCAAGCTCACTTTGCTTACCGGTCTGGTCCTCCTGCTGAACGCTCAGATAGGCAC
TGCCTATGTGCTGTCATGTTACTTCACCAACTGGGCCCAGTATAGGCCTGGCCTGGGAA
AATTCATGCCTGACAACATCGACCCGTGCCTGTGCACTCACCTGCTATATGCCTTTGCT
GGCATGTCCAACAATAAAATTACAACTTACGAATGGAATGACGAGACCCTTTACAAATC
CTTCAACGGCTTGAAGAACCAAAACAGAAACCTGAAGACTCTGCTGTCTATTGGAGGAT
GGAATTTCGGGTCAGACAAGTTCTCCACAATGGTTTCCACGCCCCAGAACCGCCAGACC
TTCATCAAGTCCGTCATCAAGTTCCTGCGCCAGTACCAATTTGATGGGCTTGACCTTGA
CTGGGAATACCCCGGCTCCAGGGGCAGCCCACCCCAGGACAAGGGTCTCTTCACTGTCC
TCGTTAAGGAAATGCTGGCAGCATTTGAGCAGGAAGCCAAACAGGTCAACAAGCCCCGT
CTCATGGTCACTGCTGCTGTTGCTGCTGGAGTTTCCACCATTCAGGCTGGCTACGAGAT
TCCTGAGCTTGGGAAGTACTTGGACTACATCCACGTGATGACTTATGACTTCCACGGCT

CCTGGGATAGAAACACTGGGGAGAACAGCCCTCTGTACAAAGGCCCAGCTGACACCGGG
GACTACGTCTACTTCAATGTTGATTATGCTATGAATTATTGGAAGAGCAATGGTGCCCC
AGCAGAGAAACTAGTTGTTGGATTTCCAGCATACGGAAATACCTTCACACTGCAAAACC
CATCTGACAATGGTCTTGGTGCACCAGCATCAGGCCCTGGGCCAGCTGGACCTTACACA
CAGGAGGCCGGGACACTGGCTTACTATGAGATCTGCACTCTCTTGAATTCTGGAGCCAC
CGAGGTTTGGGATACCCCCCAGGACGTGCCCTACACTTACAAAGGCAAGGAATGGGTTG
GCTATGACAACATCAAGAGCTACAAGATCAAGGTTGACTGGCTGAAGAAGAACAATTTT
GGAGGTGCTATGGTCTGGGCTCTTGATCTGGATGACTTCACTGGCACTTTCTGCAAGGA
AGGCAAATATCCCCTGATCACCACCCTGAAGAACGGACTTGGTCTGCAAAACAGCGACT
GCGTGTCCCCGCCTCAGCCCAATCCTCCCATCACTGAAGCTCCTAACCAGGGAAGTGGC
AGTGGGAGCGGGGGCTCAGGTGGTGGCTCTGGTGGGAGCGGTTTCTGTGCTGGCAAGGC
CAACGGCATCTACGCAGATCCAACCAACAAGAGCAACTTCTACAACTGCGTTAATGGTG
AAACCTTCATGCAGACCTGCCAGAACGGTCTCGTCTTTGATCCCAGCTGCTCCTGCTGC
AACTGGCCA
>Charadrius_vociferus_1
ATGGCCAAGCTCACTTTGCTTACCGGTCTGGCCCTCCTGAAAAAACTGCTGAACGCCCA
GATCAGCACTGCCTATGTGCTGTCATGTTACTTCACCAACTGGGCCCAGTATAGGCCTG
GCCTGGGAAAATTCATGCCTGACAACATCGACCCGTGCCTGTGCGACCATCTGATCTAC
GCCTTTGCTGGCATGTCCAACAATGAGATCACAACTTACGAATGGAACGACGAGACCCT
TTACAAATCCTTCAACGGCTTGAAGAACCAGAACAGAAATCTGAAGACCCTCCTGGCCA
TTGGAGGATGGAATTTCGGGACAGACAAGTTCTCCACAATGGTTTCCACGCCCCAGAAC
CGCCAGACCTTCATCAAGTCCGTCATCAAGTTCCTGCGCCAGCATCAATTTGATGGGCT
GGACCTTGACTGGGAATACCCTGGCTCTAGGGGCAGCCCACCCCAGGACAAGGGTCTCT
TCACTGTCCTCGTTAAGGAAATGCTGGCAGCATTTGAGCAGGAAGCCAAACAGGTCAAC
AAGCCCCGTCTCATGGTCACTGCTGCTGTTGCTGCTGGAGTTTCCACCATTCAGGCTGG
CTACGAGATTCCTGAGCTTGGGAAGTACTTGGACTACATCCACGTGATGACTTATGACT
TCTACAGCGCTTGGGATGGACGCACTGGGGAGAACAGCCCTCTGTACAGTGGTGTTAAC
AGCCACCTCAGTGTTGATTATGCTATGAATTATTGGAAGAACAATGGTGCCCCAGCAGA
GAAGCTCCTTGTTGGATTCCCAACCTATGGACGTAACTTCAACCTCCAAAATCCATCTA
ACACTGCTGTTGGGGCACCAACATCAGGGCCTGGGCCAGCTGGACCTTACACAAAGGAA
GCTGGATTGTTGGCTTACTACGAGATCTGCACATTCCTGGACTCTGGAGCCACCCAGGC
TTGGGATACCCCCCAGGACGTGCCCTACGCTTACAAAGGCAGCGAATGGGTTGGCTATG
ACAACATCAAGAGCTTCAACATCAAGGTTGACTGGCTGAAGAAGAACAATTTTGGAGGT
GCTATGGTTTGGACCATTGATCTGGATGACTTCACTGGCACTTTCTGCAAGCAGGGCAA
ATATCCCCTGATCACCACCCTGAAGAACGGTCTTGGTCTGCAAAGCAGGAACAAAACCA
ACCAGAGCAGCTTTTACCAATGTTTGAACNATCAGAACTTCTTTCAGAATTGTTATGAC
AATCTTGTTTATAATACCAGCTGCTCCTGCTACAACTGGCCA
>Tympanuchus_pallidicinctus_2
ATGGCCAAGCTCGTTTTGCTTACCGGTCTGGCCCTCCTGCTGAACGCCCAGATAGGCTC
TGCCTATGTGCTGTCATGCTATTTCACCAACTGGGCTCAATACAGGCCTGGCTCAGGAC
GCTTTACAGTCGACAACATCGACCCGTGCCTCTGTGACCATCTGATCTACGCCTTCGCT
GGGATGTCCAACAATGAGATCACAACCATTGAATGGAATGATGAGACCCTCTACAAATC
CTTCAATGGACTGAAAAATCAGAATGGAAATCTCAAGACCCTCCTGGCAATTGGAGGAT
GGAATTTTGGGACAGCCAAGTTCTCCACAATGGTTTCCACTCCTGAGAACCGCCAGACC
TTCATCAACTCCGTCATCAAATTCCTGCGCCAGTACCAATTTGATGGGCTGGACATTGA
CTGGGAATACCCTGGATCAAGGGGCAGCTCTTCTCAGGACAAAGCTCTCTTCACCGTCC
TGGTTCAGGAAATGCTGGCTGCCTTTGAGCAGGAAGCCAAGCAGGTGAACAAGCCCCGT
CTCATGATCACGGCTGCTGTTGCTGCAGGACTTTCCAACATTCAGGCTGGCTACCAGAT
TGCTGAGCTCGGAAAGTACTTGGACTACTTTCATGTGATGACTTACGACTTCTACAGCT
CTGGGGATGGACAAACAGGGGAGAACAGCCCTCTGTACAGTGGCAGTAATGTATACCTC
AGTGTTGATTATGCTATGAACTATTGGAAGAGCAATGGTGCTCCAGCTGAGAAACTCCT
GGTTGGATTCCCAACCTATGGTCATAGCTTCAACCTCCAGAATCCATCCAACACTGCTG
TTGGGGCACCAACATCGGGCCCTGGGCCAGCTGGGCCTTACACAAGAGAAGCTGGGTTG
CTGGCTTACTATGAGATCTGCACATTCCTGGACTCTGGGGCCACCCAGGCTTGGGATGC

TCCCCAGGATGTGCCCTATGCCTACAAGAGCAGTGAATGGGTTGGCTATGACAACATCA
AGAGCTTCAACATCAAGGTTGACTGGCTGAAGAAGAACAACTATGGCGGTGCTATGGTT
TGGGCCCTTGATATGGATGACTTCACTGGCACTTTCTGTAAACAGGGCAGATATCCCCT
GATTACCACCCTGAAGAATGCTCTTGGTCTTCAAAGCAGCAGCTGTGTGCCCACAACGC
CCAGTCCTACCACCACCGCAGTTCTCTGTAATACGCAAGGAAGTGAAAGCGGGAGTGAG
AGTGGCAGCTCCGGCAGCAATCCCGACAGCTCAGGTGGGAGTGCATTCTGTGCTGGCAA
GGCCAACGGCATCTACGCAGATCCAACCAACAAGAGCAAGTTCTACAACTGCAATAATG
GCGAAACCTTCACGCAGTCGTGCCAGGCCGGTCTCGTCTTTGATTCCAGCTGCTCCTGC
TGCAACTGGGCA
>Tympanuchus_pallidicinctus_1
ATGGCCAAGCTCGTTTTGCTTACCGGTCTGGCCCTCCTGCTGAACGCCCAGATAGGCTC
TGCCTATGTGCTGTCATGCTATTTCACCAACTGGGCTCAATACAGGCCTGGCCTGGGAA
AATACATGCCAGATAACATCGACCCGTGCCTCTGTGACCATCTGATCTACGCCTTCGCT
GGGATGTCCAACAATGAGATCACAACCATTGAATGGAATGATGAGACCCTCTACAAATC
CTTCAATGGACTGAAAAATCAGAATGGAAATCTCAAGACCCTCCTGGCAATTGGAGGAT
GGAATTTTGGGACAGCCAAGTTCTCCACAATGGTTTCCACTCCTGAGAACCGCCAGACC
TTCATCAACTCCGTCATCAAATTCCTGCGCCAGTACCAATTTGATGGGCTGGACATCGA
CTGGGAATACCCTGGATCAAGGGGCAGCTCTTCTCAGGACAAAGCTCTCTTCACCGTCC
TGGTTCAGGAAATGCTGGCTGCCTTTGAGCAGGAAGCCAAGCAGGTGAACAAGCCCCGT
CTCATGATCACGGCTGCTGTTGCTGCAGGACTTTCCAACATTCAGGCTGGCTACCAGAT
TGCTGAGCTTGGAAAGTACTTGGACTATTTCCATGTGATGACTTATGATTTCCATGGCT
CCTGGGATGGAGAAACAGGGGAGAACAGCCCTCTGTACAAAGGCCCCGCTGACACTGGT
GACCTCATCTACTTCAACGTTGATTATGCTATGAACTATTGGAAGAGCAATGGTGCTCC
AGCTGAGAAACTCCTGGTTGGATTCCCAACCTATGGACATAGCTACATCCTCAAGAATC
CTTCCAACACTGCTGTTGGGGCACCAACATCGGGCCCTGGGCCAGCTGGGCCTTACACA
AGGCAGTCTGGTTTCTTAGCTTACTATGAGGTATGTGTGTTTCATCACACCTGGGCAGC
CAAGTATGGTCTCAAAACTTCCTACAAAGGAATTTTATCCCATGGATTCATGGAATATT
CCAGCCACCCATTTCTATGGGACAGTTAC
>Dryobates_pubescens_1
ATGGCCAAGCTCACTTTGCTCACCGGTCTGGTCCTGCTGCTGAATGCACAGCTAGGCTC
TGCCTATATCCTCTCCTGCTACTTCACCAACTGGGCCCAGTACAGGCCTGGCCTGGGCA
AGTACACCCCTGAGAATGTGGACCCCTGCCTGTGCAACCACCTGATCTATGCCTTCGCT
GGGATGGCCAACAACGAGATCACCACCTACGAGTGGAACGATGAGACCCTCTACAAGTC
CTTCAACGGCCTGAAGAACCAGAACAAGAACCTGAAGACCCTCCTGGCCATCGGGGGAT
GGAACTTCGGGACAGAGAAGTTCTCCACCATGGTCTCCACCCCCCAGAACCGTCAGACC
TTCATCAAGTCTGTCATCAGGTTCCTGAGGCAGTACCAGTTTGATGGGCTGGACCTCGA
CTGGGAGTACCCTGGCTCCAGGGGCAGCCCAGCCCAGGACAAGGCTCTCTTCACCGTGC
TGGTGAAGGAAATGGTGGCAGCCTTTGAGCAGGAAGCCAAAGAGGTCAACAAGCCCAGG
CTCATGGTCACAGCTGCCGTTGCTGCAGGGCTCTCCACCATCCAGGCTGGCTACGAGAT
TGCTGAGCTTGGCAAGTACCTGGACTACATCCACGTCATGACCTACGACTTCTCCAGCG
CCTACGACGGCCGCACGGGGGAGAACAGTCCCCTGCACAACACTGCCAACAGGCAGTTC
AGTGTTGAGTATGCCATGAGCTACTGGAGGGACAGCGGAGCCCCAGCTCAGAAGCTGCT
CGTTGGCTTCCCGACCTATGGCCACAGCTTCACCCTCCAGAACCCTTCCAACACTGCTG
TTGGGGCTCCCACAACAGGGCCTGGCCCAGCTGGACCCTACACTGGGGAGGATGGGCTC
CTGGCTTACTATGAGATCTGCACAATCCTTGACTCTGGAGCCACCCAGGCTTGGGATGC
CTCCCAGGACGTGCCCTATGCCTACAAGGGCAGCCAGTGGGTTGGCTATGACAACACCA
AGAGCTTCAGCCTCAAGGTGGACTGGCTGAAGAAGAACAACTTTGGAGGTGCCATGGTT
TGGACCATTGACCTGGATGACTTCACTGGCACCTTCTGCAAGCAGGGCAAATATCCTCT
GATCAGCACCCTGAAGAATGGCCTGGGGCTGAGGGGCTGCAGCAGCTAC
>Dryobates_pubescens_2
ATGGCCAAGCTCACTTTGCTCACCGGTCTGGTCCTGCTGCTGAACGCCCACATAGGCTC
TGCCTATATCCTCTCCTGCTACTTCACCAACTGGGCCCAGTACAGGCCTGGCCTGGGCA
AGTACACCCCTGAGAATGTGGACCCCTGCCTGTGCAACCACCTGATCTATGCCTTCGCT
GGGATGGCCAACAACGAGATCACCACCTACGAGTGGAACGATGAGACCCTCTACAAGTC

CTTCAACGGCCTGAAGAACCAGAACAAGAACCTGAAGACCCTCCTGGCCATCGGGGGAT
GGAACTTCGGGACAGAGAAGTTCTCCACCATGGTCTCCACCCCCCAGAACCGTCAGACC
TTCATCAAGTCTGTCATCAGGTTCCTGAGGCAGTACCAGTTTGATGGGCTGGACCTCGA
CTGGGAGTACCCTGGCTCCAGGGGCAGCCCAGCCCAGGACAAGGCTCTCTTCACCGTGC
TGGTGAAGGAAATGGTGGCAGCCTTTGAGCAGGAAGCCAAAGAGGTCAACAAGCCCAGG
CTCATGGTCACAGCTGCCGTTGCTGCAGGGCTCTCCACCATCCAGGCTGGCTACGAGAT
TGCTGAGCTTGGCAAGTACCTGGACTACTTCCACGTCATGACCTACGACTTCCACGGGG
CCTGGGACTCCACCACAGGGGAGAACAGTCCTCTCTACAAGGGCCCAGCCGACACTGGG
GACTTGGTCTACTTCAATGTGGACTATGCCATGAACTACTGGAAGGACAACGGAGCCCC
AGCCGAGAAGCTGCTCGTGGGCTTCCCGACCTATGGCCACAACTTCGTCCTCCAGAACC
CCTCCAACACTGCTGTTGGGGCTCCAGCAAGTGGGCCTGGCCCAGCTGGACCCTACACA
AGGCAGTCTGGATTCCTGGCTTACTATGAGATCTGCACCTTCCTGAGTGAAGGAGCCAC
CCAGGCTTGGGATGCCCCCCAGGATGTGCCCTATGCCTACAAGGGCAACGAGTGGGTTG
GCTATGACAACATCAAGAGCTTCAACATCAAGGTGGACTGGCTGAAGAAGAACAAGTTT
GGAGGTGCCATGGTTTGGGCCCTTGACATGGATGACTTCACTGGCACCTTCTGCAAGGA
GGGCAAATATCCTCTGATCAACACCCTGAAGAAAGGTCTTGGTCTGGACAGTGGTGACT
GTGTGCCTCCAGCTGAGCCCATCCCCCCCATCACCGAGGCTCCTCCTAGCCAAGGAGGT
GGCTCTGGGGGCTCTGGGGGCTCTGGGGGCTCTGGGGGCAGTGGATTCTGTGCTGGCAA
GGCCAATGGCCTCTATGCAGACCCCCAGAACAAGAAGAACTTCTACAACTGTGTGAATG
GTGTCACCTACCTGGAGAGCTGCCAGGCTGGCCTGGTCTTTGATGCCAGCTGCTCCTGC
TGCAACTGGGCC
>Opisthocomus_hoazin_2
ATGGCCAAACTCACTTTGCTTACCGATCTACCCCACCTCCTCCATTTCTCTGCAGGCAC
TGCCTATGTGCTGTCATGTTACTTCACCAACTGGGCCCAATATAGGCCTGACCTGGGAA
AGTTCATGCCTGACAACATCGACCCGTGCCTGTGCGACCACCTGATCTACGCCTTTGCT
GGGATGTCCAACAATGAGATCACAACTTACGAATGGAACGATGAGACCCTTTACAAATC
CTTCAATGACCTGAAGAACCACAGTGTAAAACTGAAGACCCTCCTGGCGATTGGAGGAT
GGAATTTTGGGACAGCCAAGTTCTCCACAATGGTTTCCACTTCCGAGAACCGCCAGACC
TTCATCAAGTCTGTCATCAAATTCCTGCGCCAGCATCAATTTGATGGGCTGGACATTGA
CTGGGAATACCCAGGGTCCAGGGGCAGCCCACCCCAGGACAAGACTCTCTTTACCGTCC
TGGTTAAGGAAATGCTGGCAGCCTTTGAGCAGGAAGCCAAACAGGTCAACAAGTCCCGG
CTCATGATCACCGCTGCTGTTGCTGCAGGACTTCCTAACATTCAGGCTGGCTATGAGAT
TCCTGAGCTTGGAAAGTACCTGGACTACATCCATGTGATGACTTATGACTTCCATGGCT
CCTGGGATGGACAAACTGGCGAGAACAGCCCTCTGTATAAAGGCCCAGCTGACACTGGT
GACCTCGTCTACCTCAATGTTGACTATGCTATGAACTATTGGAAGAACAGTGGTGCCCC
AGCTGAGAAACTCCTTGTTGGATTCCCAACCTACGGACATAACTTCAACCTCCAAAACC
CATCTGACACTGCTGTTGGGGCACCAGCATCAGGACCTGGGCCAGCTGGACCTTACACA
AGGCAGGCTGGGTTTTTGGCTTACTACGAGATCTGCACATTCCTGGACTCTGGAGCCAC
CCAGGCTTGGGATGCCCCCCAGGACGTGCCCTTTGGAGGTGCTATGGTTTGGGCCCTTG
ATATGGATGACTTCACTGGCTCTTTCTGCAAGCAGGGCAAATATCCCTTGATCACCACC
CTGAAGAATGGTCTTGGTCTGCAAAACAGTGACTGCGTGCCTCCGGCTCAGCCCAACCC
TCCAATCACTGAAGCTCCTAGCCAAGGAAGGGGAAGTGGGAGCGGGGCCTCAGGCAGCA
ATACTGGTAGCTCTGGTGGGAGCAGTTTCTGCGCTAGCAAGGCCAACGGCATCTATGCA
GATCCAACCAACAAGAGCAACTTCTACAACTGTGCTAATGGTGTAACCTTCATGCAGAG
CTGCCAGGAAGGCCTCGTTTTTGATACCAGCTGCTCCTGCTGCAACTGGCCA
>Hirundo_rustica2
ATGGCCAAGCTCACTCTGCTCACCGGCCTGGCGCTGCTGCTGAACGCCCACCTCGGCAC
TGCCTATGTGCTGACCTGTTACTTCACCAACTGGGCCCAGTACAGACCTGGTGAGGGCA
AATACACCCCTGAGAACATCGACCCGAACTTGTGCAGCCACCTGATCTATGCCTTCGCC
GGCATGAACAACAACGAGATCACCACCTACGAGTGGAACGACGAGACCCTGTACAAGTC
CTTCAATGGCCTCAAGAACCAGAACAGGAACCTGAAGACCCTGCTGGCCATTGGAGGAT
GGAATTTCGGCACACAGAAGTTCACCACCATGGTCTCCACACCCCAGAACCGCCAGACC
TTCATCAACTCCGTGGTCAGGTTCCTGCGCCAGTACGGATTTGACGGGCTGGACCTGGA
CTGGGAATACCCCGGCTCCAGGGGCAGCCCTGCCCAGGACAAGGCTCTCTTCACCGTCC

TGGTTAAGGAATTGCTGGCAGCCTTTGAGCAGGAAGCCAGGCAGAGCAACCGGCCCCGG
CTCATGGTCACTGCTGCTGTGGCTGGAGGGCTTTCCACCATCCAGGCTGGCTACGAGAT
TGCTGAGCTGGGCAAGTACCTGGATTACATCCACGTGATGACCTACGACTTCCATGGGC
CCTGGGATGGCTCCACGGGCGAGAACAGCCCCCTGTTCAACAGTGGCAGCACCCTCAGT
GTTGAATATGCCATGAACTACTGGAAGAACAACGGCGCTCCAGCTCAGAAGCTCCTGGT
GGGATTCCCAACCTATGGAAAACCTTCACCCTGCAGAACCCATCCAACACCGCCGTGG
GAGCCCCAACCTCCGGGCCTGGCCCTGCAGGACCCTATACCGGGGAGGCCGGGCTCTTG
GCTTACTACGAGATCTGCAGCTTCCTGAACTCTGGAGCCACCCAGGCTTGGGATGCCGC
CGAGGACGTGCCCTACGCCTACAGGGGCAGTGAGTGGATCGGCTATGACAATGTCAGGA
GCTTCGAACTCAAGGTGGATTGGCTGAAGAAGAACAACTTTGGAGGTGCCATGGTCTGG
ACCATCGACCTGGATGACTTCACTGGCACCTTCTGCCACCAGGGCAAATACCCCCTGAT
CTCCACCCTGAAGAAGGGCCTGGGGCTG
>Hirundo_rustica1
ATGGCCAAGCTCACTCTGCTCACCGGCCTGGCGCTGCTGCTGAACGCCCACCTCGGCAC
TGCCTATGTGCTGACCTGTTACTTCACCAACTGGGCCCAGTACAGACCTGGCCTGGGCA
AGTTCACCCCCGAAAATGTCGACCCGTGCCTATGCAACCACCTGATCTATGCCTTCGCC
GGCATGAACAACAACGAGATCACCACCTACGAGTGGAACGACGAGACCCTGTACAAGTC
CTTCAATGGCCTCAAGAACCAGAACAAAGATCTGAAGACCCTGCTGGCCATTGGAGGAT
GGAATTTTGGCACACAGAAGTTCACCACCATGGTCTCATCTCCTGAGAACCGCCAGACC
TTCATCAAGTCTGTCATCAAATTCCTGAGGCAGTATCAGTTTGACGGGCTGGACCTGGA
CTGGGAATACCCCGGCTCCAGGGGCAGCCCTGCCCAGGACAAGGCTCTCTTCACCGTCC
TGGTTAAGGAATTGCTGGCAGCCTTTGAGCAGGAAGCCAAACAGAGCAACCGGCCCCGG
CTCATGGTCACCGCCGCCGTTGCTGCCGGACTTTCCACCATCCAGGCTGGCTACGAGAT
TGCCGAGCTGGGCAAGTACCTGGATTACATCCACGTGATGACCTACGACTTCCATGGAT
CCTGGGAGAGGAACACTGGCGAGAACAGCCCCCTGTACGCTGGCCCTGCTGACAGCGGA
GACTACAAATACTTCAATGTCGAATACGCCATGAATTATTGGAAGAGCAATGGTGCCCC
AGCTGAGAAGCTCCTTGTGGGATTCCCAACCTACGGGAAGAGCTTCACCCTGCAGAACC
CATCTGACACCTCTGTTGGAGCTCCAGCATCTGGGCCTGGCCCCGCTGGGCCCTACACC
AGGGAGGCTGGAACTCTGGCTTACTACGAGATCTGCACTCTTCTGAGTTCTGGAGCCAC
CCAGGCTTGGGATGAACCCCAGGATGTTCCCTACGCCTACCAGGGCAGCGAGTGGGTCG
GCTATGACAACATCAAGAGCTTCGGCCTCAAGGTGGATTGGCTGAAGAAGAACAACTTT
GGAGGTGCCATGGTCTGGGCCCTGGACATGGATGACTTCACTGGGGATTTCTGCAAGGA
AGGCAAATACCCCCTGATCTCCACCCTGAAGAAGGGCCTGGGGCTGCAGAGCGGCGACT
GCGTTCCCCCTGCTGAGCCCCTGCCTCCCATCACCGAGGCTCCCACCACCACCAGCAGC
AGCAGCTCCGGCGGCTCCGGCGGCTCCGGCTTCTGCGCCGGGAAACCCAACGGGATCTA
CGCAGACCCCAACAACAAGAGGAACTTCTACAGCTGCCTGAACGGCCAGACCTTCCTGC
AGAGCTGCGAGCAGGGGCTGGTCTTCGACCCCGTCTGTACCTGCTGCAACTGGCCCCAG
>Sturnus_vulgaris1
ATGAACAACAACGAGCTGACAACGTACGAGTGGAACGACGAGACCCTGTACAAGTCCTT
CAATGGCCTCAAGAACCAGAACAAAGATCTGAAGACCCTGCTGGCCATTGGGGGATGGA
ATTTCGGCACAGCCAAGTTCACCACCATGGTTTCCTCTCCTGAGAACCGCCAGACCTTC
ATCAAGTCTGCCATCAAATTCCTGCGCCAGTACCAGTTTGATGGGCTGGACCTGGACTG
GGAATACCCCGGCTCCAGGGGCAGCCCCGCCCAGGACAAGGCTCTCTTCACCGTCCTGG
TTAAGGAATTGCTGGAAGCTTTTGAGCAGGAAGCCAAGCAGACCAACCAGCCCCGGCTC
CTGGTCACTGCTGCTGTGGCTGCTGGACTTTCCACCATCCAGGCTGGCTACGAGATCGC
TGAGATTGGCAAGTACCTGGATTACATCCACGTCATGACCTACGACTTCCACGGATCCT
GGGAGAGGAACACTGGCGAGAACAGCCCCCTGTACGCCGGCCCTGCTGACTCTGGGGAC
AACAAATACTTCAACGTTGAATACGCCATGAATTATTGGAAGAGCAACGGTGCCCCAGC
TGAGAAGCTGCTGGTGGGATTCCCAACCTATGGAAAGAGCTTCACCCTGCAGAACCCAT
CCAACACTGCCGTGGGAGCTCCAGCATCCGGGCCCGGCCCTGCTGGACCCTACACCAGG
GAGGCTGGAACTCTGGCTTACTATGAGATCTGCACTCTGCTGAGCTCTGGAGCCACCCA
GGCTTGGGATGAACCCCAGGATGTTCCCTACACCTACAAGGGCAGCGAATGGGTCGGCT
ACGACAACATCAAGAGCTTTGGCCTCAAGGTGGATTGGCTGAAGAAGAACAACTTTGGA
GGTGCCATGGTCTGGGCCCTGGACATGGATGACTTCACTGGGGAGTTCTGCAAGGAAGG

CAAATACCCCCTGATCTCCACGCTGAAGAAGGGCCTGGGGCTGCAGAGCGGGGGCTGCG
TTCCCCCCAGTGAGCCCCTGCCCCCCATCACTGCGGCTCCCACCACCACCAGCTCCAGC
GGCGGCTCCGGGGGCTCCGGGGGCTCCGGGGGCTCCGGGGGCTCCGGGTTCTGTGCTGG
GAAACCCAACGGGATCTACGCGGACCCCAACAACAGCAGGAACTTCTACAGCTGCCTGA
ACGGCCAGACCTTCGTGCAGAGCTGCGAGCAGGGGCTGGTTTTTGACCCTGCCTGTACC
TGCTGTAACTGGCCCCAG
>Sturnus_vulgaris2
ATGGCCAAGCTCACTCTGCTCACCGGTCTGGCCCTGCTGCTGAACGCCCAGCTCGGCAC
TGCCTATGTGCTGACCTGCTACTTCACCAACTGGGCCCAGTACAGACCTGGAGAGGGCA
AATACACCCCTGAGAACATCGACCCCAACCTGTGCAACCACCTGATCTATGCCTTCGCT
GGCATGAACAACAATGAGATCACCACCTACGAGTGGAACGACGAGACCCTGTACAAGTC
CTTCAATGGCCTCAAGAACCAGAACAGGAACCTGAAGACCCTGCTGGCTATTGGAGGAT
GGAATTTCGGCACACAGAAGTTCACCACCATGGTCTCCACACCCCAGAACCGCCAGACC
TTCATCAACTCCGTGGTCAGGTTCCTGCGCCAGTACGGATTCGATGGGCTGGACCTGGA
CTGGGAATACCCCGGCTCCAGGGGCAGCCCCGCCCAGGACAAGGCTCTCTTCACCGTCC
TGGTTAAGGAACTGCTGGCAGCCTTCGAGCAGGAAGCCAAGCAGACCAACCAGCCCCGG
CTCCTGGTCACTGCTGCTGTGGCCGGAGGACTTTCCACCATCCAGGCTGGCTACGAGAT
CGCTGAGCTGGGCAAGTACCTGGATTACATCCACGTCATGACCTACGACTTCCACGGGC
CCTGGGACGGCTCCACGGGCGAGAACAGCCCCCTGTTCAGCAGTGGCAGCACCCTCAGT
GTTGAATACGCCATGAATTATTGGAAGAACAACGGTGCTCCAGCTCAGAAGCTCCTGGT
GGGATTCCCAACCTATGGAAAAACCTTCACCCTGCAGTACCCATCCAACACTGCCGTGG
GAGCCCCAAGCTCTGGGCCTGGCCCTGCTGGACCCTACACCAGGGAGGCTGGGCTCCTG
GCTTACTACGAGATCTGCACCTTCCTGAACTCTGGAGCCACCCAGGCTTGGGATGCCCC
CGAGGATGTTCCCTACGCCTACAAGGGCAACGAATGGATTGGATATGATAACGTCAAGA
GCTTTGGCCTCAAGGTGGATTGGCTGAAGAAGAACAACTTTGGAGGTGCCATGGTCTGG
ACCATCGACCTGGATGACTTCACTGGCACCTTCTGCCACGAGGGCAAATACCCCCTGAT
CTCCACGCTGAAGAAGGGCCTGGGGCTC
>Parus_atricapillus2
ATGGCCAAGCTCACTCTGCTCACCGGCCTGGCGCTGCTGCTGAACGCCCATCTCGGCAC
TGCCTATGTGCTGACCTGTTACTTCACCAACTGGGCCCAGTACCGGCCTGGTGAGGGCA
GATACACCCCTGAGAACATCGACCCCAACCTGTGCAGCCACCTGATCTATGCCTTTGCT
GGCATGAACAACAACGAGATCACCACCTATGAGTGGAACGATGAGACCCTGTACAGGTC
CTTCAATGGCCTCAAGAACCAGAACAGCAACCTGAAGACTCTGCTGGCCATTGGAGGAT
GGAATTTCGGCACCGAGAAGTTCACCACCATGGTCTCCACACCCCAGAACCGCCAGACC
TTCATCAACTCCGTGGTCAGATTCCTGCGCCAGTACGGATTTGATGGGCTGGACCTGGA
CTGGGAATACCCCGGCTCCAGGGGCAGCCCTGCCCAGGACAAGGCTCTCTTCACCGTCC
TGGTTAAGGAACTGCTGGCAGCCTTCGAGCAGGAGGCCAGGCAGACCAACCGGCCCCGG
CTCATGGTCACCGCCGCTGTGGCCGGAGGACTTTCCACCATCCAGGCTGGCTACGAGAT
TGCTGAGCTGGGCAAGTACCTGGATTACATCCACGTGATGACCTACGACTTCCACGGGC
CCTGGGATGGCTCTGCAGGCGAGAACAGCCCCCTGTTCAGCAGCGGCAGCACCCTCAGT
GTTGAATATGCCATGAACTACTGGAAGAACAACGGTGCCCCAGCTCAGAAGCTCCTGGT
GGGATTCCCAACCTATGGAAAACCTTCACCCTGCAGAACCCATCCAACACCGCCATCG
GAGCCCCAACCTCCGGGCCTGGCCCCGCGGACCCTACACCAGGGAGGCCGGGCTCTTG
GCTTACTACGAGATCTGCACCTTCCTGAGCTCTGGAGCCACCCAGGCTTGGGATGCCCC
TGAGGATGTTCCCTACGCCTACAAGGGCAGCGAATGGGTCGGCTACGACAACGTCAAGA
GCTTCAGCCTCAAGGTGGACTGGCTGAAGCAGAACAACTTTGGAGGTGCCATGGTGTGG
ACCATCGACCTGGATGACTTCACTGGCACCTTCTGCCACGAGGGCAAATACCCCCTGAT
CTCCACCCTGAAGAAGGGCCTGGGGCTG
>Parus_atricapillus1
ATGGCCAAGCTCACTCTGCTCACCGGCCTGGCGCTGCTGCTGAACGCCCATCTCGGCAC
TGCCTATGTGCTGACCTGTTACTTCACCAACTGGGCCCAGTACCGGCCTGGCCTGGGTA
AGTACACCCCCGAAAATGTCGACCCCTGCTTGTGCAACCACCTGATCTACGCCTTCGCC
GGCATGAACAACAACGAGATCACCACCTACGAGTGGAACGATGAGACCTTGTACAGGTC
CTTCAATGGCCTCAAGAACCAGAACAAAGATCTGAAGACCCTGCTGGCCATTGGAGGAT

GGAATTTCGGCACACAGAAGTTCACCACCATGGTCTCCACACCCCAGAACCGCCAGACC
TTCATCAACTCCGTCATCAAATTCCTGCGCCAGTATCAGTTTGATGGGCTGGACCTGGA
CTGGGAATACCCCGGCTCCAGGGGCAGCCCCGCCCAGGACAAGGCTCTCTTCACCGTCC
TGGTTAAGGAACTGCTGGAAGCTTTTGAGAAGGAAGCCAAACAGACCAACCAGCCCCGC
CTCATGGTCACCGCCGCTGTTGCTGCTGGACTTTCCACCATCCAGGCTGGCTATGAGAT
TGCTGAGATTGGCAAGTACCTGGATTACATCCACGTGATGACCTACGACTTCCACGGAT
CCTGGGAGAGGAACACTGGCGAGAACAGCCCCCTGTTCGCCGGCCCTGCTGACACTGGC
GACTACAAATACTTCAACGTTGAATACGCCATGAATTATTGGAAGAGCAATGGTGCCCC
AGCTGAGAAGCTCCTGGTGGGATTCCCAACCTACGGAAAGAGCTTCACCCTGCAGAACC
CATCTGACACCTCCGTTGGAGCTCCAGCATCCGGCCCTGGCCCCGCTGGACCCTACACC
AGGGAGGCTGGAACTCTGGCTTACTACGAGATCTGCAGTCTCCTGAGCTCTGGAGCCAC
CCAGGCTTGGGATGAACCCCAGGATGTTCCCTACGCCTACAAGGGCAGCGAATGGGTCG
GCTACGACAACGTCAAGAGCTTCGGCCTCAAGGTGGACTGGCTGAAGAAGAACAACTTT
GGAGGTGCCATGGTGTGGGCCCTGGACATGGATGACTTCACTGGCACCTTCTGCCACGA
GGGCAAATACCCCCTGATCTCCACCCTGAAGAAGGGCCTGGGGCTGCAGAACGGCGACT
GCGTTCCCCCTGCTGAGCCCCTGCCTCCCATCACCGAGGCTCCCACCACCACCACCACC
AGCGGCGGCAGCGGCGGCTCCGGCTTCTGCGCCGGGAAACCCAACGGGATCTACGCGGA
CCCCGAGAACAACAGGAACTTCTACAACTGCGTGAACGGCCAGGGCATCGCGCAGAGCT
GCGAGCCAGGGCTGGTCTTCGACCCCCTCTGCAGCTGCTGCAACTGGCCCCAG
>Empidonax_traillii_2
ATGGTTTCCTCTGCTGAGAACCGCCAGACCTTCATCAAGTCTGTCATCAAATTCCTGCG
CCAGTACCAGTTTGATGGTCTGGACCTGGACTGGGAATACCCCGGCTCCAGGGGCAGCC
CAGCCCAGGACAAGGGGCTCTTCACCGTCCTGGTTAAGGAACTGCTGGAAGCCTTCGAG
CAGGAAGCCAAACAGACCAACCAGCCCCGTCTCATGGTCACTGCAGCTGTGGCTGCAGG
ACTTTCCACCATCCAGGCTGGCTACGAGATTGCTGAGATTGGGAAGTACCTGGACTACA
TCCACGTGATGACCTACGACTTCCACGGCTCCTGGGAGAGAAACACTGGCGAGAACAGC
CCCCTGTACGCAGGCCCAGCTGACACCGGCGACTACAAGTACTTCAATGTCGAATATGC
CATGAATTACTGGAAGGACAATGGTGCCCCAGCCGAAAAGCTCCTTGTTGGCTTCCCAA
CCTACGGAAAAAGCTTCACCCTGCAAAACCCATCTGACACCTCTGTCGGGGCTCCAGCA
TCCGGCCCTGGACCAGCTGGACCTTACACCAGGGAGGCTGGAACACTGGCTTACTATGA
GATCTGCTCTCTCTTGAGCTCTGGAGCCACCCAGGCTTGGGATGAACCCCAGGACGTGC
CCTATGCCTACAAGGGGAGCGAGTGGGTTGGCTACGACAACATGAAGAGCTTCAGCATC
AAGGTTGACTGGCTGAAGAAGAACAACTTTGGAGGCGCCATGGTTTGGGCCCTTGACAT
GGATGACTTCACTGGCACTTTCTGCAATGAAGGCAAATACCCCCTGATCTCCACCCTGA
AGAAGGGCCTTGGTCTGCAGAATGGCGACTGTGTGCCTCCCTCTGAGCCCCTGCCTCCA
ATCACTGAGGCTCCCACTACTACAAGTGGCAGTGGGAGCGGCGGCTCTGGCGGGAGCGG
CGGCTCTGGCGGGAGCGGCTTCTGCGCCGGCAAACCCAACGGCATCTACGCGGACCCAG
AGAACAACAGGAACTTCTACAACTGCTTGAACGGCCAGACCTTCGTGCAGAGCTGCGAA
GAGGGGCTCGTCTTCGACCCCCTCTGCACCTGCTGCAACTGGCCACGAAGCTCC
>Ammospiza_nelsoni2
ATGGCCAAGCTCACTCTGCTCACCGGTCTGGCCCTGCTGCTGAACGCCCAGCTCGGCAC
TGCCTATGTGCTGACCTGTTACTTCACCAACTGGGCCCAGTACAGGCCTGGGGAGGGGA
GGTACACCCCCGAGAACATCGACCCCAACCTGTGCAACCACCTGATCTACGCCTTCGCC
GGCATGAACAACAACGAGATCACCACCTACGAGTGGAACGACGAGACCCTCTACAAGTC
CTTCAATGGCCTCAAGAACCAGAACCGCAACCTGAAGACCCTGCTGGCCATTGGAGGAT
GGAATTTCGGCACACAGAAGTTCTCCACCATGGTCTCCACGCCCCAGAACCGCCAGACC
TTCATCAACTCCGTGGTCAGGTTCCTGCGCCAGTATCAGTTCGACGGGCTGGACCTGGA
CTGGGAATACCCCGGCTCCAGGGGCAGCCCTGCCCAGGACAAGTCCCTCTTCACCGTCC
TGGTTAAGGAAATGGTGGCAGCCTTCGAGCAGGAAGCCAAGCAGAGCAACAGGCCCCGG
CTCATGGTCACTGCTGCTGTGGCTGGAGGACTTTCCACCATCCAGGCTGGCTACGAGAT
TGCTGAGCTGGGCAAGTACCTGGATTACATCCACGTCATGACCTACGACTTCCACGGGC
CCTGGGACGGCTCCACGGGCGAGAACAGCCCCCTGTTCAGCAGCGGCAGCACCCTCAGT
GTTGAATACGCCATGAACTACTGGAAGAACAACGGCGCCCCAGCCCAGAAGCTGCTGGT
GGGATTCCCAACATATGGAAAGAGCTTCACCCTGCAGAACCCATCCAACACGGCCATCG

GTGCCCCCAGCTCCGGGCCTGGCCCTGCAGGGCCCTACACCGGGGAGGCCGGGCTCCTG
GCTTACTACGAGATCTGCACCTTCCTGAGCTCTGGAGCCACCCAGGCTTGGGATGCGCC
TCAGGATGTCCCCTACACCTACAAGGGCAGCGAATGGGTCGGCTACGACAACGAGAGGA
GCTTTGGCCTCAAGGTGGACTGGCTGAAGAAGAACAACTTTGGAGGGGCCATGGTGTGG
ACCATCGACCTGGATGACTTCACTGGCAACTTCTGCCACCAGGGCAAATACCCCCTGAT
CTCCACGCTGAAGAGGGGCCTGGGGCTG
>Ammospiza_nelsoni1
ATGGCCAAGCTCACTCTGCTCACCGGTCTGGCCCTGCTGCTGAACGCCCAGCTCGGCAC
TGCCTATGTGCTGACCTGTTACTTCACCAACTGGGCCCAGTACAGGCCTGGCCTGGGCA
AGTTCACCCCTGAAAATGTCGACCCTTGCCTGTGCAACCACCTGATCTACGCCTTCGCC
GGCATGAACAACAACGAGATCACCACCTACGAGTGGAACGACGAGACCCTCTACAAGTC
CTTCAATGGCCTCAAGAACCAGAACAAAGACCTGAAGACCCTGCTGGCCATTGGAGGAT
GGAATTTCGGCACAGCCAAGTTCTCCACCATGGTCTCCACGCCCCAGAACCGCCAGACC
TTCATCAACTCCGTCATCAAATTCCTGCGCCAGTATCAGTTCGACGGGCTGGACCTGGA
CTGGGAATACCCCGGCTCCAGGGGCAGCCCTGCCCAGGACAAGTCCCTCTTCACCGTCC
TGGTTAAGGAAATGGTGGCAGCCTTCGAGCAGGAAGCCAAGCAGAGCAACAGGCCCCGG
CTCATGGTCACTGCTGCTGTGGCTGCTGGACTCTCCACCATCCAGGCCGGCTACGAGAT
TGCTGAGATTGGCAAGTACCTGGATTACATCCATGTCATGACCTACGACTTCCACGGCT
CCTGGGAGAGGAACACTGGAGAGAACAGCCCCCTGTTCGCTGGCCCTGCCGACAGCGGC
GACTACAAATACTTCAACGTCGAATACGCCATGAATTATTGGAAGAGCAATGGTGCCCC
AGCTGAGAAGCTCCTGGTGGGATTCCCAACCTATGGAAAGAGCTTCACCCTGCAGAACC
CATCTGACACCTCCGTTGGAGCTCCAGCATCCGGCCCTGGCCCCGCTGGACCCTACACC
AGGGAGGCTGGAACTCTGGCTTACTACGAGATCTGCTCTCTCCTGAGCTCTGGAGCCAC
CCAGGCTTGGGATGAACCCCAGGATGTCCCCTACGCCTACAAGGGCAGCGAATGGGTCG
GCTACGACAACGTCAAGAGCTTTGGCCTCAAGGTGGACTGGCTGAAGAAGAACAACTTT
GGAGGAGCCATGGTGTGGGCCCTGGACATGGATGACTTCACTGGGGATTTCTGCAAGGA
AGGCAAATACCCCCTGATCTCCAGCCTGAAGAAGGGCCTGGGGCTGCAGAGCAGCGACT
GCGTTCCCCCCTCTAAGCCCCTTCCTCCCGTCACTGAGGCTCCCACCACCACCTCCGGC
GGCTCTGGCGGCTCTGGCGGCTCCGGCGGCTCTGGATTCTGTGCCGGGAAACCCAACGG
GATCTACGCAGACCCCAACAACAAGAGGAACTTCTACAGCTGCCTGAACGGCCAGACCT
TCGTGCAGAGCTGCGAGCAGGGGCTGGTCTTCGACCCCGCCTGCTCCTGCTGCAACTGG
CCCCAG
>Corapipo_altera2
ATGGCCAAGCTCACTCTGCTCACCGGTCTGGCCCTGCTGCTCAACGCCCACCTCGGCAC
TGCCTACGTGCTGACATGTTACTTCACCAACTGGGCCCAGTACAGGCCTGGCCTGGGCA
AATACACACCCGAAAATGTCGACCCCTGCCTGTGCAACCACCTGATCTACGCCTTCGCC
GGGATGAACAACAACGAGATCACCACTTATGAATGGAACGATGAGACCCTCTACAAATC
CTTCAATGGCCTGAAGAACCAGAACAAAGATCTGAAGACACTCCTGGCCATTGGAGGAT
GGAATTTTGGCACAGCCAAGTTCACTACAATGGTTTCCTCTGCTGAGAACCGCCAGACC
TTCATCAAGTCTGTCATCAAATTCCTGCGCCAGTACCAGTTTGATGGGCTGGACCTGGA
CTGGGAATACCCCGGCTCCAGGGGCAGCCCAGCCCAGGACAAGGGGCTCTTCACCGTCC
TGGTTAAGGAATTGCTGGCAGCCTTTGAGCAGGAAGCCAAACAGACCAACCAGCCCCGT
CTCCTGGTCACCGCGGCTGTGGCTGGAGGACTTTCCACCATCCAGGCCGGCTACGAGAT
TGCTGAGCTGGGCAAGTACCTGGACTACATCCACGTGATGACCTATGACTTCCATGGCT
CCTGGGAGAGAAACACTGGCGAGAACAGCCCCCTGTTCACAGGCCCAGCTGACACTGGG
GACTACAAGTACTTCAACATCGAATATGCTATGAATTACTGGAAGGACAATGGTGCCCC
AGCTGAGAAGCTCCTTGTTGGCTTCCCAACCTACGGAAAAAGCTTCACCCTGCAAAACC
CATCTGACACCTCTGTTGGGGCTCCAGCATCAGGCCCTGGGCCAGCTGGACCTTACACC
AGGGAGGCTGGGACACTGGCTTACTATGAGATCTGCTCCCTCCTGAGCTCTGGAGCCAC
CCAGGCTTGGGATGAACCCCAGGACGTGCCCTATGCCTACAAGGAGAGCGAATGGGTTG
GCTACGACAACACGAAGAGCTTCAGCATCAAGGTTGACTGGCTGAAGAAGAACAACTTT
GGAGGGGCCATGGTTTGGGCCCTCGACATGGATGATTTCACTGGCACTTTCTGCAATGA
GGGCAAATACCCCCTGATCTCCACCCTGAAGAAGGGCCTCGGTCTGCAGAACGGCGACT
GTGTGCCTCCTGCTGAGCCCCTGCCTCCAGTCACTGAGGCTCCCACTACCACCAGTGGA

AGCGGGAGCGGCGGCTCTGGTGGGAGCGGCTTCTGCGCCGGCAAAGCCAACGGCATCTA
CGCAGACCCAGAGAACAACAGGAACTTCTACAACTGCTTGAACGGCCAAACCTTCGTCC
AGAGCTGCCAACAGGGTCTCGTCTTCGACCCCGTCTGCTCCTGCTGCAACTGGCCA
>Haemorhous_mexicanus
ATGGCCAAGCTCCCTCTGCTCGCCGGTGTGGCCCTGCTGCTGAACGCCCAGCTCGGCTC
TGCCTATGTGCTGACCTGCTACTTCACCAACTGGGCCCAGTACAGGCCAGGTGAGGGCA
GGTACACCCCCGAGAACATCGACCCTTGTCTGTGCAACCACCTGATCTACGCCTTCGCC
GGCATGAGCAACAACGAGATCACCACCTACGAGTGGAACGACGAGACCCTCTACCAGTC
CTTCAACGGCCTCAAGAACCAGAATAAAGATCTGAAGACCCTGCTGGCCATTGGAGGAT
GGAATTTCGGCACAGCCAAGTTCTCCACCATGGTCTCCACGCCCCAGAACCGCCAGACC
TTCATCAACTCCGTCATCAAATTCCTGCGCCAGTACGGATTCGACGGGCTGGACCTGGA
CTGGGAATACCCCGGCTCCAGGGGCAGCCCTGCCCAGGACAAGGCTCTCTTCACCCTCC
TGGTTAAGGAACTGGTGGCAGCCTTCGAGCAGGAGGCCGAGCAGACCAACAGGCCCCGG
CTCATGGTCACTGCTGCTGTGGCCGGAGGGCTCTCCACCATCCAGGCTGGCTACGAGAT
CGCTGAGCTGGGCAAGTACCTGGATTACATCCACGTCATGACCTACGACTTCCACGGGC
CCTGGGACGGCTCCACGGGCGAGAACAGCCCCCTGTTCAGCAGTGGCAGCCCCCTCAGT
GTTGAATACGCCATGAACTACTGGAAGAACAGCGGCGCCCCAGCCCAGAAGCTGCTGGT
GGGATTCCCGACCTATGGAAAGAGCTTCACCCTGCAGAGCCCGTCCGACAGCGCCGTCG
GAGCCCCCAGCTCCGGGCCTGGCCCTGCAGGACCCTACACCAGGGAGGCCGGGCTCCTG
GCTTACTACGAGATCTGCTCCCTCCTGAGCTCCGGAGCCACCCAAGCCTGGGATGCCCC
CCAGGATGTCCCCTACACCTACAAGGGCAGCGAGTGGGTCGGCTACGACAACGAGAAGA
GCTTCGGCCTCAAGGTGGACTGGCTGAAGAAGAACAACTTTGGAGGGGCCATGGTGTGG
GCCCTGGACATGGATGACTTCACTGGCACCTTCTGCCACCAGGGCAAATACCCCCTGAT
CTCCAGCCTGAAGAGGGGCCTGGGGCTGCAGAGCGGCGGCTGCACTCCCCCCGCTGAGC
CCCTGCCTCCCATCGCTGAGGCTCCCACCACCCCCAACAGTGGCTCTGGTGACTCCGGT
GGCTCCGGTGGCTCCGGTGGCTCTGGTGGCTCCGGTGGCTCTGGTTTCTGTGCCGGGAA
ACCCAACGGGATCTACGCCGACCCCAGCAACAAGGGGAACTTCTACAGCTGCCTGAACG
GGCGGACCTTCGTGCAGAGCTGCCAGCAGGGGCTGGTCTTCGACGCCCTCTGCTCCTGC
TGCAACTGGCCCCAG
>Ammospiza_caudacuta2
ATGGCCAAGCTCACTCTGCTCACCGGTCTGGCCCTGCTGCTGAACGCCCAGCTCGGCAC
TGCCTATGTGCTGACCTGTTACTTCACCAACTGGGCCCAGTACAGGCCTGGGGAGGGGA
GGTACACCCCCGAGAACATCGACCCCAACCTGTGCAACCACCTGATCTACGCCTTCGCC
GGCATGAACAACAACGAGATCACCACCTACGAGTGGAACGACGAGACCCTCTACAAGTC
CTTCAATGGCCTCAAGAACCAGAACCGCAACCTGAAGACCCTGCTGGCCATTGGAGGAT
GGAATTTCGGCACACAGAAGTTCTCCACCATGGTCTCCACGCCCCAGAACCGCCAGACC
TTCATCAACTCCGTGGTCAGGTTCCTGCGCCAGTATCAGTTCGACGGGCTGGACCTGGA
CTGGGAATACCCCGGCTCCAGGGGCAGCCCTGCCCAGGACAAGTCCCTCTTCACCGTCC
TGGTTAAGGAAATGGTGGCAGCCTTCGAGCAGGAAGCCAAGCAGAGCAACAGGCCCCGG
CTCATGGTCACTGCTGCTGTGGCTGGAGGACTTTCCACCATCCAGGCTGGCTACGAGAT
TGCTGAGCTGGGCAAGTACCTGGATTACATCCACGTCATGACCTACGACTTCCACGGGC
CCTGGGACGGCTCCACGGGCGAGAACAGCCCCCTGTTCAGCAGCGGCAGCACCCTCAGT
GTTGAATACGCCATGAACTACTGGAAGAACAACGGCGCCCCAGCCCAGAAGCTGCTGGT
GGGATTCCCAACATATGGAAAGAGCTTCACCCTGCAGAACCCATCCAACACGGCCATCG
GTGCCCCCAGCTCCGGGCCTGGCCCTGCAGGGCCCTACACCGGGGAGGCCGGGCTCCTG
GCTTACTACGAGATCTGCACCTTCCTGAGCTCTGGAGCCACCCAGGCTTGGGATGCGCC
TCAGGATGTCCCCTACACCTACAAGGGCAGCGAATGGGTCGGCTACGACAACGAGAGGA
GCTTTGGCCTCAAGGTGGACTGGCTGAAGAAGAACAACTTTGGAGGGGCCATGGTGTGG
ACCATCGACCTGGATGACTTCACTGGCAACTTCTGCCACCAGGGCAAATACCCCCTGAT
CTCCACGCTGAAGAGGGGCCTGGGGCTG
>Ammospiza_caudacuta1
ATGGCCAAGCTCACTCTGCTCACCGGTCTGGCCCTGCTGCTGAACGCCCAGCTCGGCAC
TGCCTATGTGCTGACCTGTTACTTCACCAACTGGGCCCAGTACAGGCCTGGCCTGGGCA
AGTTCACCCCTGAAAATGTCGACCCTTGCCTGTGCAACCACCTGATCTACGCCTTCGCC

GGCATGAACAACAACGAGATCACCACCTACGAGTGGAACGACGAGACCCTCTACAAGTC
CTTCAATGGCCTCAAGAACCAGAACAAAGACCTGAAGACCCTGCTGGCCATTGGAGGAT
GGAATTTCGGCACAGCCAAGTTCTCCACCATGGTCTCCACGCCCCAGAACCGCCAGACC
TTCATCAACTCCGTCATCAAATTCCTGCGCCAGTATCAGTTCGACGGGCTGGACCTGGA
CTGGGAATACCCCGGCTCCAGGGGCAGCCCTGCCCAGGACAAGTCCCTCTTCACCGTCC
TGGTTAAGGAAATGGTGGCAGCCTTCGAGCAGGAAGCCAAGCAGAGCAACAGGCCCCGG
CTCATGGTCACTGCTGCTGTGGCTGCTGGACTCTCCACCATCCAGGCCGGCTACGAGAT
TGCTGAGATTGGCAAGTACCTGGATTACATCCATGTCATGACCTACGACTTCCACGGCT
CCTGGGAGAGGAACACTGGAGAGAACAGCCCCCTGTTCGCTGGCCCTGCCGACAGCGGC
GACTACAAATACTTCAACGTCGAATACGCCATGAATTATTGGAAGAGCAATGGTGCCCC
AGCTGAGAAGCTCCTGGTGGGATTCCCAACCTATGGAAAGAGCTTCACCCTGCAGAACC
CATCTGACACCTCCGTTGGAGCTCCAGCATCCGGCCCTGGCCCCGCTGGACCCTACACC
AGGGAGGCTGGAACTCTGGCTTACTACGAGATCTGCTCTCTCCTGAGCTCTGGAGCCAC
CCAGGCTTGGGATGAACCCCAGGATGTCCCCTACGCCTACAAGGGCAGCGAATGGGTCG
GCTACGACAACGTCAAGAGCTTTGGCCTCAAGGTGGACTGGCTGAAGAAGAACAACTTT
GGAGGAGCCATGGTGTGGGCCCTGGACATGGATGACTTCACTGGGGATTTCTGCAAGGA
AGGCAAATACCCCCTGATCTCCAGCCTGAAGAAGGGCCTGGGGCTGCAGAGCAGCGACT
GCGTTCCCCCCTCTAAGCCCCTTCCTCCCGTCACTGAGGCTCCCACCACCACCTCCGGC
GGCTCTGGCGGCTCTGGCGGCTCCGGCGGCTCTGGATTCTGTGCCGGGAAACCCAACGG
GATCTACGCAGACCCCAACAACAAGAGGAACTTCTACAGCTGCCTGAACGGCCAGACCT
TCGTGCAGAGCTGCGAGCAGGGGCTGGTCTTCGACCCCGCCTGCTCCTGCTGCAACTGG
CCCCAG
>Oenanthe_melanoleuca2
ATGGCCAAGCTGACTCTGCTCGCCGGCCTGGCCGTGCTGCTGAACGCCCACCTCGGCTC
TGCCTATGTGCTGACCTGCTACTTCACCAACTGGGCCCAGTACCGGCCTGGGGAGGGCA
GGTACACCCCTGAGAACATCGACCCCAACCTGTGCAGCCACCTGATCTACGCCTTCGCT
GGCATGAGCAACAACCAGATCACCACCTACGAGTGGAACGATGAGACCCTCTACAAGTC
CTTCAACGGCCTCAAGAACCAGAACAGGAACCTGAAGACCCTGCTGGCCATTGGAGGAT
GGAATTTCGGCACAGAGAAGTTCACCACCATGGTCTCCACTCCTCAGAACCGCCAGACC
TTCATCAGCTCCGTGGTCAGGTTCCTGCGGCAGCACGGCTTCGACGGGCTGGACCTGGA
CTGGGAGTACCCCGGCTCCAGGGGCAGCCCCGCCCAGGACAAGGCTCTGTTCACTGTCC
TGGTTAAGGAGCTGCTGGCAGCCTTCGAGCAGGAAGCCAAACAGACCAACCAGCCCCGG
CTCATGGTCACGGCTGCTGTGGCCGGAGGACTGTCCACCATCCAGGCTGGCTACGAGAT
CGCTGAGCTGGGCAAGTACCTGGATTACATCCACGTCATGACCTACGACTTCCACGGGC
CCTGGGACGGCTCCACGGGCGAGAACAGCCCCCTGTTCAGCAGCGGCACCCTCAGCGTG
GAATACGCCATGAACTACTGGAAGAACAACGGTGCCCCAGCTCAGAAGCTGCTGGTGGG
ATTCCCAACCTACGGAAAACCTTCACCCTGCAGAACCCATCCAACACGGCCGTGGGAG
CCCCCAGCTCCGGCCCTGGCCCTGCTGGGCCCTACACCCAGGAGGCTGGACTCCTGGCT
TACTATGAGATCTGCAGCTTCCTGAGCTCTGGAGCCACCCAGGCCTGGGATGCCCCTGA
GGATGTTCCTTATGCCTACAAGGGCAGTGAATGGATTGGATACGACAACGTCAAGAGCT
TCAGCCTCAAGGTGGATTGGCTGAAGAAGAACAACTTTGGAGGTGCCATGGTCTGGACC
ATCGACCTGGATGACTTCACTGGCAACTTCTGCCACGAGGGCAGATACCCCCTGATCTC
CACCCTGAAGAAGGGGCTGGGGCTG
>Oenanthe_melanoleuca1
ATGGCCAAGCTGACTCTGCTCGCCGGCCTGGCCCTGCTGCTGAACGCCCACCTCGGCTC
TGCCTATGTGCTGACCTGCTACTTCACCAACTGGGCCCAGTACCGGCCTGGCCTGGGTA
AGTTCACCCCCGAAAATGTCGACCCCTGCCTGTGCAACCACCTGATCTACGCCTTCGCT
GGCATGAACAACAACGAGCTCACCACCTACGAGTGGAACGATGAGACCCTCTACAAGTC
CTTCAACGGCCTCAAGAACCAGAACAAAGACCTGAAGACCCTGCTGGCCATTGGGGGAT
GGAATTTCGGCACAGCCAAGTTCACCACCATGGTCTCCACTCCTGAGAACCGCCAGACC
TTCATCAAGTCTGCCATCAAATTCCTGCGGCAGTACCAGTTCGACGGGCTGGACCTGGA
CTGGGAGTACCCCGGCTCCAGGGGCAGCCCCGCCCAGGACAAGGCTCTGTTCACTGTCC
TGGTTAAGGAACTGCTGGCAGCCTTCGAGCAGGAAGCCAAACAGACCAACCAGCCCCGG
CTCATGGTCACGGCTGCTGTGGCTGCCGGGCTCTCCACCATCCAGGCTGGCTACGAGAT

CGCTGAGATTGGCAAGTACCTGGATTACATCCACGTCATGACCTACGACTTCCACGGCT
CCTGGGAGAGGAACACGGGTGAGAACAGCCCCCTGTACGCCGGCCCTGCTGACACTGGC
GACTACAAATACTTCAACGTTGAATACGCCATGAATTATTGGAAGAGCAACGGTGCCCC
AGCTGAGAAACTCCTGGTGGGATTCCCAACCTATGGGAAGAGCTTCACCCTGCAGGACC
CATCCAACACGGCCGTGGGAGCCCCAGCGTCCGGCCCCGGCCCCGCCGGGCCCTACACC
AGGGAGGCTGGAACTCTGGCTTACTACGAGATCTGCACTCTGCTGAGCTCTGGAGCCAC
CCAGGCTTGGGATGAGCCCCAGGATGTCCCTTATGCCTACAAGGGCAGCGAGTGGGTCG
GCTACGACAACGTCAAGAGCTTTGGCATCAAGGTGGATTGGCTGAAGAAGAACAACTTT
GGAGGAGCCATGGTCTGGGCCCTGGACATGGATGACTTCACTGGGGATTTCTGCAAGGA
GGGCAAATACCCGCTGATCTCCACGCTGAAGAAGGGGCTGGGGCTGCAGAGCGGCGAGT
GCGTTCCCCCCAGCGAGCCGCTGCCCCCCATCACCGAGGCTCCCCCCACCCCCGGCGGC
TCGGGCGGCTCGGGCGGCTCCGGGTTCTGCGCCGGGAAACCCAACGGGCTGTACGCCGA
CCCCACGGACAGCAGGAACTTCTACAGCTGCGTGAACGGCCAGGGCTTCCTGCAGAGCT
GCGAGCAGGGGCTGGTCTTCGACCCCGCCTGCAGCTGCTGCAACTGGCCCCAG
>Agelaius_phoeniceus2
ATGGCCAAGCTCACTCTGCTCACCGGTCTGGCCCTGCTGCTGAACGCCCAGCTCGGCAC
TGCCTATGTGCTGACCTGTTACTTCACCAACTGGGCCCAGTACAGGCCTGGCGAGGGGA
GGTACACCCCCGAGAACATCGACCCCAACCTTTGCAACCACCTGATCTACGCCTTCGCC
GGCATGAACAACAACGAGATCACCACCTACGAGTGGAACGACGAGACCCTCTACAAGTC
CTTCAATGGCCTCAAGAACCAGAACAGGAACCTGAAGACCCTTCTGGCCATTGGAGGAT
GGAATTTCGGCACACAGAAGTTCTCCACCATGGTCTCCACGCCCCAGAACCGCCAGACC
TTCATCAACTCCGTCATCAAATTCCTGCGCCAGTATCAGTTTGATGGGCTGGACCTGGA
CTGGGAATACCCCGGCTCCAGGGGCAGCCCTGCCCAGGACAAGTCTCTCTTCACCGTCC
TGGTCAAGGAAATGGTGGCAGCCTTTGAGCAGGAAGCCAAGCAGACCAACAGGCCCCGG
CTCATGGTCACTGCTGCTGTTGCTGGAGGACTCTCCACCATCCAGTCTGGCTATGAGAT
CGCTGAGCTGGGCAAGTACCTGGATTACATCCACGTCATGACCTACGACTTCCATGGGC
CCTGGGACGGCTCCACGGGCGAGAACAGCCCCCTGTTCAGCAGCGGCAGCACCCTCAGT
GTTGAATACGCCATGAACTACTGGAAGAACAACGGCGCCCCAGCCCAGAAGCTGCTGGT
GGGATTCCCAACCTACGGAAAGAGCTTCACCCTGCAGAACCCATCCAACACGGCCATCG
GAGCCCCCAGCTCCGGGCCTGGCCCCGCTGGGCCCTACACCAAGGAGGCCGGGCTCCTG
GCTTACTACGAGATCTGCACCTTCCTGAGCTCTGGAGCCACCCAGGCTTGGGATGCCCC
TGAGGATGTCCCCTACGCCTACAAGGGCAGCGAATGGGTCGGCTACGACAACGAGAGGA
GCTTTGGCCTCAAGGTGGACTGGCTGAAGAAGAACAACTTTGGAGGGGCCATGGTGTGG
ACCATCGACCTGGATGACTTCACTGGCACCTTCTGCCACCAGGGCAAATACCCCCTGAT
CTCCACCCTGAAGAAGGGCCTGGGGCTG
>Agelaius_phoeniceus1
ATGTTTGTCCATCCCTGTCGGTCCCACGTTGGCAGCAGAGAGGAGCAGGGTATAAAAGA
GGGAGAGAGCCCGAGCACATCAGTCTGGTCCAAGATGGCCAAGCTCACTCTGCTCACCG
GTCTGGCCCTGCTGCTGAACGCCCAGCTCGGCACTGCCTATGTGCTGACCTGTTACTTC
ACCAACTGGGCCCAGTACAGGCCTGGCCTGGGCAAGTTCACCCCTGAAAATGTCGACCC
TTGCCTGTGCAACCACCTGATCTACGCCTTTGCCGGCATGAACAACAACGAGATCACCA
CCTACGAGTGGAACGACGAGACCCTCTACAAGTCCTTCAATGGCCTCAAGAACCAGAAC
AAAGATCTGAAGACCCTGCTGGCCATTGGAGGATGGAATTTCGGCACAGCCAAGTTCTC
CACCATGGTCTCCACGCCCCAGAACCGCCAGACCTTCATCAACTCCGTCATCAAATTCC
TGCGCCAGTATCAGTTTGATGGGCTGGACCTGGACTGGGAATACCCCGGCTCCAGGGGC
AGCCCTGCCCAGGACAAGTCTCTCTTCACCGTCCTGGTTAAGGAAATGGTGGCAGCCTT
TGAGCAGGAAGCCAAGCAGACCAACAGGCCCCGGCTCATGGTCACTGCTGCTGTTGCTG
CAGGACTCTCCACCATCCAGTCTGGCTACGAGATCGCTGAGATTGGCAAGTACCTGGAT
TACATCCACGTCATGACCTACGACTTCCACGGCTCCTGGGAGAGGAACACGGGCGAGAA
CAGCCCCCTGTTTGCTGGCCCTGCTGACAGCGGCGACTACAAATACTTCAACGTTGAAT
ACGCCATGAACTATTGGAAGAGCAATGGTGCCCCAGCTGAGAAGCTCCTTGTGGGATTC
CCAACCTATGGAAAGAGCTTCACCCTGCAGAACCCATCTGACACCTCTGTTGGGGCTCC
AGCATCCGGCCCTGGCCCCGCTGGGCCCTACACCAGGGAGGCTGGAACTCTGGCTTACT
ACGAGATCTGCTCTCTCCTGAGCTCTGGAGCCACCCAGGCTTGGGATGAACCCCAGGAT

GTCCCCTACGCCTACAAGGGCAGCGAATGGGTCGGCTACGACAACATCAAGAGCTTTGG
CCTCAAGGTGGACTGGCTGAAGAAGAACAACTTTGGAGGGGCCATGGTGTGGGCCCTGG
ACATGGATGACTTCACTGGGGATTTCTGCAAGGAAGGCAAATACCCCCTGATCTCCACC
CTGAAGAAGGGCCTGGGGCTGGAGAGTGGCGACTGCGTTCCCCCCTCTGAGCCCCTTCC
TCCCATCACTGAGGCTCCCACCACCACCAGCGGTGGCTCCGGCGGCTCCGGCGGCTCCG
GCGGCTCTGGATTCTGTGCCGGGAAACCCAACGGGATCTACGCAGACCCCAACAACAAG
AGGAACTTCTACAGCTGCCTGAACGGCCAGACCTTCGTGCAGAGCTGTGAGGAGGGGCT
GGTCTTCGACCCCGCCTGCTCCTGCTGCAACTGGCCCCAG
>Melozone_crissalis2
ATGGCCAAGCTCACTCTGCTCACGGGTCTGGCCCTGCTGCTGAACGCCCAGCTCGGCAC
TGCCTATGTGCTGACCTGTTACTTCACCAACTGGGCCCAGTACAGGCCTGGGGAGGGGA
GGTACACCCCCGAGAACATCGACCCCAACCTGTGCAACCACCTGATCTACGCCTTCGCC
GGCATGAACAACAACGAGATCACCACCTACGAGTGGAACGACGAGACCCTCTACAAGTC
CTTCAATGGCCTCAAGAACCAGAACCGCAACCTGAAGACCCTGCTGGCCATTGGAGGAT
GGAATTTCGGCACACAGAAGTTCTCCACCATGGTCTCCACGCCCCAGAACCGCCAGACC
TTCATCAACTCCGTGGTCAGGTTCCTGCGCCAGTACGGATTCGACGGGCTGGACCTGGA
CTGGGAATACCCCGGCTCCAGGGGCAGCCCTGCCCAGGACAAGTCTCTCTTCACCGTCC
TGGTTAAGGAAATGGTGGCAGCCTTCGAGCAGGAAGCCAAGCAGAGCAACAAGCCCCAG
CTCATGGTCACTGCTGCTGTGGCTGGAGGACTTTCCACCATCCAGTCTGGCTACGAGAT
TGCTGAGCTGGGCAAGTACCTGGATTACATCCACGTCATGACCTACGACTTCCACGGGC
CCTGGGACGGCTCCACGGGCGAGAACAGCCCCCTGTTCAGCAGCGGCAGCACCCTCAGT
GTTGAATACGCCATGAACTACTGGAAGAACAACGGCGCCCCAGCCCAGAAGCTGCTGGT
GGGATTCCCAACCTATGGAAAGAGCTTCACCCTGCAGAGCCCATCCAACACGGCCATTG
GTGCTCCTAGCTCCGGGCCTGGCCCTGCAGGGCCCTACACCGGGGAGGCCGGGCTCCTG
GCTTACTACGAGATCTGCACCTTCCTGAGCTCTGGAGCCACCCAGGTTTGGGATGCCCC
TGAGGATGTCCCCTACGCCTACAAGGCCAACGAGTGGGTCGGCTACGACAACGAGAAGA
GCTTCGGCCTCAAGGTGGACTGGCTGAAGAAGAACAACTTTGGAGGGGCCATGGTGTGG
ACCATCGACCTGGATGACTTCACTGGCAACTTCTGCCACCAGGGCAAATACCCCCTGAT
CTCCACGCTGAAGAAGGGCCTGGGGCTG
>Melozone_crissalis1
ATGGCCAAGCTCACTCTGCTCACCGGTCTGGCCCTGCTGCTGAACGCCCAGCTCGGCAC
TGCCTATGTGCTGACCTGTTACTTCACCAACTGGGCCCAGTACAGGCCTGGCCTGGGCA
AGTTCACCCCTGAAAATGTCGACCCTTGCCTGTGCAACCACCTGATCTACGCCTTCGCC
GGCATGAACAACAACGAGATCACCACCTACGAGTGGAACGACGAGACCCTCTACAAGTC
CTTCAATGGCCTCAAGAACCAGAACAAAGACCTGAAGACCCTGCTGGCCATTGGAGGAT
GGAATTTCGGCACAGCCAAGTTCTCCACCATGGTCTCCACTCCTGAGAACCGCCAGACC
TTCATCAAGTCCGTCATCAAATTCCTGCGCCAGTATCAGTTCGACGGGCTGGACCTGGA
CTGGGAATACCCCGGCTCCAGGGGCAGCCCTGCCCAGGACAAGTCTCTCTTCACCGTCC
TGGTTAAGGAAATGGTGGCAGCCTTCGAGCAGGAAGCCAAGCAGAGCAACAGGCCCCAG
CTCATGGTCACTGCTGCTGTGGCTGCTGGACTCTCCACCATCCAGGCTGGCTATGAGAT
CGCTGAGATTGGCAAGTACCTGGATTACATCCACGTCATGACCTACGACTTCCACGGCT
CCTGGGAGAGGAACACGGGCGAGAACAGCCCCCTGTTCGCCGGCCCTGCCGACAGCGGC
GACTACAAATACTTCAACGTTGAATACGCCATGAATTATTGGAAGAGCAATGGTGCCCC
GGCTGAGAAGCTCCTGGTGGGATTCCCAACCTATGGAAAGAGCTTCACCCTGCAGAACC
CATCTGACACCTCCGTTGGAGCTCCAGCATCCGGCCCTGGCCCCGCTGGGCCCTACACC
AGGGAGGCTGGAACTCTGGCTTACTATGAGATCTGCTCTCTGCTGAGCTCTGCAGCCAC
CCAGGCTTGGGATGAACCCCAGGATGTCCCCTACGCCTACAAGGGCAGCGAGTGGGTCG
GCTACGACAACGTCAAGAGCTTCGGCCTCAAGGTGGACTGGCTGAAGAAGAACAACTTT
GGAGGAGCCATGGTGTGGGCCCTGGACATGGATGACTTCACTGGGGATTTCTGCAAGGA
AGGCAAATACCCCCTGATCTCCAGCCTGAAGAAGGGCCTGGGGCTGCAGAGCGGCGACT
GCGTTCCCCCCTCTGAGCCCCTTCCTCCCATCACTGAGGCTCCCACCACCACCTCTGGC
GGCTCCGGCGGCTCCGGCGGCTCCGGCGGCTCTGGGTTCTGTGCCGGCAAACCCAACGG
GATCTACGCAGACCCCAACAACAAGAGGAACTTCTACAGCTGCCTGAACGGCCAGACCT
TCGTGCAGAGCTGCGAGCAGGGGCTGGTCTTCGACCCCGCCTGCTCCTGCTGCAACTGG

CCCCAG
>Vidua_macroura1
ATGGCCTCGCTCACTCTGCTCACCGGCCTGGCCGTGCTGCTGAACGCCCACCTCGGCTC
TGCCTATGTGCTGACCTGTTACTTCACCAACTGGGCCCAGTACCGGCCTGGCCTGGGCA
AGTTCAGCCCTGAGAACATCGACCCCAACCTGTGCAACCACCTGATCTACGCCTTCGCC
GGCATGAACAACAACGAGATCAGCACGTACGAGTGGAACGACGAGACCCTCTACAGGTC
CTTCAACGGCCTCAAGAGCCAGAACAAAGATCTGAAGACCCTGCTGGCCATTGGAGGAT
GGAATTTTGGCACGCAGAAGTTCTCCACCATGGTCTCCACAGCCCAGAACCGCCAGACC
TTCATCAAGTCCGTGGTCAAATTCCTGCGGCAGTACGGATTCGATGGGCTGGACCTGGA
CTGGGAATACCCCGGCTCCAGGGGCAGCCCTGCCCAGGACAAGGCTCTCTTCACCGTCC
TGGTTAAGGAGCTGCTGGCAGCCTTCGAGCAGGAGGCCAGAGAGACCAACCAGCCCCGG
CTCATGGTCACCGCCGCCGTGGCTGCCGGACTTTCCACCATCCAGGCCGGCTACGAGAT
CGCCGAGCTGGGCAAGTACCTGGATTACTTCCACGTCATGACCTACGACTTCCACGGCT
CCTGGGAGCGGAACACTGGCGAGAACAGCCCCCTGTTCGCCGGCCCTGCTGACAGCGGC
GACTACAAACACTTCAACGTGGAATACGCTATGAATTATTGGAAGAGCAACGGTGCCCC
AGCTGAGAAGCTCCTTGTGGGATTCCCAACCTATGGAAAGAGCTTCACCCTGCAGAGCC
CATCCGACACCTCCGTCGGAGCTCCAGCATCCGGCCCTGGCCCCGCTGGGCCCTACACC
AGGGAGGCCGGGCTCCTGGCTTACTACGAGATCTGCTCTCTCCTGAGTTCTGGAGCCAC
CCAGGCTTGGGATGCCCCCCAGGATGTCCCCTACGCCTACAAGGGCAGCGAATGGGTCG
GCTACGACAACGTCAAGAGCTTCGGCCTCAAGGTGGACTGGCTGAAGAAGAACAGCTTT
GGAGGGGCCATGGTGTGGGCCCTGGACATGGATGACTTCAGTGGGGATTTCTGCCACGA
GGGCAAATACCCCCTGATCTCCAGCCTGAAGAAGGGCCTGGGGCTGCAGGGCGGCGGCT
GCGTTCCCCCCGCTGAGCCCCAGCCTCCCATCACTGAGGCTCCCACCACCACCACCAGC
GGCGGCTCCGGCGGCTCCGGATTCTGCGCTGGGAAACCCAACGGGATCTACGCAGACCC
CAGCAACAGGAGGAACTTCTACAGCTGCCTGAACGGCGAGACCTTCGTGCAGAGCTGCC
AGCTGGGGCTGGTCTTTGACCCCGGCTGCTCCTGCTGCAACTGGCCCCAG
>Vidua_macroura2
ATGGCCTCGCTCACTCTGCTCACCGGCCTGGCCGTGCTGCTGAATGCCCACCTCGGCTC
TGCCTATGTGCTGACCTGTTACTTCACCAACTGGGCCCAGTACCGGCCTGGCCTGGGCA
AGTTCAGCCCTGAGAACATCGACCCCAACCTGTGCAACCACCTGATCTACGCCTTCGCC
GGCATGAACAACAACGAGATCAGCACGTACGAGTGGAACGACGAGACCCTCTACAGGTC
CTTCAACGGCCTCAAGAGCCAGAACAAAGATCTGAAGACCCTGCTGGCCATTGGAGGAT
GGAATTTCGGCACGCAGAAGTTCTCCACCATGGTCTCCACACCCCAGAACCGCCAGACC
TTCATCAAGTCCGTGGTCAAATTCCTGCGGCAGTACGGATTCGATGGGCTGGACCTGGA
CTGGGAATACCCCGGCTCCAGGGGCAGCCCTGCCCAGGACAAGGCTCTCTTCACCGTCC
TGGTTAAGGAGCTGCTGGCAGCCTTCGAGCAGGAGGCCAGAGAGACCAACCAGCCCCGG
CTCATGGTCACCGCCGCCGTGGCTGCCGGACTTTCCACCATCCAGGCCGGCTACGAGAT
CGCCGAGCTGGGCAAGTACCTGGATTACTTCCACGTCATGACCTACGACTTCCACGGCT
CCTGGGACGGCTCCACGGGCGAGAACAGCCCCCTGTTCAGCAGGGGCAGCACCCTCAGC
GTTGAATACGCCATGAATTATTGGAAGAGCAACGGTGCCCCAGCTCAGAAGCTCCTGGT
GGGATTCCCAACCTACGGAAAAGCTTCACCCTGCAGAGCCCATCCGACACCACCGTTG
GAGCTCCAGCATCCGGCCCTGGCCCTGCTGGGCCCTACACCAGGGAGGCCGGGCTCCTG
GCTTACTACGAGATCTGCTCTCTCCTGAGTTCTGGAGCCACCCAGGCTTGGGATGCCCC
CCAGGATGTCCCCTACGCCTACAAGGGCAGCGAATGGGTCGGCTACGACAACGTCAAGA
GCTTCGGCCTCAAGGTGGACTGGCTGAAGAAGAACGACTTTGGAGGGGCCATGGTGTGG
GCCCTGGACATGGATGACTTCAGTGGGGACTTCTGCAAGGAGGGCAAATACCCCCTGAT
CTCCAGCCTGAAGAAGGGCCTGGGGCTG
>Vidua_chalybeata2
ATGGCCTCGCTCACTCTGCTCACCGGCCTGGCCGTGCTGCTGAACGCCCACCTCGGCTC
TGCCTATGTGCTGACCTGTTACTTCACCAACTGGGCCCAGTACCGGCCTGGCCTGGGCA
AGTTCAGCCCTGAGAACATCGACCCCAACCTGTGCAACCACCTGATCTACGCCTTCGCC
GGCATGAACAACAACGAGATCAGCACGTACGAGTGGAACGACGAGACCCTCTACAGGTC
CTTCAACGGCCTCAAGAGCCAGAACAAAGATCTGAAGACCCTGCTGGCCATTGGAGGAT
GGAATTTCGGCACGCAGAAGTTCTCCACCATGGTCTCCACACCCCAGAACCGCCAGACC

TTCATCACGTCCGTGGTCAAATTCCTGCGGCAGTACGGATTCGATGGGCTGGACCTGGA
CTGGGAATACCCCGGCTCCAGGGGCAGCCCTGCCCAGGACAAGGCTCTCTTCACCGTCC
TGGTTAAGGAGCTGCTGGCAGCCTTCGAGCAGGAGGCCAGAGAGACCAACCAGCCCCGG
CTCATGGTCACCGCCGCCGTGGCTGCCGGACTTTCCACCATCCAGGCCGGCTACGAGAT
CGCCGAGCTGGGCAAGTACCTGGATTACTTCCACGTCATGACCTACGACTTCCACGGCT
CCTGGGACGGCTCCACGGGCGAGAACAGCCCCCTGTTCAGCAGGGGCAGCACCCTCAGC
GTTGAATACGCCATGAATTATTGGAAGAGCAACGGTGCCCCAGCTGAGAAGCTGCTGGT
GGGATTCCCAACCTATGGAAAGAGCTTCACCCTGCAGAACCCATCCGACACTGCCGTTG
GAGCGCCCAGCTCCGGGCCAGGCCCTGCTGGGCCTTACACCAGGGAGGCCGGAACTCTG
GCTTACTACGAGATCTGCTCTCTCCTGGGTTCTGGAGCCACCCAGGCTTGGGATGCCCC
CCAGGATGTCCCCTACGCCTACAAGGGCAGCGAATGGGTCGGCTACGACAACGTCAAGA
GCTTCGGCCTCAAGGTGGACTGGCTGAAGAAGAACAACTTTGGAGGGGCCATGGTGTGG
GCCCTGGACATGGATGACTTCAGTGGGGATTTCTGCCACGAGGGCAAATACCCCCTGAT
CTCCAGCCTGAAGAAGGGCCTGGGGCTG
>Vidua_chalybeata1
ATGGCCTCGCTCACTCTGCTCACCGGCCTGGCCGTGCTGCTGAACGCCCACCTCGGCTC
TGCCTATGTGCTGACCTGTTACTTCACCAACTGGGCCCAGTACCGGCCTGGCCTGGGCA
AGTTCAGCCCTGAGAACATCGACCCCAACCTGTGCAACCACCTGATCTACGCCTTCGCC
GGCATGAACAACAACGAGATCAGCACGTACGAGTGGAACGACGAGACCCTCTACAGGTC
CTTCAATGGCCTCAAGAGCCAGAACAAAGATCTGAAGACCCTGCTGGCCATTGGAGGAT
GGAATTTCGGCACGCAGAAGTTCTCCACCATGGTCTCCACACCCCAGAACCGCCAGACC
TTCATCAAGTCCGTGGTCAAATTCCTGCGGCAGTACGGATTCGATGGGCTGGACCTGGA
CTGGGAATACCCCGGCTCCAGGGGCAGCCCTGCCCAGGACAAGGCTCTCTTCACCGTCC
TGGTTAAGGAGCTGCTGGCAGCCTTCGAGCAGGAGGCCAGAGAGACCAACCAGCCCCGG
CTCATGGTCACCGCCGCCGTGGCTGCCGGACTTTCCACCATCCAGGCCGGCTACGAGAT
CGCCGAGCTGGGCAAGTACCTGGATTACTTCCACGTCATGACCTACGACTTCCACGGCT
CCTGGGAGCGGAACACGGGCGAGAACAGCCCCCTGTTCGCCGGCCCTGCTGACAGCGGC
GACTACAAATACTTCAACGTGGAATACGCCATGAATTATTGGAAGAGCAATGGTGCCCC
AGCTGAGAAGCTCCTGGTGGGATTCCCAACCTATGGAAAGAGCTTCACCCTGCAGAACC
CATCCGACACCTCCGTTGGAGCTCCGGCATCCGGCCCTGGCCCCGCCGGGCCTTACACC
AGGGAGGCCGGAACTCTGGCTTACTACGAGATCTGCTCTCTCCTGGGTTCTGGAGCCAC
CCAGGCTTGGGATGCCCCCCAGGATGTCCCCTACGCCTACAAGGGCAGCGAATGGGTCG
GCTACGACAACGTCAAGAGCTTCGGCCTCAAGGTGGACTGGCTGAAGAAGAACGGCTTT
GGAGGGGCCATGGTGTGGGCCCTGGACATGGATGACTTCAGTGGGGATTTCTGCCACGA
GGGCAAATACCCCCTGATCTCCAGCCTGAAGAAGGGCCTGGGGCTGCAGGGCGGCGGCT
GCGCTCCCCCCGCTGAGCCCCAGCCTCCCATCACTGAGGCTCCCACCACCACCGCCAGC
GGCGGCTCCGGATTCTGCGCCGGGAAACCCAACGGGATTTACGCAGACCCCAGCAACAA
GAGGAACTTCTACAGCTGCCTGAACGGCGATACCTTCGTGCAGAGCTGCCAGCTGGGGC
TGGTCTTCGACCCCGGCTGCTCCTGCTGCAACTGGCCCCAG
>Manacus_candei1
ATGGCCAAGCTCACTCTGCTCACCGGTCTGGCCCTGCTGCTCAACGCCCACCTCGGCAC
TGCCTACGTGCTGACATGTTACTTCACCAACTGGGCCCAGTACAGGCCTGGGGAGGGGA
AATACACCCCCGAGAACATCGACCCCAACCTGTGCAGCCACCTGATCTACGCCTTCGCC
GGGATGAACAACAATGAGATCACCACTTATGAATGGAACGACGAGACCCTCTACAAATC
CTTCAACGGCCTGAAGAACCAGAACAGAAACCTGAAGACCCTGCTGGCCATTGGAGGAT
GGAATTTCGGCACAGAAAAGTTCACTACGATGGTTTCCACCCCCCAGAACCGCCAGACT
TTCATCAACTCCGTTGTCAGATTCCTGCGCCAGTATGGATTCGATGGGCTGGACCTGGA
CTGGGAATACCCCGGCTCCAGGGGCAGCCCAGCCCAGGACAAGGGGCTCTTCACTGTCC
TGGTTAAGGAATTGCTGGCAGCCTTTGAGCAGGAAGCCAAACAGACCAACCAGCCCCGG
CTCCTGGTCACCGCGGCTGTGGCTGCAGGACTTTCCACCATCCAGGCTGGCTACGAGAT
TGCTGAGCTGGGCAAGTACCTGGACTACATCCACGTGATGACCTACGACTTCCACAGCC
CCTGGGACGGCTCCACTGGCGAGAACAGCCCCCTGTTCAGCAGCGGCAGCACCTTCAGT
GTGGAATACTCTATGAACTACTGGAAGAACAATGGTGCCCCAGCTGAGAAGCTCCTGGT
TGGCTTCCCAACCTACGGAAAGACCTTCACCCTGCAAAACCCCTCCAACACTGGGATTG

GGGCCCCAACCTCGGGCCCTGGGCCAGCAGGACCCTACACCAGGGAGGCCGGGCTTCTG
GCTTACTACGAGATCTGCTCGTTCCTGAACACCGGAGCCACCCAGTCTTGGGATGCCCC
TGAGGACGTGCCCTATGCCTACAAGGGCAACGAGTGGATTGGCTACGACAACACGAAGA
GCTTCAGTGCAAAGATCGACTGGCTGAAGCAGAACAACTTTGGAGGGGCCATGGTTTGG
ACCATCGACCTGGATGACTTCACTGGCACTTTCTGCCATGAAGGCAAATACCCCCTGAT
CTCCACCCTGAAGAAGGGCCTTGGTTTG
>Manacus_candei2
ATGGCCAAGCTCACTCTGCTCACCGGTCTGGCCCTGCTGCTCAACGCCCACCTCGGCAC
TGCCTACGTGCTGACATGTTACTTCACCAACTGGGCCCAGTACAGGCCTGGCCTGGGCA
AATACACACCCGAAAATGTCGACCCCTGCCTGTGCAGCCACCTGATCTACGCCTTCGCC
GGGATGAACAACAACGAGATCACCACTTATGAATGGAACGACGAGACCCTCTACAAATC
CTTCAACGGCCTGAAGAACCAGAACAAGGATCTGAAGACACTCCTGGCGATTGGAGGAT
GGAATTTTGGCACAGCCAAGTTCACTACGATGGTTTCCTCTGCTGAGAACCGCCAGACC
TTCATCAATTCTGTCATCAAATTCCTGCGCCAGTACCGGTTTGATGGGCTGGACCTGGA
CTGGGAATACCCCGGCTCCAGGGGCAGCCCAGCCCAGGACAAGGGGCTCTTCACTGTCC
TGGTTAAGGAATTGCTGGCAGCCTTTGAGCAGGAAGCCAAACAGACCAACCAGCCCCGG
CTCCTGGTCACCGCGGCTGTGGCTGGAGGACTTTCCACCATCCAGGCTGGCTACGAGAT
TGCTGAGCTGGGCAAGTACCTGGACTACATCCACGTGATGACCTACGACTTCCACGGCT
CCTGGGAGAGAAACACTGGCGAGAACAGCCCCCTGTTCACAGGCCCAGCTGACACTGGG
GACTTCAAGTACTTCAACGTCGAATATGCTATGAATTACTGGAAGGACAATGGTGCCCC
AGCTGAGAAGCTCCTTGTTGGCTTCCCAACCTACGGAAAAAGCTTCACCCTGCAAAACC
CATCTGACACCTCTGTTGGGGCTCCAGCATCAGGCCCTGGACCAGCTGGACCTTACACC
AGGGAGGCTGGGACACTGGCTTACTATGAGATCTGCTCTCTCCTGAGCTCTGGAGCCAC
CCAGGCTTGGGATGAACCCCAGGACGTGCCCTATGCCTACAAGGAGAGCGAATGGGTTG
GCTATGATAACACAAAGAGCTTCAGCATCAAGGTCGACTGGCTGAAGAAGAATAACTTT
GGAGGGGCCATGGTTTGGGCCCTCGACATGGATGATTTCACTGGCACTTTCTGCAATGA
AGGCAAATACCCCCTGATCTCCACCCTGAAGAAGGGCCTTGGTCTGCAGAACGGTGACT
GTGTGCCTCCTGCTGAGCCCCTGCCTCCAGTCACTGAGGCCCCCACTACCACCAGTGGA
AGTGGGAGCAGTGGCTCTGGTGGGAGTGGTGGCTCTGGTGGGAGCGGCTTCTGCACCGG
CAAAGCCAACGGCATCTACGCAGACCCAGAGAACAGCAAGAACTTCTACAACTGCTTGA
ACGGCCAGACCTTCGTGCAGAGCTGCCAACAGGGCCTCGTCTTCGACCCCGTCTGCTCC
TGCTGCAACTGGCCA
>Myiozetetes_cayanensis2
ATGGCCAAGCTCACTCTGCTCACCGGTCTGGCCCTGCTGCTCAACGCCCACCTCAGCAC
TGCCTACGTGCTGACATGTTACTTCACCAACTGGGCCCAGTACAGGCCTGGCCTGGGCA
AATACACACCCGAAAATGTTGACCCTTGCCTGTGCAACCACCTGATCTACGCCTTCGCC
GGGATGAACAACAACGAGATCACCACTTACGAATGGAACGACGAGACCCTCTACAAATC
CTTCAACGGCCTGAAGAACCAGAACAAAGATCTGAAGACACTCCTGGCTATTGGAGGAT
GGAATTTTGGCACACAGAAGTTCACTACAATGGTTTCCTCTGCTGACAACCGCCAGACC
TTCATCAAGTCTGTCATCAAATTCCTGCGCCAGTACCAGTTTGATGGGCTGGACCTGGA
CTGGGAATACCCTGGCTCCAGGGGCAGCCCAGCCCAGGACAAGGGGCTCTTCACCGTCC
TGGTTAAGGAACTGCTGGCAGCCTTTGAGCAGGAAGCCAAACAGACCAACCAGCCCCGT
CTCTTGGTCACTGCGGCTGTGGCTGCAGGACTTTCCACCATCCAGGCTGGCTACGAGAT
TGCTGAGATTGGGAAGTACCTGGACTACATCCACGTCATGACCTACGACTTCCATGGCT
CCTGGGAGAGGAACACTGGTGAGAACAGCCCCCTGTACGCAGGCCCAGCTGACACCGGT
GACTACAAGTACTTCAATGTCGAATATGCTATGAATTACTGGAAGGACAACGGTGCCCC
AGCTGAGAAGCTCCTTGTTGGCTTCCCAACCTATGGAAAAAGCTTCACCCTGCAGAACC
CATCTGACACCTCTGTTGGGGCTCCAGCATCCGGCCCTGGACCAGCTGGACCTTACACC
AGGGAGGCTGGAACACTGGCTTACTATGAGATCTGCTCTCTCTTGAGCTCTGGAGCCAC
TCAGGCTTGGGATGAACCCCAGGACGTGCCCTATGCCTACAAGGAGAGCGAGTGGGTTG
GCTATGACAACATGAAGAGCTTCAGCATCAAGGTTGACTGGCTGAAGAAGAACAACTTT
GGAGGCGCCATGGTCTGGGCCCTTGACATGGATGACTTCACTGGCACTTTCTGCAATGA
AGGCAAATACCCCCTGATCTCCACCCTGAAGAAGGGCCTTGGTCTGCAGAACGGCGACT
GTGTGCCTCCCGCTGAGCCCCTGCCTCCAATCACTGAGGCTCCCACTACCACAAGTGGC

AGCGGGAGCGGCGGCTCTGGCGGGAGCGGCGGCTCTGGCGGGAGCGGCTTCTGCGCCGG
CAAAGCCAACGGCATCTACGCGGACCCAGAGAACAACAGGAACTTCTACAACTGCTTGA
ACGGCCAGACCTTCGTGCAGAGCTGCCAACAGGGGCTCGTCTTCGACCCCCTCTGCTCC
TGCTGCAACTGGCCA
>Myiozetetes_cayanensis1
ATGGCCAAGCTCACTCTGCTCACCGGTCTGGCCCTGCTGCTCAACGCCCACCTCAGTAG
CCTTGCCAGCAGCTCTGCTCCTGATGCCCTGCCTGAAGCCACTGGTGCCTCTGGAGCAG
CCCCCCAGCTCCTCTGTCTCCCCACAGGCACTGCCTACGTGCTGACATGTTACTTCACC
AACTGGGCCCAGTACAGGCCTGGGGAGGGGAAATACACCCCTGACAACATCGACCCCAA
CCTGTGCAGCCACCTGATCTACGCCTTTGCCGGGATGAACAACAACGAGATCACCACTT
ACGAATGGAACGACGAGACCCTCTACAAATCCTTCAACGGCCTGAAGAACCAGAACAGA
AACCTGAAGACCCTGCTGGCCATTGGAGGATGGAATTTCGGCACACAGAAGTTCACTAC
AATGGTTTCCACCCCCCAGAACCGCCAGACCTTCATCACCTCTGTTGTCAGATTCCTGC
GCCAGTATGGATTCGATGGGCTGGACCTGGACTGGGAATACCCCGGCTCCAGGGGCAGC
CCAGCCCAGGACAAGGGGCTCTTCACCGTCCTGGTTAAGGAACTGCTGGCAGCCTTTGA
GCAGGAAGCCAGGCAGACCAACAAGCCCCGTCTCTTGGTCACCGCGGCTGTGGCTGCAG
GACTTTCCACCATCCAGGCCGGCTACGAGATTGCTGAGCTGGGGAAGTACCTGGACTAC
ATCCACGTGATGACCTACGACTTCCACAGCCCCTGGGATGGCTCCACTGGCGAGAACAG
CCCCCTGTTCAGCAGCGGCACTCTCAGTGTGGAATATGCCATGAACTACTGGAAGAACA
ACGGTGCCCCAGCTCAGAAGCTGCTGGTTGGCTTCCCAACCTACGGCAAGACCTTCACC
CTGCAGAACCCCTCCAACAACGGCATTGGGGCCCCAAGCTCGGGCCCTGGGCCAGCAGG
ACCTTACACCGGGGAGGCCGGGCTTTTGGCTTACTATGAGATCTGCACATTCCTGAACA
CCGGAGCCACCCAGGCTTGGGATGCCCCTGAGGACGTGCCCTATGCCTACAAGGGCAAC
GAGTGGATTGGCTACGACGACGTGAAGAGCTTCAACATCAAGGTTGACTGGCTGAAGCA
GAACAACTTTGGAGGGGCCATGGTTTGGACCATCGACCTGGATGACTTCACTGGCAATT
TCTGCCACGAAGGCAAATACCCCCTGATCTCCACCCTTAAGAAGGGCCTTGGTCTG
>Lepidothrix_coronata1
ATGAAGGTGACTTCCCACCACTGGTGTCTGAGGAGCAGCAGGCAGCCCCCCCAGCTCCT
GTGTCTCCCCACAGGCACTGCCTACGTGCTGACATGTTACTTCACCAACTGGGCCCAGT
ACAGGCCTGGGGAGGGGAAATACACCCCCGAGAACATCGACCCCAACCTGTGCAGCCAC
CTGATCTACGCCTTCGCCGGGATGAACAACAACGAGATCACCACTTATGAATGGAACGA
CGAGACCCTCTACAAATCCTTCAACGGCCTGAAGAACCAGAACAGAAACCTGAAGACCC
TGCTGGCCATTGGAGGATGGAATTTCGGCACAGAAAAGTTCACTACGATGGTTTCCACC
CCCCAGAACCGCCAGACTTTCATCAACTCCGTTGTCAGATTCCTGCGCCAGTATGGATT
CGATGGGCTGGACCTGGACTGGGAATACCCCGGCTCCAGGGGCAGCCCAGCCCAGGACA
AGGGGCTCTTCACTGTCCTAGTTAAGGAATTGCTGGTAGCCTTTGAGCAGGAAGCCAAA
CAGACCAACCAGCCCCGTCTCCTGGTCACCGCGGCTGTGGCTGGAGGACTTTCCACCAT
CCAGGCTGGCTACGAGATTGCTGAGCTGGGCAAGTACCTGGACTACATCCACGTGATGA
CCTACGACTTCCACAGCCCCTGGGACGGCTCCACTGGCGAGAACAGCCCCCTGTTCAGC
AGCGGCAGCACCTTCAGTGTGGAATACGCTATGAACTACTGGAAGAACAATGGTGCCCC
AGCTCAGAAGCTCCTGGTTGGCTTCCCAACCTACGGAAAGACCTTCACCCTGCAAAACC
CCTCCAACACTGGGATTGGGGCCCCAACCTCGGGCCCTGGGCCAGCAGGACCTTACACC
AGGGAGGCCGGGCTTCTGGCTTACTATGAGATCTGCTCGTTCCTGAACACCGGAGCCAC
CCAGGCTTGGGATGCCCCTGAGGACGTGCCCTATGCCTACAAGGGCAACGAGTGGATTG
GCTACGACAACACGAAGAGCTTCAGTGCAAAGGTCGACTGGCTGAAGCAGAACAACTTT
GGAGGGGCCATGGTTTGGACCATCGACCTGGATGACTTCACTGGCACTTTCTGCCATGA
AGGCAAATACCCCCTGATCTCCACCCTGAAGAAGGGCCTCGGTCTG
>Lepidothrix_coronata2
ATGCTCAACTCCCGCCTCGGCACTGCCTACGTGCTGACATGTTACTTCACCAACTGGGC
CCAGTACAGGCCTGGCCTGGGCAAATACACCCCCGAAAATGTCGACCCCTGCCTGTGCA
ACCACCTGATCTATGCCTTTGCCGGGATGAACAACAACGAGATCACCACTTATGAATGG
AACGACGAGACCCTCTACAAATCCTTCAACGGCCTGAAGAACCAGAACAAGGATCTGAA
GACACTCCTGGCCATTGGAGGATGGAATTTTGGCACAGCTAAGTTCACTACAATGGTTT
CCTCTGCTGAGAACCGGCAGACCTTCATCAAGTCTGTCATCAAATTCCTGCGCCAGTAC

CAGTTTGATGGGCTGGACCTGGACTGGGAATACCCCGGCTCCAGGGGCAGCCCAGCCCA
GGACAAGGGGCTCTTCACCGTCCTGGTTAAGGAATTGCTGGCAGCCTTTGAGCAGGAAG
CCAAACAGACCAACCAGCCCCGTCTCCTGGTCACCGCGGCTGTGGCTGGAGGACTTTCC
ACCATCCAGGCCGGCTACGAGATTGCTGAGCTGGGCAAGTACCTGGACTACATCCACGT
GATGACCTACGACTTCCACGGCTCCTGGGAGAAAAACACTGGCGAGAACAGCCCCCTGT
TCACAGGCCCAGCTGACACTGGGGACTACAAGTACTTCAATGTCGAATACGCTATGAAT
TACTGGAAGGACAATGGTGCCCCAGCTGAGAAGCTCCTGGTTGGCTTCCCAACCTATGG
AAAAAGCTTCACCCTGCAAAACCCATCTGACACCTCTGTTGGGGCTCCAGCATCAGGCC
CTGGACCAGCTGGACCTTACACCAGGGAGGCTGGGACACTGGCTTACTATGAGATCTGC
TCTCTCCTGAGCTCTGGAGCCACTCAGGCTTGGGATGAACCCCAGGACGTGCCCTATGC
CTACAAGGAGAGCGAATGGGTTGGCTATGACAACACGAAGAGCTTCAGCATCAAGGTCG
ACTGGCTGAAGAAGAATAACTTTGGAGGGGCCATGGTTTGGGCCCTTGACATGGATGAC
TTCACTGGCACTTTCTGCAATGAAGGCAAATACCCCCTGATCTCCACCCTGAAGAAGGG
CCTCGGTCTGCAGAACGGCGACTGTGTGCCTCCTGCTGAGCCCCTGCCTCCAGTCACTG
AGGCTCCCACTACCACCAGTGGAAGCGGGAGCGGTGGCTCTGGTGGGAGCGGCGGCTCT
GGTGGGAGCGGCTTCTGCGCCGGCAAAGCCAACGGCATCTACGCAGACCCAGAGAACAA
CAAGAACTTCTACAACTGCTTGAACGGCCAAACCTTCGTGCAGAGCTGCCAACAGGGTC
TCGTCTTCGACCCCGTCTGCTCCTGCTGCAACTGGCCA
>Corvus_brachyrhynchos2
ATGGCCAAGCTCACTCTGCTCACCGGCCTGGCCCTGCTGCTGAACGCCCACGTCGGCAC
TGCCTATGTGCTGACCTGTTACTTCACCAACTGGGCCCAGTACAGGCCTGGCCTGGGCA
AATACACCCCTGAGAACATCGACCCCAACCTGTGCAACCACCTGATCTACGCCTTCGCC
GGCATGAACAACAACGAGATCACCACCTACGAGTGGAACGATGAGACCCTGTACAAGTC
CTTCAACGGCCTCAAGAACCAGAACAGGAACCTGAAGACCCTTCTGGCCATTGGAGGAT
GGAATTTCGGCACAGAGAAGTTCACCACCATGGTTTCCACACCCCAGAACCGCCAGACC
TTCATCAACTCCGTGGTCAGGTTCCTGCGCCAGTACGGATCCCGCCCCCGGCTCATGGC
CCCCGCCGCTGTGGCCGGAGGACTTTCCACCATCCAAGCTGGCTATGAGATTGCTGAGT
TGGGCAAGTACCTGGATTACATCCACGTGATGACCTACGACTTCCACGGGCCCTGGGAC
GGCTCCACGGGCGAGAACAGCCCCCTGTTCAGTAGCGGCACCCTCAGTGTTGAATACGC
CATGAACTACTGGAAGAACAACGGCGCCCCAGCTCAGAAGCTCCTGGTGGGATTCCCAA
CCTACGGGAAAACCTTCACCCTGCAGAACCCATCCAACACCGGCATCGAGGCCCCAACC
TCCGGCCCTGGTCCTGCGGGACCCTACACCAAGGAGGCCGGGCTCTTGGCTTACTACGA
GATCTGCACCTTCCTGAACTCCGGAGCCACCCAGGCTTGGGATGAACCCCAGGACGTGC
CCTACGCCTACAAGGGCAGCGAATGGATCGGCTACGACAACGTCAAGAGCTTCGGCCTC
AAGGTGGATTGGCTGAAGAAGAACAACTTTGGCGGGGCCATGGTCTGGACCATCGACCT
GGATGACTTCACCGGCACCTTCTGCCACGAGGGCAAATACCCCCTGATCTCCACGCTGA
AGAAGGGCCTGGGGCTG
>Corvus_hawaiiensis1
ATGGCCAAGCTCACTCTGCTCACCGGCCTGGCCCTGCTGCTGAACGCCCACGTCGGCAC
TGCCTATGTGCTGACCTGTTACTTCACCAACTGGGCCCAGTACAGGCCTGGCCTGGGCA
AGTACACCCCCGAAAATGTCGACCCCTGCCTGTGCAACCACCTGATCTACGCCTTCGCC
GGCATGAACAACAACGAGATCACCACCTACGAGTGGAACGATGAGACCCTGTACAAGTC
CTTCAATGGCCTCAAGAACCAGAACAAAGACCTGAAGACCCTGCTGGCCATTGGAGGAT
GGAATTTTGGCACAGAGAAGTTCACCACCATGGTTTCCACACCCCAGAACCGCCAGACC
TTCATCAACTCCGTGGTCAGGTTCCTGCGCCAGTACGGATTCGACGGGCTGGACCTGGA
CTGGGAATACCCCGGCTCCAGGGGCAGCCCAGCCCAGGACAAGGGTCTCTTCACCGTCC
TGGTTAAGGAACTGCTGGCAGCCTTCGAGCAGGAAGCCAAACAGACCAGCCGGCCCCGG
CTCATGGTCACCGCCGCTGTGGCTGCCGGACTTTCCACCATCCAGGCTGGCTATGAGAT
TGCTGAGCTGGGCAAGTACCTGGATTACATCCACGTGATGACCTACGACTTCCACGGCT
CCTGGGAGAGGAACACTGGTGAGAACAGCCCCCTGTTTGCTGGCCCTGCTGACACTGGG
GACTACAAATACTTCAACGTTGAATATGCCATGAATTATTGGAAGAGCAACGGTGCCCC
AGCTGAGAAGCTCCTGGTGGGATTCCCAACCTATGGAAAGAGTTTCACCCTGCAGAACC
CATCTGACACCTCTGTTGGAGCTCCAGCGTCCGGCCCTGGCCCAGCTGGGCCCTATACC
AGGGAGGCCGGAACCCTGGCTTACTACGAGATCTGCACCTTCCTGAGCTCTGGAGCCAC

CCAGGCTTGGGATGAACCCCAGGACGTGCCCTACGCCTACAAGGACAGCGAATGGGTCG
GCTACGACAACATCAAGAGCTTCGGCCTCAAGGTGGATTGGCTGAAGAAGAACAACTTT
GGTGGGGCCATGGTCTGGGCCCTGGACATGGATGACTTCACCGGCACCTTCTGCCACGA
GGGCAAATACCCCCTGATCTCCACGCTGAAGAAGGGCCTGGGGCTGGAGAGCGGCGACT
GTGTTCCCCCCGCTGAGCCCCTGCCTCCCATCACCGAGGCTCCCACCACCACCACCACC
AGCGGCGGCTCCGGCGGCTCCGGCGGCAGCGGCTTCTGCGCCGGGAAACCCAACGGGAT
CTATTCAGACCCCGACAACAAGAGGAACTTCTACAGCTGCGTGAACGGGCAGACCTTCG
TGCAGAGCTGCGAGCAGGGGCTGGTCTTCGACCCCGTCTGCACCTGCTGCAACTGGCCC
CAG
>Corvus_hawaiiensis2
ATGGCCAAGCTCACTCTGCTCACCGGCCTGGCCCTGCTGCTGAACGCCCACGTCGGCAC
TGCCTATGTGCTGACCTGTTACTTCACCAACTGGGCCCAGTACAGGCCTGGCGAGGGCA
AATACACCCCTGAGAACATCGACCCCAACCTGTGCAACCACCTGATCTACGCCTTCGCC
GGCATGAACAACAACGAGATCACCACCTACGAGTGGAACGATGAGACCCTGTACAAGTC
CTTCAACGGCCTCAAGAACCAGAACAGGAACCTGAAGACCCTTCTGGCCATTGGAGGAT
GGAATTTTGGCACAGAGAAGTTCACCACCATGGTTTCCACACCCCAGAACCGCCAGACC
TTCATCAACTCCGTGGTCAGGTTCCTGCGCCAGTACGGATTCGACGGGCTGGACCTGGA
CTGGGAATACCCCGGCTCCAGGGGCAGCCCAGCCCAGGACAAGGGTCTCTTCACCGTCC
TGGTTAAGGAACTGCTGGCAGCCTTCGAGCAGGAAGCCAAACAGACCAGCCGGCCCCGG
CTCATGGTCACCGCCGCTGTGGCTGGAGGACTTTCCACCATCCAAGCTGGCTATGAGAT
TGCTGAGCTGGGCAAGTACCTGGATTACATCCACGTGATGACCTACGACTTCCACGGGC
CCTGGGACGGCTCCACGGGCGAGAACAGCCCCCTGTTCAGTAGCGGCACCCTCAGTGTT
GAATACGCCATGAACTACTGGAAGAACAACGGTGCCCCAGCTCAGAAGCTCCTGGTGGG
ATTCCCAGCCTACGGGAAAACCTTCACCCTGCAGAACCCATCCAACACCGGCATCGAGG
CCCCAGCCTCCGGCCCTGGTCCTGCGGGACCCTACACCAGGGAGGCCGGGCTCTTGGCT
TACTACGAGATCTGCACCTTCCTGAACTCCGGAGCCACCCAGGCTTGGGATGAACCCCA
GGACGTGCCCTACGCCTACAAGGGCAGCGAATGGATCGGCTACGACAACGTCAAGAGCT
TCGGCCTCAAGGTGGATTGGCTGAAGAAGAACAACTTTGGCGGGGCCATGGTCTGGACC
ATCGACCTGGATGACTTCACCGGCACCTTCTGCCACGAGGGCAAATACCCCCTGATCTC
CACGCTGAAGAAGGGCCTGGGGCTG
>Corvus_kubaryi2
ATGGCCAAGCTCACTCTGCTCACCGGCCTGGCCCTGCTGCTGAACGCCCACGTCGGCAC
TGCCTATGTGCTGACCTGTTACTTCACCAACTGGGCCCAGTACAGGCCTGGCCTGGGCA
AGTACACCCCCGAAAATGTTGACCCTTGCCTGTGCAACCACCTGATCTACGCCTTCGCC
GGCATGAACAACAACGAGATCACCACCTACGAGTGGAACGATGAGACCCTGTACAAGTC
CTTCAACGGCCTCAAGAACCAGAACAAAGACCTGAAGACCCTGCTGGCCATTGGAGGAT
GGAATTTTGGCACAGCCAAGTTCACCACCATGGTTTCCACACCCCAGAACCGCCAGACC
TTCATCAACTCCGTGGTCAGGTTCCTGCGCCAGTACGGATTCGACGGGCTGGACCTGGA
CTGGGAATACCCCGGCTCCAGGGGCAGCCCAGCCCAGGACAAGGGTCTCTTCACCGTCC
TGGTTAAGGAACTGCTGGCAGCCTTCGAGCAGGAAGCCAAACAGACCAGCCGGCCCCGG
CTCATGGTCACCGCCGCTGTGGCCGGAGGACTTTCCACCATCCAGGCTGGCTACGAGAT
TGCTGAGCTGGGCAAGTACCTGGATTACATCCACGTGATGACCTACGACTTCCACGGGC
CCTGGGACGGCTCCACGGGCGAGAACAGCCCCCTGTTCAGTAGCGGCACCCTCAGTGTT
GAATACGCCATGAACTACTGGAAGAACAATGGCGCCCCAGCTCAGAAGCTCCTGGTGGG
ATTCCCAACCTACGGGAAAACCTTCACCCTGCAGAACCCATCCAACACCGGCATCGAGG
CCCCAACCTCCGGCCCTGGTCCTGCGGGACCCTACACCAGGGAGGCCGGGCTCTTGGCT
TACTACGAGATCTGCACCTTCCTGAACTCTGGAGCCACCCAGGCTTGGGATGAACCCCA
GGACGTGCCCTACGCCTACAAGGGCAGCGAATGGATCGGCTACGACAACGTCAAGAGCT
TCGGCCTCAAGGTGGATTGGCTGAAGAAGAACAACTTTGGCGGGGCCATGGTCTGGACC
ATCGACCTGGATGACTTCACCGGCACCTTCTGCCACGAGGGCAAATACCCCCTGATCTC
CACGCTGAAGAAGGGCCTGGGGCTG
>Corvus_kubaryi1
ATGGCCAAGCTCACTCTGCTCACCGGCCTGGCCCTGCTGCTGAACGCCCACGTCGGCAC
TGCCTATGTGCTGACCTGTTACTTCACCAACTGGGCCCAGTACAGGCCTGGCGAGGGCA

AATACACCCCTGAGAACATCGACCCCAACCTGTGCAACCACCTGATCTACGCCTTCGCC
GGCATGAACAACAACGAGATCACCACCTACGAGTGGAACGATGAGACCCTGTACAAGTC
CTTCAACGGCCTCAAGAACCAGAACAGGAACCTGAAGACCCTGCTGGCCATTGGAGGAT
GGAATTTCGGCACAGAGAAGTTCACCACCATGGTTTCCACACCCCAGAACCGCCAGACC
TTCATCAACTCCGTGGTCAGGTTCCTGCGCCAGTACGGATTCGACGGGCTGGACCTGGA
CTGGGAATACCCCGGCTCCAGGGGCAGCCCAGCCCAGGACAAGGGTCTCTTCACCGTCC
TGGTTAAGGAACTGCTGGCAGCCTTCGAGCAGGAAGCCAAACAGACCAACCGGCCCCGG
CTCATGGTCACCGCCGCTGTGGCTGCCGGACTTTCCACCATCCAGGCTGGCTACGAGAT
TGCTGAGATTGGCAAGTACCTGGATTACATCCACGTGATGACCTACGACTTCCACGGCT
CCTGGGAGAGGAACACTGGTGAGAACAGCCCCCTGTTTGCTGGCCCTGCTGACACTGGG
GACTACAAATACTTCAACGTTGAATATGCCATGAATTATTGGAAGAGCAACGGTGCCCC
AGCTGAGAAGCTCCTGGTGGGATTCCCAACCTATGGAAAGAGTTTCACCCTGCAGAACC
CATCCGACACCTCCGTTGGAGCTCCAGCGTCCGGCCCTGGCCCAGCTGGGCCCTACACC
AGGGAGGCCGGAACCCTGGCTTACTACGAGATCTGCACCTTCCTGAGCTCTGGAGCCAC
CCAGGCTTGGGATGAACCCCAGGACGTGCCCTACGCCTACAAGGGCAGCGAATGGATCG
GCTACGACAACGTCAAGAGCTTCGGCCTCAAGGTGGATTGGCTGAAGAAGAACAACTTT
GGCGGGGCCATGGTCTGGGCCCTGGACATGGATGACTTCACCGGCACCTTCTGCCACGA
GGGCAAATACCCCCTGATCTCCACGCTGAAGAAGGGCCTGGGGCTGGAGAGCGGCGACT
GTGTTCCCCCCGCTGAGCCCCTGCCCCCCATCACCGAGGCTCCCACCACCACCACCACC
AGCGGCGGCTCCAGCGGCTCCGGCGGCAGCGGCTTCTGCGCCGGGAAACCCAACGGGAT
CTACGCAGACCCCGACAACAAGAGGAACTTCTACAGCTGCGTGAACGGGCAGACCTTCG
TGCAGAGCTGCGAGCAGGGGCTGGTCTTCGACCCCGTCTGCTCCTGCTGCAACTGGCCC
CAG
>Corvus_moneduloides2
ATGGCCAAGCTCACTCTGCTCACCGGCCTGGCCCTGCTGCTGAACGCCCACGTCGGCAC
TGCCTATGTGCTGACCTGTTACTTCACCAACTGGGCCCAGTACAGGCCTGGCCTGGGCA
AGTACACCCCCGAAAATGTCGACCCCTGCCTGTGCAACCACCTGATCTACGCCTTCGCC
GGCATGAACAACAACGAGATCACCACCTACGAGTGGAACGATGAGACCCTGTACAAGTC
CTTCAACGGCCTCAAGAACCAGAACAGGAACCTGAAGACCCTGCTGGCCATTGGAGGAT
GGAATTTCGGCACAGAGAAGTTCACCACCATGGTTTCCACACCCCAGAACCGCCAGACC
TTCATCAACTCCGTGGTCAGGTTCCTGCGCCAGTACGGATTCGATGGGCTGGACCTGGA
CTGGGAATACCCCGGCTCCAGGGGCAGCCCAGCCCAGGACAAGGGTCTCTTCACCGTCC
TGGTTAAGGAACTGCTGGCAGCCTTCGAGCAGGAAGCCAAACAGACCAACCGGCCCCGG
CTCATGGTCACCGCCGCTGTGGCCGGAGGACTTTCCACCATCCAAGCTGGCTACGAGAT
TGCTGAGTTGGGCAAGTACCTGGATTACATCCACGTGATGACCTATGACTTCCACGGGC
CCTGGGACGGCTCCACGGGCGAGAACAGCCCCCTGTTCAGTAGCGGCACCCTCAGTGTT
GAATACGCCATGAACTACTGGAAGAACAACGGCGCCCCAGCTCAGAAGCTCCTGGTGGG
ATTCCCAACCTACGGGAAAACCTTCACCCTGCAGAACCCATCCAACACCGGCATCGAGG
CCCCAACCTCCGGCCCTGGTCCTGCGGGACCCTACACCAAGGAGGCCGGGCTCTTGGCT
TACTACGAGATCTGCACCTTCCTGAACTCTGGAGCCACCCAGGCTTGGGATGAACCCCA
GGACGTGCCCTACGCCTACAAGGGCAGCGAATGGATCGGCTACGACAACGTCAAGAGCT
TCGGCCTCAAGGTGGATTGGCTGAAGAAGAACAACTTTGGCGGGGCCATGGTCTGGACC
ATCGACCTGGATGACTTCACCGGCACCTTCTGCCACGAGGGCAAATACCCCCTGATCTC
CACGCTGAAGAAGGGCCTGGGGCTG
>Corvus_moneduloides1
ATGGCCAAGCTCACTCTGCTCACCGGCACTGCCTATGTGCTGACCTGTTACTTCACCAA
CTGGGCCCAGTACAGGCCTGGCCTGGGCAAGTACACCCCCGAAAATGTCGACCCCTGCC
TGTGCAACCACCTGATCTACGCCTTCGCCGGCATGAACAACAACGAGATCACCACCTAC
GAGTGGAACGATGAGACCCTGTACAAGTCCTTCAACGGCCTCAAGAACCAGAACAAAGA
CCTGAAGACCCTGCTGGCCATTGGAGGATGGAATTTTGGCACAGCCAAGTTCACCACCA
TGGTTTCCACACCCCAGAACCGCCAGACCTTCATCAACTCCGTGGTCAGGTTCCTGCGC
CAGTACGGATTCGATGGGCTGGACCTGGACTGGGAATACCCCGGCTCCAGGGGCAGCCC
AGCCCAGGACAAGGGTCTCTTCACCGTCCTGGTTAAGGAACTGCTGGCAGCCTTCGAGC
AGGAAGCCAAACAGACCAACCGGCCCCGGCTCATGGTCACCGCCGCTGTGGCTGCCGGA

CTTTCCACCATCCAGGCTGGCTACGAGATTGCTGAGATTGGCAAGTACCTGGATTACAT
CCACGTGATGACCTACGACTTCCACGGCTCCTGGGAGAGGAACACTGGTGAGAACAGCC
CCCTGTTTGCTGGCCCTGCTGACACTGGGGACTACAAATACTTCAACGTTGAATATGCC
ATGAATTATTGGAAGAGCAACGGTGCCCCAGCTGAGAAGCTCCTGGTGGGATTCCCAAC
CTATGGAAAGAGTTTCACCCTGCAGAACCCATCTGACACCTCCGTTGGAGCTCCAGCGT
CCGGCCCTGGCCCAGCTGGGCCCTACACCAGGGAGGCCGGAACCCTGGCTTACTACGAG
ATCTGCACCTTCCTGAGCTCCGGAGCCACCCAGGCTTGGGATGAACCCCAGGACGTGCC
CTACGCCTACAAGGACAGCGAATGGGTCGGCTACGACAACGTCAAGAGCTTCGGCCTCA
AGGTGGATTGGCTGAAGAAGAACAACTTTGGCGGGGCCATGGTCTGGGCCCTGGACATG
GATGACTTCACCGGCACCTTCTGCCACGAGGGCAAATACCCCCTGATCTCCACGCTGAA
GAAGGGCCTGGGGCTGGAGAGCGGCGACTGTGTTCCCCCCGCTGAGCCCCTGCCTCCCA
TCACCGAGGCTCCCACCACCACCACCACCACCAGCGGTGGCTCCGGCGGCTCCGGCGGC
TCCGGCGGCAGCGGCTTCTGCGCCGGGAAACCCAACGGGATCTACGCAGACCCCGACAA
CAAGAGGAACTTCTACAGCTGCGTGAACGGGCAGACCTTCGTGCAGAGCTGTGAGCAGG
GGCTGGTCTTCGACCCCGTCTGCTCCTGCTGCAACTGGCCCCAG
